# Supplementary material for: Airborne eDNA captures three decades of ecosystem biodiversity
Source: Nat Commun. 2025 Dec 18;16:11281. doi: 10.1038/s41467-025-67676-7 (PMC12717267; doi:10.1038/s41467-025-67676-7)
Supplement: Supplementary file 1 — Supplementary information [file 41467_2025_67676_MOESM1_ESM.pdf]

Supplementary Information for

Airborne eDNA captures three decades of ecosystem biodiversity

Alexis R. Sullivan*, Edvin Karlsson*, Daniel Svensson, Björn Brindefalk, Jose Antonio Villegas, Amanda Mikko, Daniel Bellieny, Abu Bakar Siddique, Anna-Mia Johansson, Håkan Grahn, David Sundell, Anita Norman, Per-Anders Esseen, Andreas Sjödin, Navinder J Singh, Tomas Brodin, Mats Forsman and Per Stenberg

*: These authors contributed equally.

Corresponding author: per.stenberg@umu.se

The PDF file includes:

Supplementary Methods
Supplementary Figures 1 to 26
Supplementary Tables 1 to 8

Other Supplementary Information for this manuscript include the following:

Supplementary Data 1 to 13

Table of Contents

Table of Contents 2

Supplementary Methods 5

Summary of supplementary methods 5

Supplementary Figure 1. Brief summary of the analysis workflow. 6

1. Aerosol sampling station and catchment areas 7

1.1. Ecological context 7

Supplementary Figure 2. Land cover around the aerosol sampling station from the Swedish National Landcover Database (NMB). 8

1.2. Aerosol sampling 9

Supplementary Figure 3. The Kiruna aerosol sampling station. 9

1.3. Atmospheric modeling 10

1.3.1. Catchment area estimation 10

Supplementary Figure 4. Particle dispersion bootstrapping and Monte Carlo simulation results 11

Supplementary Table 1. Catchment area shape, two-way ANOVA test results. 12

Supplementary Table 2. Catchment area linear mixed-effect model results. 12

Supplementary Table 3. Weekly variation, two-way ANOVA test results 13

1.3.2. Estimation of geographic origins of cesium-137 and Alces, Gadus, and Rangifer eDNA 13

Supplementary Figure 5. Comparison of cesium-137 deposition patterns and inferred source areas 14

Supplementary Figure 6. Number of HYSPLIT back-trajectory segment endpoints per 0.1° grid cell over the entire 1974-2008 time series. 15

Supplementary Figure 7. Potential source areas for weekly cesium-137 measurements taken at the aerosol sampling station between 1996-2006. 16

Supplementary Figure 8. Potential source areas of reindeer (Rangifer tarandus) eDNA captured by the aerosol sampling station during summer weeks. 19

Supplementary Figure 9. Potential source areas of reindeer (Rangifer tarandus) eDNA captured by the aerosol sampling station during non-summer weeks. 20

Supplementary Figure 10. Potential source areas of Atlantic cod (Gadus morhua) eDNA captured by the aerosol sampling station. 22

Supplementary Figure 11. Potential source areas of moose (Alces alces) eDNA captured by the aerosol sampling station. 24

2. DNA sequencing 25

2.1. Extraction 25

2.2. Sequencing 26

Supplementary Figure 12. Number of paired-end reads sequenced from each weekly air filter. 27

Supplementary Table 4: Output of PERMANOVA tests for differences in Extraction batch and Sequencing batch, based on Euclidian distances using centered log-ratio transformed data. 27

3. Bioinformatics pipeline 28

3.1. Read preprocessing and filtering 28

Supplementary Figure 13. Human read removal. 28

3.2. Taxonomic read classification 28

3.2.1. Custom Kraken 2 database 29

3.2.2. Kraken 2 classification and filtering with StringMeUp 29

Supplementary Figure 14. A larger fraction of reads are classified to taxa reported in Torne lappmark whereas classification success decreases with increasing stringency cutoffs in Kraken 2. 30

4. Relative abundance transformations and detrending 31

4.1. Removal of zero inflated taxa and log-ratio transformations 31

4.2. Detrending 33

Supplementary Figure 15. Proportion of human reads and mean read length. 33

Supplementary Figure 16. Comparison of relative abundances before and after detrending. 34

4.3. Classification refinement with gradient boosting 34

4.3.1. Feature engineering 34

4.3.2. Training data acquisition 36

Supplementary Table 5. Taxonomic composition of training data 37

4.3.3. Parameter tuning and classification 37

Supplementary Figure 17. Distributions of the four most influential features in the gradient boosting machine classification model. 38

Supplementary Table 6. Gradient boosting machine (GBM) classification performance 39

4.4. Alignment-based validation of classified taxa 39

Supplementary Figure 18. Summary of methods used to validate genus classifications. 40

4.4.1. Genera selection 40

Supplementary Figure 19. Selection criteria met by the 66 genera subjected to read-level validation. 41

4.4.2. Read mapping and consensus calling 41

4.4.3. BLAST searches 41

5. Dimensionality reduction and clustering 42

5.1. Taxa-based clustering and ordination 42

5.2. Control taxonomic composition 42

Supplementary Figure 20. The relative number of reads and taxonomic composition in controls and real samples. 43

6. Diversity metrics 44
6.1. Per-taxon γ-diversity contributions 46
7. Climatic variables 46
7.1. Data sources and construction 46
Supplementary Table 7. Summary of climatic covariables. 47
7.2. Variable selection 49
7.3. Missing year interpolation 49
Supplementary Figure 21. Climatic covariables with imputed values for 1994. 50
7.4. Variable clustering and categorization 50
8. Time series analysis 51
8.1. Introduction to state-space models 51
8.2. eDNA abundance and diversity trends 52
8.2.1. Structural time series models 52
8.2.2. Prior distribution specifications 53
Supplementary Table 8. Prior distributions used in the production models. 55
8.2.3. Model fit and convergence diagnostics 55
8.2.4. Leave-future-out cross validation 56
8.3. Abundance trends from traditional monitoring data 56
8.3.1. Data acquisition 56
8.3.2. State space models 57
Supplementary Figure 22. Scaled annual abundances indices for eight bird genera estimated from point surveys (orange) and PLR transformed eDNA (blue) 58
9. Land use 59
Supplementary Figure 23. Forest harvests outside formally protected areas in northern Sweden. 59
9.1. Forest management 60
Supplementary Figure 24. Land cover from the Swedish National Land Cover Database (NMD) and formally protected areas (hatched lines) within 350 km of the aerosol station 61
Supplementary Figure 25. Forest age and standing biomass in northern Sweden. 62
Supplementary Figure 26. Comparison of trends in pine forest cover and pine-associated eDNA abundance. 63
References 64

Supplementary Methods

Summary of supplementary methods

DNA was extracted from weekly air filters sampled in even-numbered years from 1974 to 2008 by a radionuclide aerosol sampling station in northern Sweden. DNA isolates from each week were shotgun sequenced on their own Illumina NovaSeq 6000 S4 flow cell. Reads were subjected to quality control and then taxonomically classified using a large custom reference database. Classification stringency parameters were optimized for genus-rank using publicly-available species observations made in the vicinity of the aerosol sampling station. Read counts per genus were log-ratio transformed and detrended to remove potential biases from *e.g.,* read length variation. The last processing step removed putative false positive taxa using a novel machine learning approach (supplementary fig. 1).

The final 2,739 high confidence genera were then clustered based on shared temporal patterns. For this analysis we used samples from weeks 21-41 since weeks outside this range did not produce sequencing libraries for a majority of the years. Time series analysis of the resulting 17 cluster abundances, community diversity components, and of individual genus abundances was performed using Bayesian state space models. The relative predictive power of covariables representing endogenous seasonal patterns, variation in climate and weather-related parameters, and weekly changes in the aerosol station's catchment area were compared using leave-future out cross validation.

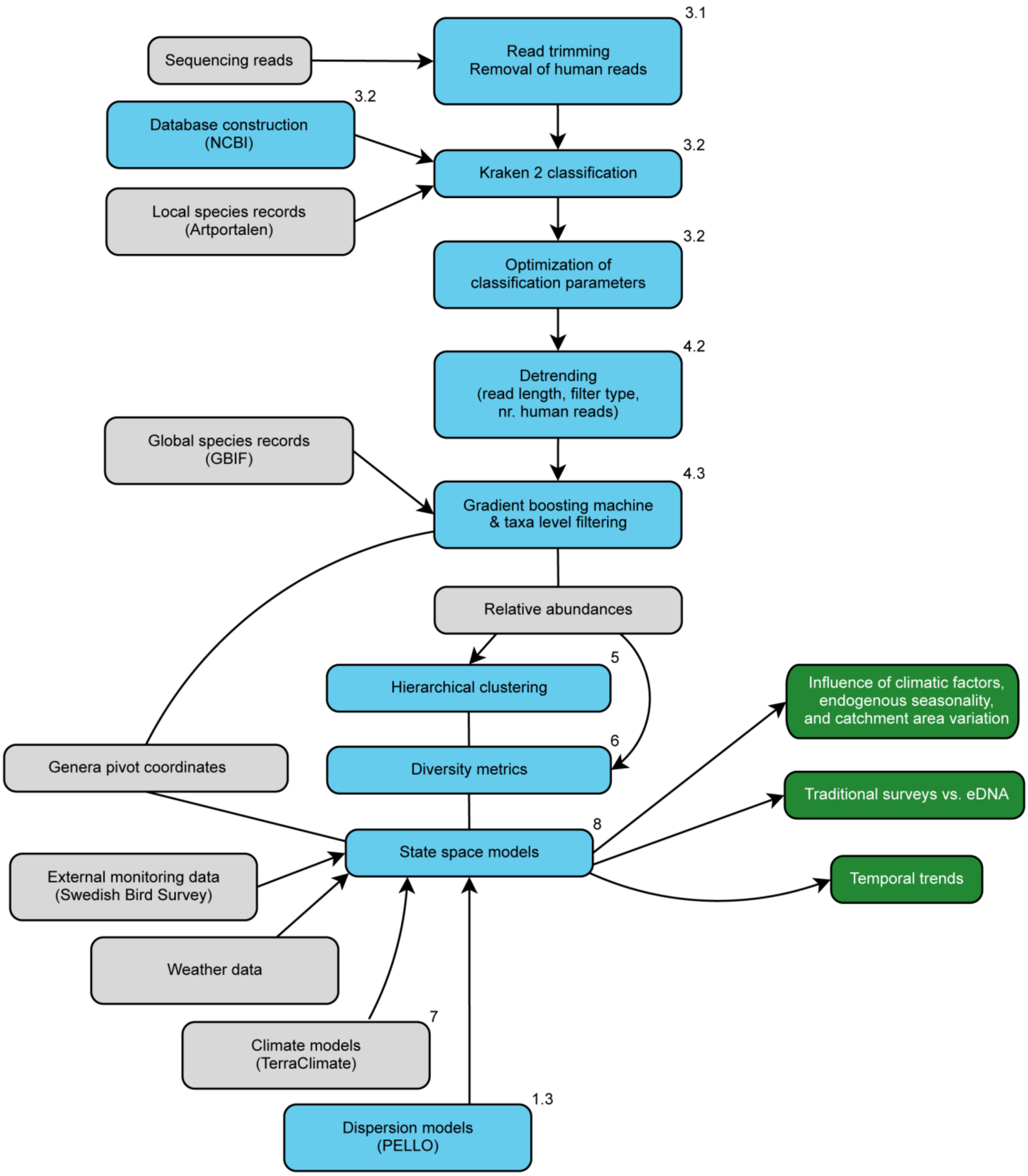

Supplementary Figure 1. Brief summary of the analysis workflow.
For more details of each step, see the indicated section.

1. Aerosol sampling station and catchment areas

1.1. Ecological context

The aerosol sampling station is located in the northern boreal zone in the province of Norrbotten, the northernmost in Sweden, *ca*. 9 km east of the mining town of Kiruna (population 23,000, 67.84°N, 20.42°E) and *ca*. 2 kilometers northeast of Kiruna airport (a low-volume airport with on average 7 landings per day). Data on land cover were extracted from the Swedish National Land Cover Database[1], mapped in 2017-2019. The data consist of a base map with 25 thematic classes in three hierarchical levels and has a raster format with 10 m pixel size. Using ArcGIS *v*. 10.3, we extracted the area and proportion of land cover classes within 50, 20, 5, 0.5, and 0.1 km radius of the aerosol sampling station. Thematic classes were aggregated into nine classes. Forests outside and on wetlands were not separated.

Land cover within 50 km was dominated by vegetated open land (39%; mainly low and middle alpine belts), open wetland (20%), coniferous forests (15%), deciduous forests (11%), mixed forests (6%), and water (6%; supplementary fig. 2). Minor classes included temporarily deforested land (1.8%; clearcuts), open land without vegetation (0.8%; mainly high alpine belt), and artificial vegetation-free surfaces (0.7%; e.g., mining areas, building, and road/railway). Agriculture was uncommon. Forested area increased from 32% at the 50 km scale to 64% at the 0.5 km scale with a commensurate decrease in open habitats. Land cover within 0.1 km of the aerosol sampling station was composed of 75% forest, 12% open wetland, 8% artificial surfaces, and 5% other land cover. A large, contiguous network of formally-protected nature reserves spans much of the subalpine zone west of the aerosol sampling station (see Section 9).

In 2020, we inventoried a total of eleven 10 m radius plots located at 25 (four plots) and 100 m (seven plots) distance from the station. We recorded the diameter at breast height (DBH; 1.3 m) and species of trees with DBH $\geq$ 10 cm and calculated basal area per hectare. At this scale, the forests were dominated by pine (71% of basal area), followed by spruce (18%), and birch (11%). They were old, multi-layered, and semi-open (mean basal area 15 m^2 ha^{-1}). The forests had a semi-natural character, with a few old stumps indicating past selective logging. Understory vegetation was dominated by dwarf shrubs (*Empetrum nigrum* ssp. *hermaphroditum*, *Vaccinium myrtillus*, *V. vitis-idaea*) and bryophytes (*Hylocomium splendens, Pleurozium schreberi*), with patches of terricolous lichens (e.g., *Cladonia* spp., *Nephroma arcticum*, *Peltigera* spp.).

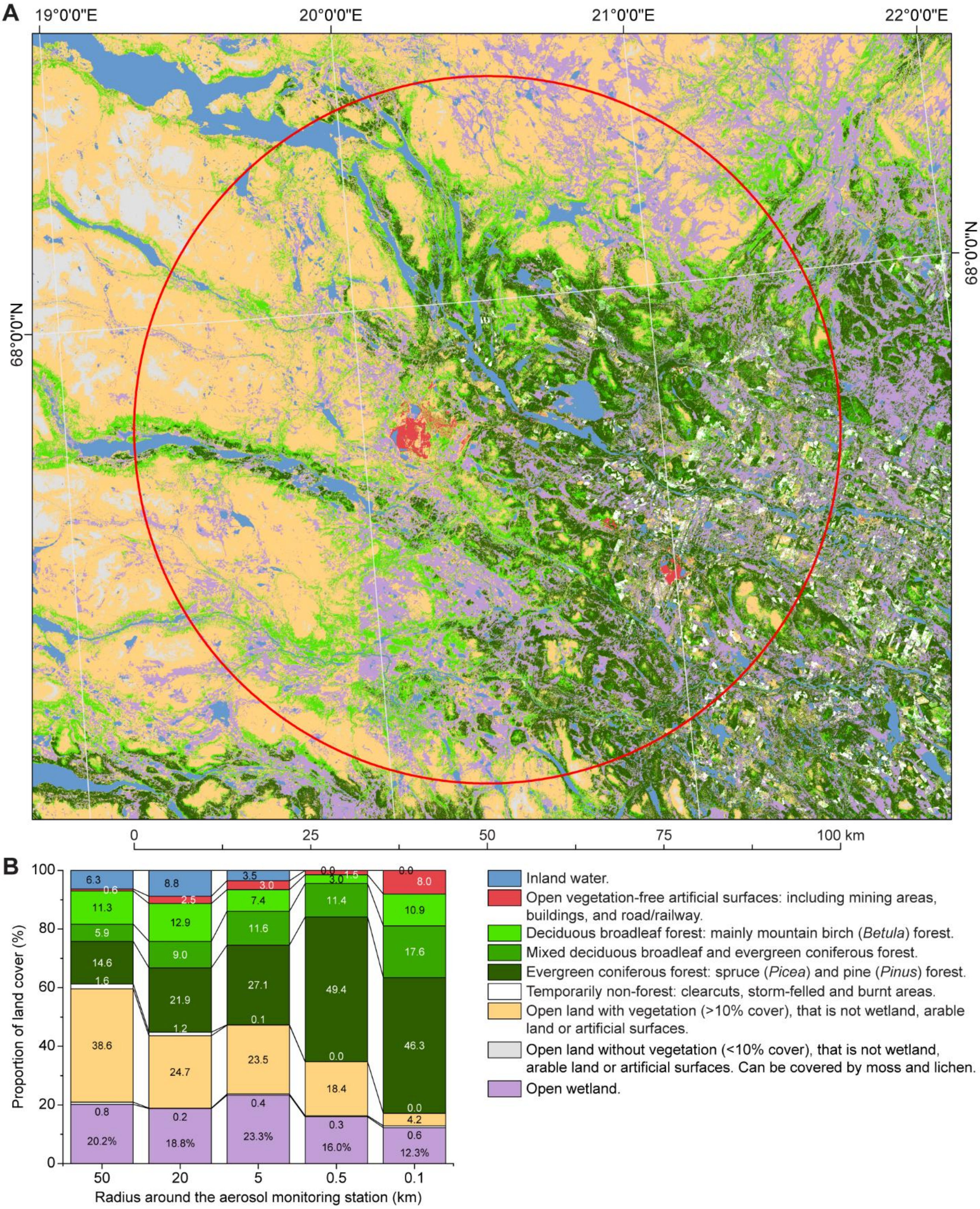

Supplementary Figure 2. Land cover around the aerosol sampling station from the Swedish National Landcover Database (NMB).

A) Map of nine land cover classes in a 50 km buffer around the aerosol sampling station. **B**) Relative composition (%) of land cover at five different spatial scales (50 km to 0.1 km). Based on land cover data with 10 x 10 m pixel size.

1.2. Aerosol sampling

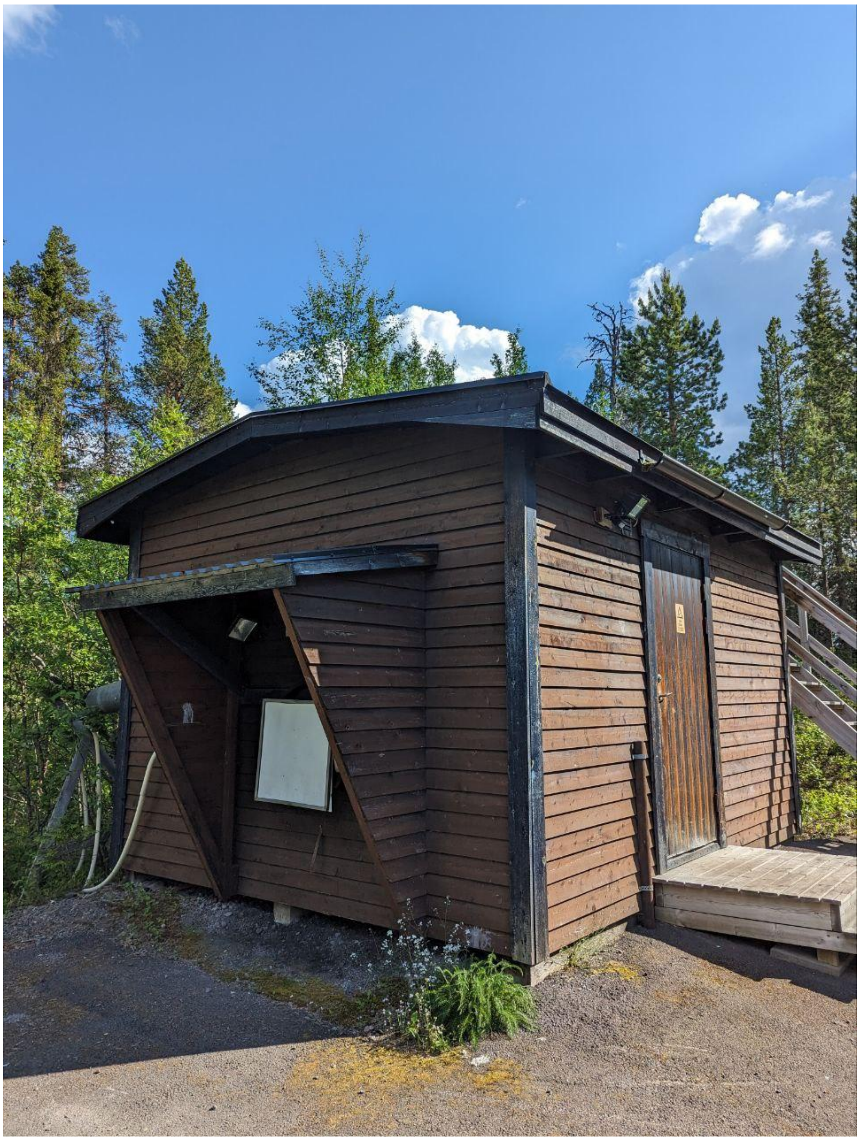

Supplementary Figure 3. The Kiruna aerosol sampling station.
The building houses an air pump that forces air through the external aerosol filter mounted on the wall. Photo: Daniel Svensson.

Air filters were collected once a week between 1974 and 2008 by the Swedish Defense Research Agency (FOI[a]) to monitor radioisotopes in surface level aerosols[2]. The filters belong to a larger collection spanning the five decades of continuous and ongoing radionuclide surveillance at stations across Sweden. Each station consists of a small building housing an air pump that forces air through an external 60x60 cm aerosol filter mounted on the wall of the building approximately 1 meter above ground (supplementary fig. 3). Filters are made of glass fiber with a pore size of 0.2 µm and filter more than 100,000 m^3 of air each week. The manufacturer changed in 1996 (from Camfil type CS 5.0, Camfil Svenska AB, to HB5773, Hollingsworth & Vose Company Ltd.), but the new filters were produced with the same

[a] Totalförsvarets forskningsinstitut

specifications. We detrended the sequence data (section 4.2 Detrending) to account for potential effects of the filter manufacturer change. From 1976-1984, filters were stored in airtight rectangular plastic containers and in cylinder shaped containers in all other years. We selected weekly air samples from every other year between 1974 and 2008. For DNA extraction we use ~5% of the total filter sample, representing approximately 5,000 m^3 of air. We attempted DNA extraction from filters installed during weeks with a mean temperature > 0°C because aerosol DNA concentrations are low during freezing conditions[3]. Before DNA extraction all samples were assigned a random number. DNA extraction, sequencing library construction and multiplexing in the sequencing was performed in batches according to the random sample IDs. This was done to avoid systematic batch-effects in the data.

1.3. Atmospheric modeling

1.3.1. Catchment area estimation

Bioaerosols are airborne particles released into the atmosphere such as fungal spores, bacteria, pollen, and shed cells. During their journey to an aerosol sampler, bioaerosols undergo processes such as deposition and coagulation and interact with atmospheric moisture as they are carried by complex and chaotic wind patterns. These processes determine the spatial extent of sources sampled by the aerosol station, which we refer to as catchment areas.

We employed PELLO[4], a random displacement Lagrangian particle model validated[5] and applied in several studies,[6–8] to estimate catchment areas. PELLO is normally used in applications with some basic knowledge of the source (*i.e.*, position and characteristics of the pollutant released in the atmosphere), but we lacked two important source properties: position and time. The straightforward solution to this problem is to define a large number of sources covering the entire calculation domain both in time and space and then keep track of all aerosols that enter the filter station. This was unfeasible in our scenario due to the large number of sources we would have to define, and hence the large number of model particles to handle, to cover the region of interest in time and space. Our approach was therefore to use an adjoint version of PELLO where model particles advected with wind and dispersed due to turbulence backward in time from the aerosol station to their origin. This backward simulation let us define only one source, but spread in time, which shortened the computation time by several orders of magnitude.

PELLO models particle transport with data from numerical weather predictions (NWP) from the European Centre for Medium-Range Weather Forecasts (ECMWF). For this study, we used the ERA-5 dataset[9,10] (1980-2008, except 1994 as the data for that year could not be retrieved) with a 1.0 x 1.0° horizontal resolution and a vertical resolution of 79 hybrid sigma pressure levels (in ERA-5, this is the lowest 16 km of the atmosphere). We used a 6 and 12 hour forecast step starting at 06:00 and 18:00, resulting in four forecast fields per day. The spatial domain of the weather data covered Europe, including western part of Russia and Northern Africa. Aerosol dry and wet deposition were modeled but no other biological or chemical particle properties were incorporated.

As a source for the adjoint dispersion, we used particles with diameters of 5, 22, and 60 µm and a density of 800 kg/m^3 [11], representing smaller fungal spores or bacterial aggregates[12–14], birch pollen, and pine pollen, respectively. The spatial domain of the release

of bioaerosol for the adjoint dispersion was defined with a horizontal domain of 30 x 30 m and a vertical domain stretching from 0-300 m, roughly corresponding to the planetary boundary layer (PBL) in a neutral atmosphere. The source domain represents a ground source on the regional scale where the bulk of the bioaerosols are well mixed in the PBL. Although we only modeled three different particle diameters, we expect it to provide a rough estimation of the catchment area within this regional context.

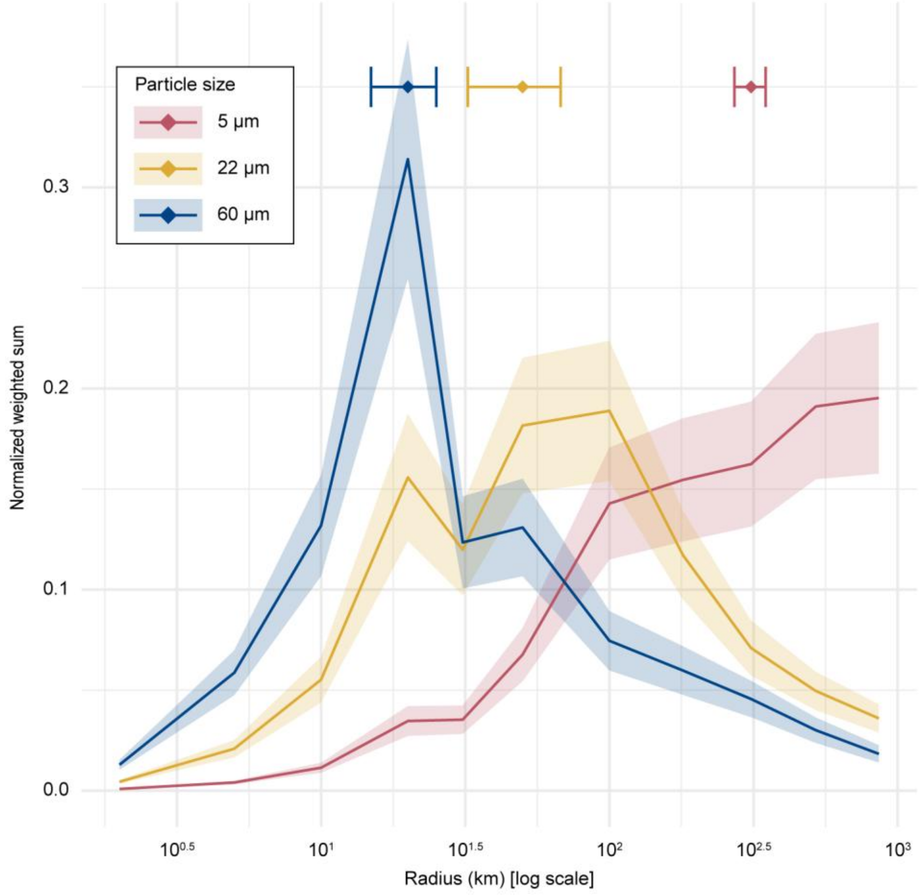

Supplementary Figure 4. Particle dispersion bootstrapping and Monte Carlo simulation results.

Normalized weighted sums (*i.e.*, contributions from various distances closed to one) plotted against the distance (log-scale) from the aerosol sampling station, color-coded according to particle size. Shaded areas correspond to the normalized standard error obtained from the bootstrap procedure for each particle size. Horizontal error bars (standard deviation) and data-points at top of plot correspond to the results of the Monte Carlo simulation equal to 50% of cumulative particle mass from all directions, color-coded according to particle size, using the block bootstrapping as input.

We summarized the spatial extent of the catchment areas and the proportion of particles originating from eight cardinal directions. The particle mass originating from different distances from the aerosol sampling station (2, 5, 10, 20, 31, 50, 100, 180, 310, 520, and 860 km) was calculated for each week in the even-numbered years from 1980 to 2008, excluding 1994 (supplementary data 1). For each particle size, the cumulative mass within each radius was scaled by the sum within the 860 km radius area (supplementary fig. 4). Yearly averages of the proportion of particle mass originating from each cardinal direction

were also calculated for each distance (supplementary data 1). These weekly sums for the 22 µm particle size were used as regression covariates in the time series analysis in Section 8 to assess the potential influence of changes in bioaerosol sources on weekly eDNA compositions.

To assess the range of particle dispersion and its associated uncertainty, block bootstrapping with *R* package 'boot' *v.* 1.3-28.1[15,16] was employed. Each bootstrap replicate consisted of 1,000 resamples with a block size of four weeks, approximating a lag of one month. The bootstrapped data were then normalized using weighted sums (supplementary fig. 4). To identify the 50% cumulative particle mass originating from all directions, a weighted Monte Carlo simulation was conducted using the normalized weighted sums and their standard errors as input parameters over 1,000 draws.

To determine if the shape of the catchment area varied between particle sizes, the weekly dispersion for each particle size was scaled and centered and tested with two-way ANOVA according to the formula: dispersion ~ particle size × cardinal point. The interaction term between particle size and cardinal direction (i.e., the shape of the catchment area for the different particle sizes) was not found to be significant (supplementary table 1).

Supplementary Table 1. Catchment area shape, two-way ANOVA test results.

Two-way analysis of variance, testing the effects of particle size and cardinal direction on catchment-area shape, followed by Tukey post-hoc comparisons. Reported are degrees of freedom (*df*), sums of squares (SumSq), mean squares (MeanSq), *F*-values, and *P*-values.

Term	*df*	SumSq	MeanSq	F-value	*p*-value
Particle size	1	5.53e-16	5.53e-16	246.11	<2e-16
Cardinal direction	7	7.59e-17	1.08e-17	4.83	2.5e-05
Particle size × Cardinal direction	7	5.4e-18	8.0e-19	0.35	0.93
Residuals	728	1.64e-15	2.2e-18		

Supplementary Table 2. Catchment area linear mixed-effect model results.

Two-sided linear mixed-effects model, testing year-to-year variation in the shape of the catchment area (22 µm particle size). Reported values are fixed-effect estimates, standard errors, degrees of freedom (*df*), *t*-values, and *P*-values where applicable. No multiple-comparison correction was applied.

Effect	estimate	std. error	*df*	*t*-value	*p*-value
Intercept	0	0.86	1204	0.00	1.00
year (fixed effect)	0	0.00	12	0.01	0.99
cardinal directions (fixed)	not shown individually				n.s.
year × cardinal direction	not shown individually				n.s.

To evaluate the year-to-year variation in the shape of the catchment area for the even years between 1980 and 2008, a linear mixed-effects model was implemented using the R package 'nlme' *v.* 3.1-163[17,18]. The dependent variable was the scaled particle mass value, normalized to sum to one. Fixed effects included the year and the cardinal direction, as well as their interaction. A random intercept for the year was included to account for repeated measures, along with a first-order autoregressive correlation term to handle autocorrelation. The mixed-effects model indicated no significant year-to-year variation in the shape of the

catchment area across the studied period for any of the particle sizes (data shown for 22 µm, supplementary table 2).

Although the catchment area shape does not vary significantly between particle sizes or across the time series on a yearly basis, local weather conditions influence particle dispersion values on a weekly basis (as tested with two-way ANOVA, according to the formula dispersion ~ week × particle size). Furthermore, local conditions affect different particle sizes differently, as would be expected from larger particles being less sensitive to wind and other atmospheric conditions due to faster settling times (supplementary table 3).

Supplementary Table 3. Weekly variation, two-way ANOVA test results.

Two-way analysis of variance, testing weekly variation in particle dispersion as a function of week and particle size. Reported are degrees of freedom (*df*), sums of squares (SumSq), mean squares (MeanSq), *F*-values, and *P*-values. No multiple-comparison correction was applied.

**Term**	***df***	**SumSq**	**MeanSq**	**F-value**	***p*-value**
Week	31	1.33e-14	4.28e-16	56.7	<2e-16
Particle size	2	6.74e-14	3.37e-14	4465	<2e-16
Week × Particle size	62	3.01e-15	4.86e-17	6.43	1.37e-50
Residuals	114480	8.64e-13	7.55e-18	NA	NA

1.3.2. Estimation of geographic origins of cesium-137 and **Alces, Gadus,** *and* **Rangifer** *eDNA*

Dispersion models are powerful tools for simulating the transport and deposition of specific particles based on known aerodynamic properties. However, when bioaerosol properties are unknown, as is the case for most airborne eDNA (except for well-characterized pollen and spores), back-trajectory models provide an alternative method for estimating potential source regions. Trajectory ensemble receptor models (TERMs) compare temporal variation of particle concentrations (*e.g.*, eDNA) to the history of air parcels arriving at the sampling site. TERMs have two distinct components: 1) a grid of back-trajectory endpoints calculated by an atmospheric transport, and 2) a statistical model to relate these endpoints to the observed particle concentrations[19,20].

We calculated back-trajectories using HYSPLIT (Hybrid Single-Particle Lagrangian Integrated Trajectory), a widely used atmospheric transport and dispersion model. Back-trajectories were started every 6 hours from the date the air filter was removed back until the date it was installed and followed 24, 48, and 72 h into the past, with starting heights of 10, 100, 300, and 500 meters. Archived meteorology from the NCAR/NCEP Reanalysis project[21] was used, which has lower horizontal and vertical resolution than the ERA-5 dataset used for dispersion modeling but spans the entire filter archive.

We then applied simplified quantitative transport bias analysis (SQTBA)[22–24] to compare the HYSPLIT back-trajectory endpoints to weekly PLR-transformed eDNA relative abundances (see Sections 2-4). In brief, SQTBA considers the HYSPLIT trajectories as centerlines in a Gaussian plume dilution model, which results in a gridded probability distribution of trajectories for each week. This is weighted by the corresponding eDNA abundances to obtain a gridded spatial concentration field. SQBTA was conducted using the `trajLevel` function in "OpenAir" R package version 2.18-2[25].

We applied TERMs to eDNA from three vertebrates: moose (*Alces alces*), a forest browser, cod (*Gadus morhua*), a demersal marine fish, and reindeer (*Rangifer tarandus*), a migratory browser-grazer. These genera were chosen because their spatial distributions are distinct and well-defined, allowing us to compare the inferred source regions to specific hypotheses. That is, if the aerosol sampling station captures vertebrate eDNA emissions at least at a regional scale (*ca*. 50 km[b]), then source regions for moose eDNA should overlap with the boreal forest, the Norwegian and/or Barents seas for cod, and seasonal grazing areas (see Section 9) for reindeer. Cod provides an additional sanity check, as a non-marine source estimate could indicate contamination or a methodological error in some step of our analysis. For moose and cod, we estimated TERMs for each combination of starting height (10, 100, 300, 500 m) and duration (24, 48, 72 hr) using eDNA relative abundances over the study duration. For reindeer, we estimated TERMs separately for summer (weeks 24–36, roughly mid-June to early September) and non-summer (weeks 21–23 and 37–41) periods.

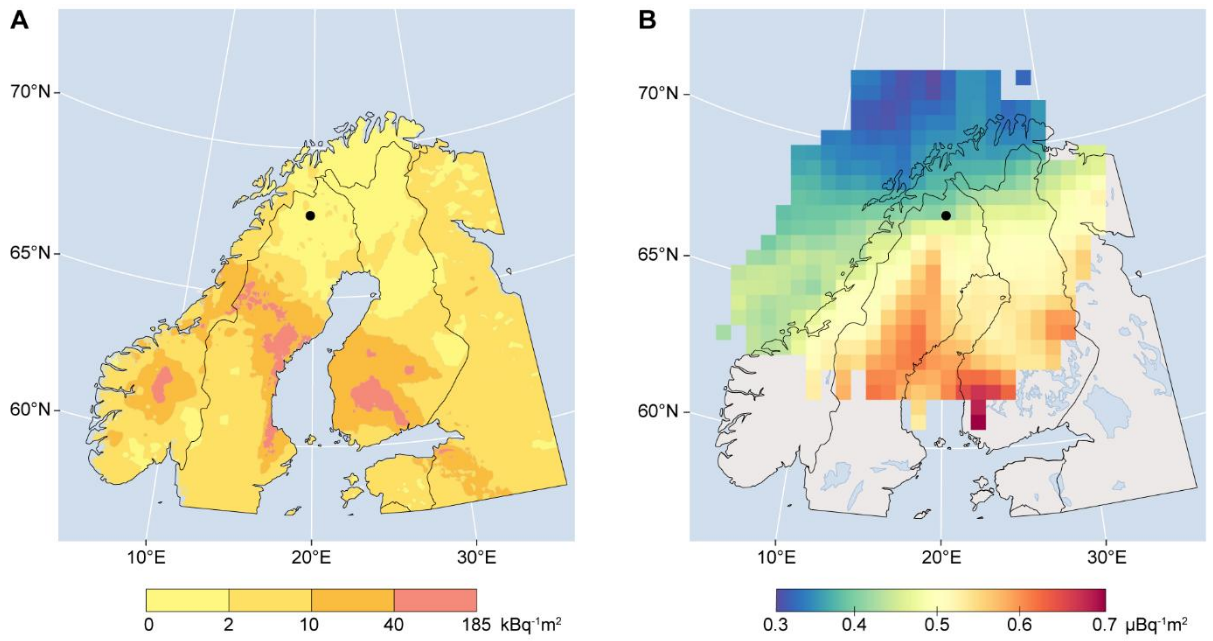

Supplementary Figure 5. Comparison of cesium-137 deposition patterns and inferred source areas.

A) Total cesium-137 deposition across Fennoscandia following the 1986 Chernobyl nuclear power plant accident[26]. **B**) Potential source areas for weekly cesium-137 measurements from the aerosol sampling station. Source areas were inferred using simplified quantitative transport bias analysis (SQTBA) applied to 48-hour, 300 m back-trajectories calculated with HYSPLIT. The location of the aerosol sampling station is indicated by a black dot. Map from Natural Earth.

For comparison, we fit the same TERMs to weekly measurements of cesium-137 (one of the radionucleotides measured as part of the aerosol stations' routine operations), a nuclear fission product, from the same filters used for eDNA sequencing. In Europe, the primary source of cesium-137 is fallout from the Chernobyl disaster in 1986, with deposition occurring primarily by precipitation, leading to a highly heterogeneous spatial pattern across Fennoscandia (supplementary fig. 5A; European Commission 1998[26]). Because airborne

[b] A minimum estimate, given the average windspeed in Kiruna (8 km h^{-1}) and the temporal resolution (6 hr) of the meteorology

cesium-137 originates from resuspension of contaminated soil and emissions from contaminated biomass[27], it provides an independent reference for evaluating long-range transport patterns. To minimize the effects of radioactive decay ($t_{1/2}$ = 30.17 years), we used non-winter cesium-137 measurements from 1996 and 2006.

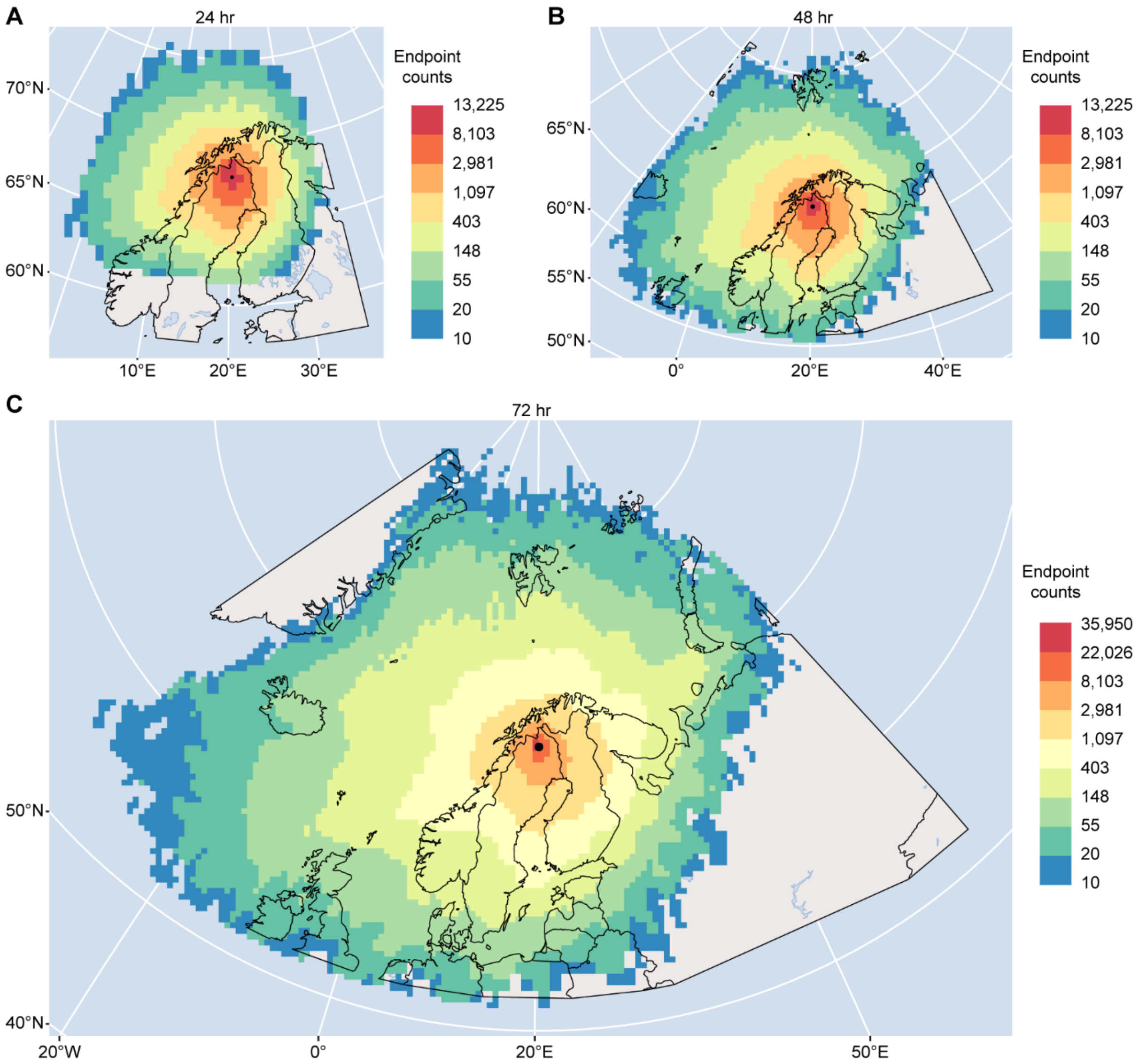

Supplementary Figure 6. Number of HYSPLIT back-trajectory segment endpoints per 0.1° grid cell over the entire 1974-2008 time series.

Endpoint counts are shown for **A**) 24-hour, **B**) 48-hour, and **C**) 72-hour back-trajectories, respectively. Back-trajectories were initiated every six hours at 100 m above ground level (AGL). Map from Natural Earth.

Supplementary fig. 6 illustrates the geographic regions most frequently traversed by air masses on their way to the aerosol sampling station. These trajectory endpoints are qualitatively similar to the catchment areas estimated by dispersion models in PELLO (see also Section 1.3.1). This result is expected but reassuring: despite using different models and meteorological datasets, both approaches broadly agree on the movement of airmasses within the PBL.

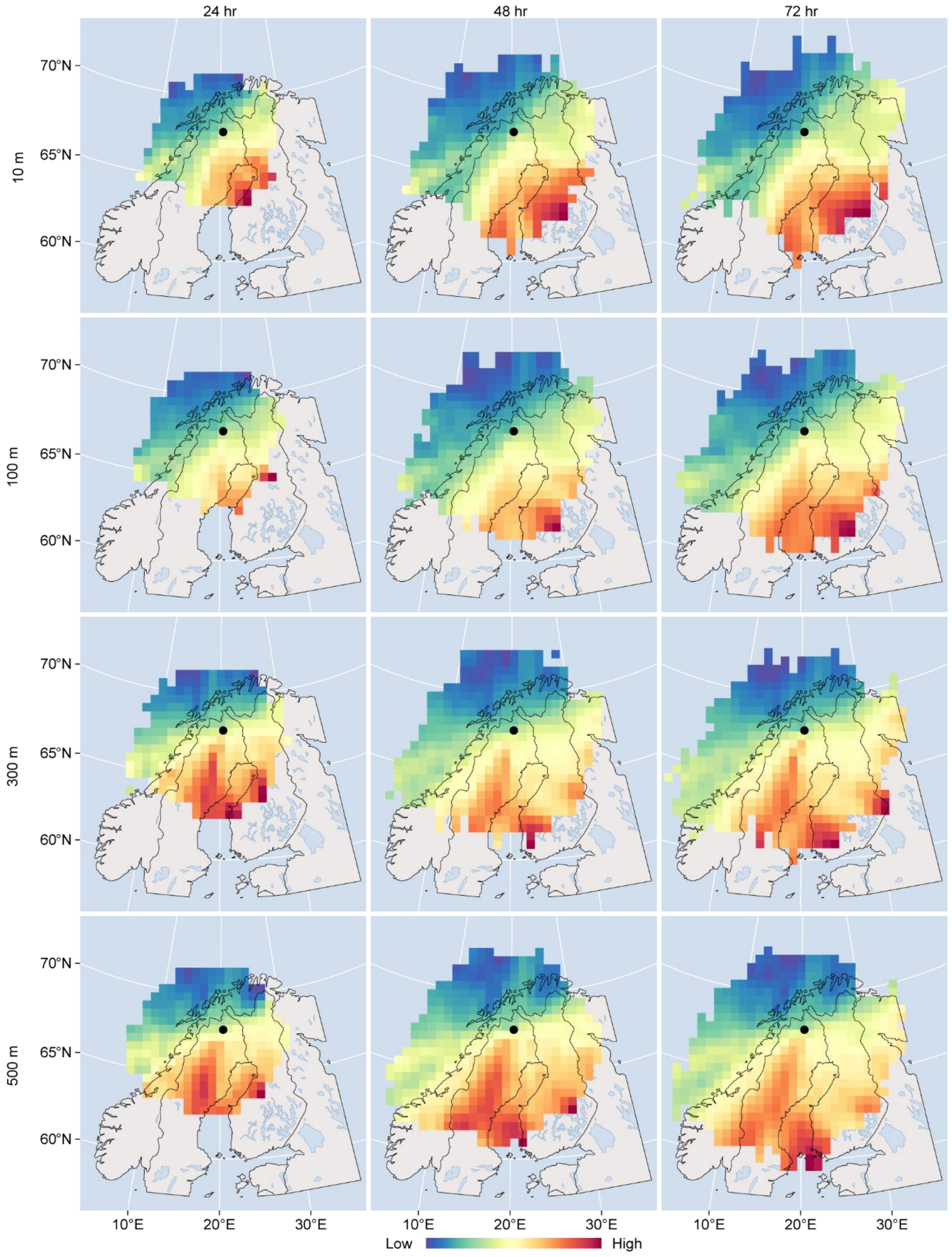

Supplementary Figure 7. Potential source areas for weekly cesium-137 measurements taken at the aerosol sampling station between 1996-2006.

Source areas were inferred using simplified quantitative transport bias analysis (SQTBA) applied to HYSPLIT back-trajectories initiated at 10, 100, 300, and 500 m above ground level (AGL), in rows, with durations of 24, 48, and 72 hours, in columns. The location of the aerosol sampling station is indicated by a black dot. Colours represent signal strength, with warmer tones (red–yellow) indicating stronger potential sources and cooler tones (blue–green) indicating weaker ones. Map from Natural Earth.

Back-trajectory duration is a key TERM parameter: longer trajectories could potentially detect more distant source regions, but horizontal error rates increase with trajectory length, from *ca.* 15% at 24 hrs to 35% at 72 hrs[28]. For SQBTA, we limited the source estimates to grid cells crossed by ≥ 2.5% of trajectories to minimize the influence of grid cells with few counts[19,23].

Source regions for cesium-137 estimated by SQTBA were broadly consistent with the spatial pattern of Chernobyl fallout (supplementary fig. 5). Concentrations measured at the aerosol sampling station were highest when arriving air masses spent more time over southeastern Finland and lowland Sweden (supplementary fig. 5 and 7). However, supplementary fig. 7. illustrates two of the important limitations of single-site SQBTA: 1) limited discrimination between sources and transport pathways and 2) poor or no resolution of the back-edges of source regions[24].

Cesium-137 source regions showed some sensitivity to trajectory starting heights and duration (supplementary fig. 7). Higher starting heights tended to result in less discrimination between the transport paths and source regions within Sweden (supplementary fig. 7; supplementary fig. 5A). In contrast, the combination of lower starting heights and shorter durations suggested cesium-137 was more likely to originate from Finland, with limited contributions from mid-Sweden (supplementary fig. 7). Source estimates using the 300 m, 72 hr trajectories suggested a source region in eastern Finland – near Joensuu – that was not supported by other parameter combinations.

Some sensitivity to starting height and duration is expected. Low starting heights are sensitive to the topographic resolution of the meteorological grids[20] and trajectory estimates that intersect with the ground suffer information loss. Higher starting heights imply stronger winds and thus potentially more distant source estimates found by SQTBA[29]. Starting heights within the PBL are generally used in TERMs, as turbulent mixing is expected to homogenize particle concentrations. Although TERMs generally assume airborne particles are well-mixed within the PBL, it remains uncertain whether airborne eDNA behaves similarly—particularly for particles lacking protective exine shells, which may degrade more rapidly in the atmosphere. Encouragingly, a recent airborne eDNA study detected vertebrate eDNA throughout the PBL using light aircraft[30], but further research is needed to confirm this generality.

Results for all height and duration combinations are shown in supplementary fig. 8 and 9 for reindeer (summer and non-summer weeks, respectively), supplementary fig. 10 for cod, and supplementary fig. 11 for moose. We present SQTBA source estimates based on 48 hour back-trajectories at a 300 m in Fig. 2 in the main manuscript.

Given the cesium-137 results and the known limitations of SQBTA, these source regions should be interpreted qualitatively[19,31]. In single site SQBTA, the angular (*i.e.*, direction) resolution of potential source region is generally higher than the radial (*i.e.*, distance) resolution[24]. Local sources tend to be underestimated given the temporal resolution of the meteorology[24,31], while the detection of more distant sources is constrained by the trajectory duration. Despite these caveats, we chose moose, cod, and reindeer because non-local eDNA sources should exhibit a clear directional bias, whereas local sources should show no strong directional pattern.

Reindeer source estimates

Reindeer in Fennoscandia follow distinct seasonal migration patterns that vary by region. In Sweden, most reindeer migrate to alpine meadows in summer and return to boreal forests for the rest of the year. In Norway, they move between coastal summer pastures and interior tundra wintering grounds, while in Finland, reindeer herding is largely sedentary, with only small-scale seasonal movements (see Section 9). The inferred source regions for reindeer eDNA in summer (supplementary fig. 8) and non-summer weeks (supplementary fig. 9) correspond well to the average seasonal distribution of reindeer biomass across Fennoscandia and were robust to parameter choice. For reindeer, the largest discrepancies were in the summer source estimates, where trajectory duration influenced the strength of the source due north of the station (e.g., 24 vs. 48 h at 100 m) and trajectory height influenced the steepness of the gradient between weak and strong source regions, particularly with the 10 m models. This differences likely result from the complex terrain of the region, which may not be well-represented by the meteorological data. Interestingly, source estimates for cod showed a similar pattern (supplementary fig. 10), which further implicates terrain as an important variable.

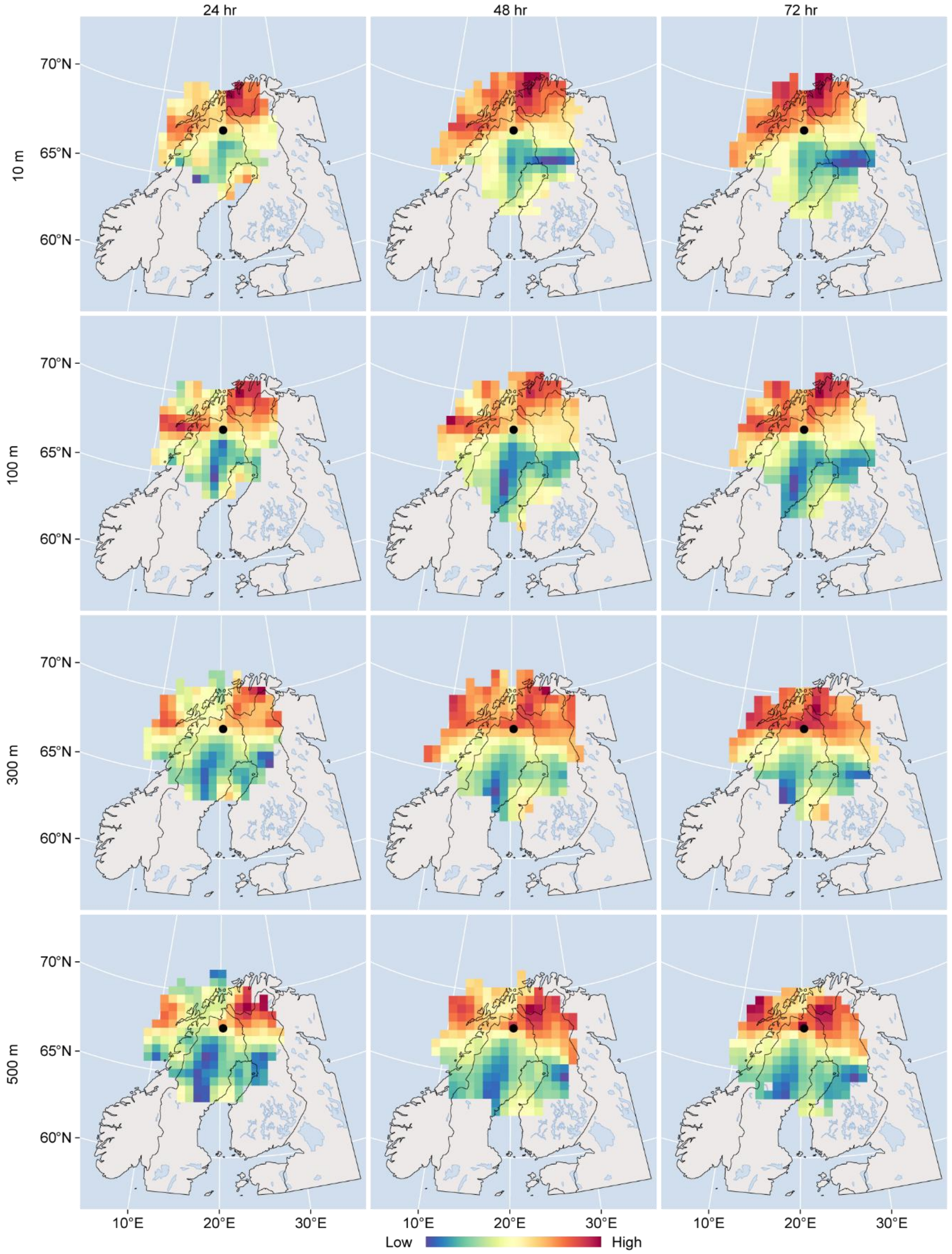

Supplementary Figure 8. Potential source areas of reindeer (*Rangifer tarandus*) eDNA captured by the aerosol sampling station during summer weeks.

Source areas were inferred using simplified quantitative transport bias analysis (SQTBA) applied to HYSPLIT back-trajectories initiated at 10, 100, 300, and 500 m above ground level (AGL), in rows, with durations of 24, 48, and 72 hours, in columns. The location of the aerosol sampling station is indicated by a black dot. Colours represent signal strength, with warmer tones (red–yellow) indicating stronger potential sources and cooler tones (blue–green) indicating weaker ones. Map from Natural Earth.

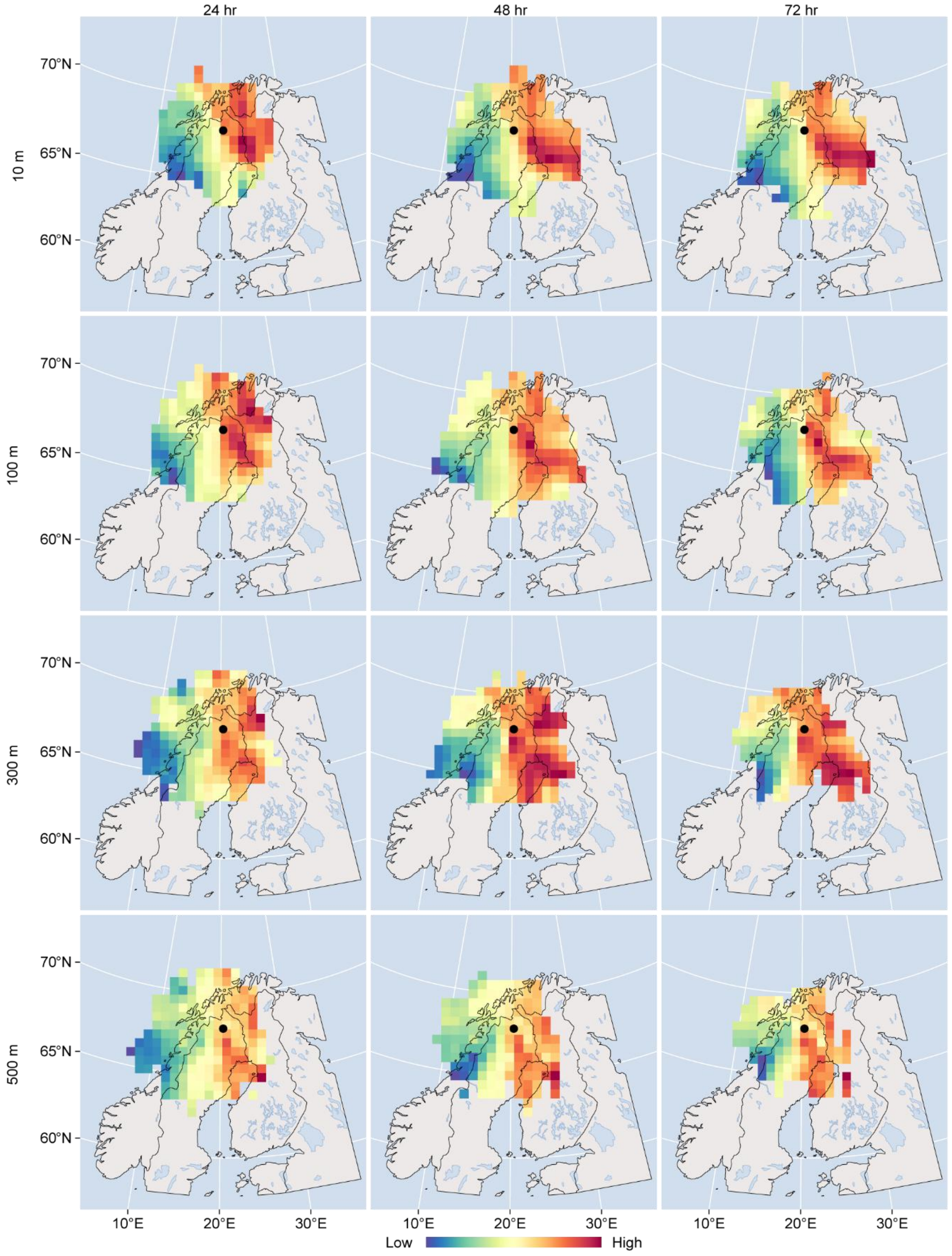

Supplementary Figure 9. Potential source areas of reindeer (*Rangifer tarandus*) eDNA captured by the aerosol sampling station during non-summer weeks.

Source areas were inferred using simplified quantitative transport bias analysis (SQTBA) applied to HYSPLIT back-trajectories initiated at 10, 100, 300, and 500 m above ground level (AGL), in rows, with durations of 24, 48, and 72 hours, in columns. The location of the aerosol sampling station is indicated by a black dot. Colours represent signal strength, with warmer tones (red–yellow) indicating stronger potential sources and cooler tones (blue–green) indicating weaker ones. Map from Natural Earth.

Cod source estimates

All height-duration parameter combinations found that cod eDNA relative abundances are higher when air masses originate from the north to northeast (supplementary fig. 10), consistent with a marine origin and the life-history of Atlantic cod. High latitude populations display either a sedentary or migratory ecotype, with the former spending their entire life cycle in the fjords, while the latter move between the Barents Sea and the fjords to spawn[32,33].

Lower starting heights (10 m, and to a lesser extent, 100 m) resolved the strong source to the north but the gradient between strong and weak sources was less pronounced. Reindeer in summer weeks showed a similar pattern (supplementary fig. 8). Poor topographic resolution of the complex terrain of the Norwegian coast, particularly the Lyngen Alps, located due north of the aerosol sampling station, may be the source of this pattern.

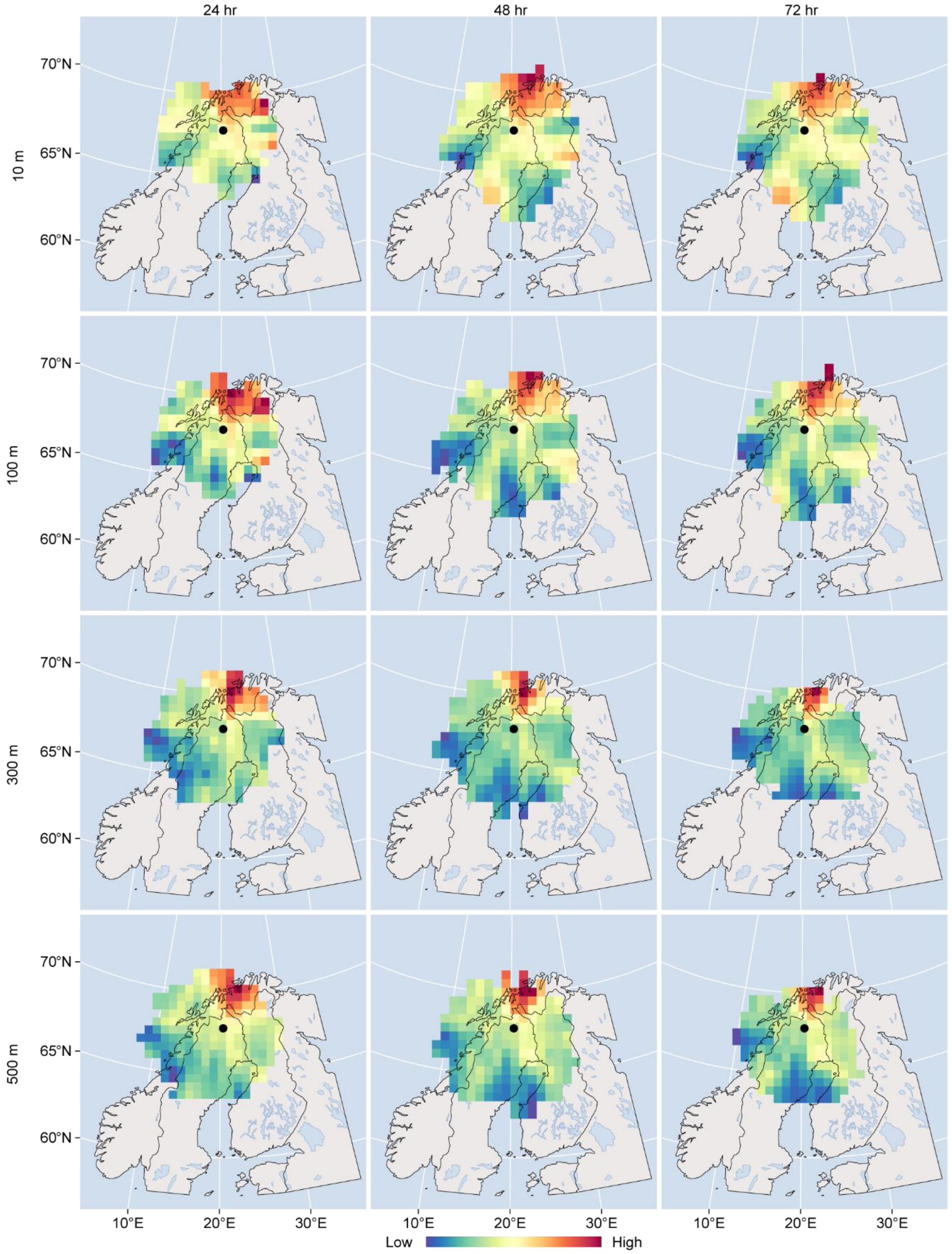

Supplementary Figure 10. Potential source areas of Atlantic cod (*Gadus morhua*) eDNA captured by the aerosol sampling station.

Source areas were inferred using simplified quantitative transport bias analysis (SQTBA) applied to HYSPLIT back-trajectories initiated at 10, 100, 300, and 500 m above ground level (AGL), in rows, with durations of 24, 48, and 72 hours, in columns. The location of the aerosol sampling station is indicated by a black dot. Colours represent signal strength, with warmer tones (red–yellow) indicating stronger potential sources and cooler tones (blue–green) indicating weaker ones. Map from Natural Earth.

Moose source estimates

Moose eDNA source estimates exhibited the greatest sensitivity to parameter settings (supplementary fig. 11). At 10 and 100 m, the strongest potential sources were located *ca.* 200 km east and southeast of the aerosol sampling station. Across all models, moose eDNA abundance was consistently lower when air masses arrived from the southeast. Trajectory estimate error could be higher over the Gulf of Bothnia, or unmodeled processes (*e.g.*, deposition) may be more important for moose eDNA than the other genera. Most parameter combinations identified a strong source region in Finnish Lapland, directly east of the aerosol sampling station. However, moose densities in Finnish Lapland are expected to be low[34] and comparable to those near the aerosol sampling station[35], although estimates of density and distribution patterns at a finer scale are lacking. Despite these uncertainties, source estimates indicate that moose eDNA relative abundance is highest when air masses originate from boreal forest regions and lowest when they originate from mountains to the west and the tundra to the north.

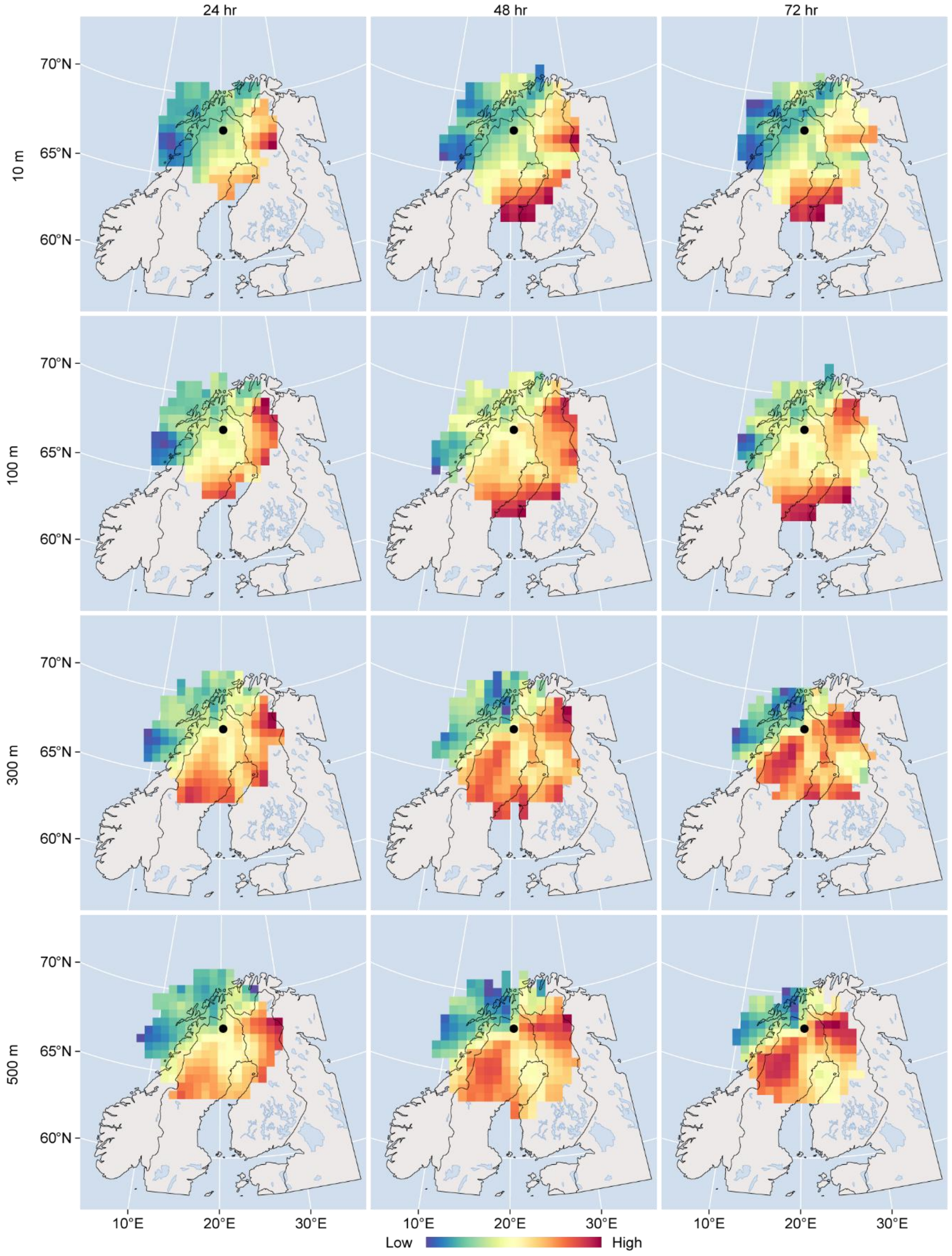

Supplementary Figure 11. Potential source areas of moose (*Alces alces*) eDNA captured by the aerosol sampling station.

Source areas were inferred using simplified quantitative transport bias analysis (SQTBA) applied to HYSPLIT back-trajectories initiated at 10, 100, 300, and 500 m above ground level (AGL), in rows, with durations of 24, 48, and 72 hours, in columns. The location of the aerosol sampling station is indicated by a black dot. Colours represent signal strength, with warmer tones (red–yellow) indicating stronger potential sources and cooler tones (blue–green) indicating weaker ones. Map from Natural Earth.

2. DNA sequencing

2.1. Extraction

The DNA extraction protocol was adopted from[3,36,37] with a few modifications. Prior to extraction, each airtight air filter container was wiped clean using 10% bleach and placed in sterile plastic bags containing a biopsy punch (Ø8 mm, Integra Miltex, Plainsboro, NJ, USA) and 2.0 mL screw cap tubes containing 1.0 g of 0.1 mm zirconia/silica beads and 0.5 g of 1.0 mm zirconia/silica beads (BioSpec, Bartlesville, OK, USA). For each air filter, three punches were punched out within the sealed sterile plastic bag using the biopsy punch and collected in three separate screw cap tubes. Air filter containers were resealed before opening the sealed bags to extract the screw cap tubes containing the filter punches. Prior to extraction, lysis and binding solutions were UV-radiated for a minimum of 90 min. A volume of 1.0 mL lysis buffer was then added to each tube (0.5 M EDTA, pH 8.0 (Thermo Fisher Scientific, Waltham, MA, USA), 0.5% Tween-20 (Sigma-Aldrich, Saint Louis, MO, USA) and 20 mg/mL Proteinase K (Thermo Fisher Scientific) and briefly agitated in a FastPrep-24 instrument (MP Biomedicals, Santa Ana, CA, USA) for 10 s at 4.0 m/s. The samples were then incubated at 37°C overnight. The next morning the samples were agitated for the same duration and speed and then centrifuged 15 min at 16,000 g. The supernatants (3 x 0.5 mL) belonging to the same air filter were pooled in a 50 mL screw cap tube (Sarstedt, Newton, NC, USA). An additional 0.5 mL buffer (0.5 M EDTA, 0.5% Tween-20) was added to each filter punch, agitated for 30 s at 5.0 m/s and centrifuged for 15 min at 16,000 g. The supernatants were collected and added to the corresponding 50 mL tube. This procedure was repeated once more with a 30 s, 6 m/s agitation, and a 5 min centrifugation step.

To each 50 mL tube, 8.8 volumes of binding buffer were added (5M GuHCl, (≥ 99%, Sigma-Aldrich), 40% Isopropanol (Thermo Fisher Scientific), 90 mM NaAc (pH 5.2, Sigma-Aldrich), 0.05% Tween-20 (Sigma-Aldrich), Nuclease free water (Qiagen, Hilden, Germany), followed by 10 s vortexing. Using a QIAvac 24 Plus vacuum manifold (Qiagen), the solution was then passed through a Zymo-Spin IIICG column (Zymo Research, Irvine, CA, USA) mounted with conical reservoirs (Zymo Research). The column was washed once with 0.75 mL binding buffer and twice with 0.75 mL 80% Ethanol (Thermo Fisher Scientific). The column was dried by centrifugation for 2 min at 13,000 g. The column was then moved to a DNA LowBind tube (Sarstedt) and 60 µL EB buffer (Qiagen) was added to the column. The column was then left for 5 min before the DNA was eluted by centrifugation for 1 min at 13,000 g. The eluted DNA was further cleaned using DNeasy PowerClean pro (Qiagen) and repaired using NEBNext FFPE DNA Repair Mix (New England Biolabs) as per manufacturers' protocol. The final DNA concentrations were measured using Qubit Fluorometric Quantification and the Qubit 1X dsDNA HS Assay Kit (Thermo Fisher Scientific). The amount of DNA extracted from each sample is available in supplementary data 2. In each sample batch (24 samples/batch) of the DNA extraction, blank samples were included. These samples were treated identical to the real samples, except that no air filter punches were added to the tubes, and none of them (n=21) showed any measurable amount of DNA at the end of the extraction protocol.

To estimate the amount of contaminating DNA potentially introduced to the filter DNA extractions, we further analyzed two control samples through DNA sequencing. The control samples consisted of a DNA extraction from one of the 21 reagent blank samples and a DNA extraction from a blank filter that had never been mounted in the aerosol sampling station. For both samples, the DNA concentrations were too low to measure using Qubit (HS Assay Kit). Since 10 ng of DNA is required for the DNA sequencing library preparation, we used the whole DNA extractions of both control samples and further included 10 ng DNA from *Drosophila melanogaster* as a spike-in. The DNA from both control samples and the *Drosophila* spike-in were barcoded separately before being pooled, where after a single sequencing library was prepared and sequenced on an Illumina MiSeq flow cell (Illumina MiSeq Reagent Kit v3). In total we obtained 1.311 x 10^7 reads. After de-multiplexing, 1.309 x 10^7 reads came from the spike-in, 1.97 x 10^4 reads from the reagent blank and 361 reads from the blank filter. Although not perfectly quantifiable, this shows that the reagent blanks contained approximately a thousand times less DNA (~10 pg) than the spike-in (10 ng), and the blank filter negligible amounts.

2.2. Sequencing

Libraries were prepared from isolates with a minimum of ~10 ng DNA (n=384 out of 505) at the Swedish National Genomics Infrastructure (SciLifeLab, SNP&SEQ, Uppsala) using the Thruplex DNA-Seq kit (Takara, Kusatsu, Shiga, Japan) with 7 PCR cycles according to the manufacturer's protocol. In the library production dual combinatorial index was used, and blank samples were included in each batch. None of those blank samples produced any measurable amount of library (using qPCR) and were not sequenced together with the air filter samples. Libraries (n=380 out of 384) were sequenced on Illumina NovaSeq 6000 S4 flow cells using 2 x 150 bp output (Illumina, San Diego, CA, USA). During de-multiplexing only one miss-match was allowed, and selection of barcodes was done so that two or more nucleotides varied between each sample barcode. Read numbers for sequenced weeks are shown in supplementary fig. 12. Sequencing data are available through the NCBI Sequence Read Archive under project PRJNA808200. The files are named according to the following format Ki-YYYY-WW-RandID, where Ki is short for Kiruna station, YYYY and WW are the ISO year and week, respectively, and RandID is the randomized ID that determined the order of DNA extraction and sequencing.

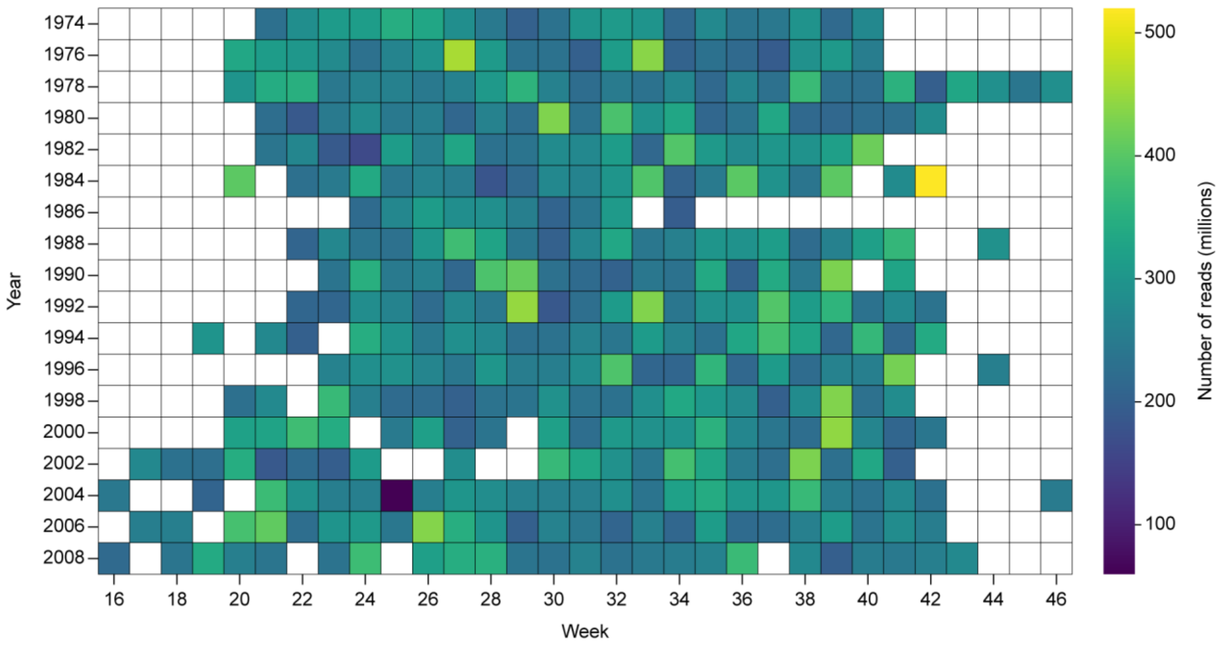

Supplementary Figure 12. Number of paired-end reads sequenced from each weekly air filter.

White cells indicate weeks without data. The consecutive run of missing data in the end of 1986 was due to air filters missing from the archive.

Supplementary Table 4: Output of PERMANOVA tests for differences in Extraction batch and Sequencing batch, based on Euclidian distances using centered log-ratio transformed data.

Two-sided permutational multivariate analysis of variance tests, evaluating possible batch effects associated with DNA extraction and sequencing. Reported are degrees of freedom (*df*), sums of squares (SumSq), mean squares (MeanSq), *F*-statistics (*F*.Model), coefficients of determination (R^2), and permutation-based *P*-values. No multiple-comparison correction was applied.

Term	*df*	SumSq	MeanSq	F.Model	R2	Pr(>F)
Extraction Batch	1	3734	3734.4	0.71181	0.00292	0.907
Residuals	243	1274867	5246.4	0.99708		
Total	244	1278601	1			
Sequencing Batch	1	5461	5460.9	1.0423	0.00427	0.342
Residuals	243	1273140	5239.3	0.99573		
Total	244	1278601	1			

To rule out possible batch effects originating from the DNA-extraction process and/or the sequencing, we calculated the Euclidean distances between samples using CLR-transformed data and evaluated the batch-effect by performing PERMANOVA tests as implemented in the “adonis” function in the R package ‘vegan’ v. 2.6-4[38]. No significant effects were found for the extraction (F = 0.71, p-value = 0.91) nor the sequencing batches, (F = 1.04, p-value = 0.34) as shown in supplementary table 4.

3. Bioinformatics pipeline

3.1. Read preprocessing and filtering

We first trimmed adapter sequences using Cutadapt *v.* 2.0[39] and retained reads with length ≥ 50 bp. Air filters are replaced at the aerosol sampling station by hand. Therefore, we removed reads mapping to the human reference genome hg19 using BBMap *v.* 38.69[40] with the following parameters: `minid: 0.95 maxindel: 3 minhits: 2 bandwidthratio: 0.16 bandwidth: 12 qtrim: "rl" trimq: 10 quickmatch: "quickmatch" fast: "fast" untrim: "untrim"`. The proportion of human reads detected and removed from the weekly sequence data are displayed in supplementary fig. 13.

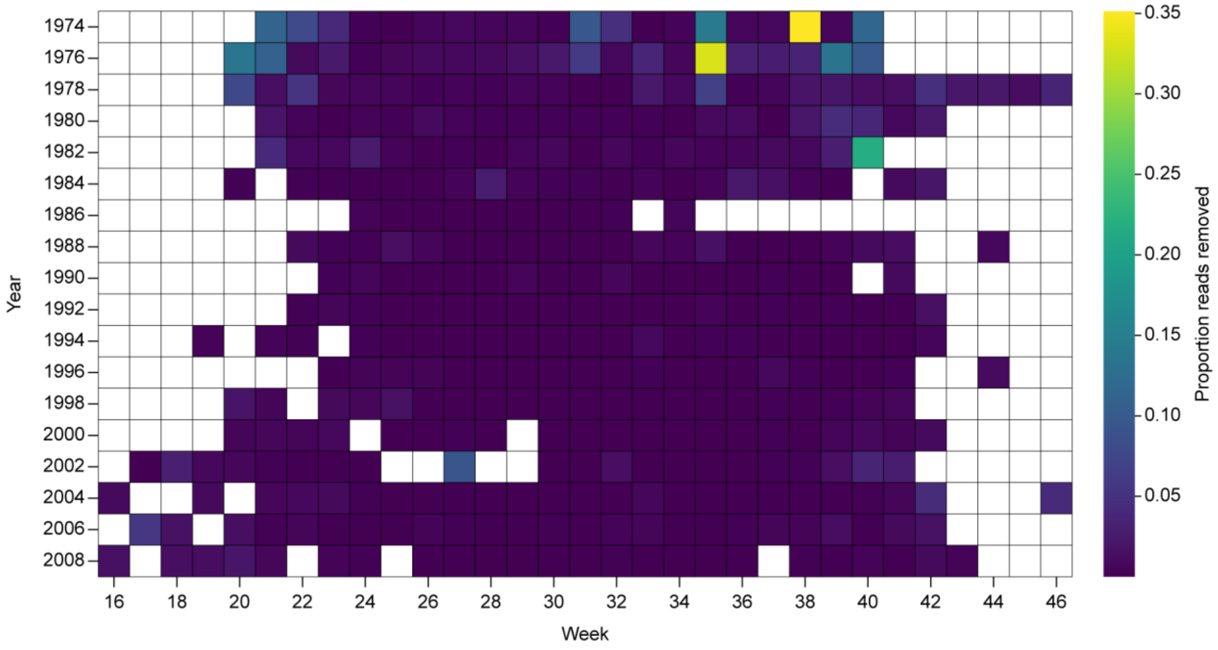

Supplementary Figure 13. Human read removal.

Proportion of paired-end reads from each week that mapped to the human reference genome and were removed prior to further analysis.

3.2. Taxonomic read classification

We used a version of Kraken 2 *v.* 2.0.8-beta[41] that we forked[c] to report the number of minimizer hit groups in the standard output and StringMeUp,[d] a post-processing python script developed in-house. StringMeUp allows reclassification of reads based on a user-specified confidence score stringency and/or minimum minimizer hit groups cutoff. It only requires the output from Kraken 2 and the taxonomy used to build the database. In short, StringMeUp processes each read by evaluating the confidence score at the currently assigned node. If the confidence score is less than the user-specified cutoff, the read is reclassified to the parent of the current node and the confidence score is recalculated as outlined in the manual of Kraken

[c] https://doi.org/10.5281/zenodo.17570001
[d] https://doi.org/10.5281/zenodo.17569636

2.[e] This continues until the confidence score requirement is satisfied. If the current node is the root and the confidence score is less than the cutoff, the read is deemed unclassified.

3.2.1. Custom Kraken 2 database

Input data for the Kraken2 database comprised nucleotide sequences from the 1) NCBI non-redundant nucleotide (nt), 2) NCBI RefSeq genomic, and 3) GenBank whole genome shotgun (WGS) databases. The nt fasta file contained 256 GB of sequence data and was downloaded[f] using the Kraken 2 command `--download-library`. The RefSeq genomic blast database was downloaded[g] from the NCBI ftp,[h] converted to a 1.6 terabyte (TB) fasta file using the NCBI blast+ package[42] application `blastdbcmd`, and staged for inclusion in the Kraken 2 database with the Kraken 2 command `--add-to-library`.

The WGS assemblies were selected in a multi-step process. First, a list of available WGS projects was acquired through the NCBI Sequence Set Browser[i] and WGS projects (at the species rank) non-redundant with the nt or RefSeq genomic databases were identified. Projects with unannotated (UNA) or environmental (ENV) sequences or that lacked a biosample or taxonomic ID were excluded, leaving 13,731 projects from 4,809 unique species and 2.4 TB of sequence data. Fasta files were downloaded using `fastq-dump`, part of the SRA toolkit,[j] and subsequently staged for inclusion in the Kraken 2 database in the same way as the RefSeq genomic fasta file.

Input for the Kraken 2 database build summed to 4.2 TB and included sequence data from 1,740,636 taxa from 89,168 named genera (supplementary data 3). From this, a 2.2 TB hash table (database) was built using 72 threads with a wall time of 75 hours. Minimizer and *k*-mer size settings were kept at their defaults.

3.2.2. Kraken 2 classification and filtering with StringMeUp

Sequences from the 380 weeks were classified using the Kraken 2 database (section 3.2.1) using 72 threads with a mean wall time of 1.96 hours per sample. Classifications were made under minimal stringency settings, *i.e.*, `--confidence 0` and `--minimum-hit-groups 1`. The reads were classified in this way so that StringMeUp could be applied on the output and stringency settings freely selected from a wide range. We found that 76,521 genera had at least one classified read under the minimum stringency threshold.

---

[e] https://github.com/DerrickWood/kraken2/wiki/Manual#confidence-scoring
[f] date: 2 January 2020
[g] date: 11 December 2019
[h] ftp://ftp.ncbi.nlm.nih.gov/blast/db/
[i] https://www.ncbi.nlm.nih.gov/Traces/wgs/
[j] https://trace.ncbi.nlm.nih.gov/Traces/sra/sra.cgi?view=software

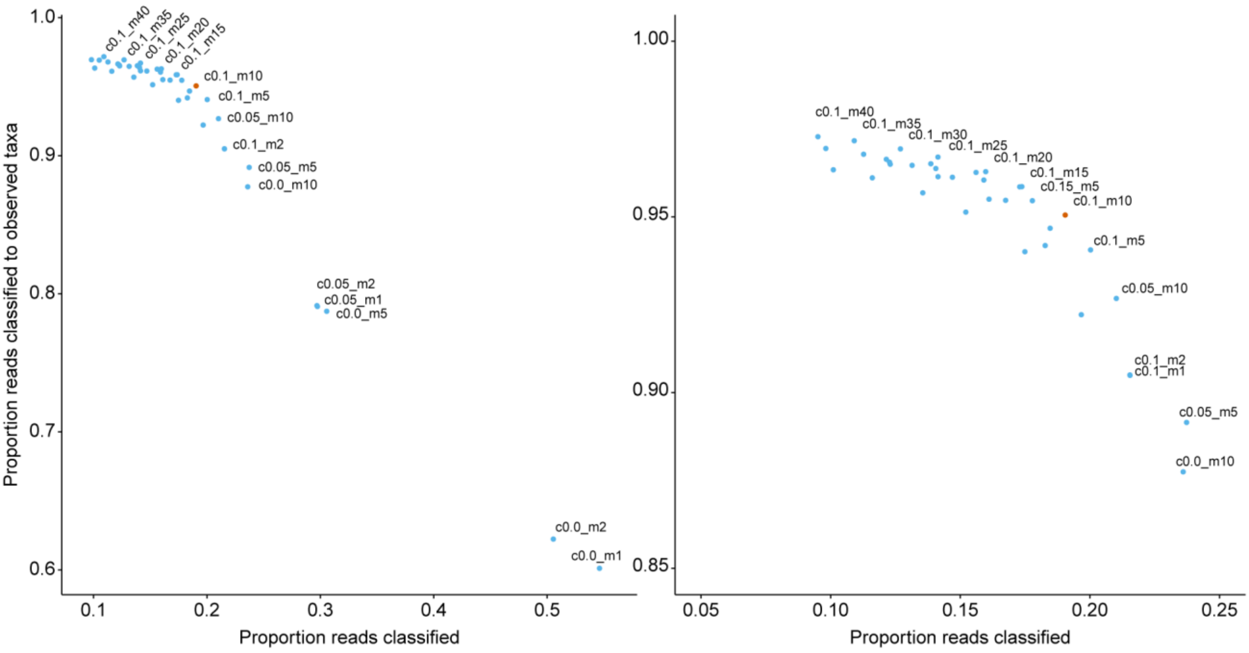

Supplementary Figure 14. A larger fraction of reads are classified to taxa reported in Torne lappmark whereas classification success decreases with increasing stringency cutoffs in Kraken 2.

Stringency was altered by varying the cutoffs for minimum confidence scores and hit groups (*e.g.*, c0.1_m2 denotes a confidence score of 0.1 and 2 minimum number of hit groups). The combination we used is marked in red (c0.1_m10). Parameter combinations that have a lower proportion of reads assigned to observed taxa at a comparable level of total assigned reads are unlabeled. The left panel shows all tested combinations and the right shows a detailed view of the more stringent parameter settings.

The penultimate step in the read classification pipeline was to select confidence score and hit group threshold. We randomly selected two weeks from each year (*n* = 36), subset the reads assigned to the most abundant genera (> 25th percentile), and then calculated the fraction of reads assigned to a taxonomic family observed in Torne lappmark[k] out of all assigned reads over a grid of cutoff combinations. Taxa observations were retrieved from the Swedish Species Observation System database[l;43] (supplementary data 4). Confidence scores were evaluated at 0, 0.05, 0.1, 0.15, 0.2, 0.25, and 0.3. Minimum hit groups were evaluated at 1, 2, 5, and 10 for all confidence scores and at 15, 20, 25, 30, 35, and 40 for confidence scores 0, 0.05, and 0.1. The parameter space was extended until no improvement (in proportion of reads assigned to Torne lappmark taxa) was observed.

We considered a minimum confidence of 0.1 with a minimum of 10 hit groups to be a good trade-off between the fraction of reads assigned to taxa plausibly present near the aerosol sampling station and the total number of classified reads (supplementary fig. 14). Using this level of stringency, 40,034 genera had at least one classified read. More stringent cutoffs marginally increased the Torne lappmark fraction but the total number of classified

[k] a historic administrative division, roughly extending 100 km north, east, and west and 15 km south of the aerosol sampling station

[l] Artportalen, a repository for biological surveys in Sweden and quality-reviewed community observations: https://artportalen.se/

reads continued to decrease almost linearly. A less stringent cutoff combined with the machine classifier in Section 4.3 may have increased the sensitivity of our assignments, but we preferred this more conservative approach for the ecosystem-level biodiversity analyses. Finally, we removed taxa that did not have > 10 classified reads in any of the weekly samples, leaving 15,672 genera.

4. Relative abundance transformations and detrending

4.1. Removal of zero inflated taxa and log-ratio transformations

Metagenomic datasets are a type of compositional data because the maximum number of reads is constrained by the sequencing instrument. In our dataset, classified reads for a given week comprise a *D*-part composition, where *D* is the number of genera. The sample space of a *D*-part composition is a subset of $\mathbb{R}^D$ known as the simplex, $\mathbb{S}^{D-1}$[44]. Because a composition is only free to vary in $\mathbb{S}^{D-1}$, operations defined on $\mathbb{R}^D$ are invalid. More simply, compositional vectors cannot be directly added, subtracted, or multiplied, and methods based on the covariance matrix cannot be expected to give sensible results.

Log-ratio transformations are increasingly common[45] because they allow relative abundances to be analyzed using standard statistical methods, such as PCA, regression, and cluster analysis. Log-ratio transformation use the Aitchison geometry to define a Euclidean vector space on the simplex and express compositions in $\mathbb{R}$ with respect this geometry[46]. Importantly, read counts are inherently compositional and log-ratio transformations simply facilitate robust statistical analysis in $\mathbb{R}^D$.

We performed most subsequent analyses on log-ratio transformed data, which requires addressing zero count data first. An observation of zero reads from an organism may be due to its true absence from the catchment area, but we assume zeros from regularly detected taxa are artifacts of limited, stochastic sampling. We removed 9,380 genera with zero counts in ≥ 2/3 of the weeks and imputed zeros for the remaining 6,292 using geometric Bayesian multiplicative replacement[47] as implemented by the `cmultRepl` function in the *R* package 'zCompositions' *v.* 1.4.0-1[48]. This method replaces zeros with estimates drawn from a multinomial distribution and preserves the sum and correlation structure of the composition.

The centered log-ratio (CLR) transformation maps a composition from the simplex $\mathbb{S}^{D-1}$ to the unconstrained space of $\mathbb{R}^D$. The CLR transformation is an isometry, meaning the Euclidean distances between two parts of a composition in $\mathbb{R}^D$ is equivalent to the Aitchison distance in $\mathbb{S}^{D-1}$. Because each week has the same set of *D* taxa, the CLR transformation basis remains consistent across time, ensuring that comparisons remain valid. The CLR provides a one-to-one transformation of all features, which makes interpretation easier, but the resulting covariance matrix is always singular.

For a weekly composition $x^{(w)} \in \mathbb{S}^{D-1}$,

$$clr\left(x^{(w)}\right) = \left(ln\left(\frac{x_{w,1}}{g\left(x^{(w)}\right)}\right), ln\left(\frac{x_{w,2}}{g\left(x^{(w)}\right)}\right), \ldots, ln\left(\frac{x_{w,D}}{g\left(x^{(w)}\right)}\right)\right) \quad (1)$$

where $g\left(x^{(w)}\right)$ is the geometric mean of the D parts in the weekly composition $x^{(w)}$.

An alternative is the isometric log-ratio (ILR) transformation, which assigns coordinates in $\mathbb{R}^{D-1}$ with respect to an orthonormal basis in $\mathbb{S}^{D-1}$. This transformation can be done according to the formulae:

$$ilr(x^{(w)}) = z^{(w)} = (z_{w,1}, \dots, z_{w,D-1}) \quad (2)$$

$$z_{w,j} = \sqrt{\frac{D-\mathrm{j}}{D-\mathrm{j}+1}} ln\left(\frac{x_{w,j}}{\sqrt[D-\mathrm{j}]{\prod_{k=\mathrm{j}+1}^{D} x_{w,k}}}\right), \qquad \mathrm{j} = 1, \dots, D-1 \quad (3)$$

where D is the number of parts in the weekly composition $x^{(w)}$.

Like the CLR transformation, the basis system in the ILR transformation is constant across weeks. The ILR transformation is also an isometry and matches the dimension of the simplex in $\mathbb{R}$ and therefore does not result in singular covariance matrices. The tradeoff is the ILR transformation losses interpretability because matching the dimensionality of $\mathbb{S}$ means there cannot be a one-to-one correspondence of the D compositional parts. While the ILR transformation is statistically robust[46,49,50], the loss of interpretation can be a limitation. We used the ILR transformation and its inverse when intermediate results were not of primary interest, such as in the detrending procedures described in Section 4.2. This allows confounding effects to be accounted for without distorting the relative relationships among taxa.[50]

We used a special case of the ILR transformation for the univariate analyses of the clusters and individual genera. This is called the pivot log-ratio (PLR) transformation[46] and simply involves designating the taxon (or cluster) of interest as the first balance in the transformation. After the PLR transformation, all information about the selected taxon x_1 is contained the first coordinate z_1. The same cannot be said about the z_2 to z_{D-1} coordinates since *e.g.*, x_2 is used in the calculation of z_1 and z_2. However, the PLR transformation can be applied to each $x = (x_1, \dots, x_D)$ taxa. This results in PLR-transformed relative abundances on D different basis systems, measuring the relative dominance of each of the D taxa in the composition[46]. Despite the difference in basis between PLR-transformed taxa, constructing D separate regression models using the PLR transformation is equivalent to performing multivariate regression in the native simplex sample space[46,51]. This makes the PLR-transformation a pragmatic solution when univariate analysis or visualization of compositional parts is desired or necessary, although it is important to note that this does not remove the dependence between compositional parts. Ensuring this distinction is maintained is essential for the proper presentation and interpretation of results. For the cluster time series models (Sections 5 and 8), we back-transformed the trends into standard relative abundances. Univariate representations of PLR-transformed genera (Sections 1.3.2 and Section 8.3) were supported by external, non-eDNA data. PLR transformations were made with the *R* package 'robCompositions' *v*. 2.3.1[46,52].

4.2. Detrending

We identified three confounding factors that could bias eDNA abundance estimates: 1) a change in air filter manufacturer in 1996, 2) potentially more human contamination earlier in the time series (supplementary fig. 15), and 3) read length variation due to partial DNA degradation (supplementary fig. 15).

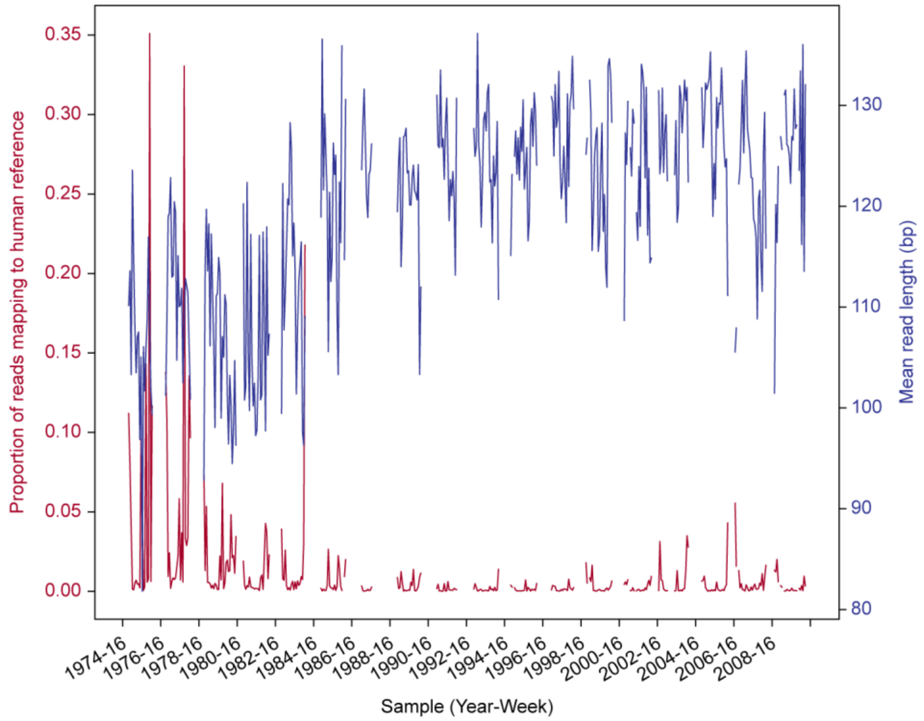

Supplementary Figure 15. Proportion of human reads and mean read length.
The proportion of reads that mapped to the human reference genome (red) and the mean read length in base pairs (blue).

We addressed read length variation by removing trends between genera abundances and their weekly mean read length. First, we applied the ILR transformation to both read lengths and relative abundances prior to detrending using the *R* package 'compositions' *v*. 2.0-6[53]. We modeled the weekly abundance of a given ILR component as a function of mean read length using generalized linear models (GLM). GLMs for each component were fit using the python module 'statsmodels' *v*. 0.11.1[54] with the log, identity, and inverse link functions. The best fit was inferred using the Akaike information criterion[55]. Weeks with a zero read count for a given component were not included in the models, leaving their imputed zero values unchanged. Sample means were re-added to the residuals, which were inversely transformed to relative abundances using the 'compositions' package. Redundancy analysis (RDA) was applied to the relative abundance matrix conditioned on air filter type and human read count proportion using the *R* package 'vegan' *v*. 2.6-4[38] and the residuals were then used for subsequent analysis. For a comparison of the data before and after detrending, see supplementary fig. 16.

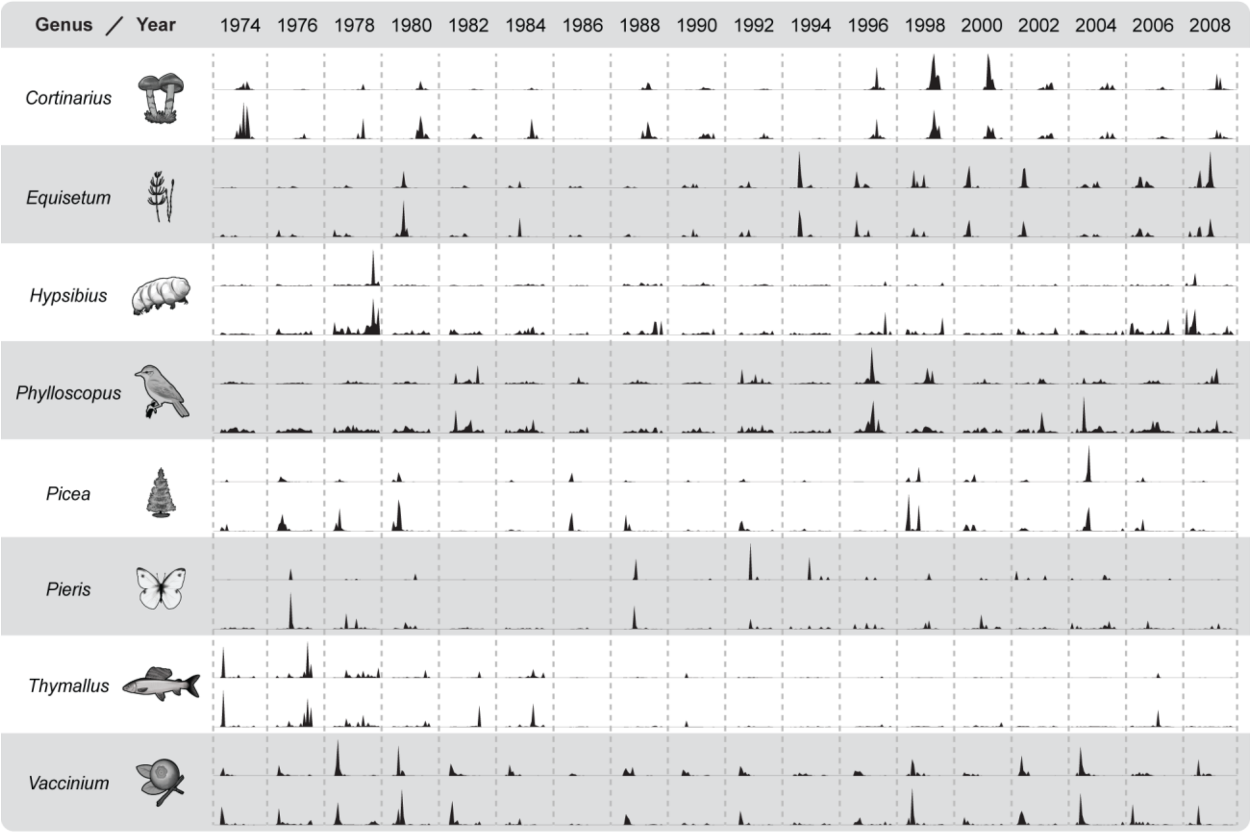

Supplementary Figure 16. Comparison of relative abundances before and after detrending.

For each genus two data tracks are shown. The top track shows relative abundances before detrending and the bottom after detrending. Abundances are scaled between 0 and 1. Organism illustrations by Thomas Ågren.

4.3. Classification refinement with gradient boosting

The 6,292 genera putatively captured by the air filters included unlikely taxa such as the white rhinoceros (*Ceratotherium simum*). Besides initial misclassification due to read quality, redundant *k*-mers, and low sequence abundance, false positives may also arise from contaminants and other issues in the reference genomes and the unique computational burden imposed by any given database[56]. To address this problem, we developed a gradient boosting machine (GBM) to distinguish between taxa likely to be true and false positives based on their classification metrics and abundance patterns throughout the time series.

4.3.1. Feature engineering

Based on the known limitations of the classification pipeline[56] and the behavior of a few conspicuous false positives (see also Section 4.4), we hypothesized that false positive genera would have lower abundances; be detected rarely, or alternatively, with unusual consistency; occur more frequently in lineages with more sequence data and/or larger genomes, and have distinct per-read Kraken 2 classification quality metric profiles. We defined 31 features (parentheses correspond to column names in supplementary data 5) from these expectations and calculated them for each genus:

1) mean abundance (abundance_mean) and 2) its square (abundance_mean_squared),
3) median abundance (abundance_median) and 4) its square (abundance_median_squared),
5) 5th percentile of weekly abundances (abundance_percentile_5th) and 6) its square (abundance_percentile_5th_squared),
7) 95th percentile of weekly abundances (abundance_percentile_95th) and 8) its square (abundance_percentile_95th_squared),
9) number of weeks with relative abundance > 0 (weeks_present) and 10) its square (weeks_present_squared),
11) standard deviation of relative abundance (abundance_sd) and 12) its square (abundance_sd_squared),
13) abundance coefficient of variation (CV) and 14) its square (CV_squared),
15) number of minimizers per clade (minimizers_clade),
16) number of minimizers per taxon (minimizers_taxon),
17) total sequences per clade (total_sequence_clade),
18) total sequences per taxon (total_sequence_taxon),
19) ratio of mean abundance to number of clade minimizers (abundance_mean_minimizerC_ratio),
20) ratio of median abundance to number of clade minimizers (abundance_median_minimizerC_ratio),
21) ratio of mean abundance to number of taxon minimizers (abundance_mean_minimizerT_ratio),
22) ratio of median abundance to number of taxon minimizers (abundance_median_minimizerT_ratio),
23) Kraken 2 confidence score: total number of *k*-mers classified to a given genus divided by the total number of *k*-mers from the corresponding reads (confidence_original),
24) alternative confidence score: same as feature 22 but without unclassified *k*-mers in the denominator (confidence_classified),
25) other *k*-mers lineage ratio: number of *k*-mers classified to any node leading to the assigned genus divided by the total number of *k*-mers, excluding those classified to the genus (other_kmers_lineage_ratio),
26) other *k*-mers root ratio; the ratio of *k*-mers classified to the root node to the total number of *k*-mers, excluding those classified to the genus (other_kmers_root_ratio),
27) other *k*-mers classified ratio: the ratio of classified to unclassified *k*-mers, excluding those classified to the genus (other_kmers_classified_ratio),
28) other *k*-mers distance: the average taxonomic distance (number of intervening edges) between the nodes that the *k*-mers are classified to (other than the genus) and the genus that the corresponding reads are classified to (other_kmers_distance),

29) other *k*-mers distance lineage excluded: as in feature 27, but excluding *k*-mers classified to any rank in the lineage leading to the genus (other_kmers_distance_lineage_excluded),

30) total *k*-mers: sum of *k*-mers classified to the genus clade across the time series (total_kmers), and

31) number minimizer hit groups per *k*-mer: the sum of minimizer hit groups from reads classified to a genus divided by the total number of *k*-mers (mhg_per_kmer).

We also considered the possibility that one or more weeks could be enriched for false positives by including the weekly abundance of each genus as features. Finally, we one-hot encoded kingdom-rank assignments to allow these features to differ in their utility and probability distributions. All features were calculated from PLR transformed data (Section 4.1).

4.3.2. Training data acquisition

As we lacked empirically known training data, we used species occurrence records to create two groups that we expect to be enriched for true and false positive taxa, respectively. As positive training data, we used genera registered in the Swedish Species Observation System[m] with > 3 observations reported from ≤ 40 km of the aerosol sampling station between 1974-2008[57]. We also included humans, dogs, *Aedes*, and 33 bacterial genera identified in soil and water samples from a similar ecosystem,[n] yielding 317 in total. For negative taxa, we identified 379 taxa that 1) have no reported occurrences in the Global Biodiversity Information Facility online database (GBIF) within 5,000 km of the aerosol sampling station[58], and 2) are not closely related to any European taxa lacking a reference genome. For example, Glossinidae, containing the *Glossina* tsetse flies, is in the same superfamily as the Hippoboscidae, which occur in Europe and lack a representative genome, so *Glossina* was not considered a negative genus. These criteria presumably exclude many actual false positives (*i.e.*, where the classification does not result from shared ancestry) from the training data, but we wanted to allow genera poorly represented in the reference database to be captured at higher taxonomic ranks. Prior to model training, we randomly selected and set aside 13% (n = 91) of the presences and absences as test data. The full list of labeled taxa and their feature data are provided as supplementary data 5 and their taxonomic composition is summarized in supplementary table 5.

---

[m] Artportalen, a repository for biological surveys in Sweden and quality-reviewed community observations: https://artportalen.se/

[n] NCBI Bioproject accession number PRJNA767205

Supplementary Table 5. Taxonomic composition of training data.
Taxa are divided by kingdom into positive and negative and test and training fractions. Orders with more than 15 labeled taxa are shown; the remaining taxa in each kingdom are summed as 'others'.

taxon	training		test		total	
	neg.	pos.	neg.	pos.	neg.	pos.
Bacteria	*0*	*29*	*0*	*4*	*0*	*33*
Metazoa	*212*	*70*	*35*	*12*	*247*	*82*
Mammalia	110	11	18	0	128	11
Aves	27	31	8	4	35	35
Insecta	4	25	0	6	4	31
Actinopteri	40	0	6	1	46	1
others	31	3	3	1	34	4
Viridiplantae	*90*	*99*	*15*	*16*	*105*	*115*
Magnoliopsida	33	60	5	10	38	70
Pinopsida	26	3	4	0	30	3
Polypodiopsida	24	7	6	0	30	7
Bryopsida	0	16	0	3	0	19
others	7	13	0	3	7	16
Fungi	*24*	*81*	*3*	*6*	*27*	*87*
Agaricomycetes	16	52	1	2	17	54
Lecanoromycetes	0	14	0	3	0	17
others	8	15	2	1	10	16
**total**	**326**	**279**	**53**	**38**	**379**	**317**

4.3.3. Parameter tuning and classification

We trained the GBM using the *R* interface for xgboost *v.* 1.7.5.1[59]. We iteratively performed grid searches with 5-fold cross validation over a total of 6,561 hyperparameter combinations to identify a set approaching the smallest binary classification error rate. First, we fixed the learning rate (eta) to 0.3 and explored regularization and tree-specific parameters over the grid:

```
max_depth = c(1, 3, 5, 7, 9),
min_child_weight = c(1, 3, 5, 7, 9),
gamma = c(0.0, 0.01, 0.1, 0.3, 0.5, 1.0),
subsample = c(0.4, 0.6, 0.8),
colsample_bytree = c(0.4, 0.6, 0.8),
reg_alpha = c(1e-5, 1e-2, 0.1, 1, 100),
reg_lambda = c(1.0, 1.5, 2.0, 3.0, 4.5).
```

We defined successively narrower ranges over six tuning rounds and, in the final round of tuning, tested `eta = c(0.1, 0.15, 0.2, 0.25, 0.3)` with the remaining parameters fixed. The final trained model used: `eta = 0.3, max_depth = 5, min_child_weight = 2,`

`subsample = 0.7, colsample_bytree = 0.4, reg_alpha = 1e-05, gamma = 0.3, reg_lamba = 1.5.`

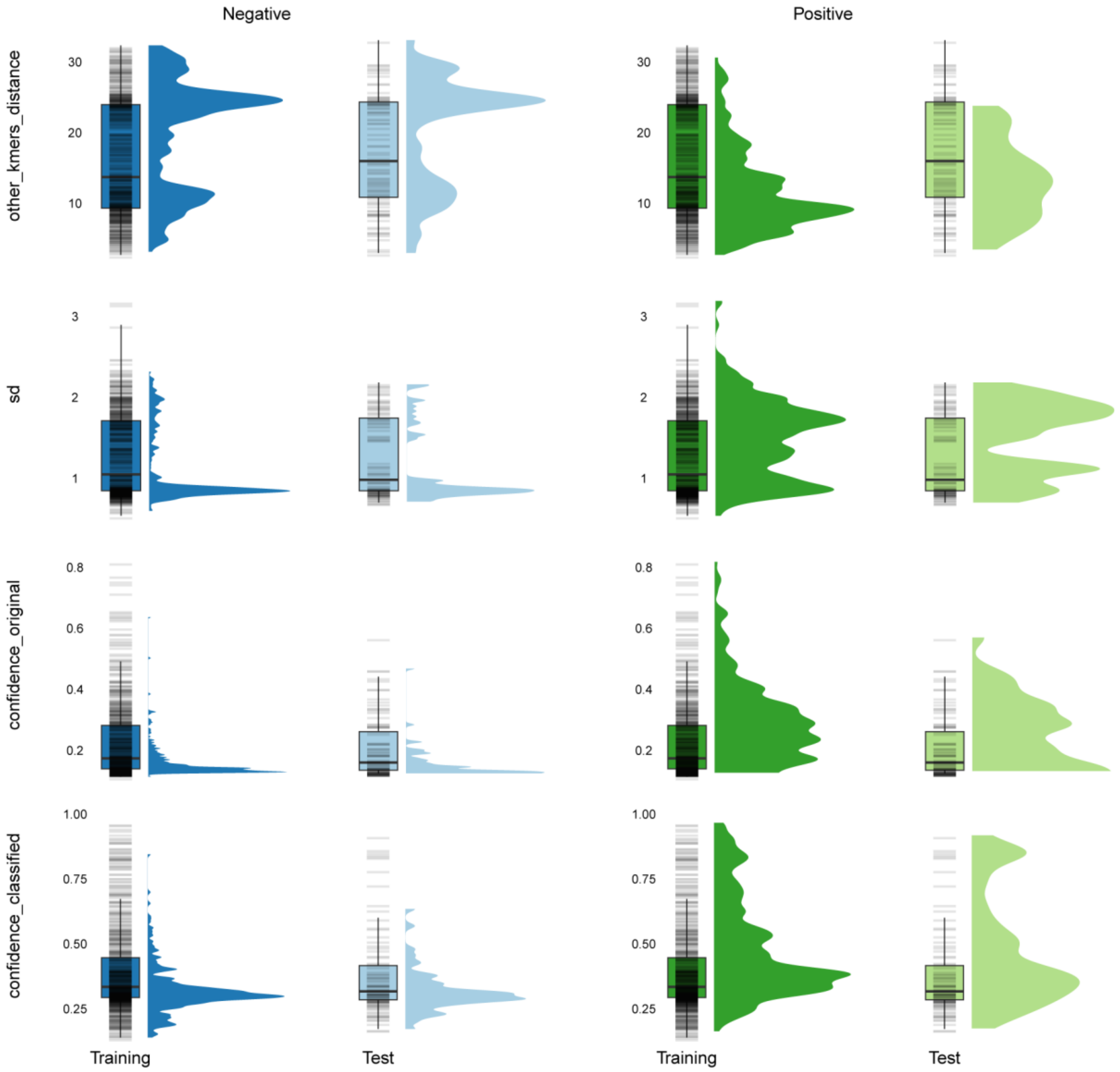

Supplementary Figure 17. Distributions of the four most influential features in the gradient boosting machine classification model.

Each row corresponds to a feature. The first two columns (blues) show values for the labeled negative genera and the last two columns (greens) show labeled positive genera. Training data are shown in a darker shade and test data in a lighter shade.

Four features comprised 45% of the binary classification error improvement: the original Kraken 2 confidence score (25%; feature 23 in Section 4.3.1); other *k*-mers distance (13%; feature 28), relative abundance standard deviation (4%; feature 11), and the classified confidence score (3%; feature 24). Negative genera tended to have a larger other *k*-mers distance, a smaller standard deviation, and lower confidence scores than positives (supplementary fig. 17). This suggests false positive genera are likely to show limited variation in abundance over the time series and that reads with *k*-mers assigned to false positives tend to also contain *k*-mers assigned to taxonomically-distant clades. In particular,

k-mer distances > 20 result if a read contains *k*-mers classified to both eukaryotes and prokaryotes, which can occur from reference genome contamination (Section 4.4).

We compared the F1 score, false discovery rate (FDR), precision, and recall for the test data over a range of predicted classification probabilities (supplementary table 6). For the final classification of the 6,292 genera dataset, we selected the 0.75 probability threshold, which classified 2,739 as true occurrences (weekly relative proportions of these taxa are given in supplementary data 6).

Supplementary Table 6. Gradient boosting machine (GBM) classification performance. F1 score, false positive rate (FPR), precision, false discovery rate (FDR), and recall are reported for the n = 91 test dataset using predictive probabilities from 0.50 to 0.95 as the cutoff for a positive classification. '#negative' and '#positive' denote the number of genera below or above a given cutoff, respectively, out of the 6,292 genera dataset.

cutoff	F1	FPR	precision	FDR	recall	#negative	#positive
0.50	0.79	0.09	0.85	0.15	0.74	2,830	3,462
0.55	0.80	0.08	0.88	0.13	0.74	2,941	3,351
0.60	0.80	0.08	0.88	0.13	0.74	3,083	3,209
0.65	0.80	0.08	0.88	0.13	0.74	3,225	3,067
0.70	0.81	0.06	0.90	0.10	0.74	3,369	2,923
0.75	0.81	0.04	0.93	0.07	0.71	3,553	2,739
0.80	0.81	0.04	0.93	0.07	0.71	3,737	2,555
0.85	0.73	0.04	0.92	0.08	0.61	3,960	2,332
0.90	0.66	0.02	0.95	0.05	0.50	4,267	2,025
0.95	0.64	0.00	1.00	0.00	0.47	4,795	1,497

4.4. Alignment-based validation of classified taxa

We developed a classification validation framework from two predictions for a correctly-classified genus: 1) reads should originate from the entire genome, resulting in a positive correlation between contig length and read depth, and 2) sequences should be similar to closely-related organisms, evident in the taxonomic distribution of BLAST best-scoring sequence pairs (BSPs). In summary, we mapped Kraken 2 classified reads to their respective sequences in the reference database (supplementary fig. 18). Then, we generated consensus sequences for the 100 regions with the highest read depth and used BLASTN to compare these to the nt database. If BLASTN found BSPs with unrelated organisms, we generated consensus sequences for the next 100 regions by depth and repeated this process until no new potential contaminants were found.

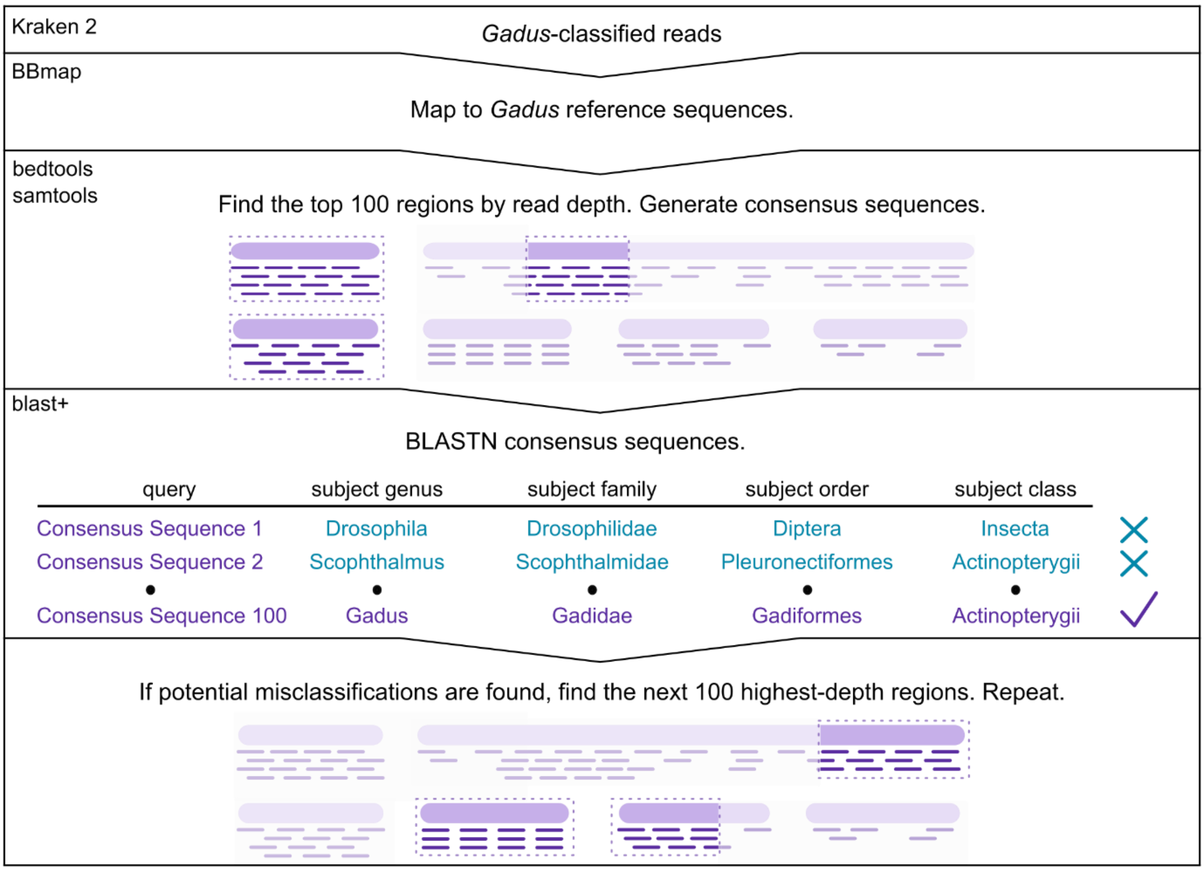

Supplementary Figure 18. Summary of methods used to validate genus classifications. Validation of gradient-boosting classifications using read alignment to reference assemblies and sequence similarity searches with BLAST. For more details see supplementary data 7.

We also queried classified reads directly against the nt database for a subset (n = 26) of genera. These results are also described in supplementary data 7, but consensus sequences enabled more powerful detection of misclassified reads and could frequently determine their true identity.

4.4.1. Genera selection

Alignment-based methods, particularly the BLAST component of the validation framework, are prohibitively computationally intensive and slow. Therefore, we prioritized the genera most important for the validity and interpretation of this manuscript. Genera were flagged for validation if:

- their detection in air is noteworthy because they lack an aerial or arboreal life stage, *e.g.* fish (*Gadus*, *Salmo*, *Thymallus*), mammals (*Rangifer*, *Alces*), or amphibians (*Rana*)
- they were analyzed individually, *i.e.*, *Gadus*, *Alces* (Section 1.3.2), and nine bird genera (Section 8.3)
- had large γ-diversity changes over the time series; these are annotated in Fig. 5D in the main text, e.g. *Lachnellula*, *Melamspora*, and *Mielichhoferia*
- or were abundant (top 50), macroscopic organisms without > 3 GBIF occurrence records within 40 km of the aerosol station between 1974-2008.

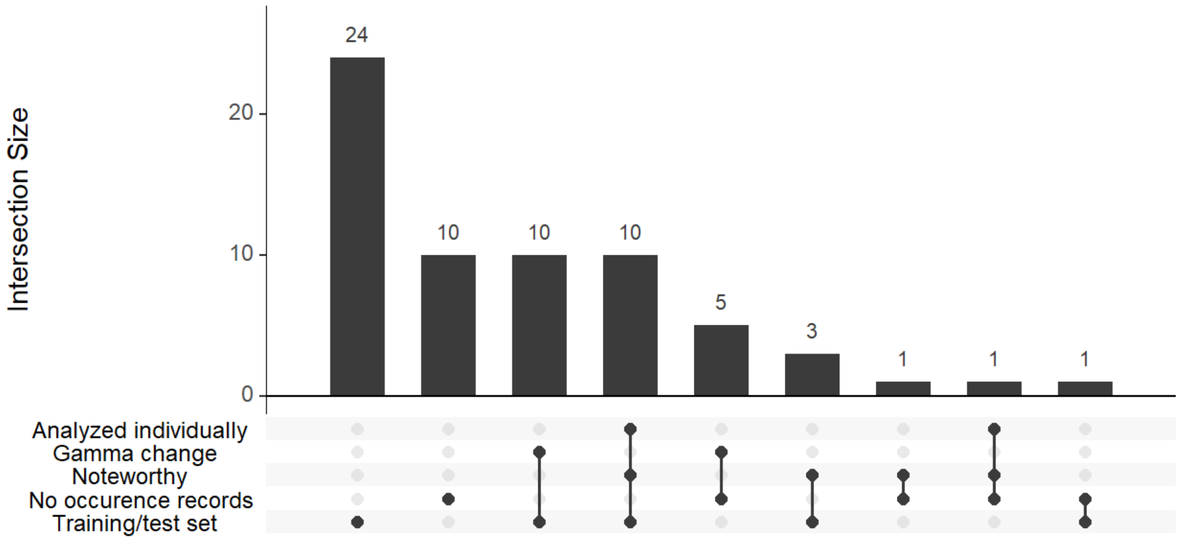

Supplementary Figure 19. Selection criteria met by the 66 genera subjected to read-level validation.

Many genera fulfilled multiple criteria and/or were included in the GBM training data (supplementary fig. 19). To estimate label noise in the training dataset (*e.g.*, a positive genus that is truly absent), we selected 14 more positive training genera (with positive binary classifications) to match the taxonomic composition of the full training/test set as closely as possible. Finally, we included 9 negative training genera (flowering plants, vertebrates, and an insect) for comparison.

4.4.2. Read mapping and consensus calling

We mapped reads classified to a genus back to their respective reference sequences in the Kraken 2 database (masked for low-complexity sequences) using using BBMap *v.* 38.98[40] with the following parameters: `pairedonly = t ambiguous = best killbadpairs = f minid = 0.97` (other parameters set as default). We extracted classified reads from all weeks for most genera but used reads from the week with the highest abundance in each year for *Larix* (9.5 x 10^8 reads) and the three weeks of highest abundance for *Betula*, *Picea*, and *Pinus* (2.4–3.5 x 10^8 reads).

We used bedtools *v.* 2.18[60] to find reference features with continuous read coverage with the command: `bedtools merge -i maped_reads.bam -d 1 -c 1 -o count`. Then, we used the `consensus` utility from samtools *v.* 1.20[61] with default parameters to generate consensus sequences from high read-depth regions. For all genera, we called consensus sequences for the 100 regions with the highest read depth and expanded this up to 1,000 regions if BLASTN alignments (described below) indicated potential read misclassifications. Long consensus sequences were divided into non-overlapping 3,000 bp subsets

4.4.3. BLAST searches

We used BLAST *v.* 2.10.1+[42] to compare consensus sequences from all genera against the nt database using the following parameters (unspecified parameters kept as default):

```
-task blastn
```

```
-db nt
-outfmt "6 std staxids sscinames sskingdoms"
-num_threads 4
-max_hsps 5
-evalue 1e-10
```

For 26 genera, we queried 100 randomly-selected paired-end reads using the same settings.

We filtered the BLAST results to remove self-matches (*i.e.*, with the same accession number or from same assembly), alignments ≤ 150 bp, ≤ 80% identity, or with an e-value > 1 x 10^{-25}, and then retained the five best-scoring sequence pairs (BSPs) by bitscore per query. Results for the read queries were filtered similarly, except we removed alignments ≤ 100 bp or with an e-value > 1 x 10^{-15} and could not remove self-matches. We summarized the distribution and mean alignment length, identity, e-value, and bitscore of BSPs by taxonomic rank. For the consensus sequences, we weighted BSPs by their read depth. That is, BSPs for a consensus called from 100 mapped reads would count towards the taxonomic distribution of alignments 100 times. This allowed estimation of the read-level misclassification rate even when a large number of reads mapped to a small number of reference contaminants, which was the typical pattern for the genera analyzed here (see supplementary data 7).

5. Dimensionality reduction and clustering

5.1. Taxa-based clustering and ordination

Standard measures of correlation and distance are inappropriate for compositional data due to their constrained covariance structure. Therefore, we employed an analogue of dissimilarity calculated from the pairwise variance between CLR transformed abundances[62,63]:

$$\varphi_s(D_i, D_j) = \frac{var(D_i - D_j)}{var(D_i + D_j)} \tag{4}$$

We then performed hierarchical clustering of the 2,739 genera based on their pairwise φ_s using Ward's method. This method was considered one of the most feasible options based on a benchmarking routine that evaluated various clustering methods, including Gaussian mixture models (GMM), DB-SCAN, k-Means, and hierarchical clustering. The clustering performance was assessed through a combination of silhouette[64] and Calinski-Harabasz[65] indices. The cluster membership of each genus at k = 17 is included in supplementary data 6 and the taxonomic composition of each cluster is summarized in supplementary data 8.

5.2. Control taxonomic composition

Contamination during DNA extraction is extremely low (see 2.1. Extraction and supplementary fig. 20A), and we further note that these low levels are dominated by taxa belonging to the clusters 14, 15, 16, and 17 (supplementary fig. 20B). These clusters contain mammalian (incl. human) commensals and microbes common in indoor environments (supplementary data 8).

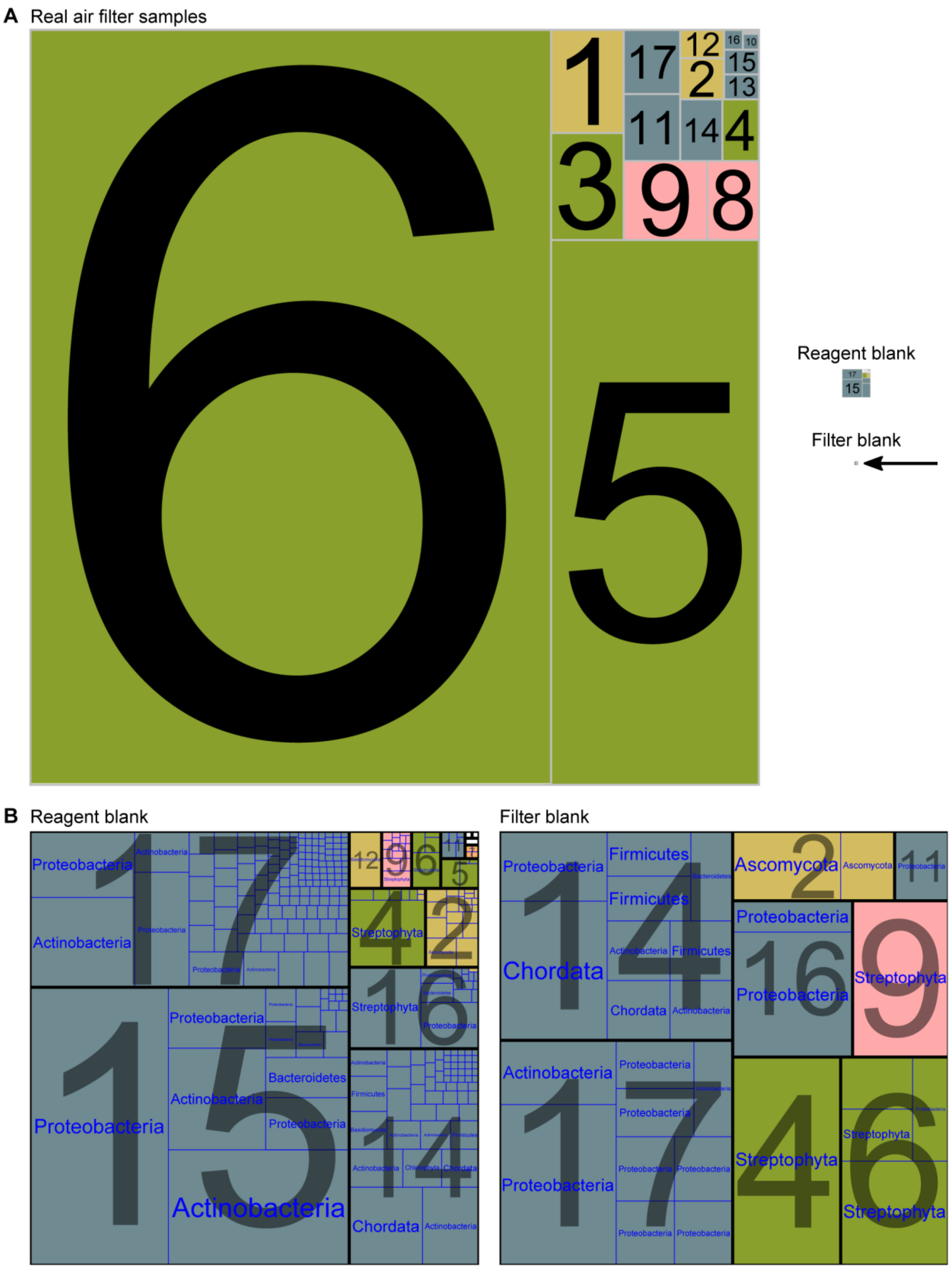

Supplementary Figure 20. The relative number of reads and taxonomic composition in controls and real samples.

A) Treemaps illustrating the relative number of reads in the average air filter sample and two control samples (one reagent blank and one filter blank, see section 2.1). The control samples were sequenced on an Illumina MiSeq flow cell, which has a lower output than the NovaSeq flow cells used for the air filter samples. To account for this, read counts from the two control samples were scaled by a factor of 20.1. The scaling factor was determined by dividing the average number of reads obtained from the air

filter sample sequencing with the number of reads obtained from the MiSeq control sequencing (2.63×10^8 / 1.311×10^7). All treemap areas are proportional to the number of reads after scaling, allowing for a comparison between controls and real samples. This illustrates the low level of cross-contamination between samples (reagent blank) and the even lower contamination during filter production (filter blank). Numbers indicate cluster membership of high-confidence taxa identified, and the clusters are coloured as in Fig 4 in the main text. **B**) Zoomed-in treemaps of the two control samples in A, showing phylum and cluster affiliations for the high-confidence taxa detected. The number and colour of each treemap field correspond to cluster affiliations, while blue text denotes phylum affiliation for the most common taxa.

6. Diversity metrics

Estimates of organism abundances can be conceptualized as probability distributions in order to measure some aspect of their diversity. For example, Shannon entropy quantifies the predictability of a probability distribution, and its exponential can be interpreted as the effective number of organisms in an ecosystem[66,67]. Similarly, Simpson diversity, another case of entropy, measures the probability that two randomly selected individuals will belong to the same species[67]. Here, the relative abundances for each week are an estimate of a probability distribution on the set of $\{1, \ldots, S\}$ genera. The collection of relative abundance estimates for all N weeks can also be considered a probability distribution $\in \{1, \ldots, N\} \times \{1, \ldots, S\}$, which we denote as the matrix P in Equation 5. Each column contains the relative abundances from week $w_1, w_2, \ldots, w_N$ and each row contains the relative abundance of each genus $p_1, p_2, \ldots, p_S$ where $\sum_n w_n = \sum_S p_S = \sum_{s,n} P_{sn} = 1$:

$$P = \begin{pmatrix} P_{11} & \cdots & P_{1n} \\ \vdots & & \vdots \\ P_{S1} & \cdots & P_{SN} \end{pmatrix} \qquad p = \begin{pmatrix} p_1 \\ \vdots \\ p_S \end{pmatrix} \qquad p_s = \sum_n P_{sn} \qquad w = (w_1, \ldots, w_N) \qquad w_n = \sum_s P_{sn} \tag{5}$$

This is equivalent to considering P_{SN} as a probability distribution $\in \{1, \ldots, S\} \times \{1, \ldots, N\}$ with marginal distributions p and w. For our data, $S = 2{,}739$ positive-classified genera (Section 4.3) and $N = 378$ sequence compositions from calendar weeks 21-41 in even-numbered years from 1974-2008.

We partitioned the diversity observed in week n into alpha (α), beta (β), and gamma (γ) diversity components following the framework of[68] and[69]. As in the Hill numbers and Shannon entropy, α-diversity here quantifies the evenness, or average rarity, of $P_{.n}$ independently from the rest of the time series. In contrast, β- and γ- diversity relate $P_{.n}$ to p, the vector of marginal relative abundances. β-diversity scales p by w_n, the size of the community in week n to measure the distinctiveness of the composition. Scaling by w_n allows comparison of changes in compositional uniqueness that are conditionally independent of α-diversity. γ-diversity measures the average rarity of taxa in week n with respect to the entire time series, that is, γ = β + α.

α-diversity is Hill diversity and equal to the exponential of Shannon entropy when $q = 1$:

$$\alpha_n = \prod_s \frac{P_{sn}}{w_n}^{-P_{sn}/w_n} \qquad q = 1 \qquad (6.1)$$

$$\alpha_n = \left(\sum_s P_{sn}\left(\frac{1}{(P_{.n}/w_n)_s}\right)^{1-q}\right)^{1/1-q} \qquad q \neq 1 \qquad (6.2)$$

Larger values of q increasingly emphasize dominant over rare taxa; ${}^{q=0}\alpha$ is taxon richness and ${}^{q=2}\alpha$ is also known as Simpson's concentration index. Higher α-diversity (for $q > 0$) indicates a more even abundance distribution, that is, a larger number of effective taxa. α-diversity obtains its maximum $\alpha = S$ if all $1, \ldots, S$ taxa are present in equal relative abundances.

β-diversity is the exponential of Rényi's relative entropy and equal to the exponential of Kullback-Leilber divergence for $q = 1$:

$$\beta_n = \prod_s \left(\frac{P_{sn}}{p_s w_n}\right)^{P_{sn}/w_n} \qquad q = 1 \qquad (7.1)$$

$$\beta_n = 1\Bigg/\left(\sum_s \frac{P_{sn}}{w_n}\left(\frac{p}{P_{.n}}\right)_s^{1-q}\right)^{1/(1-q)} \qquad q \neq 1 \qquad (7.2)$$

β-diversity measures the distinctiveness of the genera abundance distribution of the nth week relative to the entire time series. β-diversity is 1 if the composition in a week is identical to the whole time series (*i.e.*, perfectly representative) and increases as genera are more overrepresented in week n relative to $p_s w_n$ to a maximum of $\beta_n = 1/w_n$.

γ-diversity is the exponential of Rényi's cross entropy:

$$\gamma_n = \prod_s \left(\frac{1}{p_s}\right)^{P_{sn}/w_n} \qquad q = 1 \qquad (8.1)$$

$$\gamma_n = \left(\sum_s \frac{P_{sn}}{w_n}\left(\frac{1}{(p)_s}\right)^{1-q}\right)^{1/(1-q)} \qquad q \neq 1 \qquad (8.2)$$

This measures the average rarity of genera in week n relative to the whole time series. This means that if the composition of a week is identical to the marginal distribution p, its γ-diversity would equal the α-diversity of the whole time series. γ-diversity increases with evenness, as in α-diversity, and as genera are more common in week n compared to their overall rarity, up to a maximum of $\gamma_n = S/\mathrm{w}_n$.

We estimated diversity components for three sets of genera: 1) the total 2,739-genera dataset, 2) the 1,558 eukaryotic genera, and 3) the total dataset with the exclusion of the 208 genera in cluster 14, which comprised genera associated with animal microbiomes, including humans and domestic animals (supplementary data 6 and 8). These clusters include the taxa that we found to be trace contaminants in the blank control sequencing (supplementary fig. 20B). For each set, we calculated α-, β-, and γ-diversity of order q = 1, 2, and 3. Higher orders increasingly emphasize the contributions of abundant taxa, and q = 1 weighs taxa proportionally to their relative abundances (for more details and results see supplementary data 12).

6.1. Per-taxon γ-diversity contributions

We tested for significant differences in the weekly γ-diversity contributions from each genus, *i.e.*, the multiplicand in Equation 8.1, in matched calendar weeks between the early and late years of the time series using the two-sided Wilcoxon rank sum test. We initially assessed the sensitivity of the results to the years used as the 'early' and 'late' periods using comparisons between '74-'80 vs. '02-'08, '74-'82 *vs*. '00-'08, '74-'84 *vs*. '98-'08, '74-'86 *vs*. '96-'08, and '74-'88 *vs*. '94-'08. We avoided comparisons including '90 and '92 because these years correspond to the temporary peak in *Pinus* abundance and the lowest γ-diversity. With the exception of *Picea*, we found no difference in the significance of Benjamini-Hochberg adjusted *p*-values (FDR = 0.05) or the direction of change for the genera with the largest differences in γ-diversity contributions (those in Fig. 3C in the main text). *Picea* changed both signs and significance depending on the weeks used in the comparison, likely because pollen production is irregular in Norway spruce. We therefore used '74-'88 *vs*. '94-'08 for the analysis. The median per-genus difference in γ-diversity contribution, 95% confidence intervals, and Benjamini-Hochberg adjusted *p*-values are given in supplementary data 9.

7. Climatic variables

7.1. Data sources and construction

We used observations from a weather station[o] located *ca*. 3 km from the aerosol sampling station[70] and 1/24° gridded daily estimates[71] to construct 24 base variables capturing changes in the mean, variance, skewness, and kurtosis of local precipitation and temperature. Fifteen follow the ETCCDI climate extreme indices[72], including inhomogeneity adjustments[73], but we estimate their values over multiple rolling intervals. We derived 20 variables describing water and energy available for primary production from the monthly values in the 1/24° TerraClimate dataset[74]. Given the frequency of the eDNA samples, we disaggregated the Terraclim variables to weekly intervals using cubic spline interpolation such that monthly means (or sums, if applicable) remained unchanged. Similarly, we interpolated weekly values from the monthly indices of the North Atlantic[75] and Atlantic Multidecadal[76] oscillations, which influence regional temperature and precipitation. Weekly

[o] World Meteorological Organization (WMO) number: SWE00140904

values for the Arctic oscillation were calculated from daily indices[77]. All 56 base variables and their data sources are summarized in supplementary table 7.

The duration of exposure to thermal and moisture variability can modulate vital rates and phenological patterns. For example, accumulated temperature is a key signal of bud burst and insect emergence and the balance between duration and intensity influences the ability of organisms to acclimate to stressful conditions[78]. To incorporate some of this complexity into our models, we applied summary statistics to each base variable over rolling windows covering up to the previous 78 weeks. Intervals were selected to reflect local seasonal patterns between 1961 and 2009: four and eight weeks cover the period between the first (last) days consistently > 0°C and ≥ 5°C ($\hat{x}$ = 3.9, σ = 2.0); 13, 17, and 26 weeks connect the current week to conditions during the prior spring thaw (week number $\hat{x}$ = 17.8, σ = 1.5), snow melt ($\hat{x}$ = 19.7, σ = 1.1), and start of the 5°C growing season ($\hat{x}$ = 22.7, σ = 1.4); and the 52 and 78 windows include the influence of the prior growing and dormant season, with the latter including the two previous dormant seasons. Additionally, we included one- and two-week windows for daily counts of 0°C temperatures and mean daily temperature and precipitation. For disaggregated variables, we considered standard deviations over ≥ 8-week intervals due to their original monthly resolution. Note that observations are equally weighted within windows and do not incorporate time-lagged effects *per se* but values of $TNN_{52,78}$ and $TXN_{52,78}$ are determined by the previous year's winter temperatures and $TXX_{52,78}$ and $TNX_{52,78}$ by summer.

Supplementary Table 7. Summary of climatic covariables.

name	base description	*f*(x)	windows (weeks)
AET[a]	Actual Evapotranspiration; weekly total water extracted from plants and soil	x̄	4,8,13,17,26,52,78
		σ	8,13,17,26,52,78
AMOI[b]	Atlantic Multidecadal Oscillation Index; weekly mean	x̄	4,8,13,17,26,52,78
		σ	8,13,17,26,52,78
AMOI.LP[b]	10-yr low-pass Atlantic Multidecadal Oscillation Index; weekly mean	x̄	4,8,13,17,26,52,78
		σ	8,13,17,26,52,78
AOI[c]	Arctic Oscillation Index: daily Hurrell station-based value	x̄	4,8,13,17,26,52,78
		σ	8,13,17,26,52,78
DS[d]	Dry spells; ≥ 6 consecutive days with < 1 mm precipitation	Σ	4,8,13,17,26,52,78
CSD[e]	Cold spell duration; ≥ 6 consecutive days where T_{MIN} < 10th percentile†	Σ	4,8,13,17,26,52,78
WS[d]	Wet spells; ≥ 6 consecutive days with ≥ 1 mm precipitation	Σ	4,8,13,17,26,52,78
deficit[a]	Deficit: difference between weekly PET and AET totals	x̄	4,8,13,17,26,52,78
		σ	8,13,17,26,52,78
DTR[e]	Diurnal temperature range; difference between daily T_{MIN} and T_{MAX}†	x̄	4,8,13,17,26,52,78
FCF[e]	Frost change frequency; days where T_{MIN} < 0°C and T_{MAX} > 0°C	%	1,2,4,8,13,17,26,52,78
FD[e]	Frost days; T_{MIN} < 0°C†	Σ	1,2,4,8,13,17,26,52,78
ID[e]	Ice days; T_{MAX} < 0°C†	Σ	1,2,4,8,13,17,26,52,7

			8
NAOI[f]	North Atlantic Oscillation Index; weekly mean Hurrell station-based value	x̄<br>σ	4,8,13,17,26,52,78<br>8,13,17,26,52,78
PD[a]	Potential deficit; difference between weekly precipitation and PET totals	x̄<br>σ	4,8,13,17,26,52,78<br>8,13,17,26,52,78
PDSI[a]	Palmer Drought Severity Index; weekly mean	x̄<br>σ	4,8,13,17,26,52,78<br>8,13,17,26,52,78
PET[a]	Potential evapotranspiration; weekly total Penman-Montieth reference evapotranspiration	x̄<br>σ	4,8,13,17,26,52,78<br>8,13,17,26,52,78
precip[d]	Total daily precipitation	x̄<br>σ	1,2,4,8,13,17,26,52,7<br>8
pressure[e]	Daily mean atmospheric pressure	x̄<br>σ	4,8,13,17,26,52,78<br>8,13,17,26,52,78
radiation[a]	Weekly total downward surface shortwave solar radiation	x̄<br>σ	4,8,13,17,26,52,78<br>8,13,17,26,52,78
RM10[d]	Days with ≥ 10 mm precipitation†	Σ	4,8,13,17,26,52,78
runoff[a]	Weekly total precipitation and snowmelt exceeding PET and soil recharge	x̄<br>σ	4,8,13,17,26,52,78<br>8,13,17,26,52,78
RX1day[d]	Maximum 1-day precipitation†	Σ	4,8,13,17,26,52,78
soil[a]	Weekly total soil column moisture	x̄<br>σ	4,8,13,17,26,52,78<br>8,13,17,26,52,78
SWE[a]	Snow water equivalent; amount of liquid water in snow pack	x̄<br>σ	4,8,13,17,26,52,78<br>8,13,17,26,52,78
TAVG[d]	Daily mean temperature	x̄<br>σ	1,2,4,8,13,17,26,52,7<br>8
TMAX[e]	Daily maximum temperature	x̄	1,2,4,8,13,17,26,52,7<br>8
TMIN[e]	Daily minimum temperature	x̄	1,2,4,8,13,17,26,52,7<br>8
TN10p[e]	Cool nights; days where $T_{MIN} < 10^{th}$ percentile†	%	4,8,13,17,26,52,78
TN90p[e]	Warm nights; days where $T_{MIN} > 90^{th}$ percentile†	%	4,8,13,17,26,52,78
TNN[e]	Minimum daily T_{MIN}†	min	4,8,13,17,26,52,78
TNX[e]	Maximum daily T_{MIN}†	max	4,8,13,17,26,52,78
TX10p[e]	Cool days; days where $T_{MAX} < 10^{th}$ percentile†	%	4,8,13,17,26,52,78
TX90p[e]	Warm days; days where $T_{MAX} > 90^{th}$ percentile†	%	4,8,13,17,26,52,78
TXN[e]	Minimum daily T_{MAX}†	min	4,8,13,17,26,52,78
TXX[e]	Maximum daily T_{MAX}†	max	4,8,13,17,26,52,78
VP[a]	Vapor pressure; weekly mean atmospheric pressure exerted by water vapor	x̄<br>σ	4,8,13,17,26,52,78<br>8,13,17,26,52,78
VPD[a]	Vapor pressure deficit; weekly mean difference between saturated vapor pressure and actual vapor pressure	x̄<br>σ	4,8,13,17,26,52,78<br>8,13,17,26,52,78

WSD[e]	Warm spell duration; ≥ 6 consecutive days where T_{MAX} > 90th percentile†	Σ	4,8,13,17,26,52,78

a – TerraClimate[74]; b – Trenberth and Shea[76]; c – Climate Prediction Center, NOAA[77]; d – PTHBV v. 3.0[71]; e – Menne et al. 2012[70], station code: SWE00140904; f – Hurrel[75]; †ETCCDI index[72].

7.2. Variable selection

We first excluded variables with > 50% zero-valued observations during the aerosol sampling period (weeks 21-41), which removed 16 related to cold spells, ice days, frost days, and consecutive wet days. Then, we used the `findCorrelation` function in the *R* package 'caret' *v.* 6.0-93 to identify the largest subset with all pairwise $|\hat{\rho}| < 0.70$. We curated this subset to include variables with potentially greater mechanistic importance or clearer interpretations over those that simply maximized the size of the regressor matrix (e.g., VPD over PDSI, FCF_{17} over $runoff\_sd_{13}$). The final regressor matrix comprised 75 variables with pairwise $|\hat{\rho}| = 0.15$ $(\sigma = 0.13)$ and is provided in supplementary data 10.

7.3. Missing year interpolation

Daily measurements for TMIN, TMAX and air pressure were not reported from 1993-1995 by the nearest weather station.[p] In practice, this resulted in 21 missing observations for their derived variables. We initially considered using other nearby weather stations[70] to supplement the observations but they either also lacked these measurements or their temporal coverage did not overlap sufficiently to assess potential inhomogeneity. Therefore, we interpolated values for 1994 for the 18 affected variables: $pressure_{4,8,26}$, $DTR_{4,13,52}$, $FCF_{17,26,78}$, FD_{365}, $TN10p_{4,13}$, $TN90p_{4,26}$, TNX_{52}, TXN_{52}, and $TXX_{26,52}$. We followed the state space model framework described in section 8, with the following modifications: 1) we used the entirety of the reported data from 1959-2008 to inform parameter estimation, 2) only trigonometric seasonal dummy variables were included in the regressor matrix, and 3) we considered the model with the lowest cumulative one-step-ahead forecast errors to be the best prediction. We examined the rank-transformed time series and considered the imputed 1994 estimates to be plausible, especially for variables calculated over longer periods or with long-term trends or cycles (supplementary fig. 21).

[p] World Meteorological Organization (WMO) number: SWE00140904

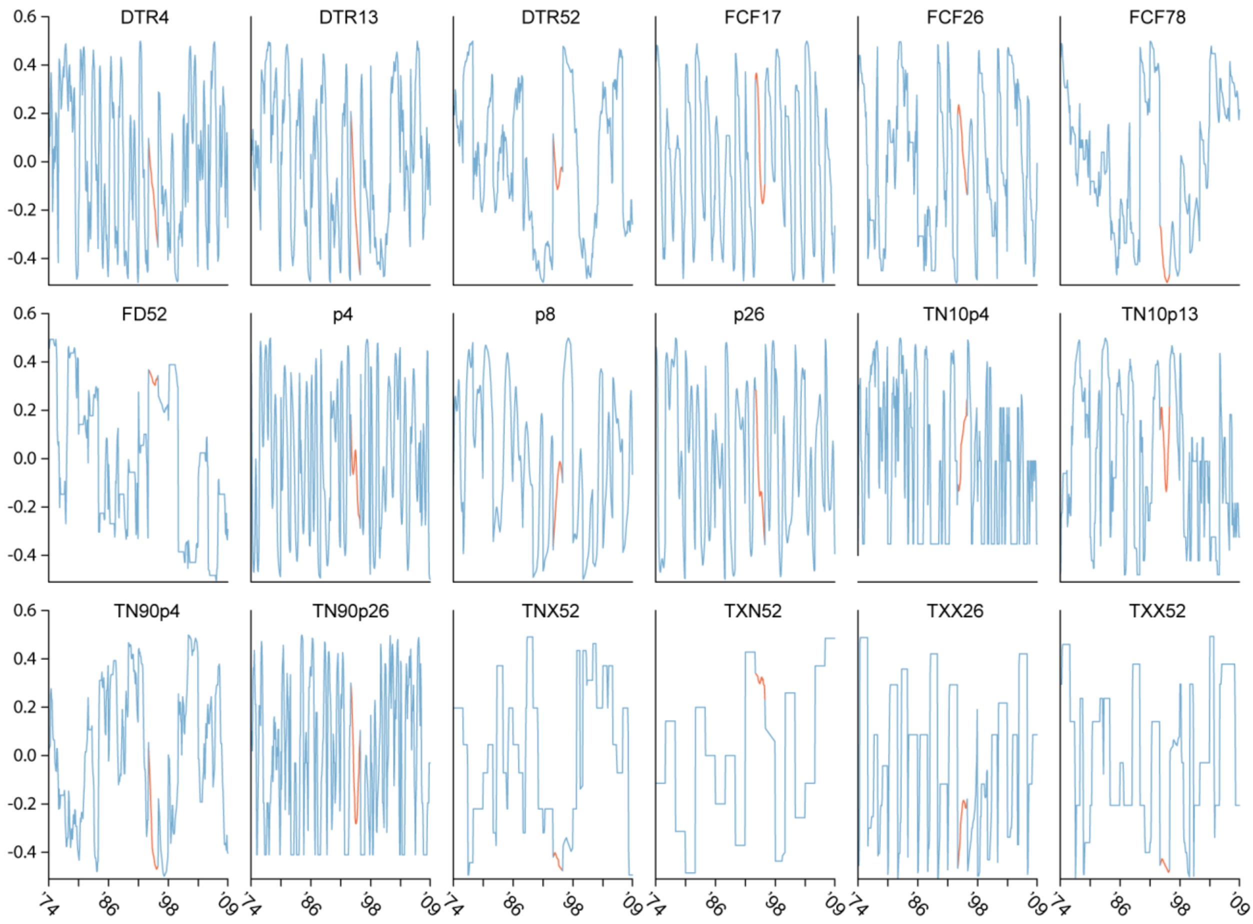

Supplementary Figure 21. Climatic covariables with imputed values for 1994.
Rank-transformed observed data are shown in blue and imputed values in orange.

7.4. Variable clustering and categorization

We related each of the original 393 variables to their larger 'climatic' neighborhood using densMAP[q;79] combined with hdbscan[r;80] with 'densvis' *v.* 1.8.1[79] and 'dbscan' *v.* 1.1-11[81] for *R*, respectively. Informally, variables within a neighborhood describe the same, or a similar, climatic feature while those in distant neighborhoods are more likely generated by a different latent process.

Both densMAP and hdbscan are sensitive to hyperparameter choices. In the absence of a more objective cost function, we considered hyperparameter combinations with higher classification rates to be better summaries of the climatic data. We conducted a grid search over the densUMAP and hdbscan parameters: `n_neighbors = c(10, 15, 20, 25)`, `n_components = c(10, 20, 30, 40, 60)`, `lambda = c(0.05, 0.1, 0.15)`, `metric = c("correlation", "cosine", "manhattan", "euclidean")`, `min_samples = c(10, 11, 12, 13, 14, 15, 16, 17, 18)`. Cluster number varied by `min_samples`, which directly specifies the smallest permitted cluster size but no other hyperparameter had a clear individual effect, nor did any independently influence the

[q] density-preserving manifold approximation and projection
[r] hierarchical density-based spatial clustering of applications with noise

classification rate. Combinations with classification rates above the 75th percentile (n = 32, mean = 95.53%) most frequently resolved 3 and 7 clusters (n = 16 and 9, respectively). We compared the climatic variable assignments at k = 3 and k = 7 to assess their stability. Assignments differed primarily in resolution and in identity of unclassified variables, although the Manhattan distance differed in both cases and additionally produced hierarchically incompatible k = 3 and k = 7 assignments. The remaining three k = 7 assignments differed by only a single successfully classified variable and were consistent with the k = 3 results.

We considered the k = 7 assignments as the best estimate of high-dimensional neighborhood space and examined each group to identify common features. Based on this, we suggest our climatic variables can be summarized as aspects of seven latent axes:

1) precipitation, which includes precipitation variables with < 52-week intervals;
2) water storage, comprising most runoff and soil moisture variables with ≥ 8-week intervals, running means of the PDSI, and precipitation variables with ≥ 52-week intervals;
3) snow accumulation, inferred from the inclusion of ≥ 52-week snow water variables and running means of the NAO and AO indices;
4) warming trend, based on the inclusion of most temperature-derived variables with ≥ 52-week intervals and all estimates of TN90p and TX90p;
5) seasonal transitions, which consists of variables delimiting the potential vegetative growth period, including the recent number of frost and ice days, temperature variability, and short window estimates of runoff, snow cover, radiation, PET, and AET;
6) evapotranspiration, a group with similar base variables as seasonal transitions but with ≥ 8-week windows, in addition to most sub-annual temperature variables and estimates of water deficit and soil moisture variability; and
7) the Atlantic Multidecadal Oscillation, which simply consists of running means of both AMO indices.

Like the climatic variables themselves, these categories are an abstraction intended to represent the environment experienced by a hypothetical organism. However, we use them as a heuristic device because they help clarify the kind of variation represented by abstruse regressors (*e.g.*, the standard deviation of the NAOI falls on the ‘seasonal transitions’ axis), and they emphasize the relationship between trends and potentially more proximate factors, rather than a single index.

8. Time series analysis

8.1. Introduction to state-space models

We used linear state-space models (SSMs) to analyze eDNA and traditional count-based time series. Such models consider time series data to be the result of two connected stochastic systems: 1) a hidden, or latent, process that generates variation across time, and 2) a measurement process that allows discrepancies between the latent state and observed data.

The relationship between sequenced eDNA from a given taxon y and the true DNA abundance in the catchment area μ, for example, can be written:

$$y_t = \mu_t + \varepsilon_t, \qquad \varepsilon_t \sim N(0, \sigma_\varepsilon^2)$$
$$\mu_t = \mu_{t-1} + \eta_t, \qquad \eta_t \sim N(0, \sigma_\mu^2) \tag{9}$$

where

- y is the vector of eDNA abundance at time steps $t = 1 \ldots T$,
- ε is measurement error with variance σ_ε^2,
- μ is the corresponding latent population size,
- and η represents variation in μ with variance σ_μ^2.

Recursive algorithms, most commonly the Kalman filter[82], solve Equation 9 by formalizing the intuition that the historic performance of a model can be used to refine future predictions. The filter computes $p(x_t | y_{1:t-1}, \sigma_\varepsilon^2, \sigma_\mu^2)$ and uses the prediction errors $v_t = x_t - y_t$ and their variance F_t to obtain minimum-variance unbiased estimates of x_t and the system parameters, in this case, σ_ε^2 and σ_μ^2[83]. SSMs fit by a Kalman filter can be framed in maximum-likelihood or Bayesian terms, and we employ both as a matter of accessibility given the available implementations suitable for ecological time series.

8.2. eDNA abundance and diversity trends

8.2.1. Structural time series models

We modeled eDNA abundances observed in calendar weeks 21-41 of each year using the *R* package 'bsts' *v*. 0.9.9[84,85]. Here, the simple model in Equation 9 is extended to include a second latent state, δ_t, to allow a stochastic directional trend:

$$\mu_t = \mu_{t-1} + \delta_{t-1} + \eta_{\mu,t}, \qquad \eta_{\mu,t} \sim N(0, \sigma_\mu^2) \tag{10.1}$$

$$\delta_t = \delta_{t-1} + \eta_{\delta,t}, \qquad \eta_{\delta,t} \sim N(0, \sigma_\delta^2) \tag{10.2}$$

$$y_t = \mu_t + D^T d_t + \varepsilon_t, \qquad \varepsilon_t \sim N(0, \sigma_\varepsilon^2) \tag{10.3}$$

This is known as a local linear trend (LLT) or 'random walk with drift' model. If σ_δ^2 approaches zero but σ_μ^2 does not, the model reduces to the local level (LL) in Equation 9 and indicates that μ is equally likely to increase as decrease at each time step. Conversely, a relatively large σ_δ^2 with σ_μ^2 approaching zero results in an integrated random walk (IRW) model, where μ changes according to a stochastic but directional trend[86].

We tested for potential responses to climatic (Section 7) and aerosol dispersion-related (Section 1.3.1) variation by comparing predictive power of Equation 10 using three different sets of d_t covariables:

1) six variables representing generic seasonal patterns, defined by the trigonometric function[83]:

$$D^T d_t = \sum_{j=1}^{J} \widetilde{D}_j \cos \lambda_j t + \widetilde{D}_j^* \sin \lambda_j t,\ \lambda_j = \frac{2\pi j}{S}, j = 1 \dots 3, S = 21 \quad (11)$$

where λ denotes the jth harmonic and S specifies the length of the season;

2) these combined with the 75 climatic variables described in Section 7;
3) or the trigonometric seasonality combined with the particle dispersion variables described in Section 1.3.1.

Our SSMs are limited to linear Gaussian cases, but ecology theory predicts unimodal or skewed responses to environmental variation[87]. Therefore, we tested five transformations (Yeo-Johnson, exponential, minmax, ranks, and standard scores) of the covariate matrices in a regression model for each of the 17 cluster abundances. We compared their forecast errors using the diagnostic tests in Section 8.2.3 to identify which transformation best conformed with model assumptions for the majority of the clusters. Rank transformation was most consistently adequate for the climatic regressors whereas all transformations performed well with the particle dispersion variables. For better comparability between the models, we applied the rank transformation to both regressor matrices.

8.2.2. Prior distribution specifications

Completing the model in Equation 10 requires specifying prior distributions on the estimated parameters $\theta = \sigma_\varepsilon^2, \sigma_\mu^2, \sigma_\delta^2, D$. In 'bsts,' variance terms are drawn from the gamma distribution:

$$\frac{1}{\sigma^2} \sim \Gamma(\alpha, \beta) \quad (12)$$

with mean α/β and variance α/β^2. A hierarchical spike-and-slab prior is placed on the vector of regression coefficients D, where ζ is a Bernoulli distributed variable determining if $D = 0$ for each of the $1 \dots K$ covariates:

$$\zeta \sim \prod_{k=1}^{K} \pi_k^{\zeta_k} (1 - \pi_k)^{1-\zeta_k} \quad (13)$$

or is otherwise drawn from

$$D_\zeta | \sigma_\varepsilon^2 \sim N\left(0, \sigma_\varepsilon^2 \left(\Omega_\zeta^{-1}\right)^{-1}\right), \qquad \frac{1}{\sigma_\varepsilon^2} \sim \Gamma(\alpha, \beta) \tag{14}$$

where

$$\Omega^{-1} = g\big(wd^T d + (1-w)diag(d^T d)\big) \tag{15}$$

and Ω_ζ^{-1} denotes the rows and columns of Ω^{-1} where $\zeta = 1$. Equation 15 reduces to Zellner's g prior when the diagonal shrinkage parameter w is zero. More simply, Ω^{-1} conveniently scales the prior distribution on D_ζ based on the covariance structure of the subset of covariates sampled in a particular draw.

We defined the priors on σ_δ^2 and σ_μ^2 to enforce two cases of Equation 10: the local linear trend (LLT) and the integrated random walk (IRW). We compared these models explicitly because we found that in practice, LLT models simplified to an LL process when σ_δ^2 was negligible but not to an IRW process unless both σ_μ^2 and σ_ε^2 approached zero, an unlikely scenario for eDNA time series. This result is not surprising given the difficulties of estimating process error when measurement error is high. As we considered an IRW process with high measurement error and a small slope to be a plausible alternative to the LLT, we chose to enforce this outcome by fixing σ_μ^2 to an arbitrarily small value.

In a set of pilot runs on clusters 17 (prokaryotes), 8 (insects), and 5 (plants), posterior estimates were generally insensitive to priors on and over the unique combinations of $\alpha = \{0.01, 0.05, 0.1, 0.5, 1, 2\}$ and $\beta = \{10^{-2}s_y^2, 5^{-2}s_y^2, 10^1 s_y^2, 2^{-1}s_y^2\}$, where s_y^2 is the sample variance of the time series. However, MCMC diagnostics (Section 8.2.3) favored $\alpha = 1$. We then selected β with the expectation that measurement error is the largest source of variance, followed by σ_μ^2 and σ_δ^2, respectively. Prior distributions used in the production models are given in supplementary table 8.

Supplementary Table 8. Prior distributions used in the production models.

model	regressors	abbreviation	σ_μ^2	σ_δ^2	Ω^{-1}	π_k	σ_ϵ^2
local linear trend	harmonics	LLT-base	$\alpha = 1$<br>$\beta = 10^{-1} s_y^2$<br>$\sigma_\mu^2 \leq s_y^2$	$\alpha = 1$<br>$\beta = 10^{-2} s_y^2$<br>$\sigma_\delta^2 \leq 0.5\, s_y^2$	$g = 1$<br>$w = 0.5$	$\frac{2}{7}$	$\alpha = 1$<br>$\beta = 0.5\, s_y^2$<br>$\sigma_\epsilon^2 \leq s_y^2$
	harmonics & climate	LLT-climate				$\frac{3}{41}$	$\alpha = 1$<br>$\beta = 0.75\, s_y^2$<br>$\sigma_\epsilon^2 \leq s_y^2$
	harmonics & catchment	LLT-particle				$\frac{2}{27}$	$\alpha = 1$<br>$\beta = 0.75\, s_y^2$<br>$\sigma_\epsilon^2 \leq s_y^2$
integrated random walk	harmonics	IRW-base	10^{-4}	$\alpha = 1$<br>$\beta = 10^{-2} s_y^2$<br>$\sigma_\delta^2 \leq 0.5\, s_y^2$	$g = 1$<br>$w = 0.5$	$\frac{2}{7}$	$\alpha = 1$<br>$\beta = 0.5\, s_y^2$<br>$\sigma_\epsilon^2 \leq s_y^2$
	harmonics & climate	IRW-climate				$\frac{3}{41}$	$\alpha = 1$<br>$\beta = 0.75\, s_y^2$<br>$\sigma_\epsilon^2 \leq s_y^2$
	harmonics & catchment	IRW-particle				$\frac{2}{27}$	$\alpha = 1$<br>$\beta = 0.75\, s_y^2$<br>$\sigma_\epsilon^2 \leq s_y^2$

8.2.3. Model fit and convergence diagnostics

For pilot runs exploring data transformations and prior specifications, we relied primarily on omnibus tests applied to posterior mean forecast errors to efficiently compare dozens of models. Following Commandeur and Koopman[88] and Durbin and Koopman[83], we used the F variance ratio between the first (t = 2, …, 121) and last (t = 254, …, 378) thirds of the time series, the magnitude and significance of autocorrelation in the first 42 lags, and Kolomogorov-Smirnov's d to test for heteroscedasticity, serial dependence, and non-normality, respectively. Convergence was evaluated by calculating effective sample sizes (ESS) for each parameter, Geweke's convergence diagnostic[89], Raftery and Lewis's diagnostic[90] with the *R* package 'coda' *v.* 0.19-4[91] and through visual inspection of parameter trace plots. For final model runs, we verified these summary statistics using diagnostic plots of the posterior forecast error and latent state distributions and assessed identifiability by plotting univariate prior and posterior distributions, likelihood profiles, and joint posterior distributions. Pilot models were run for 10^5 and final models for 10^6 MCMC iterations, with 10% discarded as burn-in.

Some models, primarily of bacteria-dominated cluster abundances, exhibited substantial evidence against normal, identical, and independent (IID) errors in all tested combinations of priors, time series models, and covariate transformations. Poor model performance was most likely caused by a small number of extreme observations in all cases. We removed data points more extreme than 1.5 × the interquartile range in log space for both abundances and diversity metrics. Forecast errors were approximately IID after removing these outliers.

Diagnostic summary statistics and the transformations applied, if any, for all production runs in supplementary data 11.

8.2.4. Leave-future-out cross validation

We compared the predictive accuracy of models with different trend and regression specifications using the exact expected log pointwise predictive density (ELPD) estimated by leave-future-out cross validation (LFO)[92,93]. Time series models that more accurately predict the next M future observations conditioned on data from $t = 1 \ldots t_{M-1}$ are more likely to be well-specified and have generalizable parameter estimates. Thus, we computed the expected log-predictive densities $p(y_{t+1:M}|y_{1:t})$ for each $t \in \{L, \ldots, N-M\}$, where L is the minimum number of observations considered before making predictions ahead, N the sample size, and M the number of future observations:

$$\text{ELPD}_{LFO} = \sum_{t=L}^{N-M} \log p(y_{t+1:M} \mid y_{1:t}) \tag{16}$$

We used 21-step-ahead predictions (the number of time points in a year in our dataset) for the last 57 weeks (15%) of the time series, *i.e.*, $M = 21$, $L = 300$ and $N = 378$. This process refits the time series model for each $t \in \{L, \ldots, N-M\}$ and uses S random draws $\left(\theta_{1:t}^{(1)}, \ldots, \theta_{1:t}^{(S)}\right)$ from the posterior distribution $p(\theta|y_{1:t})$ to calculate the log likelihood of $p(y_{t+1:M}|y_{1:t})$:

$$\text{ELPD}_{LFO} = \log p(y_{t+1:M} \mid y_{1:t}) \approx \frac{1}{S}\sum_{s=1}^{S} p(y_{t+1:M} \mid y_{1:t}, \theta_{1:t}^{(s)}) \tag{17}$$

We used $S = 3.6 \times 10^5$ (*i.e.*, 4×10^5 iterations with the first 10% discarded as burn in) for cross-validation. Obtaining 57 forecasts for each model is still time consuming, but each model only needs to be fit to the full dataset once because the Kalman recursions can be re-filtered to obtain $p(y_{t+1:M} \mid y_{1:t})$ at each $t \in \{L, \ldots, N-M\}$. We considered a model to be the best among the candidates if the ELPD difference divided by the standard error of the difference was > 2 in all pairwise comparisons[94]. If one or more models were similarly supported, we preferred the model with the fewest number of parameters and/or the smaller regressor matrix.

8.3. Abundance trends from traditional monitoring data

8.3.1. Data acquisition

We conducted an extensive search of publicly-available data and consulted with government authorities to identify monitoring surveys within 100 km of the aerosol sampling station with at least seven years of data between 1973-2008. We found two programs meeting

these initial requirements: the Swedish Bird Survey[s;95] and the Swedish Electrofishing Register.[t] However, electrofishing data for the river closest to the aerosol sampling station, the Torne (< 5 km), was only available for four years after 2003. Our initial models indicated different population trajectories among and within river catchments, which suggests that the electrofishing data may not adequately represent the area closest to the aerosol sampling station. We excluded fish from further consideration, leaving birds for comparison.

The Swedish Bird Survey comprises point observations collected by volunteers according to a standardized protocol along predefined routes. We narrowed our search to routes surveyed for ≥ 5 years and with ≥ 10 total counts of a genus represented in the filter sequences. This initially resulted in nine genera: *Anas* (Anseriformes: Anatidae), *Corvus* (Passeriformes: Corvidae), *Cuculus* (Cuculiformes: Cuculidae), *Ficedula* (Passeriformes: Muscicapidae), *Gavia* (Gaviiformes: Gaviidae), *Lagopus* (Galiformes: Phasianidae), *Parus* (Passeriformes: Paridae), *Phylloscopus* (Passeriformes: Phylloscopidae), and *Saxicola* (Passeriformes: Muscicapidae). These were subjected to validation (Section 4.4) to ensure the robustness of the eDNA classifications. *Gavia* was the only genus without clear support, and was excluded from further analysis. For the three genera with multiple species in the Kiruna region (*Corvus corax* and *carone*; *Anas crecca* and *penelope*; and *Lagopus lagopus* and *muta*), we summed the counts and analyzed them as a single genus.

8.3.2. State space models

We modeled abundance trends from the count data using SSMs as implemented in the *R* package 'MARSS' *v.* 3.11.4[96]. For each genus, we considered survey routes as observers of the same latent population trend but with potentially different autoregressive (AR) errors[97]. This allows each survey route to be influenced by local conditions and have different random error rates. In MARSS notation, this model is written:

$$\begin{bmatrix} a \\ x_1 \\ x_2 \\ x_n \end{bmatrix}_t = \begin{bmatrix} 1 & 0 & 0 & 0 \\ 0 & b_1 & 0 & 0 \\ 0 & 0 & b_2 & 0 \\ 0 & 0 & 0 & b_n \end{bmatrix} \begin{bmatrix} a \\ x_1 \\ x_2 \\ x_n \end{bmatrix}_{t-1} + \begin{bmatrix} e \\ w_1 \\ w_2 \\ w_n \end{bmatrix}_t , \begin{bmatrix} e \\ w_1 \\ w_2 \\ w_n \end{bmatrix}_t \sim MVN\left(0, \begin{bmatrix} 1 & 0 & 0 & 0 \\ 0 & q_1 & 0 & 0 \\ 0 & 0 & q_2 & 0 \\ 0 & 0 & 0 & q_n \end{bmatrix}\right) \quad (18.1)$$

$$\begin{bmatrix} y_1 \\ y_2 \\ y_n \end{bmatrix}_t = \begin{bmatrix} 1 & 1 & 0 & 0 \\ 1 & 0 & 1 & 0 \\ 1 & 0 & 0 & 1 \end{bmatrix} \begin{bmatrix} a \\ x_1 \\ x_2 \\ x_n \end{bmatrix}_t \quad (18.2)$$

where

- a is the latent trend observed by all routes at time *t*;
- b_n is the AR(1) parameter (φ) for the $1 \ldots n^{th}$ route;
- x_n is the AR(1) trend for each route at time *t*;
- and w_n is the observation error for the $1 \ldots n^{th}$ route at time *t* with variance q_n.

[s] Svensk Fågeltaxering; http://www.fageltaxering.lu.se
[t] https://www.slu.se/institutioner/akvatiska-resurser/databaser/elfiskeregistret

We fit maximum-likelihood models for each genus via the EM algorithm with the options: `minit = 500, maxint = 2000, abstol = 1e-6, conv.test.slope.tol = 1e-6`. Because the variance of the estimated shared trend was high for most genera (supplementary fig. 22), we calculated the two-year centered moving average before extracting even-numbered years between 2000-2008 for comparison with the eDNA estimates. Our results are similar to estimates from the Norrbotten Country Board[u;98], which suggests trends estimated from the Swedish Bird Survey's standardized routes are robust to analysis method.

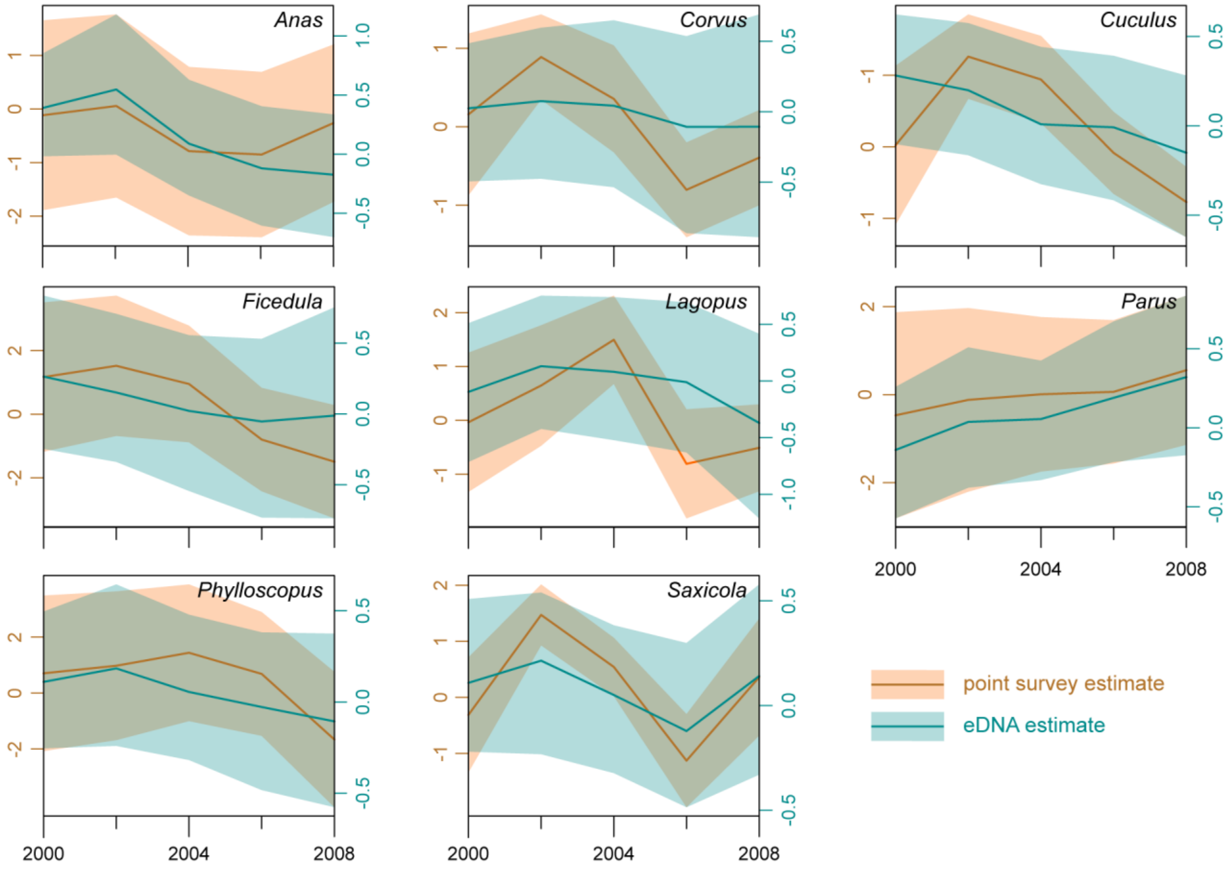

Supplementary Figure 22. Scaled annual abundances indices for eight bird genera estimated from point surveys (orange) and PLR transformed eDNA (blue).
Shaded regions show 95% confidence and credible intervals for point surveys and eDNA estimates, respectively. Note different *y*-scales are used for the two data sources.

Models of PLR-transformed eDNA abundances were estimated following the methods in Section 8. Models using the 'climate' regressors produced the best 1-year-ahead forecasts according to ELPD differences (Section 8.2.4) for five genera and were tied for the top rank for all genera (supplementary data 11). The LLT and IRW trend models performed similarly, but MCMC diagnostics (8.2.3) suggested the LLT models had convergence issues for some genera (supplementary data 11). We therefore used the 'irw climate' models for all genera and calculated annual averages from the posterior median state (supplementary fig. 22). We

[u] Länsstyrelsen Norrbotten

z-transformed the averaged eDNA and count estimates and estimated their correlation with ordinary least squares regression.

9. Land use

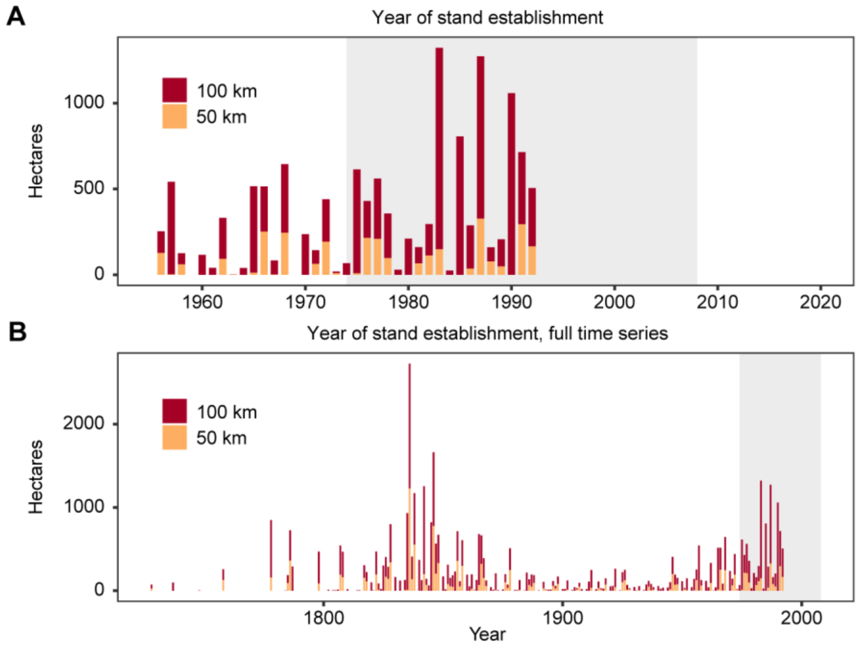

Supplementary Figure 23. Forest harvests outside formally protected areas in northern Sweden.

A) year of stand establishment within 50 and 100 km of the aerosol sampling station from 1955 to 1993, and **B**) with all available records, 1793-1993. Shaded areas in each panel denote years overlapping with the eDNA time series.

Forests have been a dominant, continuous presence in northern Fennoscandia since shortly after the last glacial maximum. Until the 19th century, the indigenous Sami people were the majority inhabitants of the region and primarily engaged in reindeer pastoralism, hunting, fishing, and low-intensity agriculture[99,100]. Colonization by Swedish and Finnish-speaking agriculturalists began by the early 17th century, but demographic and land use changes occurred slowly until the second half of the 19th century[99] or as recently as the 1880s around Kiruna[101]. We used stand age data from the Comprehensive Forest Inventory[v] to estimate the timing and spatial extent of stand-replacing disturbances within 50 and 100 km of the aerosol sampling station[102]. Consistent with the broader forest history in northern Fennoscandia[100], we found two peak periods of canopy conversions, first in the mid-1800s and later in the 1980s (supplementary fig. 23). Note that the inventory was conducted from 1982-1993 and stand establishments are likely underestimated during this period. Fire may have caused some portion of the canopy loss in the 1700 and 1800s, but clearcuts have been the dominant stand-replacing disturbance since the 1900s.

[v] Översiktlig skogsinventering; a national inventory of privately-owned property > 20 hectares conducted between 1982 and 1993

Contemporary land use comprises commercial forestry, nature-oriented tourism, reindeer husbandry by the Sámi people, and large-scale mining operations. The town of Kiruna (population 23,000), inactive open-pit mines (1900-1960), and a large underground iron mine (1960-present) lie 10 km west of the aerosol station. Another iron mine located near the town of Gällivare *ca*. 90 km southwest operated open-pit until the 1960s, and the nearby Aitik open-pit copper mine was established in 1968. A smaller open-pit mine 35 km to the southeast operated from 1965-1983. A large, contiguous network of formally-protected nature reserves spans much of the subalpine zone west of the aerosol sampling station (supplementary fig. 24).

Reindeer husbandry is a keystone of Sámi cultural heritage and identity across northern Fennoscandia. Most of the approximately 450,000 reindeer in the region are domesticated, with only ~7,500 wild individuals remaining in the Kola Peninsula[103]. In Norway and Sweden, reindeer husbandry is a protected right held exclusively by the Sámi people, whereas Finnish legislation allows any European citizen to herd reindeer. In Sweden, reindeer management is organized by 51 Sámi villages[w], a term that, despite its name, refers to economic and administrative associations regulated under the Reindeer Husbandry Act[x].

Reindeer management in Sweden and Norway is primarily pastoral, with Sámi herders guiding them between summer and winter grazing areas[104–106]. In Sweden, most herds are migratory, spending summers in alpine meadows before moving 70–250 km to lowland boreal forests for the rest of the year[106]. In Norway, summer grazing occurs along the Atlantic coast, while in winter, herds move to interior tundra and mountain birch forests[106]. In contrast, Finland's reindeer husbandry is largely sedentary, with only small-scale movements between grazing grounds due to historical and socioeconomic factors[107].

While reindeer are managed in herds, some individuals—primarily males—stray from their groups and remain in winter grazing regions year-round, including areas near the aerosol sampling station. Migration routes of three Sámi villages—Laevas, Gabna, and Talma—pass within 10 km of the station, with approximately 43,000 reindeer following these routes annually[y].

9.1. Forest management

Forestry outside the subalpine zone is intensive and extensive relative to other boreal regions. For example, the Swedish National Forest Inventory[z] (NFI)[108] reports *ca*. 14% of the total forested area in northern Norrland[å] was felled between 1986-2016[109], compared to 10% of the eastern boreal shield region (roughly, Ontario and further east) and 4% of the western shield[110]. From 1982 to 2008, *ca*. 33% of northern Norrland forests received at least one silvicultural treatment[109].[ä] The Swedish National Land Cover Database, constructed from

---

[w] Samebyar
[x] Rennäringslagen 1971:437
[y] Data from Sámediggi (Sámi Parliament of Sweden; Sametinget); https://www.sametinget.se/samebyar; last accessed 26/02/2025
[z] Riksskogstaxeringen[108]
[å] a historic region used for statistical reporting comprising the two northernmost provinces of Sweden
[ä] calculated as the sum of hectares 'cleaned' (röjning in Swedish), thinned or felled and the total forest area (skogsmark) including alpine regions in 2020 for northern Norrland; data available from the Swedish National Forest Inventory[109]

2017-2019 using satellite and LiDAR data, classified 16.5% of forests within 350 km of the aerosol station (19.5% in Norbotten county) as ‘temporarily non-forested’, that is, regrowing stands with a canopy height < 5 m[1] (supplementary fig. 24). Given the site indices[ö] typical of the region[111], these stands were likely younger than 20-40 at the time of the database construction.

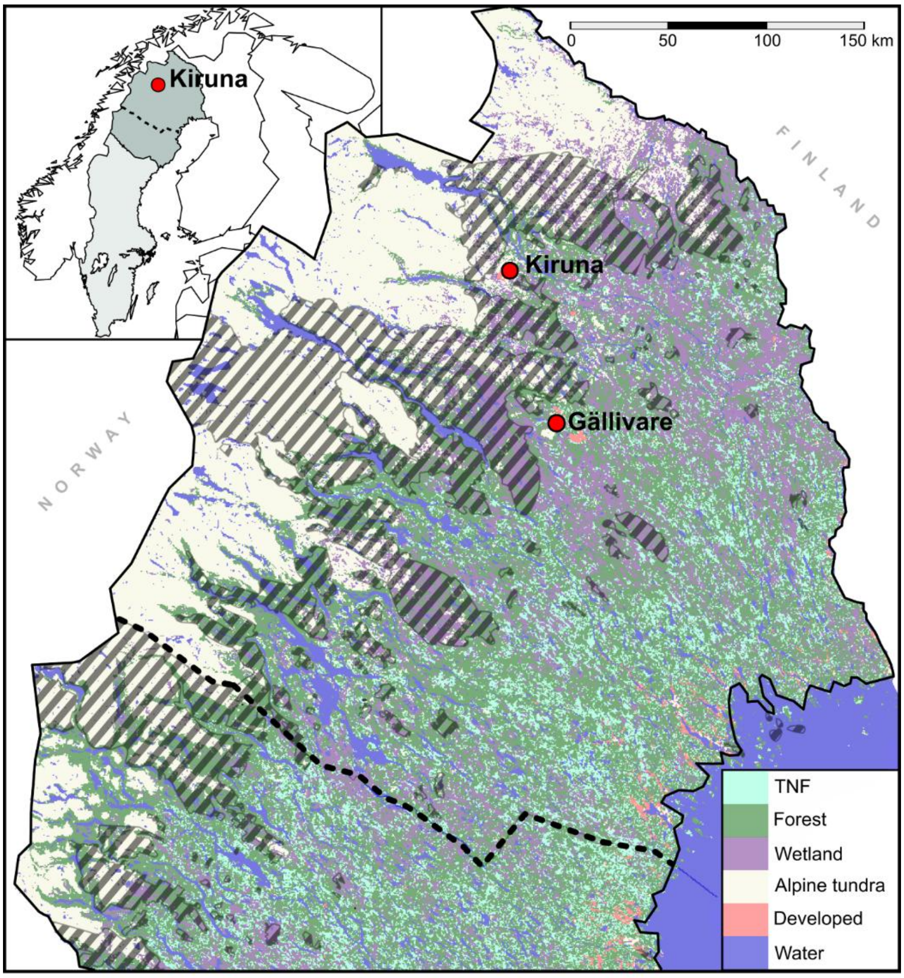

Supplementary Figure 24. Land cover from the Swedish National Land Cover Database (NMD) and formally protected areas (hatched lines) within 350 km of the aerosol station.

Land cover classes were aggregated from the original 21 thematic classes: 'TNF' are 'temporarily non-forested' with regrowing trees that are < 5 m tall; 'forests' designates areas with > 10% crown cover and > 5 m canopy height; 'wetlands' denotes non-forested areas where water covers the soil most of the year; 'alpine tundra' refers to non-wetland areas incapable of supporting forests but may be covered by vascular plants, bryophytes, or lichens; 'developed' includes permanent construction, roads, railways, and a small amount of cultivated land (< 1%); and 'water' includes all permanent water bodies. The inset map shows Sweden within Fennoscandia (light shading), highlighting Norrbotten and Västerbotten provinces (darker shading), the town of Kiruna, and the provincial border (dotted line).

[ö] ståndortsindex

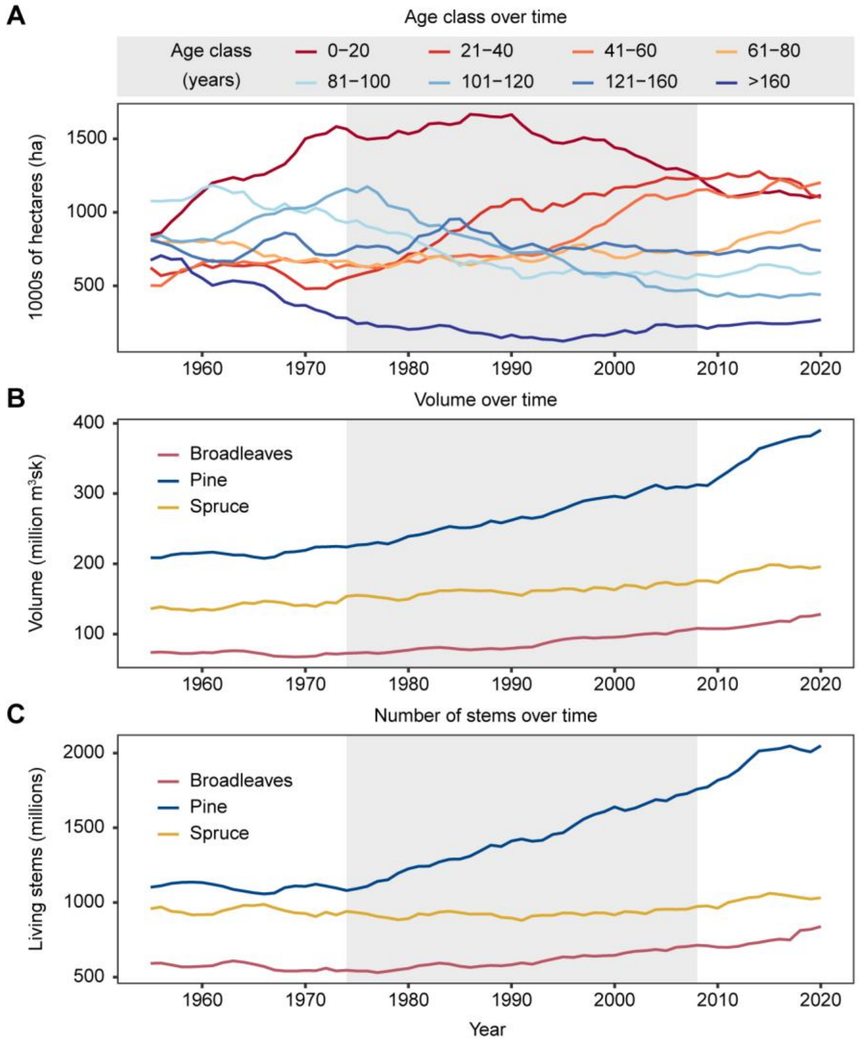

Supplementary Figure 25. Forest age and standing biomass in northern Sweden.
A) Productive forest area by age class, **B**) total standing volume in millions of forest cubic meters (m^3sk) by species across all land use classes. **C**) Total number of living stems (≥ 10 cm) in all land use classes.

Data from the NFI for northern Norrland[109] indicate the extent of > 100 year old forests declined by *ca*. 35% during the years concurrent with the eDNA time series (supplementary fig. 25A). Forests older than 160 years decreased by 55% between 1974 and 1995, or an 80% decline since 1955 (supplementary fig. 25A). The oldest forest fraction increased modestly after the mid-1990s minima (supplementary fig. 25A); these likely established from advance regeneration left by early high-grading[112] and some may be functionally ‘old-growth’ forests[113]. Forest biomass (in forest cubic meters,[aa] m^3sk) and density have generally increased since 1955, but pine has increased the most by far (supplementary fig. 25B and C).

Even-aged management strategies dominate modern Sweden forestry. Final fellings are typically made in 5-20 ha clearcuts, where trees are debranched and cut to length on site and then hauled to a roadside landing. Since the 1990s, logging residues (e.g., tops, branches, small trees) are commonly collected during or shortly after timber harvest[114], chipped at the roadside, and then sent to a forest biomass terminal[115]. Regeneration is ensured through soil

[aa] the solid over-bark volume from stump to the top of the bole; skogskubikmeter

scarification and, most commonly, planted seedlings[112] with a target density of ca. 1,800 - 2,500 pole-sized trees per hectare[116]. Pre-commercial thinning generally occurs within 10 years of establishment, followed by one or two rounds of commercial thinning[112] before final harvest. Legal minimum harvest ages are determined by species and site index but ranged from 120-130 years for northern Sweden under the 1979 Forestry Act[bb] and were reduced to 90-100 by the 1993 amendment.[cc]

Higher resolution forest history data, especially integrated in a spatiotemporal framework with eDNA data, could help identify how specific silvicultural treatments or conservation interventions impact (or not) regional biodiversity. Conversely, historic reconstructions informed by archaeological datasets (*e.g.*,[117]) would help verify and calibrate eDNA time series when contemporary remote sensing and monitoring data are lacking, as is the case here. For example, the peak in pine-associated eDNA we found during the mid-1990s coincides with a period of rapid change in the area covered by pine, which suggests aerosols emitted by harvest and afforestation activities may have also influenced this trend (supplementary fig. 26).

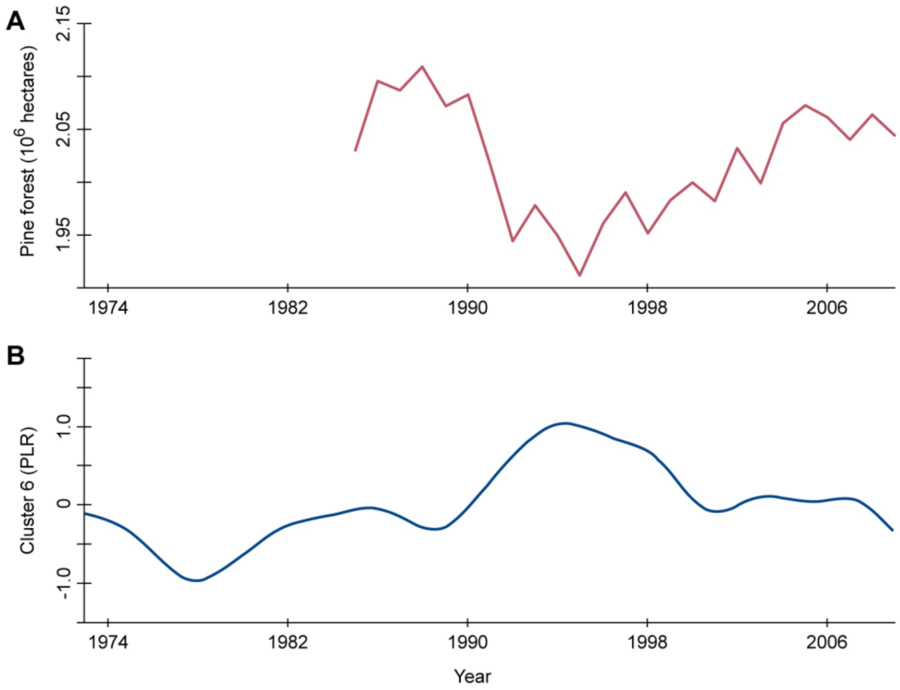

Supplementary Figure 26. Comparison of trends in pine forest cover and pine-associated eDNA abundance.

A) spatial extent of pine forests in Norrbotten province from 1985 to 2008 from the National Forest Inventory and **B**) weekly trend estimates of the PLR-transformed relative abundance of cluster 6, the pine-dominated cluster.

[bb] Skogsvårdslagen 1979:429
[cc] SFS (Swedish code of statutes) 1993:553 10§

References

1. Swedish National Land Cover Database. https://www.naturvardsverket.se/verktyg-och-tjanster/kartor-och-karttjanster/nationella-marktackedata.

2. Söderström, C., Ban, S., Jansson, P., Lindh, K. & Tooloutalaie, N. *Radionuclides in Ground Level Air in Sweden Year 2006*. FOI-R--2260--SE, Swedish Defence Research Agency (2007). https://www.foi.se/en/foi/reports/report-summary.html?reportNo=FOI-R--2260--SE

3. Karlsson, E. *et al.* Airborne microbial biodiversity and seasonality in Northern and Southern Sweden. *PeerJ* **8**, e8424 (2020).

4. Lindqvist, J. *En Stokastisk Partikelmodell i Ett Icke-Metriskt Koordinatsystem*. FOI-R–99-01086-862-SE, Swedish Defence Research Agency (1999).

5. Sato, Y. *et al.* Model Intercomparison of Atmospheric 137Cs From the Fukushima Daiichi Nuclear Power Plant Accident: Simulations Based on Identical Input Data. *Journal of Geophysical Research: Atmospheres* **123**, 11748–11765 (2018).

6. Grahn, H., von Schoenberg, P. & Brännström, N. What's that smell? Hydrogen sulphide transport from Bardarbunga to Scandinavia. *Journal of Volcanology and Geothermal Research* **303**, 187–192 (2015).

7. Björnham, O. *et al.* The 2016 Al-Mishraq sulphur plant fire: Source and health risk area estimation. *Atmos Environ* **169**, 287–296 (2017).

8. Ringbom, A. *et al.* Radioxenon Releases from A Nuclear Power Plant: Stack Data and Atmospheric Measurements. *Pure Appl Geophys* **178**, 2677–2693 (2021).

9. Muñoz-Sabater, J. ERA5-Land hourly data from 1950 to present. Copernicus Climate Change Service (C3S) Climate Data Store (CDS). DOI: 10.24381/cds.e2161bac (Accessed September 2019) (2019).

10. Hersbach, H. *et al.* ERA5 hourly data on single levels from 1940 to present. Copernicus Climate Change Service (C3S) Climate Data Store (CDS), DOI: 10.24381/cds.adbb2d47 (Accessed September 2019) (2018).

11. Efstathiou, C., Isukapalli, S. & Georgopoulos, P. A mechanistic modeling system for estimating large-scale emissions and transport of pollen and co-allergens. *Atmos Environ* **45**, 2260–2276 (2011).

12. Clauß, M. Particle size distribution of airborne micro-organisms in the environment-A review. *Landbauforschung Volkenrode* **65**, 77–100 (2015).

13. Helin, A. *et al.* Characterization of free amino acids, bacteria and fungi in size-segregated atmospheric aerosols in boreal forest: Seasonal patterns, abundances and size distributions. *Atmos Chem Phys* **17**, 13089–13101 (2017).

14. Ruiz-Jimenez, J. *et al.* Determination of free amino acids, saccharides, and selected microbes in biogenic atmospheric aerosols - Seasonal variations, particle size distribution, chemical and microbial relations. *Atmos Chem Phys* **21**, 8775–8790 (2021).

15. Canty, A. & Ripley, B. boot: Bootstrap Functions (Originally by Angelo Canty for S). R package at https://cran.r-project.org/package=boot (2022).

16. Davison, A. C. & Hinkley, D. V. *Bootstrap Methods and Their Application*. *Bootstrap Methods and their Application* (Cambridge University Press, Cambridge, 1997). doi:10.1017/cbo9780511802843.

17. Pinheiro, J. *et al.* nlme: Linear and Nonlinear Mixed Effects Models. R package at https://cran.r-project.org/web/packages/nlme/index.html (2023).

18. Pinheiro, J. & Bates, D. *Mixed-Effects Models in S and S-PLUS*. *Mixed-Effects Models in S and S-PLUS* (Springer New York, New York, 2000). doi:10.1007/b98882.

19. Hopke, P. K. Review of receptor modeling methods for source apportionment. *J Air Waste Manage Assoc* **66**, 237–259 (2016).

20. Belis, C. *et al. European Guide on Air Pollution Apportionment with receptor Models : Revised Version 2019*. EUR 29816 EN, Publications Office of the European Union, Luxembourg, JRC117306, (2019) doi:10.2760/439106.

21. Kalnay, E. *et al.* The NCEP/NCAR 40-year reanalysis project. *Bull Am Meteorol Soc* **77**, 437–472 (1996).

22. Zhou, C. *et al.* Ambient Ammonia Concentrations Across New York State. *Journal of Geophysical Research: Atmospheres* **124**, 8287–8302 (2019).

23. Zhou, L., Hopke, P. K. & Liu, W. Comparison of two trajectory based models for locating particle sources for two rural New York sites. *Atmos Environ* **38**, (2004).

24. Brook, J. R., Johnson, D. & Mamedov, A. Determination of the source areas contributing to regionally high warm season PM2.5 in eastern north america. *J Air Waste Manage Assoc* **54**, 1162–9 (2004).

25. Carslaw, D. C. & Ropkins, K. Openair - An r package for air quality data analysis. *Environmental Modelling and Software* **27–28**, 52–61 (2012).

26. De Cort, M. *et al. Atlas of Caesium Deposition on Europe after the Chernobyl Accident*. EUR 16733, European Commission - Directorate-General for Research and Innovation, Publications Office (1998).

27. Majlesi, S. Radioecology for boreal ecosystems : studies on transfer processes and effects on wildlife. (University of Eastern Finland, Kuopio, 2021).

28. Stohl, A., Hittenberger, M. & Wotawa, G. Validation of the Lagrangian particle dispersion model FLEXPART against large-scale tracer experiment data. *Atmos Environ* **32**, 4245–4264 (1998).

29. Gebhart, K. A., Schichtel, B. A. & Barna, M. G. Directional Biases in Back Trajectories Caused by Model and Input Data. *J Air Waste Manage Assoc* **55**, 1649–1662 (2005).

30. Métris, K. L. & Métris, J. Aircraft surveys for air eDNA: probing biodiversity in the sky. *PeerJ* **11**, e15171 (2023).

31. Watson, J. G., Chen, L. W. A., Chow, J. C., Doraiswamy, P. & Lowenthal, D. H. Source apportionment: Findings from the U.S. supersites program. *J Air Waste Manage Assoc* **58**, 265–288 (2008).

32. Johansen, T. *et al.* 'real-time' genetic monitoring of a commercial fishery on the doorstep of an MPA reveals unique insights into the interaction between coastal and migratory forms of the Atlantic cod. *ICES Journal of Marine Science* **75**, 1093–1104 (2018).

33. Strøm, J. F. *et al.* Movement diversity and partial sympatry of coastal and Northeast Arctic cod ecotypes at high latitudes. *Journal of Animal Ecology* **92**, 1966–1978 (2023).

34. Lavsund, S., Nygrén, T. & Solberg, E. J. Status of Moose Populations and Challenges to Moose Management in Fennoscandia. *Alces* **39**, 109–130 (2003).

35. Singh, N. J., Börger, L., Dettki, H., Bunnefeld, N. & Ericsson, G. From migration to nomadism: Movement variability in a northern ungulate across its latitudinal range. *Ecological Applications* **22**, 2007–2020 (2012).

36. Dabney, J. *et al.* Complete mitochondrial genome sequence of a Middle Pleistocene cave bear reconstructed from ultrashort DNA fragments. *Proc Natl Acad Sci U S A* **110**, 15758–15763 (2013).

37. Slon, V. *et al.* Neandertal and Denisovan DNA from Pleistocene sediments. *Science (1979)* **356**, 605–608 (2017).

38. Oksanen, J. *et al.* vegan: Community Ecology Package. R package at https://CRAN.R-project.org/package=vegan (2020).

39. Martin, M. Cutadapt removes adapter sequences from high-throughput sequencing reads. *EMBnet J* **17**, 10–12 (2011).

40. Bushnell, B. *BBMap Short Read Aligner*. *Joint Genome Instritute, department of energy* vol. **13** (2014).

41. Wood, D. E., Lu, J. & Langmead, B. Improved metagenomic analysis with Kraken 2. *Genome Biol* **20**, 1–13 (2019).

42. Camacho, C. *et al.* BLAST+: architecture and applications. *BMC Bioinformatics* **10**, 421 (2009).

43. SLU Artdatabanken 2020. Artportalen. https://artportalen.se/ (Downloaded 2020-02-20).

44. Aitchison, J. *The Statistical Analysis of Compositional Data*. *The Statistical Analysis of Compositional Data* (Chapman and Hall, London, 1986). doi:10.1007/978-94-009-4109-0.

45. Gloor, G. B., Macklaim, J. M., Pawlowsky-Glahn, V. & Egozcue, J. J. Microbiome datasets are compositional: And this is not optional. *Front Microbiol* **8**, 2224 (2017).

46. Filzmoser, P., Hron, K. & Templ, M. *Applied Compositional Data Analysis: With Worked Examples in R*. (Springer International Publishing, Cham, Switzerland, 2018).

47. Martín-Fernández, J. A., Hron, K., Templ, M., Filzmoser, P. & Palarea-Albaladejo, J. Bayesian-multiplicative treatment of count zeros in compositional data sets. *Stat Modelling* **15**, 134–158 (2015).

48. Palarea-Albaladejo, J. & Martín-Fernández, J. A. zCompositions — R package for multivariate imputation of left-censored data under a compositional approach. *Chemometrics and Intelligent Laboratory Systems* **143**, 85–96 (2015).

49. van den Boogaart, K. G. & Tolosana-Delgado, R. *Analyzing Compositional Data with R*. *Analyzing Compositional Data with R* (Springer-Verlag Berlin and Heidelberg GmbH Co. K, Heidelberg, 2013). doi:10.1007/978-3-642-36809-7.

50. Filzmoser, P., Hron, K. & Reimann, C. Univariate statistical analysis of environmental (compositional) data: Problems and possibilities. *Science of the Total Environment* **407**, 6100–6108 (2009).

51. Egozcue, J. J., Daunis-I-Estadella, J., Pawlowsky-Glahn, V., Hron, K. & Filzmoser, P. Simplicial regression. The normal model. *J Appl Probab Stat* **6**, 87–108 (2012).

52. Templ, M., Hron, K. & Filzmoser, P. *RobCompositions: An R-Package for Robust Statistical of Compositional Data*. *Compositional Data Analysis: Theory and Applications* (John Wiley and Sons, 2011). doi:10.1002/9781119976462.ch25.

53. van den Boogaart, K. G. & Tolosana-Delgado, R. 'compositions': A unified R package to analyze compositional data. *Comput Geosci* **34**, 320–338 (2008).

54. Seabold, S. & Perktold, J. Statsmodels: Econometric and Statistical Modeling with Python. in *Proceedings of the 9th Python in Science Conference* 92–96 (2010). doi:10.25080/majora-92bf1922-011.

55. Akaike, H. Information Theory and an Extension of the Maximum Likelihood Principle. in *Selected Papers of Hirotugu Akaike* (eds. Parzen, E., Tanabe, K. & Kitagawa, G.) 199–213 (Springer New York, New York, NY, 1998). doi:10.1007/978-1-4612-1694-0_15.

56. Ye, S. H., Siddle, K. J., Park, D. J. & Sabeti, P. C. Benchmarking Metagenomics Tools for Taxonomic Classification. *Cell* **178**, 779–794 (2019).

57. GBIF.org (15 May 2020) GBIF Occurrence Download https://doi.org/10.15468/dl.cjxesu.

58. GBIF.org (28 May 2020) GBIF Occurrence Download https://doi.org/10.15468/dl.xnyctg.

59. Chen, T. *et al.* xgboost: Extreme Gradient Boosting. R package at https://CRAN.R-project.org/package=xgboost (2021).

60. Quinlan, A. R. & Hall, I. M. BEDTools: A flexible suite of utilities for comparing genomic features. *Bioinformatics* **26**, 841–842 (2010).

61. Danecek, P. *et al.* Twelve years of SAMtools and BCFtools. *Gigascience* **10**, giab008 (2021).

62. Quinn, T. P., Richardson, M. F., Lovell, D. & Crowley, T. M. Propr: An R-package for Identifying Proportionally Abundant Features Using Compositional Data Analysis. *Sci Rep* **7**, 16252 (2017).

63. Lovell, D., Pawlowsky-Glahn, V., Egozcue, J. J., Marguerat, S. & Bähler, J. Proportionality: A Valid Alternative to Correlation for Relative Data. *PLoS Comput Biol* **11**, e1004075 (2015).

64. Rousseeuw, P. J. Silhouettes: A graphical aid to the interpretation and validation of cluster analysis. *J Comput Appl Math* **20**, 53–65 (1987).

65. Caliñski, T. & Harabasz, J. A Dendrite Method For Cluster Analysis. *Communications in Statistics* **3**, 1–27 (1974).

66. Hill, M. O. Diversity and Evenness: A Unifying Notation and Its Consequences. *Ecology* **54**, 427–432 (1973).

67. Jost, L. Entropy and diversity. *Oikos* **113**, 363–375 (2006).

68. Reeve, R. *et al.* How to partition diversity. Preprint at https://doi.org/10.48550/arXiv.1404.6520 (2016).

69. Leinster, T. *Entropy and Diversity: The Axiomatic Approach*. (Cambridge University Press, Cambridge, 2021).

70. Menne, M. J., Durre, I., Vose, R. S., Gleason, B. E. & Houston, T. G. An overview of the global historical climatology network-daily database. *J Atmos Ocean Technol* **29**, 897–910 (2012).

71. PTHBV - en areellt högupplöst klimatdatabas för hydrologiska modellberäkningar. https://www.smhi.se/kunskapsbanken/hydrologi/pthbv-en-areellt-hogupplost-klimatdatabas-for-hydrologiska-modellberakningar-1.190268.

72. Zhang, X. *et al.* Indices for monitoring changes in extremes based on daily temperature and precipitation data. *Wiley Interdiscip Rev Clim Change* **2**, 851–870 (2011).

73. Zhang, X., Hegerl, G., Zwiers, F. W. & Kenyon, J. Avoiding inhomogeneity in percentile-based indices of temperature extremes. *J Clim* **18**, 1641–1651 (2005).

74. Abatzoglou, J. T., Dobrowski, S. Z., Parks, S. A. & Hegewisch, K. C. TerraClimate, a high-resolution global dataset of monthly climate and climatic water balance from 1958-2015. *Sci Data* **5**, 170191 (2018).

75. Hurrell, J. W. NAO Index Data provided by the Climate Analysis Section, NCAR, Boulder, USA, Hurrell (2003). Updated regularly. Accessed 31 December 2022.

76. Trenberth, K. E. & Shea, D. J. AMO Index Data provided by the Climate Analysis Section, NCAR, Boulder, USA, Trenberth and Shea (2006). Updated yearly. Accessed 31 December 2022.

77. Climate Prediction Center, NOAA. Accessed 31 December 2022. https://www.cpc.ncep.noaa.gov/products/precip/CWlink/daily_ao_index/ao.shtml.

78. Weiskopf, S. R. *et al.* Climate change effects on biodiversity, ecosystems, ecosystem services, and natural resource management in the United States. *Science of the Total Environment* **733**, 137782 (2020).

79. Narayan, A., Berger, B. & Cho, H. Assessing single-cell transcriptomic variability through density-preserving data visualization. *Nat Biotechnol* **39**, 765–774 (2021).

80. Campello, R. J. G. B., Moulavi, D. & Sander, J. Density-Based Clustering Based on Hierarchical Density Estimates. in *Advances in Knowledge Discovery and Data Mining. PAKDD 2013. Lecture Notes in Computer Science()* (eds. Pei, J., Tseng, V. S., Cao, L., Motoda, H. & Xu, G.) vol. **7819** 160–172 (Springer Berlin Heidelberg, Berlin, Heidelberg, 2013).

81. Hahsler, M., Piekenbrock, M. & Doran, D. dbscan: Fast Density-Based Clustering with R. *J Stat Softw* **91**, 1–30 (2019).

82. Kalman, R. E. A new approach to linear filtering and prediction problems. *Transactions of the ASME--Journal of Basic Engineering* **82**, 35–45 (1960).

83. Durbin, J. & Koopman, S. J. *Time Series Analysis by State Space Methods*. *Time Series Analysis by State Space Methods* (Oxford University Press, Oxford, 2012). doi: https://doi.org/10.1093/acprof:oso/9780199641178.001.0001.

84. Scott, S. L. & Varian, H. R. Predicting the present with Bayesian structural time series. *International Journal of Mathematical Modelling and Numerical Optimisation* **5**, 4–23 (2014).

85. Scott, S. L. bsts: Bayesian Structural Time Series. R package at https://CRAN.R-project.org/package=bsts (2022).

86. Young, P. C., Ng, C. N., Lane, K. & Parker, D. Recursive forecasting, smoothing and seasonal adjustment of non-stationary environmental data. *J Forecast* **10**, 57–89 (1991).

87. Merow, C. *et al.* What do we gain from simplicity versus complexity in species distribution models? *Ecography* **37**, 1267–1281 (2014).

88. Commandeur, J. J. F. & Koopman, S. J. *An Introduction to State Space Time Series Analysis*. (Oxford University Press, Incorporated, 2007).

89. Geweke, J. Evaluating the Accuracy of Sampling-Based Approaches to the Calculation of Posterior Moments. in *Bayesian Statistics* (eds. Bernardo, J. M., Berger, O., Dawid, A. P. & Smith, A. F. M.) vol. **4** 169–193 (Clarendon Press, Oxford, 1992).

90. Raftery, A. E. & Lewis, S. M. Comment: One long run with diagnostics: Implementation strategies for markov chain monte carlo. *Statistical Science* **7**, 493–497 (1992).

91. Plummer, M., Best, N., Cowles, K. & Vines, K. CODA: Convergence Diagnosis and Output Analysis for MCMC. *R News* **6**, 7–11 (2006).

92. Vehtari, A., Gelman, A. & Gabry, J. Practical Bayesian model evaluation using leave-one-out cross-validation and WAIC. *Stat Comput* **27**, 1413–1432 (2017).

93. Bürkner, P. C., Gabry, J. & Vehtari, A. Approximate leave-future-out cross-validation for Bayesian time series models. *J Stat Comput Simul* **90**, 2499–2523 (2020).

94. Sivula, T., Magnusson, M., Matamoros, A. A. & Vehtari, A. Uncertainty in Bayesian Leave-One-Out Cross-Validation Based Model Comparison. Preprint at https://doi.org/10.48550/arXiv.2008.10296 (2022).

95. GBIF.org (29 March 2021) GBIF Occurrence Download https://doi.org/10.15468/dl.k76kgd.

96. Holmes, E. E., Ward, E. J. & Wills, K. MARSS: Multivariate autoregressive state-space models for analyzing time-series data. *R Journal* **4**, 11–19 (2012).

97. Holmes, E. E., Scheuerell, M. D. & Ward, E. J. Detecting a signal from noisy sensors. in *Applied time series analysis for fisheries and environmental data. Edition 2021.*

98. Dahlén, J., Green, M. & Lindström, Å. *Fåglar i Norrbottens Län – Förekomst Och Populations-Utveckling 1998-2007.* Länsstyrelsens rapportserie nr 1/2009, Länsstyrelsen Norrbotten, Luleå (2009).

99. Brännlund, I. & Axelsson, P. Reindeer management during the colonization of Sami lands: A long-term perspective of vulnerability and adaptation strategies. *Global Environmental Change* **21**, 1095–1105 (2011).

100. Östlund, L. & Norstedt, G. Preservation of the cultural legacy of the indigenous Sami in northern forest reserves – Present shortcomings and future possibilities. *For Ecol Manage* **502**, 119726 (2021).

101. Avango, D. Imprints on the Resource Landscape: The Long History of Mining in the Arctic. *Journal of Northern Studies* **14**, 67–82 (2020).

102. Översiktlig skogsinventering (ÖSI). https://geodpags.skogsstyrelsen.se/geodataport/feeds/Osi.xml.

103. Kuznetsov, N. *Доклад о Состоянии и Об Охране Окружающей Среды Мурманской Области в 2013 Году [Report on the State and Protection of the Environment of the Murmansk Region in 2013]*. https://mpr.gov-murman.ru/upload/iblock/da1/2013.pdf (2014).

104. Pape, R. & Löffler, J. Climate change, land use conflicts, predation and ecological degradation as challenges for reindeer husbandry in northern europe: What do we really know after half a century of research? *Ambio* **41**, 421–434 (2012).

105. Käyhkö, J. & Horstkotte, T. *Reindeer Husbandry under Global Change in the Tundra Region of Northern Fennoscandia*. (2017) doi:10.13140/RG.2.2.22151.39841.

106. Stark, S. *et al.* The ecosystem effects of reindeer (Rangifer tarandus) in northern Fennoscandia: Past, present and future. *Perspect Plant Ecol Evol Syst* **58**, 125716 (2023).

107. Tyler, N. J. C., Hanssen-Bauer, I., Førland, E. J. & Nellemann, C. The Shrinking Resource Base of Pastoralism: Saami Reindeer Husbandry in a Climate of Change. *Front Sustain Food Syst* **4**, 585685 (2021).

108. Fridman, J. *et al.* Adapting National Forest Inventories to changing requirements - The case of the Swedish National Forest Inventory at the turn of the 20th century. *Silva Fennica* **48**, 1095 (2014).

109. The Swedish National Forest Inventory. https://www.slu.se/riksskogstaxeringen.

110. Wulder, M. A., Hermosilla, T., White, J. C. & Coops, N. C. Biomass status and dynamics over Canada's forests: Disentangling disturbed area from associated aboveground biomass consequences. *Environmental Research Letters* **15**, 094093 (2020).

111. Swedish Forest Agency. *Swedish Statistical Yearbook of Forestry 2014*. (Official Statistics of Sweden, Swedish Forest Agency, Sweden, 2014).

112. Roberge, J.-M. *et al. Forest Management in Sweden Current Practice and Historical Background*. RAPPORT 2020/4, Swedish Forest Agency (2020).

113. *Skogsdata 2023*. Dept. of Forest Resource Management, Swedish University of Agricultural Sciences, Umeå (2023).

114. Nilsson, L. J. *et al.* Seeing the wood for the trees: 25 years of renewable energy policy in Sweden. *Energy for Sustainable Development* **8**, 67–81 (2004).

115. Routa, J., Asikainen, A., Björheden, R., Laitila, J. & Röser, D. Forest energy procurement: State of the art in Finland and Sweden. *Wiley Interdiscip Rev Energy Environ* **2**, 602–613 (2013).

116. Nilsson, U., Luoranen, J., Kolström, T., Örlander, G. & Puttonen, P. Reforestation with planting in northern Europe. *Scand J For Res* **25**, 283–294 (2010).

117. Ostlund, L., Zackrisson, O. & Axelsson, A. L. The history and transformation of a Scandinavian boreal forest landscape since the 19th century. *Canadian Journal of Forest Research* **27**, 1198–1206 (1997).
