# Supplementary material for: Airborne eDNA captures three decades of ecosystem biodiversity
Source: Nat Commun. 2025 Dec 18;16:11281. doi: 10.1038/s41467-025-67676-7 (PMC12717267; doi:10.1038/s41467-025-67676-7)
Supplement: Supplementary file 2 — Descriptions of Additional Supplementary Files [file 41467_2025_67676_MOESM2_ESM.pdf]

Descriptions of Additional Supplementary Files

Supplementary Data 1 - Particle mass originating from different distances from the aerosol sampling station for each week (sheet 1) and yearly averages as well as proportion of particle mass originating from all cardinal directions (sheet 2).

Supplementary Data 2 - The total amount in ng of DNA extracted from each air filter.

Supplementary Data 3 - Taxonomic composition of the Kraken 2 database and the total sequence (in basepairs) used as input.

Supplementary Data 4 - List of observed genera in Torne lappmark according to the Swedish Species Observation System.

Supplementary Data 5 - Labelled genera used to train the gradient boosting classifier. “tax_id” denotes the NCBI taxonomic identification code assigned to the reads by Kraken 2 and “genus” is the corresponding name; “type” indicates if a genus was considered as a true or false positive; “set” identifies those used in model training or reserved for model testing; and columns 5-419 contain feature data and are described in the Supplementary Materials.

Supplementary Data 6 - Relative abundances of the 2,739 positively classified genera for each sampling week (n = 380). Columns include the NCBI taxonomy identifier (tax_id), full taxonomic hierarchy (domain to genus), predicted probability of being a true positive (pp), and cluster assignment (cluster). Time stamps are given as YEAR_WEEK, where WEEK corresponds to the ISO calendar week number.

Supplementary Data 7 - Validation of 65 gradient-boosting classifications using read alignment toreference assemblies and sequence similarity searches with BLAST.

Supplementary Data 8 - Summary of the taxonomic composition of the 17 clusters identified through hierarchical clustering of pairwise covariance in logratios. Taxonomic ranks from domain through genus that comprise ≥ 5% of a given cluster are enumerated, along with their mean relative abundance. Taxonomy follows the NCBI taxonomic database.

Supplementary Data 9 - Median differences and two-sided 95% nonparametric confidence intervals from Wilcoxon signed-rank tests for per-genus differences in γ-diversity contributions between 1974–1988 and 1994–2008. Negative values indicate larger contributions in 1994–2008. P-values were adjusted for multiple comparisons using the Benjamini– Hochberg procedure (5% FDR). Cluster membership and NCBI taxonomy are provided for convenience.

Supplementary Data 10 - Climatic regressor matrix used in time series models. Variable abbreviations correspond to table s4.

Supplementary Data 11 - Summary of Bayesian state space model fit and convergence diagnostics for production runs. Models are grouped by sheet, where ‘abundances’ refers to cluster abundance models using the full time series data, ‘catchment’ refers to abundances truncated to match the time period of the particle dispersion models, ‘diversity’ contains α-, β-, γ- diversity of order q = 1, 2, and 3 for each of the the ‘total’, ‘no14’ and ‘eukaryotic’ fractions of the eDNA community; and ‘birds’ contains the summary results for eight genera with contemporaneous survey data. The trend and regressor matrix specification comprising the model are indicated, and the expected log pointwise predictive densities (ELPD), its standard error (ELPD.SE), along with model prediction and residual standard deviations and r2 . Residual diagnostics include the maximum residual autocorrelation (acf.max) and its lag (acf.max.lag), the F variance ratio, and Kolomogorov-Smirnov’s d (KS.d). Effective sample sizes (ESS), the Geweke statistic, and Raftery and Lewis's diagnostic (RL) are given for each parameter. The ELPD difference (ELPD.diff) and the standard error of this difference (ELPD.diff.se) is reported between a given model and the highest-scoring model in a comparison.

Supplementary Data 12 - Supplemental time series model results for eDNA temporal cluster abundances and community diversity metrics.

Supplementary Data 13 - Marginal inclusion probabilities and median coefficient estimate with 95% credible intervals for each regressor. Results are shown for climatic regression models that were supported over alternative specifications by differences in expected log pointwise predictive densities (ELPD).
