# Supplementary material for: Airborne eDNA captures three decades of ecosystem biodiversity
Source: Nat Commun. 2025 Dec 18;16:11281. doi: 10.1038/s41467-025-67676-7 (PMC12717267; doi:10.1038/s41467-025-67676-7)
Supplement: Supplementary file 9 — Supplementary Data 7 [file 41467_2025_67676_MOESM9_ESM.pdf]

Supplementary Data 7

Validation of 65 gradient-boosting classifications using read alignment to reference assemblies and sequence similarity searches with BLAST.

Contents

Contents 1

Summary..4

True negatives..7

Ceratotherium; Chordata; Mammalia; Perissodactyla; Rhinocerotidae (9806) 7

Eucalyptus; Streptophyta; Magnoliopsida; Myrtales; Myrtaceae (3932) 9

Hydrophis; Chordata; Squamata; Hydrophiidae (8683) 11

Liriodendron; Streptophyta; Magnoliopsida; Magnoliales; Magnoliaceae (3413) 12

Ornithorhynchus; Chordata; Mammalia; Monotremata; Ornithorhynchidae (9257) 14

Timema; Arthropoda; Insecta; Phasmatodea; Timematidae (61471) 16

Tragelaphus; Chordata; Mammalia; Artiodactyla; Bovidae (9944)..17

True positives..19

Birds 19

Anas; Chordata; Aves Anseriformes; Anatidae (8835) 19

Corvus; Chordata; Aves; Passeriformes; Corvidae (30420)..20

Cuculus; Chordata; Aves; Cuculiformes; Cuculidae (33592)...22

Ficedula; Chordata; Aves; Passeriformes; Muscicapidae (36292)..24

Lagopus; Chordata; Aves; Galliformes; Phasianidae (30409)..25

Parus; Chordata; Aves; Passeriformes; Paridae (9154)...26

Phylloscopus; Chordata; Aves; Passeriformes; Phylloscopidae (9181) 27

Saxicola; Chordata; Aves; Passeriformes; Muscicapidae (69509)..28

Other vertebrates 30

Alces; Chordata; Mammalia; Artiodactyla; Cervidae (9851) 30

Gadus; Chordata; Actinopteri; Gadiformes; Gadidae (8048) 32

Rana; Chordata; Amphibia; Anura; Ranidae (8399) 35

Rangifer; Chordata; Mammalia; Artiodactyla; Cervidae (9869) 36

Salmo; Metazoa; Chordata; Actinopteri; Salmoniformes; Salmonidae (8028) 37

Thymallus; Chordata; Actinopteri; Salmoniformes; Salmonidae (36184) 38

Insects..40

Aedes; Arthropoda; Insecta; Diptera; Culicidae (7158)..40

Belgica; Arthropoda; Insecta; Diptera; Chironomidae (315555)..41

Limnephilus; Arthropoda; Insecta; Trichoptera; Limnephilidae (177674) 43

Contarinia; Arthropoda; Insecta; Diptera; Cecidomyiidae (153220) 44
Mayetiola; Arthropoda; Insecta; Diptera; Cecidomyiidae (39757) 46
Operophtera; Arthropoda; Insecta; Lepidoptera; Geometridae (104451) 47
Pieris; Arthropoda; Insecta; Lepidoptera; Pieridae (7115) 49
Fungi 50
Cladonia; Ascomycota; Lecanoromycetes; Lecanorales; Cladoniaceae (5199) 50
Cortinarius; Basidiomycota; Agaricomycetes; Agaricales; Cortinariaceae (34451) 51
Evernia; Ascomycota; Lecanoromycetes; Lecanorales; Parmeliaceae (87256) 52
Fibularhizoctonia (syn. *Athelia*); Basidiomycota; Agaricomycetes; Atheliales; Atheliaceae (56747) 53
Fomitopsis; Basidiomycota; Agaricomycetes; Polyporales; Fomitopsidaceae (34474) 55
Lachnellula; Ascomycota; Leotiomycetes; Helotiales; Lachnaceae (47830) 57
Lactarius; Basidiomycota; Agaricomycetes; Russulales; Russulaceae (34444) 58
Melampsora; Basidiomycota; Pucciniomycetes; Pucciniales; Melampsoraceae (5260) 59
Paxillus; Basidiomycota; Agaricomycetes; Boletales; Paxillaceae (5395) 61
Porodaedalea; Basidiomycota; Agaricomycetes; Hymenochaetales; Hymenochaetaceae (175857) 63
Pseudevernia; Ascomycota; Lecanoromycetes; Lecanorales; Parmeliaceae (88743) 64
Suillus; Basidiomycota; Agaricomycetes; Boletales; Suillaceae (5379) 65
Russula; Basidiomycota; Agaricomycetes; Russulales; Russulaceae (5402) 67
Trametes; Basidiomycota; Agaricomycetes; Polyporales; Polyporaceae (5324) 68
Tricholoma; Basidiomycota; Agaricomycetes; Agaricales; Tricholomataceae (40144) 69
Plants 71
Alnus; Streptophyta; Magnoliopsida; Fagales; Betulaceae (3515) 71
Betula; Streptophyta; Magnoliopsida; Fagales; Betulaceae (3504) 72
Dactylis; Streptophyta; Magnoliopsida; Poales; Poaceae (4508) 73
Dryopteris; Streptophyta; Polypodiopsida; Polypodiales; Dryopteridaceae (3287) 74
Equisetum; Streptophyta; Polypodiopsida; Equisetales; Equisetaceae (3257) 75
Hordeum; Streptophyta; Magnoliopsida; Poales; Poaceae (4512) 77
Mielichhoferia; Streptophyta; Bryopsida; Bryales; Mniaceae (67233) 79
Pleurozium; Streptophyta; Bryopsida; Hypnales; Hylocomiaceae (34162) 80
Picea; Streptophyta; Pinopsida; Pinales; Pinaceae (3328) 82
Pinus; Streptophyta; Pinopsida; Pinales; Pinaceae (3337) 84
Physcomitrella; Streptophyta; Bryopsida; Funariales; Funariaceae (3217) 85

Populus; Streptophyta; Magnoliopsida; Malpighiales; Salicaceae (3689) 86
Quercus; Streptophyta; Magnoliopsida; Fagales; Fagaceae (3511) 87
Sphagnum; Streptophyta; Sphagnopsida; Sphagnales; Sphagnaceae (13804) 89
Triticum; Streptophyta; Magnoliopsida; Poales; Poaceae (4564) 90
Vaccinium; Streptophyta; Magnoliopsida; Ericales; Ericaceae (13749) 92
False positives 94
Chironomus; Arthropoda; Insecta; Diptera; Chironomidae (7150) 94
Brugia; Nematoda; Chromadorea; Rhabditida; Onchocercidae (6278) 96
Gavia;Chordata;Aves;Gaviiformes;Gaviidae (37038) 97
Larix; Streptophyta; Pinopsida; Pinales; Pinaceae (3325) 99
Termitomyces; Basidiomycota; Agaricomycetes; Agaricales; Lyophyllaceae (71927) 101
Mentha; Streptophyta; Magnoliopsida; Lamiales; Lamiaceae (21819) 103
References 105

Summary

Methods

We developed a classification validation framework from two predictions for a correctly-classified genus: 1) reads should originate from the entire genome, resulting in a positive correlation between contig length and read depth, and 2) sequences should be similar to closely-related organisms, evident in the taxonomic distribution of BLAST best-scoring sequence pairs (BSPs). In summary, we mapped Kraken 2 classified reads to their respective sequences in the reference database. Then, we generated consensus sequences for the 100 regions with the highest read depth and used BLASTN to compare these to the nt database. If BLASTN found BSPs with unrelated organisms, we generated consensus sequences for the next 100 regions by depth and repeated this process until no new potential contaminants were found. We also queried classified reads directly for a subset of genera (n = 26) and these results are presented where available, but we found consensus sequences enabled more powerful detection of contamination (see *Quercus*, *Hordeum* and *Triticum*) so we applied this method to all genera.

Read mapping and BLAST results

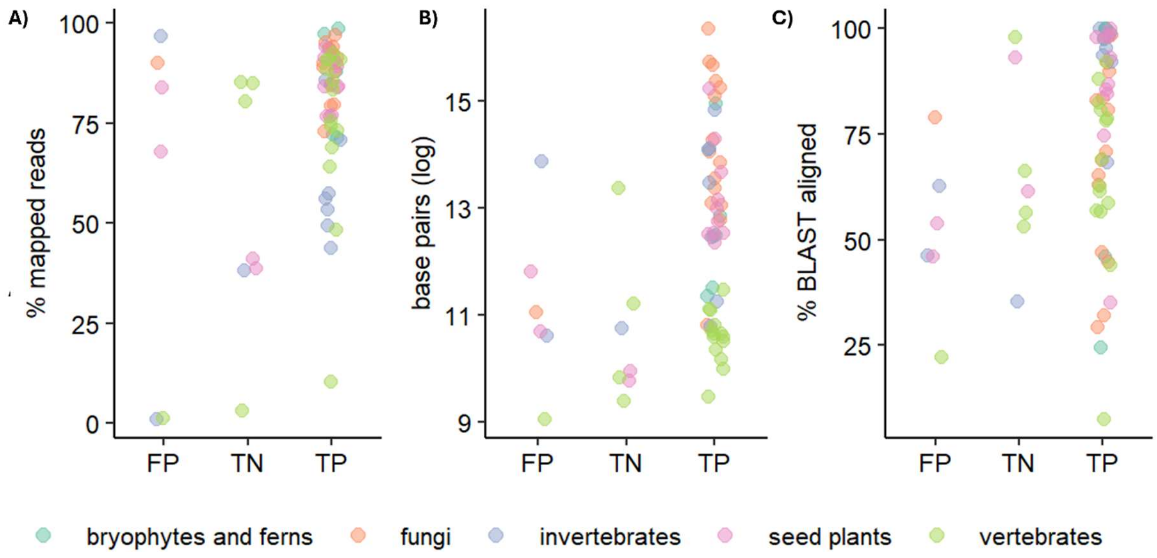

Figure 1. Mapping rates (A), the amount of consensus sequences compared to the BLAST nt database (B), and the proportion of consensus queries with at least one high-scoring sequence pair (C). FP are false positives (n = 6), genera with a positive binary classification from the gradient boosting machine (GBM) classifier but failed read-level validation. TN are true negatives (n = 7), those with a negative GBM classification and lacked any evidence of correctly-classified reads based on BLASTn alignments. TP are true positives (n = 52) were classified as positives by the GBM and were supported by multiple lines of evidence.

Read mapping rates were generally high (mean = 72%, SD = 24%), even with the relatively stringent mapping parameters we employed (Fig. 1A), which supports our finding that BLAST searches of consensus queries and individual reads produce similar error estimates overall. Read-level results are also provided for genera with low mapping rates. On average, we generated 1 Mb (SD = 2 Mb, range = 8,697 to 12,810,516 bp) of consensus sequences per genus to compare against the BLAST nt database (Fig. 1B), with more search effort given to putative true positive genera to confirm their classifications. Consensus sequences originated from 105 contigs on average, although this number was highly variable due to differences in reference (SD = 117),

and we found significant BLAST alignments (%ID >80%, length > 150bp, e-value < 1e-25) for an average of 72% of queried contigs (SD = 24%).

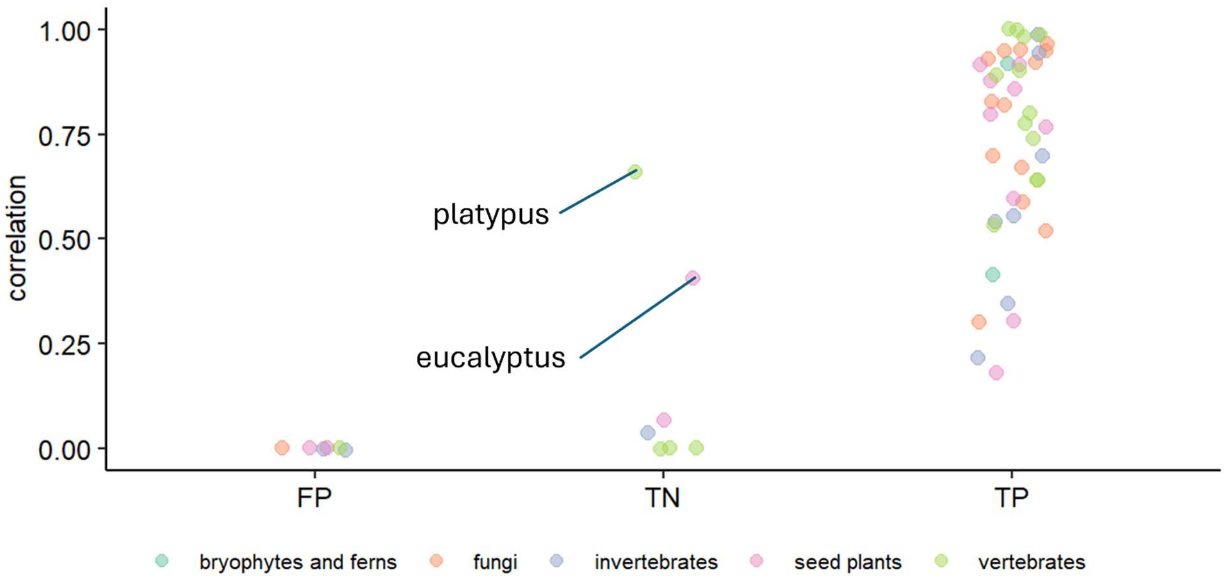

Figure 2. Correlation coefficient (*r*) between contig length and reads mapped for genera with non-organelle reference sequences > 50 kb. FP are false positives (n = 6), genera with a positive binary classification from the gradient boosting machine (GBM) classifier but failed read-level validation. TN are true negatives (n = 7), those with a negative GBM classification and lacked any evidence of correctly-classified reads based on BLASTn alignments. TP are true positives (n = 52) were classified as positives by the GBM and were supported by multiple lines of evidence.

Correlations between contig length and reads mapped were more complex in practice due to differences among genera in the number, quality, and diversity of reference database sequences. Nevertheless, we did find a more positive correlation for true positive (median $r = 0.80$; SD = 0.23) than negative (median $r = 0.04$; SD = 0.26) genera (Fig. X). In particular, contig length and mapped reads were strongly correlated among positive vertebrates ($r = 0.85$; SD = 0.16) and fungi ($r = 0.95$; SD = 0.28).

Like the mapping statistics, the taxonomic composition of the nt database and the generally low confidence in taxonomic delimitations and/or phylogenetic relationships at even higher ranks for some organisms (e.g. Fungi) introduced complexity to the interpretation of the BLAST high-scoring alignment pairs. We excluded self-matches and matches to the same assembly, which excluded almost all possible matches within the same genus or higher ranks (e.g. *Pleurozium*, *Evernia*). Where relevant to the BLAST results, we also present summaries of available sequences and a review of recent literature on recent taxonomic revisions and phylogenetic uncertainties.

Despite these complexities, most read misclassifications could be detected from BLAST alignments with other taxonomic families, orders, or classes (Table 1). Read misclassifications were largely explained by reference contamination. In particular, we detected contigs from moulds (*Cladosporium*, *Aspergillus*), trees with high pollen production (Betulaceae, *Picea*, *Pinus*), and humans in several different reference assemblies, which is consistent with contamination from the ambient environments. We also found contaminants that likely originated

from the holobiome of the target organism, including fungal gnats in mushrooms and endosymbiotic bacteria in insects.

Table 1. Median percentage of reads aligning to the same family, class, and order for false positive, true negative, and true positive genera. Standard deviations are given in parentheses.

decision	% same family	% same order	% same class
false positive	0 (39)	0 (39)	17.5 (45)
true negative	0 (13)	0 (34)	6 (46)
true positive	93 (29)	98 (14)	99 (4)

Read misclassifications from cross-contamination could be more difficult to detect, especially if multiple assemblies from a taxonomic group were produced by the same research program. We dealt with this challenge in two ways. First, we analyzed the five best-scoring sequence pairs per query to improve the chance of finding independent alignments. Secondly, we manually investigated if contigs with unusually high read depth had BLAST alignments with sequences released by different institutions (e.g., *Saxicola*, *Evernia*, *Porodaedalea*). These details are also presented in Supplementary Materials 7, but we did not find any cases where cross-contamination confounded the BLAST results.

Post-validation classifications

We identified six false positives: two genera that were included as negative training data but were nevertheless classified positively by the GBM (*Larix* and *Termitomyces*), two (out of 16; *Brugia* and *Mentha*) flagged for validation due to their lack of occurrence reports near the monitoring station, and two that were including as positive training data (*Chironomus* and *Gavia*). This gives a 10% false discovery rate (6/58; FDR), compared to the 7% estimated from the out-of-sample GBM training data.

Read misclassification rates for the 59 genera passing validation were generally low, ranging from undetectable to a maximum of 30%. Estimated read misclassification rates were ≥ 10% for three genera: *Cuculus*, *Hordeum*, and *Querus*.

We found limited evidence of label noise in the GBM training data. Two out of the 48 positive training genera were false positive (*Chironomus* and *Gavia*), although these two cases had a minority of correctly-classified reads. We did not attempt estimate label errors in the negative training data, although we found no evidence to suggest any of the nine negative training genera were mislabeled. We expect label noise in negative training data to be low overall; a rhinoceros in northern Sweden is unlikely to be undetected for long. Machine learning methods like the xgboost we used here are robust to moderate label noise up to *ca*. 20% (Northcutt et al. 2019).

Taken together, the validation results confirm the accuracy of all specific genera critical to the main results presented here. While these cannot scale beyond a few dozen genera, the accuracy of the training data and the high precision of the GBM classifications supports the validity of the taxonomic assignments for the entire dataset.

True negatives

Ceratotherium; Chordata; Mammalia; Perissodactyla; Rhinocerotidae (9806)

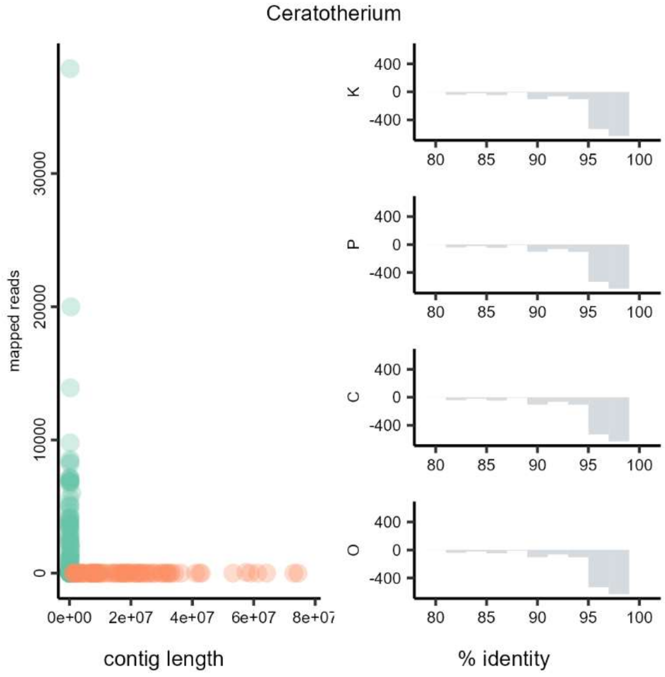

Figure 3. Left: number of mapped reads *vs*. contig length for contigs > 50 kb. Right: mirrored histograms of the sequence identity of aligned reads (ARs) with BLAST best-scoring sequence pairs (BSPs) within (top) or outside (bottom) the kingdom (K), phylum (P), class (C), and order (O) ranks of *Ceratotherium*. BSPs are shown for the 100 consensus sequences with the highest depth of coverage. ARs are the number of reads covering a given consensus sequence query and are given as *n*-fold differences.

Label: negative
Predicted probability: 0.10
Mapped reads: 349,064
Mapping rate: 89%
Base pairs queried: 647,977
Regions queried: 52
Regions aligned: 51

Ceratotherium, the white rhinoceros, was included in the gradient boosting machine (GBM) training dataset as a negative genus. Nuclear genome assemblies for *C. simum cottoni* (2.6 Gb, N50: 22 kb) and a more complete assembly for *C. simum simum* (1.7 Gb, N50: 29.9 Mb) were included in the Kraken 2 reference database.

89% of the *Ceratotherium*-classified reads mapped back to their sequences in the reference database. These reads mapped to 642 contigs in the *C. simum cottoni* assembly, indicated in green in Fig. 3.

We called consensus sequences from the 100 regions with the highest read depth and queried these against the BLAST nt database. All best-scoring sequence pairs (BSPs) for these queries were entirely within the γ-proteobacteria and were distributed between the orders Pseudomonadales and Enterobacterales (Table 2). Alignments within the Pseudomonadaceae family were particularly long on average (2,511 bp) and with very high (95%) sequence identity. We scaled the consensus queries by their read depth, which indicated that 87% of the reads with at least one BSP aligned within Pseudomonadaceae (Table 2). All of these alignments were furthermore with *Pseudomonas* sequences.

Table 2. Distribution of BLAST best-scoring sequence pairs (BSPs) for the 100 consensus sequences with the highest depth of coverage by taxonomic rank. Aligned reads (ARs) are the number of reads that contributed to a given consensus sequence query. %ARs are scaled by the sum of reads comprising queries with at least one BSP. Results are shown for taxonomic ranks comprising >5% of ARs and the hierarchy is collapsed to the lowest rank with identical results. %ID is mean percent sequence identity, length is the mean alignment length, e-value is the mean expect value, and bitscore is the mean bitscore.

kingdom	phylum	class	order	family	%ARs	%ID	length	e-value	bitscore

Prokaryota	Pseudomon-adota	γ-proteobacteria			100	93	2,154	2^{-52}	3,399
Prokaryota	Pseudomon-adota	γ-proteobacteria	Pseudomon-adales	Pseudomon-adaceae	87	95	2,511	2^{-52}	4,079
Prokaryota	Pseudomon-adota	γ-proteobacteria	Enterobacter-ales		13	88	1,207	2^{-77}	1,599
Prokaryota	Pseudomon-adota	γ-proteobacteria	Enterobacter-ales	Erwiniaceae	7	91	1,539	6^{-78}	2,195

Ceratotherium was among the genera used to compare the BSPs obtained from querying reads *vs.* consensus sequences against the nt database. The distribution of read BSPs was very similar to that found for the consensus queries, with 82% within Pseudomonadaceae and 16% within the order Enterobacterales (Table 3).

Both the read mapping and BLAST results show that reads classified to *Ceratotherium* are incorrectly assigned. The GBM correctly classified *Ceratotherium* as a negative occurrence, making this genus a straightforward example of a true negative.

Table 3. Distribution of BLAST best-scoring sequence pairs (BSPs) by taxonomic rank for 100 randomly-selected pair-end reads. Results are shown for taxonomic ranks comprising >5% of reads and the hierarchy is collapsed to the lowest rank with identical results. %ID is mean percent sequence identity, length is the mean alignment length, e-value is the mean expect value, and bitscore is the mean bitscore.

kingdom	phylum	class	order	family	%BSPs	%ID	length	e-value	bitscore
Prokaryota	Pseudomon-adota	γ-proteobacteria			99	97	132	4^{-28}	221
Prokaryota	Pseudomon-adota	γ-proteobacteria	Pseudomon-adales	Pseudomon-adaceae	82	98	133	2^{-29}	226
Prokaryota	Pseudomon-adota	γ-proteobacteria	Enterobacter-ales		16	94	128	2^{-27}	199
Prokaryota	Pseudomon-adota	γ-proteobacteria	Enterobacter-ales	Erwiniaceae	12	95	127	3^{-27}	203

Eucalyptus; Streptophyta; Magnoliopsida; Myrtales; Myrtaceae (3932)

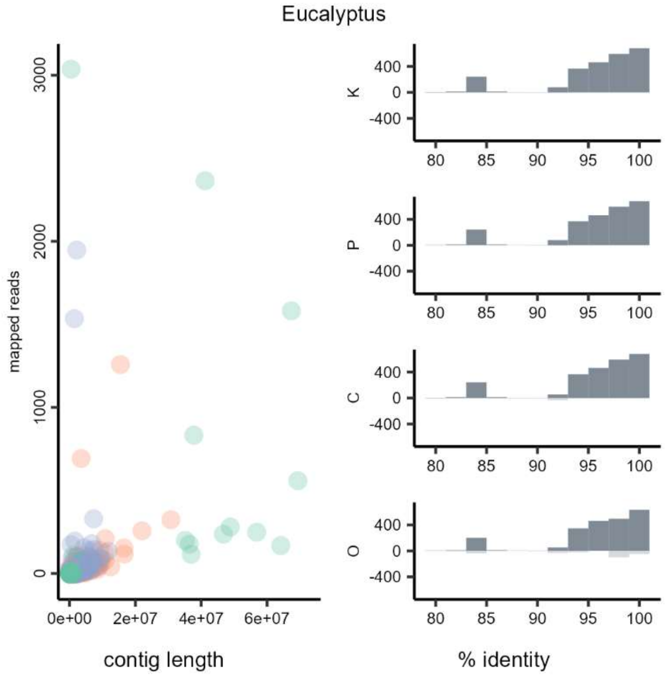

Figure 4. Left: number of mapped reads *vs*. contig length for contigs > 50 kb. Right: mirrored histograms of the sequence identity of aligned reads (ARs) with BLAST best-scoring sequence pairs (BSPs) within (top) or outside (bottom) the kingdom (K), phylum (P), class (C), and order (O) ranks of *Eucalyptus*. BSPs are shown for the 100 consensus sequences with the highest depth of coverage. ARs are the number of reads covering a given consensus sequence query and are given as *n*-fold differences.

Label: negative
Predicted probability: 0.00
Mapped reads: 87,806
Mapping rate: 39%
Base pairs queried: 21,477
Unique contigs queried: 87
Unique contigs aligned: 81

Eucalyptus was included in the gradient boosting machine (GBM) training dataset as a negative genus. Nuclear genome assemblies for *E. camaldulensis* (621.3 Mb, N50: 4 kb), *E. grandis* (608.0 Mb, N50: 48.8 Mb), *E. melliodora* (609.1 Mb, N50: 5.0 Mb), and *E. pauciflora* (564.7 Mb, N50: 3.1 Mb) were included in the Kraken 2 database.

Relatively few *Eucalyptus*-classified reads mapped back to their sequences in the reference database, with a 39% mapping rate compared to the mean rate of 75% (σ = 25%) for all 65 genera. Out of the mapped reads, 34% mapped to the *E. grandis* assembly (in green), followed by *E. pauciflora* (27%; blue), *E. melliodora* (22%; orange) and *E. camaldulensis* (16%; pink, not visible due to low N50). More reads tended to map to longer contigs ($r = 0.41$), although this correlation was weak due to the number of contigs with a disproportionately larger number of mapped reads. This tendency, although weak, was nevertheless stronger than in observed in the rest of the true negatives, the false positives, and some true positive genera (*e.g. Pieris*, *Limnephilus*).

We called consensus sequences from the 100 regions with the highest read depth and queried these against the BLAST nt database. Best-scoring sequence pairs (BSPs) for these queries were almost entirely within the class Magnoliopsida, most of which were also within the same order as *Eucalyptus,* the Myrtales (Table 3). This taxonomic distribution of BSPs was also unusual among the true negatives and the false positive genera (but see *Larix*). BSPs were short (*ca*. 240 bp) but with very high (≥ 89%) sequence identity (Table 4).

Contigs with > 1,000 mapped reads in Fig. 2 yielded consensus sequences with BSPs with the *Chamaenerion* mitochondrial genome. This included all four *Eucalyptus* assemblies, which were released by three different institutions. While all four could be independently contaminated by *Chamaenerion*, the alignments specifically with the mitochondrial genome suggest the

Eucalpyptus-classified reads could be conserved sequences in found in several lineages in the Myrtales or potentially in Magnoliopsida more generally.

Table 4. Distribution of BLAST best-scoring sequence pairs (BSPs) for the 100 consensus sequences with the highest depth of coverage by taxonomic rank. Aligned reads (ARs) are the number of reads that contributed to a given consensus sequence query. %ARs are scaled by the sum of reads comprising queries with at least one BSP. Results are shown for taxonomic ranks comprising >5% of ARs and the hierarchy is collapsed to the lowest rank with identical results. %ID is mean percent sequence identity, length is the mean alignment length, e-value is the mean expect value, and bitscore is the mean bitscore.

kingdom	phylum	class	order	family	%ARs	%ID	length	e-value	bitscore
Viridiplantae	Streptophyta				99	95	239	2^{-35}	382
Viridiplantae	Streptophyta	Magnoliopsida			98	95	240	2^{-35}	384
Viridiplantae	Streptophyta	Magnoliopsida	Myrtales		89	96	242	1^{-44}	389
Viridiplantae	Streptophyta	Magnoliopsida	Myrtales	Onagraceae	79	97	243	3^{-53}	404
Viridiplantae	Streptophyta	Magnoliopsida	Myrtales	Lythraceae	10	89	229	3^{-43}	305
Viridiplantae	Streptophyta	Magnoliopsida	Fagales		6	98	261	4^{-57}	448
Viridiplantae	Streptophyta	Magnoliopsida	Fagales	Betulaceae	5	99	272	2^{-71}	473

We scaled the consensus queries by their read depth to obtain the number of reads represented by each alignment. Scaled by depth, 79% of alignmenets were with sequences from the Onagaraceae family, which contains two genera abundant around the aerosol filter station and in Fennoscandia more generally (*Epilobium* and *Chamaenerion*). At the genus rank, most alignments were with *Oenothera* (42%), which can be found closer to the Gulf of Bothnia, and *Chamaenerion* (36%).

Eucalyptus was also one of the genera used to compare the BSPs obtained from querying reads *vs.* consensus sequences against the nt database. The two methods yielded very similar results, both in the taxonomic distribution of the reads and the high sequence identity of the alignments (Table 5).

In conclusion, *Eucalyptus* is a true negative, in the sense that none of the *Eucalyptus*-classified reads originated from their assigned genus. Unlike the other true negative examples, we found no clear evidence of reference contamination, and the read classifications seem to originate from conserved regions of Myrtales genomes. While *Eucalyptus* is a more nuanced example of a spurious taxon, the GBM correctly assigned it a very low predicted probability.

Table 5. Distribution of BLAST best-scoring sequence pairs (BSPs) by taxonomic rank for 100 randomly-selected pair-end reads. Results are shown for taxonomic ranks comprising >5% of reads and the hierarchy is collapsed to the lowest rank with identical results. %ID is mean percent sequence identity, length is the mean alignment length, e-value is the mean expect value, and bitscore is the mean bitscore.

kingdom	phylum	class	order	family	%BSPs	%ID	length	e-value	bitscore
Viridiplantae	Streptophyta				96	98	114	2^{-27}	196
Viridiplantae	Streptophyta	Magnoliopsida			93	98	115	2^{-27}	197
Viridiplantae	Streptophyta	Magnoliopsida	Myrtales		88	98	115	2^{-27}	197
Viridiplantae	Streptophyta	Magnoliopsida	Myrtales	Onagraceae	78	98	117	2^{-27}	200
Viridiplantae	Streptophyta	Magnoliopsida	Myrtales	Lythraceae	8	98	99	6^{-29}	171

***Hydrophis*; Chordata; Squamata; Hydrophiidae (8683)**

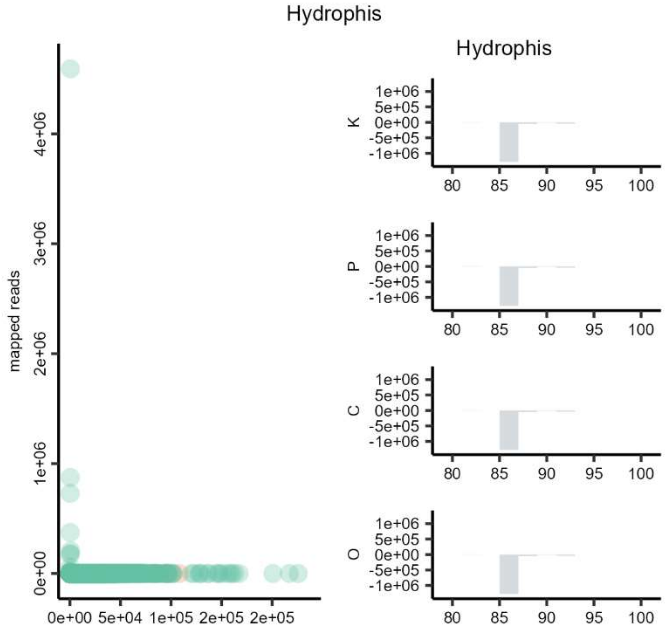

Figure 5. Left: number of mapped reads *vs*. contig length. Right: mirrored histograms of the sequence identity of aligned reads (ARs) with BLAST best-scoring sequence pairs (BSPs) within (top) or outside (bottom) the kingdom (K), phylum (P), class (C), and order (O) ranks of *Hydrophis*. BSPs are shown for the 100 consensus sequences with the highest depth of coverage. ARs are the number of reads covering a given consensus sequence query and are given as *n*-fold differences.

Label: negative
Predicted probability: 0.18
Mapped reads: 7,271,292
Mapping rate: 85%
Base pairs queried: 18,876
Unique contigs queried: 99
Unique contigs aligned: 56

Hydrophis, a group of sea snakes, was included in the gradient boosting machine (GBM) training dataset as a negative genus. Nuclear genome assembles for *H. cyanocinctus* (1.3 Gb, N50: 7 kb) and *H. hardwickii* (1.2 Gb, N50: 5 kb) were included in the Kraken 2 reference database.

Most (85%) of the *Hydrophis*-classified reads mapped back to their reference database sequences, all of which mapped to *H. cyanocinctus* assembly. 1,628 out of the 1,162,736 *Hydrophis* contigs had at least one mapped read, with no correlation between the number of mapped reads and contig length (Fig. 5).

We called consensus sequences from the 100 regions with the highest read depth and queried these against the BLAST nt database. All best-scoring sequence pairs (BSPs) were within the Pinaceae family, all of which were with *Pinus* at the genus rank (Table 6).

Table 6. Distribution of BLAST best-scoring sequence pairs (BSPs) for the 100 consensus sequences with the highest depth of coverage by taxonomic rank. Aligned reads (ARs) are the number of reads that contributed to a given consensus sequence query. %ARs are scaled by the sum of reads comprising queries with at least one BSP. Results are shown for taxonomic ranks comprising >5% of ARs and the hierarchy is collapsed to the lowest rank with identical results. %ID is mean percent sequence identity, length is the mean alignment length, e-value is the mean expect value, and bitscore is the mean bitscore.

kingdom	phylum	class	order	family	%ARs	%ID	length	e-value	bitscore
Viridiplantae	Streptophyta	Pinopsida	Pinales	Pinaceae	100	87	229	4^{-37}	281

Similarly, BSPs for 100 randomly-selected paired end reads were almost exclusively (98%) within Pinaceae, all of which were with *Pinus* at the genus rank (Table 7. These results unambiguously show that ‘Hydrophis’ is actually *Pinus*, making this genus a straightforward example of a true negative.

Table 7. Distribution of BLAST best-scoring sequence pairs (BSPs) by taxonomic rank for 100 randomly-selected pair-end reads. Results are shown for taxonomic ranks comprising >5% of reads and the hierarchy is collapsed to the lowest rank

with identical results. %ID is mean percent sequence identity, length is the mean alignment length, e-value is the mean expect value, and bitscore is the mean bitscore.

kingdom	phylum	class	order	family	%BSPs	%ID	length	e-value	bitscore
Viridiplantae	Streptophyta	Pinopsida	Pinales	Pinaceae	98	89	132	1^{-27}	167

Liriodendron; Streptophyta; Magnoliopsida; Magnoliales; Magnoliaceae (3413)

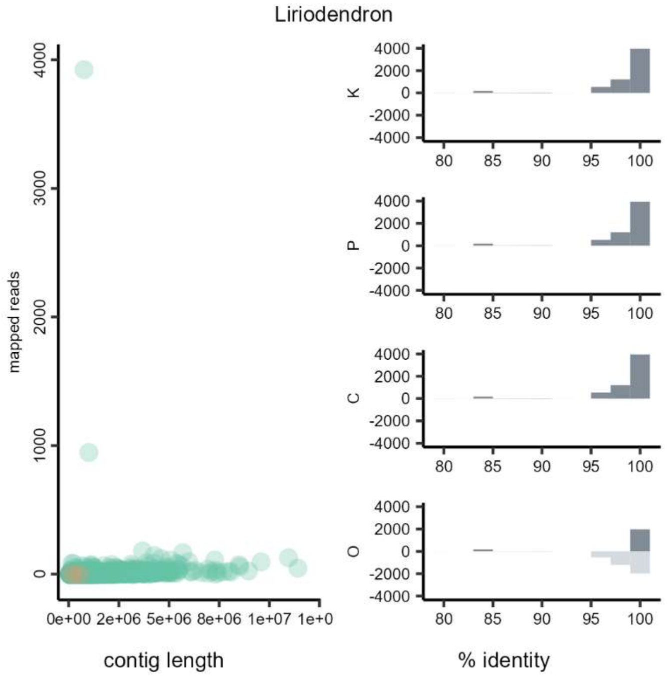

Figure 6. Left: number of mapped reads *vs*. contig length for contigs > 50 kb. Right: mirrored histograms of the sequence identity of aligned reads (ARs) with BLAST best-scoring sequence pairs (BSPs) within (top) or outside (bottom) the kingdom (K), phylum (P), class (C), and order (O) ranks of *Liriodendron*. BSPs are shown for the 100 consensus sequences with the highest depth of coverage. ARs are the number of reads covering a given consensus sequence query and are given as *n*-fold differences.

Label: negative
Predicted probability: 0.10
Mapped reads: 46,452
Mapping rate: 41%
Base pairs queried: 17,716
Unique contigs queried: 73
Unique contigs aligned: 45

Liriodendron comprises two deciduous trees, distributed in eastern North America and east Asia, and was included in the gradient boosting machine (GBM) training dataset as a negative genus. A nuclear genome assembly for *L. chinense* (1.7 Gb, N50: 2.1 Mb) was included in the Kraken 2 database.

Relatively few (41%) *Liriodendron*-classified reads mapped back to their sequences in the reference database. 947 out of the 4,178 contigs had at least one mapped read, but 36% mapped to two relatively long contigs (Fig. 6).

We called consensus sequences from the 100 regions with the highest read depth and queried these against the BLAST nt database. Best-scoring sequence pairs (BSPs) were almost entirely within the class Magnoliopsida and these were distributed among the Myrtales, Magnoliales, and Fagales (Table 8). As found in *Eucalyptus*, most alignments at the genus rank were with *Chamaenerion*, specifically with mitochondrial genomes. Within Magnoliales, BSPs were primarily with *Liriodendron* mitochondrial genomes that were not included in our Kraken 2 reference database. This included the contig in Fig. 4 with *ca.* 4,000 mapped reads. Betulaceae alignments were also with a mitochondrial genome, from *Alnus*, but also with chromosome-level sequences produced by two different institutions.

Table 8. Distribution of BLAST best-scoring sequence pairs (BSPs) for the 100 consensus sequences with the highest depth of coverage by taxonomic rank. Aligned reads (ARs) are the number of reads that contributed to a given consensus sequence query. %ARs are scaled by the sum of reads comprising queries with at least one BSP. Results are shown for taxonomic ranks comprising >5% of ARs and the hierarchy is collapsed to the lowest rank with identical results. %ID is

mean percent sequence identity, length is the mean alignment length, e-value is the mean expect value, and bitscore is the mean bitscore.

kingdom	phylum	class	order	family	%ARs	%ID	length	e-value	bitscore
Viridiplantae	Streptophyta				100	92	282	2^{-31}	426
Viridiplantae	Streptophyta	Magnoliopsida			99	91	306	2^{-31}	465
Viridiplantae	Streptophyta	Magnoliopsida	Myrtales	Onagraceae	46	96	388	1^{-56}	646
Viridiplantae	Streptophyta	Magnoliopsida	Magnoliales	Magnoliaceae	36	92	624	1^{-55}	927
Viridiplantae	Streptophyta	Magnoliopsida	Fagales	Betulaceae	17	91	247	1^{-48}	389

We queried 100 randomly selected *Liriodendron*-classified reads against the nt database and found a similar taxonomic distribution of the BSPs, also with high (97-99%) sequence identity (Table 9). Likewise, these BSPs were primarily with mitochondrial genomes from *Chamaenerion*, Oenothera (Myrtales: Onagraceae) and *Liriodendron*.

Given these results, and also those observed in *Eucalyptus*, the *Liriodendron*-classified reads seem better explained by the vagaries of genome evolution than contamination. Plant mitochondrial genomes, in particular, have a propensity for weirdness (Sullivan *et al*. 2019) and *Liriodendron*'s is unusually conserved in sequence and structure (Richardson *et al*. 2013). While the *Liriodendron*-classified reads may have a biological explanation, *Liriodendron* as genus is a spurious detection, and the GBM correctly assigned it a very low predicted probability.

Table 9. Distribution of BLAST best-scoring sequence pairs (BSPs) by taxonomic rank for 100 randomly-selected pair-end reads. Results are shown for taxonomic ranks comprising >5% of reads and the hierarchy is collapsed to the lowest rank with identical results. %ID is mean percent sequence identity, length is the mean alignment length, e-value is the mean expect value, and bitscore is the mean bitscore.

kingdom	phylum	class	order	family	%BSPs	%ID	length	e-value	bitscore
Viridiplantae	Streptophyta				97	97	127	2^{-27}	214
Viridiplantae	Streptophyta	Magnoliopsida			87	97	130	2^{-27}	219
Viridiplantae	Streptophyta	Magnoliopsida	Myrtales		61	99	134	3^{-29}	234
Viridiplantae	Streptophyta	Magnoliopsida	Myrtales	Onagraceae	58	99	133	3^{-29}	235
Viridiplantae	Streptophyta	Magnoliopsida	Magnoliales	Magnoliaceae	6	97	115	5^{-30}	187

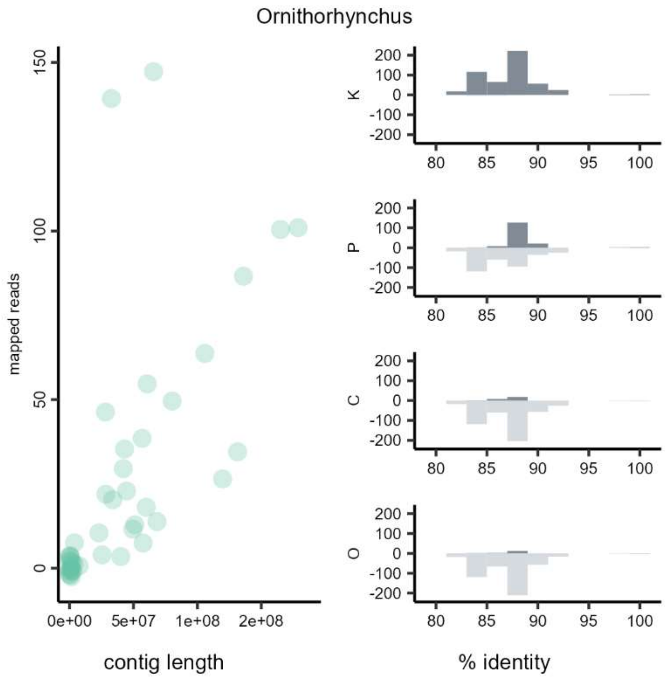

Figure 7. Left: number of mapped reads *vs*. contig length for contigs > 50 kb. Right: mirrored histograms of the sequence identity of aligned reads (ARs) with BLAST best-scoring sequence pairs (BSPs) within (top) or outside (bottom) the kingdom (K), phylum (P), class (C), and order (O) ranks of *Ornithorhynchus*. BSPs are shown for the 100 consensus sequences with the highest depth of coverage. ARs are the number of reads covering a given consensus sequence query and are given as *n*-fold differences.

***Ornithorhynchus*; Chordata; Mammalia; Monotremata; Ornithorhynchidae (9257)**

Label: negative
Predicted probability: 0.03
Mapped reads: 7,764
Mapping rate: 3%
Base pairs queried: 12,068
Unique contigs queried: 86
Unique contigs aligned: 57

Ornithorhynchus, the duck-billed platypus, was included in the gradient boosting machine (GBM) training dataset as a negative genus. A nuclear genome assembly for the only extant species, *O. anatinus*, was included in the Kraken 2 reference database (1.8 Gb, N50: 80.3 Mb).

Only 3% of the *Ornithorhynchus*-classified reads mapped back to reference database but contig length and mapped read count were positively correlated (r = 0.67; Fig. 7). This result was unexpected because contaminant sequences are generally not integrated into long contigs and instead assemble into their own short contigs.

We called consensus sequences from the 100 regions with the highest read depth and queried these against the BLAST nt database (Table 10). Best-scoring sequence pairs (BSPs) were distributed within Metazoa and most reads aligned with Insecta (62%) and Aves (21%). Sequence identity was relatively high with all BSPs from all lineages (85-83%).

Table 10. Distribution of BLAST best-scoring sequence pairs (BSPs) for the 100 consensus sequences with the highest depth of coverage by taxonomic rank. Aligned reads (ARs) are the number of reads that contributed to a given consensus sequence query. %ARs are scaled by the sum of reads comprising queries with at least one BSP. Results are shown for taxonomic ranks comprising >5% of ARs and the hierarchy is collapsed to the lowest rank with identical results. %ID is mean percent sequence identity, length is the mean alignment length, e-value is the mean expect value, and bitscore is the mean bitscore.

kingdom	phylum	class	order	family	%ARs	%ID	length	e-value	bitscore
Metazoa					98	88	180	1^{-37}	225
Metazoa	Arthropoda				64	86	181	2^{-37}	214
Metazoa	Arthropoda	Insecta			62	86	182	2^{-37}	214
Metazoa	Arthropoda	Insecta	Diptera		34	85	195	3^{-37}	222
Metazoa	Arthropoda	Insecta	Diptera	Drosophilidae	25	85	198	2^{-37}	226

kingdom	phylum	class	order	family	%ARs	%ID	length	e-value	bitscore
Metazoa	Arthropoda	Insecta	Lepidoptera		23	88	167	4^{-42}	210
Metazoa	Arthropoda	Insecta	Lepidoptera	Yponomeutidae	8	90	154	6^{-45}	208
Metazoa	Arthropoda	Insecta	Diptera	Chironomidae	7	85	207	9^{-42}	234
Metazoa	Chordata				32	90	178	1^{-42}	250
Metazoa	Chordata	Aves			21	93	195	5^{-57}	292
Metazoa	Chordata	Aves	Passeriformes		12	89	193	1^{-56}	258
Metazoa	Chordata	Aves	Passeriformes	Corvidae	11	87	215	2^{-70}	281
Metazoa	Chordata	Mammalia			6	87	154	4^{-42}	188
Metazoa	Chordata	Actinopteri			5	89	164	9^{-51}	218

We also queried 100 paired-end reads against the nt and combined RefSeq databases (Table 11). Only three had BSPs that passed our standard filtering thresholds. More lenient filtering (%ID > 70, length > 75 bp, e-value < 1^{-15}) resulted in six aligned reads. In either case, the BSPs were entirely from with Insecta, primarily with Lepidoptera and Diptera, as seen from the consensus sequence alignments (Table 10).

Table 11. Distribution of BLAST best-scoring sequence pairs (BSPs) by taxonomic rank for 100 randomly-selected pair-end reads. Results are shown for taxonomic ranks comprising >5% of reads and the hierarchy is collapsed to the lowest rank with identical results. %ID is mean percent sequence identity, length is the mean alignment length, e-value is the mean expect value, and bitscore is the mean bitscore.

kingdom	phylum	class	order	family	%BSPs	%ID	length	e-value	bitscore
Metazoa	Arthropoda	Insecta			100	90	103	4^{-22}	137
Metazoa	Arthropoda	Insecta	Coleoptera	Cantharidae	33	87	102	1^{-21}	117
Metazoa	Arthropoda	Insecta	Diptera	Anisopodidae	33	89	101	1^{-26}	133
Metazoa	Arthropoda	Insecta	Psocoptera	Mesopsocidae	33	94	107	2^{-35}	162

These BLAST results do not clearly implicate reference contamination, but the taxonomic distribution of BSPs do not seem consistent with conserved sequences, either. Many of the Insecta alignments were with sequences from the Wellcome Sanger Tree of Life Programme[1] but some were unrelated to this project. Similarly, about half of the Aves alignments were with sequences from the Vertebrate Genomes Project[2], who also produced the *Ornithorhynchus* assembly, but the remainder were from multiple groups with no apparent connection. All of the remaining Chordata alignments were from different projects and produced by different sequencing facilities.

Although we did not investigate this further, the reads classified to *Ornithorhynchus* may be mostly low-complexity, which could explain the low mapping rate, the lack of BLAST alignments for individual reads, and potentially the positive correlation between mapped reads and contig length. Why *Ornithorhynchus* was detected in this and other metagenome studies

[1] https://www.sanger.ac.uk/programme/tree-of-life/, last accessed August 20, 2024
[2] https://www.rockefeller.edu/research/vertebrate-genomes-project/, last accessed September 7, 2024

(Gonzalez et al. 2016) is unclear, but these reads show no evidence of actually being from a platypus and the GBM correctly classified it as a negative occurrence.

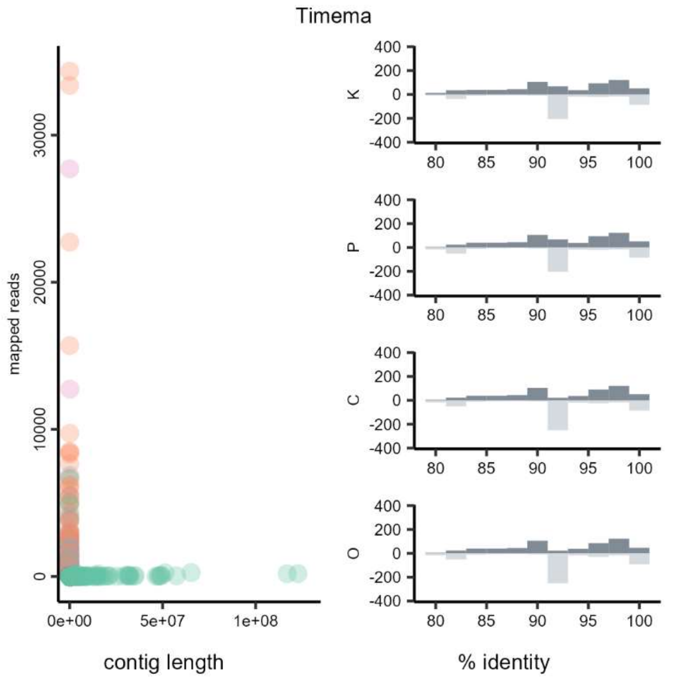

Figure 8. Left: number of mapped reads *vs*. contig length. Right: mirrored histograms of the sequence identity of aligned reads (ARs) with BLAST best-scoring sequence pairs (BSPs) within (top) or outside (bottom) the kingdom (K), phylum (P), class (C), and order (O) ranks of *Timema*. BSPs are shown for the 100 consensus sequences with the highest depth of coverage. ARs are the number of reads covering a given consensus sequence query and are given as *n*-fold differences.

***Timema*; Arthropoda; Insecta; Phasmatodea; Timematidae (61471)**

Label: negative
Predicted probability: 0.20
Mapped reads: 939,722
Mapping rate: 38%
Base pairs queried: 47,752
Unique contigs queried: 99
Unique contigs aligned: 36

Timema comprises walking-stick insects endemic to western North America and is the sole genus in Timematidae. More generally, phasmids have not been reported in Fennoscandia and are rare in northern mainland Europe.

Thirteen *Timema* nuclear genome assemblies were included in the Kraken 2 reference database: three *T. cristinae* assemblies released by the same institution (805.5 MB, N50: 263 kb; 862.3 Mb, N50: 7.6 Mb and 858.6 Mb, N50: 49.2 Mb) and ten assemblies with the same BioProject accession number, including *T. cristinae* and nine other species (976.2–1,139.5 Mb, N50: 4–208 kb).

Relatively few (38%) *Timema*-classified reads mapped back to the reference database. Of these, the majority (58%) mapped to the *T. douglasi* assembly (in orange in Fig. 8), followed by *T. poppense* (13%; blue), and *T. tahoe* (12%; pink). 9,076 contigs from 11 assemblies had at least one mapped read, out of a total of 4,167,808.

We called consensus sequences from the 100 regions with the highest read depth and queried these against the BLAST nt database. Most best-scoring sequence pairs (BSPs) were with bacteria, primarily within the orders Entomoplasmatales and Rickettsiales (Table 12). At the genus rank, 39% of ARs were with *Spiroplasma* (Mycoplasmatota: Mollicutes) and 27% with *Wolbachia* (Pseudomonadota: α-proteobacteria). Both are endosymbiotic with insects. The 10% of ARs with Pinaceae were specifically with *Picea*.

Table 12. Distribution of BLAST best-scoring sequence pairs (BSPs) for the 100 consensus sequences with the highest depth of coverage by taxonomic rank. Aligned reads (ARs) are the number of reads that contributed to a given consensus sequence query. %ARs are scaled by the sum of reads comprising queries with at least one BSP. Results are shown for taxonomic ranks comprising >5% of ARs and the hierarchy is collapsed to the lowest rank with identical results. %ID is

mean percent sequence identity, length is the mean alignment length, e-value is the mean expect value, and bitscore is the mean bitscore.

kingdom	phylum	class	order	family	%ARs	%ID	length	e-value	bitscore
Prokaryota					71	94	814	9^{-69}	1,336
Prokaryota	Mycoplasm-atota	Mollicutes	Entomop-lasmatales	Spiroplasm-ataceae	39	91	368	4^{-68}	539
Prokaryota	Pseudomon-adota				32	95	949	2^{-81}	1,577
Prokaryota	Pseudomon-adota	α-proteobacteria	Rickettsiales		28	97	1,000	1^{-97}	1,690
Prokaryota	Pseudomon-adota	α-proteobacteria	Rickettsiales	Anaplasm-ataceae	27	97	1,060	4^{-181}	1,797
Metazoa					19	88	329	3^{-32}	424
Metazoa	Arthropoda				15	90	296	5^{-32}	434
Metazoa	Arthropoda	Arachnida	Trombidi-formes	Phytoptidae	9	88	240	2^{-31}	324
Metazoa	Arthropoda	Insecta			7	91	315	5^{-34}	473
Viridiplantae	Streptophyta	Pinopsida	Pinales	Pinaceae	10	82	222	3^{-30}	216

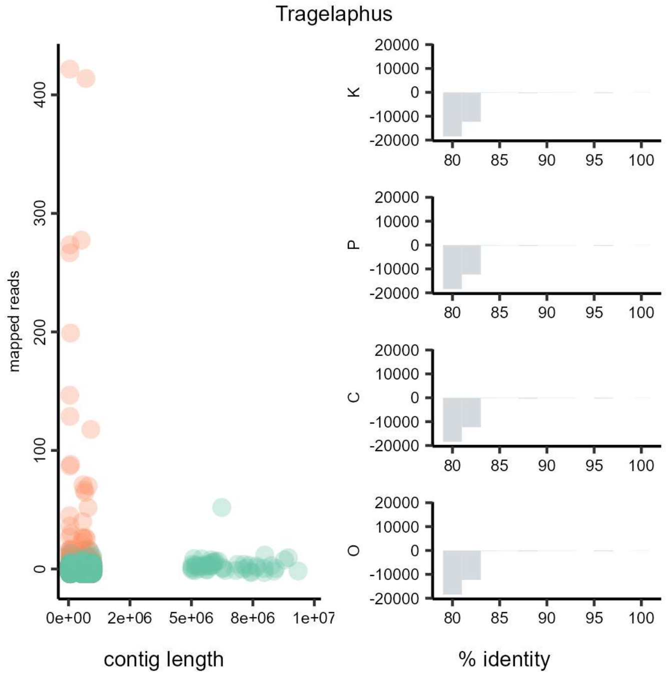

Figure 9. Left: number of mapped reads *vs*. contig length for contigs > 50 kb. Right: mirrored histograms of the sequence identity of aligned reads (ARs) with BLAST best-scoring sequence pairs (BSPs) within (top) or outside (bottom) the kingdom (K), phylum (P), class (C), and order (O) ranks of *Tragelaphus*. BSPs are shown for the 100 consensus sequences with the highest depth of coverage. ARs are the number of reads covering a given consensus sequence query and are given as *n*-fold differences.

Tragelaphus; Chordata; Mammalia; Artiodactyla; Bovidae (9944)

Label: negative
Predicted probability: 0.15
Mapped reads: 2,418,486
Mapping rate: 81%
Base pairs queried: 74,407
Unique contigs queried: 81
Unique contigs aligned: 43

Tragelaphus is a genus of spiral-horned antelopes distributed from the Sahel to South Africa. Full-length nuclear genome assemblies for seven *Tragelaphus* species were included in the Kraken 2 reference database (2.5 – 3.1 Gb, N50: 5 – 1,410 kb), along with a partial assembly for *T. strepsiceros* (90.7 Mb, N50: 437 bp).

Most (83%) *Tragelaphus*-classified reads mapped back to their sequences in the reference database. However, 98% of these mapped to two assemblies, *T. eurycerus* (86%, in orange) and *T. spekii* (12%, in green in Fig. 7). At least one

read mapped to 19,912 out of the 3,308,908 contigs in these two assemblies.

We called consensus sequences from the 100 regions with the highest read depth and queried these against the BLAST nt database. Best-scoring sequence pairs (BSPs) for these queries were almost entirely within the grass family, Poaceae (Table 13).

Table 13. Distribution of BLAST best-scoring sequence pairs (BSPs) for the 100 consensus sequences with the highest depth of coverage by taxonomic rank. Aligned reads (ARs) are the number of reads that contributed to a given consensus sequence query. %ARs are scaled by the sum of reads comprising queries with at least one BSP. Results are shown for taxonomic ranks comprising >5% of ARs and the hierarchy is collapsed to the lowest rank with identical results. %ID is mean percent sequence identity, length is the mean alignment length, e-value is the mean expect value, and bitscore is the mean bitscore.

kingdom	phylum	class	order	family	%ARs	%ID	length	e-value	bitscore
Viridiplantae	Streptophyta	Magnoliopsida			97	89	328	8^{-46}	465
Viridiplantae	Streptophyta	Magnoliopsida	Poales	Poaceae	96	81	239	2^{-45}	232

Tragelaphus was among the genera used to compare the BSPs obtained from querying reads *vs.* consensus sequences against the nt database. These alignments were primarily with Poaceae (86%), as found for the consensus queries, but 14% were with *Aspergillus*, a genus of common molds in indoor and outdoor environments (Table 4).

Table 14. Distribution of BLAST best-scoring sequence pairs (BSPs) by taxonomic rank for 100 randomly-selected pair-end reads. Results are shown for taxonomic ranks comprising >5% of reads and the hierarchy is collapsed to the lowest rank with identical results. %ID is mean percent sequence identity, length is the mean alignment length, e-value is the mean expect value, and bitscore is the mean bitscore.

kingdom	phylum	class	order	family	%reads	%ID	length	e-value	bitscore
Viridiplantae	Streptophyta	Magnoliopsida	Poales	Poaceae	86	86	135	2^{-27}	154
Fungi	Ascomycota	Eurotiomycetes	Eurotiales	Aspergillaceae	14	90	127	2^{-28}	173

True positives

Birds

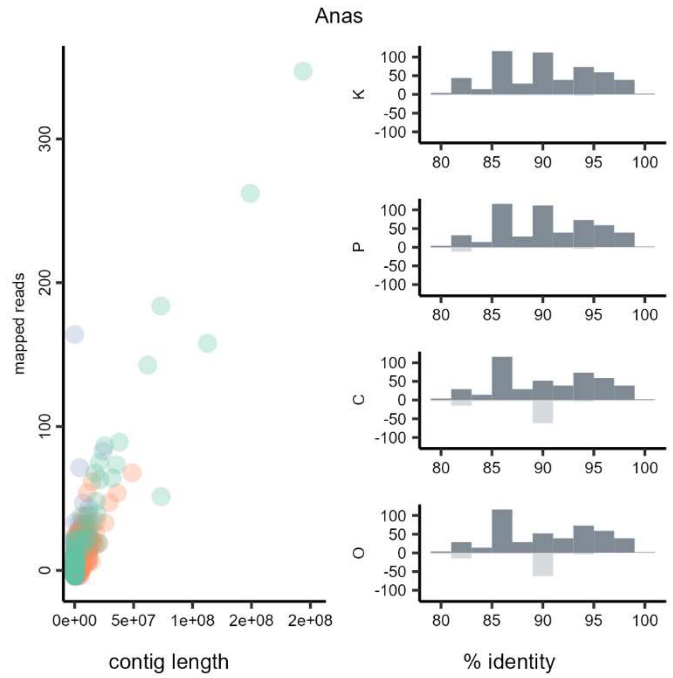

Figure 10. Left: number of mapped reads *vs*. contig length for contigs > 50 kb. Right: mirrored histograms of the sequence identity of aligned reads (ARs) with BLAST best-scoring sequence pairs (BSPs) within (top) or outside (bottom) the kingdom (K), phylum (P), class (C), and order (O) ranks of *Anas*. BSPs are shown for the 200 consensus sequences with the highest depth of coverage. ARs are the number of reads covering a given consensus sequence query and are given as *n*-fold differences.

Anas; Chordata; Aves Anseriformes; Anatidae (8835)

Label: positive
Predicted probability: 0.84
Mapped reads: 40,542
Mapping rate: 48%
Base pairs queried: 36,821
Unique contigs queried: 163
Unique contigs aligned: 72

Anas was included in the gradient boosting machine (GBM) training dataset as a positive taxon based on occurrence reports within 40 km of the aerosol filter station. *A. crecca, A. penelope* and *A. platyrhynchos* are common in the region and throughout Fennoscandia more broadly. Genome assemblies for *A. platyrhynchos* (1.1 Gb, N50: 73.1 Mb), *A. platyrhynchos* subsp. *platyrhynchos* (1.0 Gb, N50: 9.2 Mb), and *A. zonorhyncha* (1.1 Gb, N50: 2.3 Mb) were included in the Kraken 2 reference database.

48% of the *Anas*-classified reads mapped back to their reference sequences (Fig 8). A similar proportion of reads mapped to the *A. zonorhyncha* assembly (41%, in blue) and the *A. platyrhynchos* assemblies (58%, in green and orange). Reads mapped to *A. zonorhyncha* are obscured in Fig. 10 due to lower contiguity of this assembly, however, more reads mapped to longer contigs irrespective of assembly ($r = 0.89$), the expected pattern for a true positive genus.

We called consensus sequences from the 100 regions with the highest read depth and queried these against the BLAST nt database. Almost all of the best-scoring sequence pairs (BSPs) were within the Anatidae family. However, a consensus query called from 148 reads mapped to a 1,036 bp contig was most similar to human sequences (Table 15). To investigate the possibility of additional reference contamination, we expanded the BLAST analysis to include the next 100 regions with the highest read depth. We found no additional human-like sequences or evidence of other contamination in this second set of queries (Table 15). Scaling the consensus queries by their read depth revealed that *ca.* 8% of the *Anas*-classified may be of human origin due to contamination in the reference sequences. While the 13% reads aligning outside of Anatidae are potentially misclassified, 87% of reads were unambiguously similar to other waterfowl, and 54%

had BSPs within *Anas* at the genus rank. The genus is thus correctly classified as a positive occurrence.

Table 15. Distribution of BLAST best-scoring sequence pairs (BSPs) for the 200 consensus sequences with the highest depth of coverage by taxonomic rank. Aligned reads (ARs) are the number of reads that contributed to a given consensus sequence query. %ARs are scaled by the sum of reads comprising queries with at least one BSP. Results are shown for taxonomic ranks comprising >5% of ARs and the hierarchy is collapsed to the lowest rank with identical results. %ID is mean percent sequence identity, length is the mean alignment length, e-value is the mean expect value, and bitscore is the mean bitscore.

kingdom	phylum	class	order	family	%ARs	%ID	length	e-value	bitscore
Metazoa					97	93	288	4^{-35}	420
Metazoa	Chordata				96	93	289	4^{-35}	422
Metazoa	Chordata	Aves	Anseriformes	Anatidae	87	93	288	4^{-35}	422
Metazoa	Chordata	Mammalia	Primates	Hominidae	8	90	319	1^{-116}	435

***Corvus*; Chordata; Aves; Passeriformes; Corvidae (30420)**

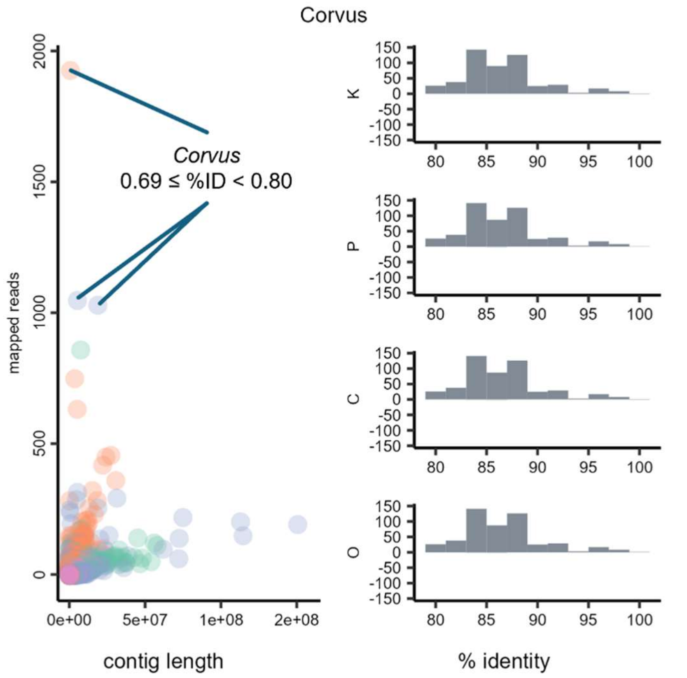

Figure 11. Left: number of mapped reads *vs*. contig length for contigs > 50 kb. Right: mirrored histograms of the sequence identity of aligned reads (ARs) with BLAST best-scoring sequence pairs (BSPs) within (top) or outside (bottom) the kingdom (K), phylum (P), class (C), and order (O) ranks of *Corvus*. BSPs are shown for the 100 consensus sequences with the highest depth of coverage. ARs are the number of reads covering a given consensus sequence query and are given as *n*-fold differences.

Label: positive
Predicted probability: 0.89
Base pairs queried: 42,543
Mapped reads: 303,222
Mapping rate: 74%
Unique contigs queried: 74
Unique contigs aligned: 61

Corvus was included in the gradient boosting machine (GBM) training dataset as a positive taxon based on occurrence reports within 40 km of the aerosol filter station. *C. cornix* and *C. corvax* are common in the region and throughout Fennoscandia more generally.

Nuclear genome assemblies for *C. brachyrhynchos* (1.1 Gb, N50: 29 kb), *C. cornix* (1.0 Gb, N50: 8.9 Mb), *C. hawaiiensis* (1.1 Gb, N50: 7.7 Mb), and *C. moneduloides* (905 Mb, N50: 2.6 Mb) were included in the Kraken 2 reference database.

74% of the *Corvus*-classified reads could be mapped back to their reference sequences (Fig. 11). Of these, the largest fraction (51%) mapped to *C. hawaiiensis* (blue), followed by *C. moneduloides* (21%, pink), *C. cornix* (16%, orange) and *C. brachyrhynchos* (9.8%, green). *C. corax* is more closely related to *C. hawaiiensis* than the other species (Jønsson 2012), which likely explains the taxonomic distribution of read mappings. The number of reads mapped to a contig generally increased with contig length, but

the strength of this relationship varied by assembly, from $r = 0.30$ for reads mapped to *C. cornix* and *C. moneduloides* to $r = 0.50$ for *C. hawaiiensis* and $r = 0.79$ for *C. brachyrhynchos.*

We called consensus sequences from the 100 regions with the highest read depth and queried these against the BLAST nt database. Best-scoring sequence pairs (BSPs) for these queries were almost entirely within the avian order Passeriformes (Table 16). We scaled the consensus queries by their read depth to obtain the number of reads represented by each alignment. 83% of the corresponding read alignments were within the Corvidae family, all of which were specifically aligned with *Corvus.* Consensus sequences from all contigs with an unexpectedly large number of mapped reads in Fig. 11 all had BSPs only within Corvidae, although the sequence identity was low in some alignments.

Table 16. Distribution of BLAST best-scoring sequence pairs (BSPs) for the 100 consensus sequences with the highest depth of coverage by taxonomic rank. Aligned reads (ARs) are the number of reads that contributed to a given consensus sequence query. %ARs are scaled by the sum of reads comprising queries with at least one BSP. Results are shown for taxonomic ranks comprising >5% of ARs and the hierarchy is collapsed to the lowest rank with identical results. %ID is mean percent sequence identity, length is the mean alignment length, e-value is the mean expect value, and bitscore is the mean bitscore.

kingdom	phylum	class	order	family	%ARs	%ID	length	e-value	bitscore
Metazoa					100	87	326	9^{-39}	392
Metazoa	Chordata	Aves	Passeriformes		99	87	331	9^{-39}	399
Metazoa	Chordata	Aves	Passeriformes	Corvidae	83	87	346	5^{-40}	431
Metazoa	Chordata	Aves	Passeriformes	Estrildidae	9	84	229	2^{-43}	243

Corvus was among the genera used to compare the BSPs obtained from querying reads *vs.* consensus sequences against the nt database. All alignments for 100 randomly-selected paired end reads were within Aves but BSPs were not as concentrated within Passeriformes or Corvidae (Table 17). We also queried the reads against the combined pro- and eukaryote RefSeq databases. All of these alignments were within Corvidae, which suggests the more ambiguous taxonomic distribution from the nt alignments resulted from database composition rather than an avian contaminant.

Table 17. Distribution of BLAST best-scoring sequence pairs (BSPs) by taxonomic rank for 100 randomly-selected pair-end reads. Results are shown for taxonomic ranks comprising >5% of reads and the hierarchy is collapsed to the lowest rank with identical results. %ID is mean percent sequence identity, length is the mean alignment length, e-value is the mean expect value, and bitscore is the mean bitscore.

kingdom	phylum	class	order	family	%BSPs	%ID	length	e-value	bitscore
nt database									
Metazoa	Chordata	Aves			100	92	125	2^{-27}	177
Metazoa	Chordata	Aves	Passeriformes		75	93	122	1^{-27}	181
Metazoa	Chordata	Aves	Passeriformes	Corvidae	46	95	124	2^{-30}	194
Metazoa	Chordata	Aves	Passeriformes	Cinclidae	10	91	109	6^{-27}	149
Metazoa	Chordata	Aves	Passeriformes	Sylviidae	8	90	129	1^{-33}	174
Metazoa	Chordata	Aves	Accipitriformes	Accipitridae	19	88	134	5^{-27}	164
prokaryote and eukaryote RefSeq databases									
Metazoa	Chordata	Aves	Passeriformes	Corvidae	100	98	115	1^{-28}	196

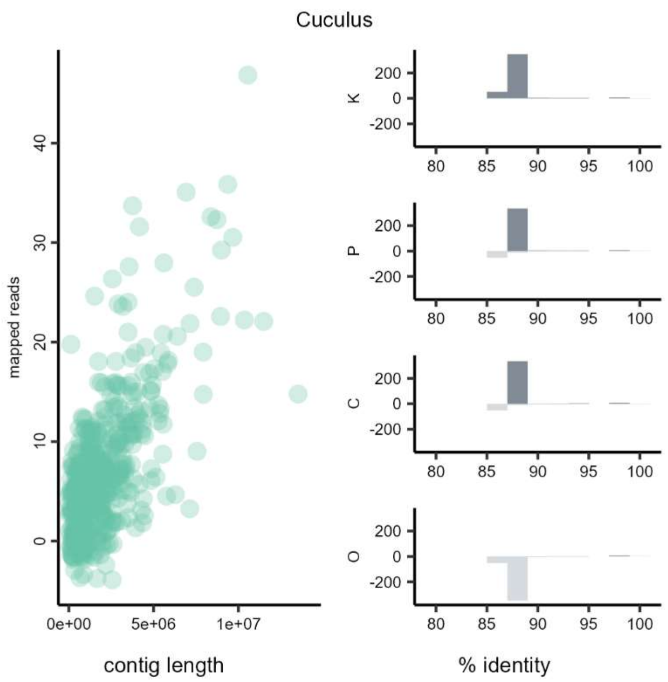

Figure 12. Left: number of mapped reads *vs*. contig length for contigs > 50 kb. Right: mirrored histograms of the sequence identity of aligned reads (ARs) with BLAST best-scoring sequence pairs (BSPs) within (top) or outside (bottom) the kingdom (K), phylum (P), class (C), and order (O) ranks of *Cuculus*. BSPs are shown for the 100 consensus sequences with the highest depth of coverage. ARs are the number of reads covering a given consensus sequence query and are given as *n*-fold differences.

***Cuculus*; Chordata; Aves; Cuculiformes; Cuculidae (33592)**

Label: positive
Predicted probability: 0.78
Mapped reads: 4,928
Mapping rate: 11%
Base pairs queried: 45801
Unique contigs queried: 156
Unique contigs aligned: 12

A nuclear genome assembly for *Cuculus canorus* (0.98 Gb, N50: 3.1 Mb) was included in the Kraken 2 reference database. The mapping rate for *Cuculus*-classified reads was relatively low (11%) but length and the number of mapped reads were positively correlated ($r = 0.71$; Fig. 12).

Consensus query BSPs were predominantly within Aves but few were within Cuculiformes (Table 18). This is likely because there were only 2,938 non-mitochondrial sequences from the Cuculiformes in the database (July 2024), although it is unclear why most alignments were within the Corvidae. Insects comprised *ca*. 13% of alignments.

Table 18. Distribution of BLAST best-scoring sequence pairs (BSPs) for the200 consensus sequences with the highest depth of coverage by taxonomic rank. Aligned reads (ARs) are the number of reads that contributed to a given consensus sequence query. %ARs are scaled by the sum of reads comprising queries with at least one BSP. Results are shown for taxonomic ranks comprising >5% of ARs and the hierarchy is collapsed to the lowest rank with identical results. %ID is mean percent sequence identity, length is the mean alignment length, e-value is the mean expect value, and bitscore is the mean bitscore.

kingdom	phylum	class	order	family	%readss	%ID	length	e-value	bitscore
Metazoa					100	88	179	8E-28	228
Metazoa	Chordata				87	89	185	1E-27	239
Metazoa	Chordata	Aves			86	89	185	1E-27	239
Metazoa	Chordata	Aves	Passeriformes		68	95	253	5E-86	386
Metazoa	Chordata	Aves	Passeriformes	Corvidae	63	88	452	6E-162	586
Metazoa	Chordata	Aves	Cuculiformes	Cuculidae	5	96	170	5E-40	275
Metazoa	Arthropoda	Insecta			13	86	156	1E-39	187
Metazoa	Arthropoda	Insecta	Lepidoptera		12	86	166	3E-40	197
Metazoa	Arthropoda	Insecta	Lepidoptera	Yponomeutidae	11	87	153	6E-40	184

Given the low mapping rate of the *Cuculus*-classified reads, we also queried all 6,140 reads > 100 bp against the nt database. As with the consensus queries, a majority of alignments were within Aves but few were within the Cuculiformes. However, the read BSPs within Aves were not concentrated within the Corvidae and were instead distributed across four different orders. Arthropods comprised 15% of the read BSPs.

While we cannot exclude the possibility that some *Cuculus*-classified reads may have originated from other birds, these had lower sequence identity than the alignments within Cuculidae. This suggests the taxonomic distribution of BSPs among birds is likely due to the limited number of Cuculiformes sequences in the nt database. However, the alignments with Insects unambiguously indicate that some fraction of the *Cuculus* reads are misclassified. Assuming all non-Aves alignments result from read misclassification gives an error rate between 14% (Table 18) and 29% (Table 19).

Table 19. Distribution of BLAST best-scoring sequence pairs (BSPs) by taxonomic rank for 6,140 reads. Results are shown for taxonomic ranks comprising >5% of reads and the hierarchy is collapsed to the lowest rank with identical results. %ID is mean percent sequence identity, length is the mean alignment length, e-value is the mean expect value, and bitscore is the mean bitscore.

kingdom	phylum	class	order	family	%reads	%ID	length	e-value	bitscore
Metazoa					96	87	124	6E-13	148
Metazoa	Chordata				76	87	124	6E-13	152
Metazoa	Chordata	Aves			71	88	124	4E-13	154
Metazoa	Chordata	Aves	Accipitriformes		25	86	127	1E-13	149
Metazoa	Chordata	Aves	Accipitriformes	Accipitridae	24	86	127	1E-13	149
Metazoa	Chordata	Aves	Cuculiformes	Cuculidae	10	93	125	3E-14	188
Metazoa	Chordata	Aves	Suliformes	Phalacrocoracidae	6	86	124	2E-14	141
Metazoa	Chordata	Aves	Charadriiformes		9	87	124	6E-13	150
Metazoa	Chordata	Aves	Charadriiformes	Laridae	5	87	123	6E-13	147
Metazoa	Arthropoda				15	86	119	2E-13	131
Metazoa	Arthropoda	Insecta			12	86	115	2E-13	130
Metazoa	Arthropoda	Insecta	Lepidoptera		5	86	110	2E-13	126

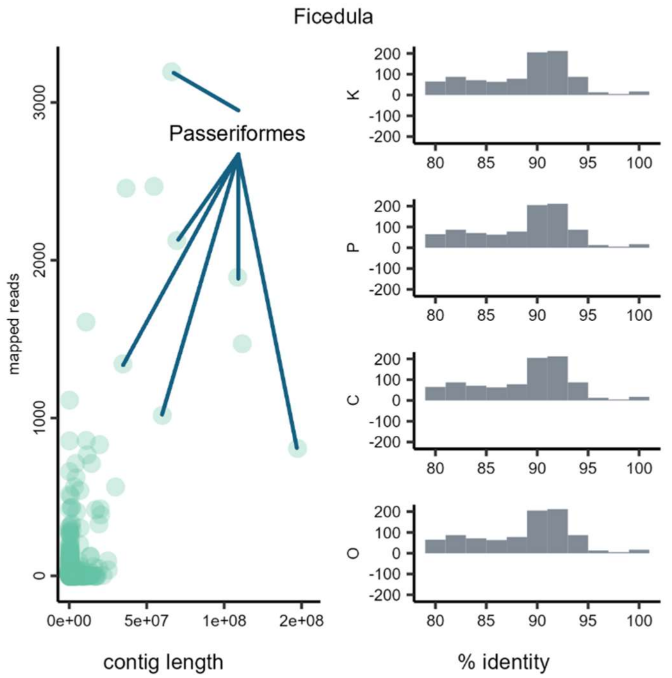

Figure 13. Left: number of mapped reads *vs*. contig length for contigs > 50 kb. Right: mirrored histograms of the sequence identity of aligned reads (ARs) with BLAST best-scoring sequence pairs (BSPs) within (top) or outside (bottom) the kingdom (K), phylum (P), class (C), and order (O) ranks of *Ficedula*. BSPs are shown for the 100 consensus sequences with the highest depth of coverage. ARs are the number of reads covering a given consensus sequence query and are given as *n*-fold differences.

Ficedula; Chordata; Aves; Passeriformes; Muscicapidae (36292)

Label: positive
Predicted probability: 0.97
Mapped reads: 300,238
Mapping rate: 84%
Base pairs queried: 66,728
Unique contigs queried: 65
Unique contigs aligned: 45

Ficedula included in the gradient boosting machine (GBM) training dataset as a positive genus based on occurrence records < 40 km of the aerosol filter station. *F. hypoleuca* is the only species present in most of Fennoscandia, except for a few Baltic islands where *F. alibicollis* also breeds. A genome assembly for *F. albicollis* (1.9 Gb, N50: 16.9 Mb) was included in the Kraken 2 reference database.

Most (84%) of the *Ficedula*-classified reads could be mapped back to their sequences in the reference database. Mapped reads and contig length were positively correlated ($r = 0.64$; Fig. 14), as expected for a true positive taxon, but this relationship was driven by contigs > 100 kb.

We called consensus sequences from the 100 regions with the highest read depth and queried these against the BLAST nt database. All best-scoring sequence pairs (BSPs) for these queries were within the same order as *Ficedula,* the Passeriformes (Table 20), including consensus queries from contigs with high read depth, as indicated in Fig. 14.

At the family rank, BSPs were primarily within Turdidae, rather than Muscicapidae (Table 20). Rescaled by read depth, 82% of alignments were within Turdidae, compared to 8% within Muscicapidae. We observed a similar taxonomic distribution of alignments for *Saxicola*, another flycatcher in the Muscicapidae family. Both genera were previously grouped in Turdidae and work to define reciprocally monophyletic families appears ongoing (Sangster et al. 2010), which likely explains the taxonomic distribution of the BSPs.

Table 20. Distribution of BLAST best-scoring sequence pairs (BSPs) for the 100 consensus sequences with the highest depth of coverage by taxonomic rank. Aligned reads (ARs) are the number of reads that contributed to a given consensus sequence query. %ARs are scaled by the sum of reads comprising queries with at least one BSP. Results are shown for taxonomic ranks comprising >5% of ARs and the hierarchy is collapsed to the lowest rank with identical results. %ID is

mean percent sequence identity, length is the mean alignment length, e-value is the mean expect value, and bitscore is the mean bitscore.

kingdom	phylum	class	order	family	%ARs	%ID	length	e-value	bitscore
Metazoa	Chordata	Aves	Passeriformes		100	87	497	2^{-31}	617
Metazoa	Chordata	Aves	Passeriformes	Turdidae	82	87	538	3^{-31}	656
Metazoa	Chordata	Aves	Passeriformes	Muscicapidae	8	91	458	6^{-34}	711

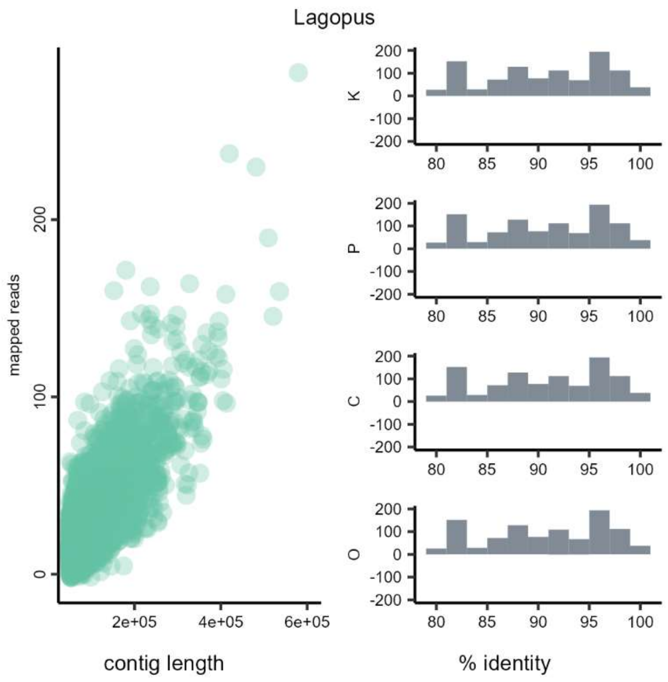

Figure 14. Left: number of mapped reads *vs*. contig length for contigs > 50 kb. Right: mirrored histograms of the sequence identity of aligned reads (ARs) with BLAST best-scoring sequence pairs (BSPs) within (top) or outside (bottom) the kingdom (K), phylum (P), class (C), and order (O) ranks of *Lagous*. BSPs are shown for the 100 consensus sequences with the highest depth of coverage. ARs are the number of reads covering a given consensus sequence query and are given as *n*-fold differences.

***Lagopus*; Chordata; Aves; Galliformes; Phasianidae (30409)**

Label: positive
Predicted probability: 0.97
Mapped reads: 331,176
Mapping rate: 91%
Base pairs queried: 43,192
Unique contigs queried: 88
Unique contigs aligned: 71

A nuclear genome assembly for *Lagopus muta japonica* (968.2 Gb, N50: 62 kb) was included in the Kraken 2 reference library. Two *Lagopus* species are present in Fennoscandia, *L. lagopus* and *L. muta.*

Most (90%) *Lagopus*-classified reads could be mapped back to their sequences in the reference database. The number of mapped reads per contig was strongly correlated with contig length ($r = 0.78$), as expected for a true-positive taxon (Fig. 15).

We called consensus sequences from the 100 regions with the highest read depth and queried these against the BLAST nt database. Almost all BSPs were with taxa in the same family as *Lagopus*, the Phasianidae (Table 21). DNA from another phasianid, the domestic chicken (*Gallus gallus*), has been reported as a contaminant in laboratory reagents (Leonard et al. 2007). However, we found no evidence to suggest chicken contamination in the *Lagopus*-classified reads. We scaled the consensus queries by their read depth to obtain the number of reads represented by each alignment, and found that 88% of these read alignments were with other grouse taxa (*Tetrao*, *Tympanuchus,* or *Lagopus*) and 12% were with *Gallus*. Although grouse and *Gallus* both had high sequence identities (93%), alignments with grouse were longer on average (608 *vs*. 219 bp).

Table 21. Distribution of BLAST best-scoring sequence pairs (BSPs) for the 100 consensus sequences with the highest depth of coverage by taxonomic rank. Aligned reads (ARs) are the number of reads that contributed to a given consensus sequence query. %ARs are scaled by the sum of reads comprising queries with at least one BSP. Results are shown for taxonomic ranks comprising >5% of ARs and the hierarchy is collapsed to the lowest rank with identical results. %ID is

mean percent sequence identity, length is the mean alignment length, e-value is the mean expect value, and bitscore is the mean bitscore.

kingdom	phylum	class	order	family	%ARs	%ID	length	e-value	bitscore
Metazoa	Chordata	Aves			100	93	496	3^{-29}	740
Metazoa	Chordata	Aves	Galliformes	Phasianidae	99	93	502	3^{-29}	749

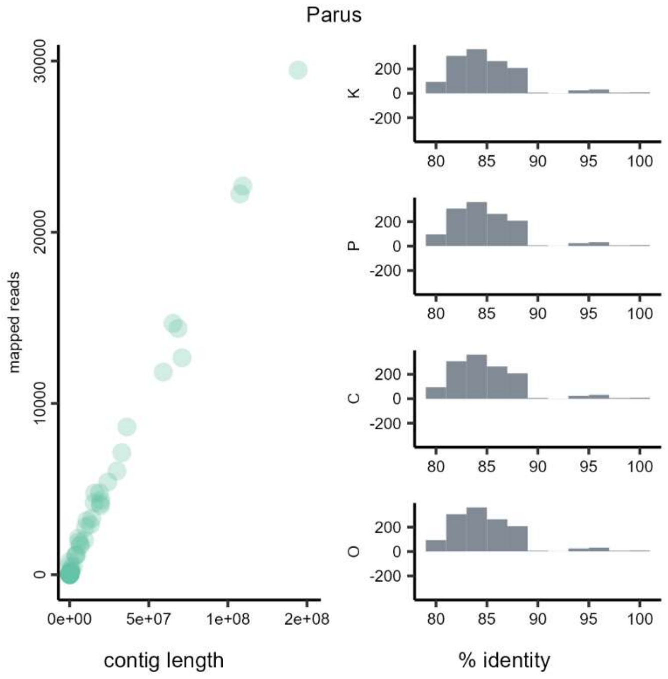

Figure 15. Left: number of mapped reads *vs*. contig length for contigs > 50 kb. Right: mirrored histograms of the sequence identity of aligned reads (ARs) with BLAST best-scoring sequence pairs (BSPs) within (top) or outside (bottom) the kingdom (K), phylum (P), class (C), and order (O) ranks of *Parus*. BSPs are shown for the 100 consensus sequences with the highest depth of coverage. ARs are the number of reads covering a given consensus sequence query and are given as *n*-fold differences.

***Parus*; Chordata; Aves; Passeriformes; Paridae (9154)**

Label: positive
Predicted probability: 0.99
Mapped reads: 235,330
Mapping rate: 91%
Base pairs queried: 31,915
Unique contigs queried: 51
Unique contigs aligned: 29

Parus was included in the gradient boosting machine (GBM) training dataset as a positive genus based on occurrence records within 40 km of the aerosol filter station. A nuclear genome assembly for *Parus major* (968.6 Mb, N50: 68.5 Mb), which is the only species recorded in northern Sweden, was included in the Kraken 2 reference database.

Most (91%) of the *Parus*-classified reads mapped back to their reference database sequences. The number of mapped reads per contig was strongly correlated with contig length (r = 0.997, Fig. 16), making *Parus* an exceptionally good example of the pattern expected from a true positive genus.

We called consensus sequences from the 100 regions with the highest read depth and queried these against the BLAST nt database. All best-scoring sequence pairs (BSPs) for these queries were within Passeriformes, almost of all of which were also within the Paridae family (Table 22).

Table 22. Distribution of BLAST best-scoring sequence pairs (BSPs) for the 100 consensus sequences with the highest depth of coverage by taxonomic rank. Aligned reads (ARs) are the number of reads that contributed to a given consensus sequence query. %ARs are scaled by the sum of reads comprising queries with at least one BSP. Results are shown for taxonomic ranks comprising >5% of ARs and the hierarchy is collapsed to the lowest rank with identical results. %ID is mean percent sequence identity, length is the mean alignment length, e-value is the mean expect value, and bitscore is the mean bitscore.

kingdom	phylum	class	order	family	%ARs	%ID	length	e-value	bitscore
Metazoa	Chordata	Aves	Passeriformes		100	86	357	1^{-32}	430

Metazoa	Chordata	Aves	Passeriformes	Paridae	99	86	362	2^{-32}	438

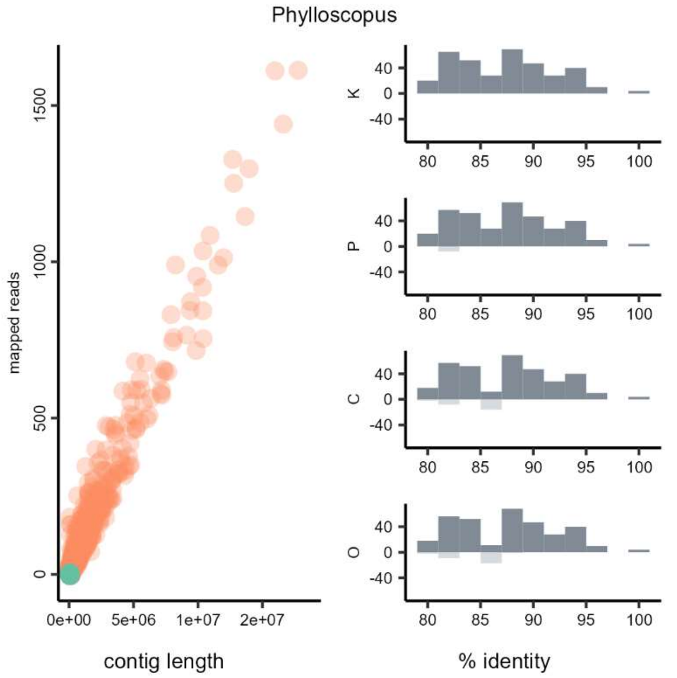

Figure 16. Left: number of mapped reads *vs*. contig length for contigs > 50 kb. Right: mirrored histograms of the sequence identity of aligned reads (ARs) with BLAST best-scoring sequence pairs (BSPs) within (top) or outside (bottom) the kingdom (K), phylum (P), class (C), and order (O) ranks of *Phylloscopus*. BSPs are shown for the 100 consensus sequences with the highest depth of coverage. ARs are the number of reads covering a given consensus sequence query and are given as *n*-fold differences.

***Phylloscopus*; Chordata; Aves; Passeriformes; Phylloscopidae (9181)**

Label: positive
Predicted probability: 0.92
Mapped reads: 137,092
Mapping rate: 64%
Base pairs queried: 59,836
Unique contigs queried: 153
Unique contigs aligned: 62

A nuclear genome assembly was available for *Phylloscopus trochilus* (998.8 Mb, N50: 3.4 Mb), which is the only species in the genus that breeds in the interior of Sweden.

The mapping rate of Phylloscopus-classified reads back to their reference sequences (64%) was lower than the average of 74% (σ = 25%) for all 57 genera analyzed here. However, the number of mapped reads per contig was very strongly correlated (r = 0.98) with contig length, as expected for a true-positive taxon (Fig. 17).

Most consensus queries, and 91% of ARs, had BSPs within Passeriformes (Table 23). At the family rank, ARs were split across six families, with the largest fraction corresponding to BSPs within the Sylviidae family. *Phylloscopus* was previously part of a larger Sylviidae family (Alström, et al. 2006). We excluded self-matches from the BLAST results, but the few remaining BSPs within Phylloscopidae had the highest sequence identities and alignment lengths.

Table 23. Distribution of BLAST best-scoring sequence pairs (BSPs) for the 100 consensus sequences with the highest depth of coverage by taxonomic rank. Aligned reads (ARs) are the number of reads that contributed to a given consensus sequence query. %ARs are scaled by the sum of reads comprising queries with at least one BSP. Results are shown for taxonomic ranks comprising >5% of ARs and the hierarchy is collapsed to the lowest rank with identical results. %ID is mean percent sequence identity, length is the mean alignment length, e-value is the mean expect value, and bitscore is the mean bitscore.

kingdom	phylum	class	order	family	%ARs	%ID	length	e-value	bitscore
Metazoa					100	87	313	7^{-29}	381
Metazoa	Chordata				98	87	314	8^{-29}	383
Metazoa	Chordata	Aves			94	87	315	8^{-29}	384
Metazoa	Chordata	Aves	Passeriformes		91	87	327	3^{-31}	403
Metazoa	Chordata	Aves	Passeriformes	Sylviidae	24	86	383	1^{-30}	444

Metazoa	Chordata	Aves	Passeriformes	Fringillidae	21	88	317	3^{-42}	408
Metazoa	Chordata	Aves	Passeriformes	Cinclidae	17	86	319	5^{-34}	362
Metazoa	Chordata	Aves	Passeriformes	Turdidae	14	89	250	3^{-43}	330
Metazoa	Chordata	Aves	Passeriformes	Phylloscopidae	6	92	715	0.0	1,045

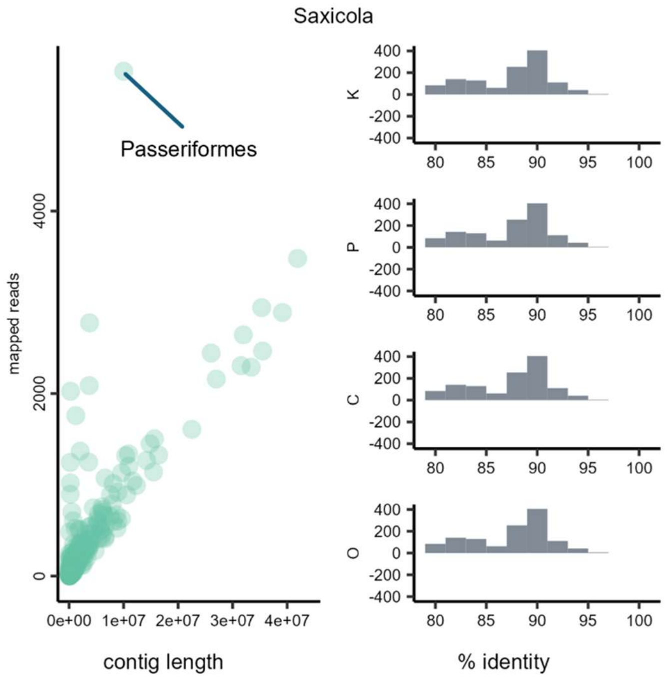

Figure 17. Left: number of mapped reads *vs*. contig length for contigs > 50 kb. Right: mirrored histograms of the sequence identity of aligned reads (ARs) with BLAST best-scoring sequence pairs (BSPs) within (top) or outside (bottom) the kingdom (K), phylum (P), class (C), and order (O) ranks of *Saxicola*. BSPs are shown for the 100 consensus sequences with the highest depth of coverage. ARs are the number of reads covering a given consensus sequence query and are given as *n*-fold differences.

***Saxicola*; Chordata; Aves; Passeriformes; Muscicapidae (69509)**

Label: positive
Predicted probability: 0.83
Mapped reads: 132,996
Mapping rate: 73%
Base pairs queried: 50,013
Unique contigs queried: 52
Unique contigs aligned: 41

A nuclear genome assembly from *Saxicola maurus* (939.3 Mb, N50: 10.0 Mb), was included in the Kraken 2 reference database. *S. rubetra* breeds in northern Fennoscandia and vagrants from other species are documented.

73% of *Saxicola*-classified reads mapped back to their reference database sequences. The number of mapped reads per contig was positively correlated with contig length ($r = 0.80$), although this relationship was noisier among shorter contigs (Fig. 18).

BSPs for all consensus blast queries were exclusively within the Passeriformes order (Table 24). At the family rank, most BSPs were within Turdidae. This pattern was also seen in *Ficedula*, another flycatcher in the Muscicapidae family. Both were previously grouped in Turdidae and work to define reciprocally monophyletic families is ongoing (Sangster et al. 2010), which likely explains the taxonomic distribution of the BSPs. Additional queries (a total of 11,816 bp) from the contig with the greatest number of mapped reads (n = 5,533) had BSPs with *Erithacus*, *Catharus* (Turdidae) and *Cinclus* (Cinclidae) genomes produced by three different labs.

Table 24. Distribution of BLAST best-scoring sequence pairs (BSPs) for the 100 consensus sequences with the highest depth of coverage by taxonomic rank. Aligned reads (ARs) are the number of reads that contributed to a given consensus sequence query. %ARs are scaled by the sum of reads comprising queries with at least one BSP. Results are shown for taxonomic ranks comprising >5% of ARs and the hierarchy is collapsed to the lowest rank with identical results. %ID is mean percent sequence identity, length is the mean alignment length, e-value is the mean expect value, and bitscore is the mean bitscore.

kingdom	phylum	class	order	family	%ARs	%ID	length	e-value	bitscore

Metazoa	Chordata	Aves	Passeriformes		100	87	480	7^{-32}	615
Metazoa	Chordata	Aves	Passeriformes	Turdidae	60	89	442	1^{-31}	589
Metazoa	Chordata	Aves	Passeriformes	Fringillidae	15	89	964	3^{-71}	1,354
Metazoa	Chordata	Aves	Passeriformes	Cinclidae	10	84	411	6^{-40}	435
Metazoa	Chordata	Aves	Passeriformes	Muscicapidae	9	89	406	3^{-64}	539

Other vertebrates

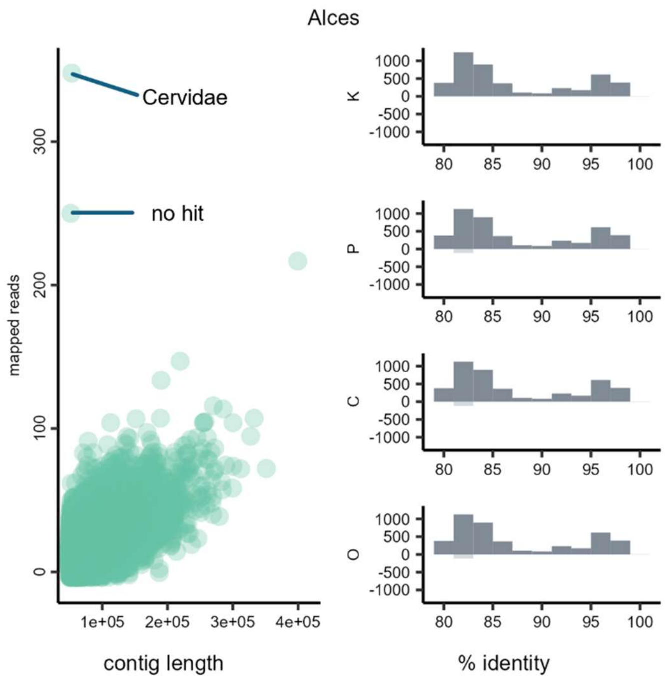

Figure 18. Left: number of mapped reads *vs*. contig length for contigs > 50 kb. Right: mirrored histograms of the sequence identity of aligned reads (ARs) with BLAST best-scoring sequence pairs (BSPs) within (top) or outside (bottom) the kingdom (K), phylum (P), class (C), and order (O) ranks of *Alces*. BSPs are shown for the 100 consensus sequences with the highest depth of coverage. ARs are the number of reads covering a given consensus sequence query and are given as *n*-fold differences.

Alces; Chordata; Mammalia; Artiodactyla; Cervidae (9851)

Label: positive
Predicted probability: 0.93
Mapped reads: 1,103,906
Mapping rate: 91%
Base pairs queried: 68,370
Unique contigs queried: 92
Unique contigs aligned: 81

A nuclear genome assembly for *Alces alces* (2.7 Gb, N50: 45 kb) was included in the Kraken 2 reference database. *A. alces* is the only species in the genus and is a common inhabitant of subalpine forests throughout Fennoscandia.

Most (91%) of *Alces*-classified reads could be mapped back to their sequences in the reference database. Although the contiguity of the *Alces* assembly was low, contig length and the number of mapped reads were positively correlated (r = 0.80) (Fig. 19).

We called consensus sequences from the 100 regions with the highest read depth and queried these against the BLAST nt database. One query had BSPs outside of the Cervidae family (Table 25). This sequence aligned with a polychaete worm and may have resulted in the misclassification of 3% of the aligned reads. Within Cervidae, 42% of ARs were aligned with *Cervus*, 37% with Alces, and 10% with *Muntiacus*. When restricted to the single best-scoring sequence pair query, 92% of ARs were with *Alces*.

Table 25. Distribution of BLAST best-scoring sequence pairs (BSPs) for the 100 consensus sequences with the highest depth of coverage by taxonomic rank. Aligned reads (ARs) are the number of reads that contributed to a given consensus sequence query. %ARs are scaled by the sum of reads comprising queries with at least one BSP. Results are shown for taxonomic ranks comprising >5% of ARs and the hierarchy is collapsed to the lowest rank with identical results. %ID is mean percent sequence identity, length is the mean alignment length, e-value is the mean expect value, and bitscore is the mean bitscore.

kingdom	phylum	class	order	family	%ARs	%ID	length	e-value	bitscore
Metazoa					100	87	465	2^{-40}	529
Metazoa	Chordata	Mammalia	Artiodactyla	Cervidae	97	87	469	2^{-40}	534

Alces was used to compare the results from simply querying reads against the nt database *vs*. the consensus sequence approach we applied to all genera for validation. Of the 100 randomly selected paired-end reads, 100% of the aligned reads were within the Artiodactyla order but a smaller proportion (91%) aligned within the Cervidae compared to the consensus sequences (Table 26). This difference is due to a larger proportion reads aligning within the Bovidae (5%) and Delphinidae (2%) families. While sequence identity with Delphinidae was low (82%) and could result from conserved homologous regions, reads aligned specifically with *Bos* (Bovidae) with relatively high identity (92%). *Bos* DNA has been found in reagent kits (Leonard et al. 2007) and we cannot exclude the possibility that some *Alces*-classified reads originated from *Bos* Therefore, we estimate the misclassification rate for *Alces* reads to between 3-10% and consider *Alces* a true positive genus.

Table 26. Distribution of BLAST best-scoring sequence pairs (BSPs) by taxonomic rank for 100 randomly-selected pair-end reads. Results are shown for taxonomic ranks comprising >5% of reads and the hierarchy is collapsed to the lowest rank with identical results. %ID is mean percent sequence identity, length is the mean alignment length, e-value is the mean expect value, and bitscore is the mean bitscore.

kingdom	phylum	class	order	family	%BSPs	%ID	length	e-value	bitscore
Metazoa	Chordata	Mammalia	Artiodactyla		100	94	116	2^{-27}	178
Metazoa	Chordata	Mammalia	Artiodactyla	Cervidae	91	95	115	1^{-27}	180

***Gadus*; Chordata; Actinopteri; Gadiformes; Gadidae (8048)**

Label: unlabeled
Predicted probability: 0.99
Mapped reads: 494,992
Mapping rate: 86%
Base pairs queried: 1,952,637
Unique contigs queried: 46
Unique contigs aligned: 36

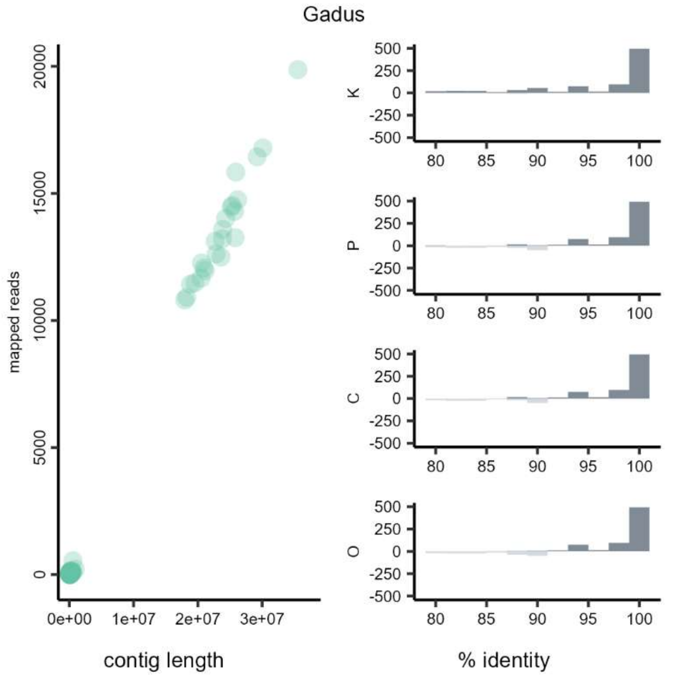

Figure 19. Left: number of mapped reads *vs*. contig length for contigs > 50 kb. Right: mirrored histograms of the sequence identity of aligned reads (ARs) with BLAST best-scoring sequence pairs (BSPs) within (top) or outside (bottom) the kingdom (K), phylum (P), class (C), and order (O) ranks of *Gadus*. BSPs are shown for the 100 consensus sequences with the highest depth of coverage. ARs are the number of reads covering a given consensus sequence query and are given as *n*-fold differences.

We flagged *Gadus* for validation because we used this genus of marine fish to give a first estimate of the potential limits of vertebrate airborne eDNA dispersal. A chromosome-scale (548.1 Mb; N50: 23.9 Mb) and a partial assembly (14.1 Mb, N50: 110 kb) of *G. morhua*, were included in the Kraken 2 reference database. This is the species expected to be present in the Atlantic ocean.

Most (86%) *Gadus*-classified reads could be mapped back to their sequences in the reference database. The number of mapped reads per contig was strongly correlated ($r = 0.999$) with contig length (Fig. 20), making *Gadus* an exceptionally good example of the expected pattern for a true positive.

Most consensus sequences had BSPs exclusively within the Gadidae family, corresponding to 85% of the queried reads (Table 27). However, nine consensus queries, all from short transcriptome sequences (< 10 kb), aligned with insects. We extended the BLAST search to the next 100 sequences with the highest coverage and found no additional BSPs with taxa outside of Actinopteri and all but one were within the Gadidae family. The exception originated from a chromosome-length scaffold (LR633957.1) with BSPs with taxa in the Pleuronectiformes order.

Table 27. Distribution of BLAST best-scoring sequence pairs (BSPs) for the 100 consensus sequences with the highest depth of coverage by taxonomic rank. Aligned reads (ARs) are the number of reads that contributed to a given consensus sequence query. %ARs are scaled by the sum of reads comprising queries with at least one BSP. Results are shown for taxonomic ranks comprising >5% of ARs and the hierarchy is collapsed to the lowest rank with identical results. %ID is mean percent sequence identity, length is the mean alignment length, e-value is the mean expect value, and bitscore is the mean bitscore.

kingdom	phylum	class	order	family	%ARs	%ID	length	e-value	bitscore
Metazoa					99	94	398	5^{-39}	642
Metazoa	Chordata				87	95	430	1^{-50}	706
Metazoa	Chordata	Actinopteri			86	95	431	1^{-50}	708
Metazoa	Chordata	Actinopteri	Gadiformes	Gadidae	85	96	437	8^{-51}	721
Metazoa	Arthropoda	Insecta			12	85	185	4^{-38}	215

Metazoa	Arthropoda	Insecta	Diptera		8	85	183	7^{-38}	212

We conducted a BLAST search using 684,987 bp of consensus sequences from LR633957.1 in non-overlapping 3,000 bp windows. In addition, we queried 1.23 Mb of consensus sequences from two randomly selected chromosome-sized scaffolds (LR633961.1 and LR633944.1). All BSPs were within Actinopteri and 94-98% of ARs from the three scaffolds aligned within the Gadidae family (Table 28). BSPs with 29 other fish families accounted for the remaining ARs, but none individually amounted to more than 1.6% of ARs. Limiting the BLAST results to the single best-scoring sequence pair per query reduced the non-Gadidae hits to 0-3% of ARs. This, combined with the rarity of non-Gadidae BSPs and their diffuse taxonomic distribution among other fish clades, are more consistent with relatively conserved sequences than contamination in the chromosome-length reference assembly. Overall, 1,542 out of the 60,526 investigated *Gadus*-classified reads (2.5%) may be spurious due to putative contamination.

Table 28. Distribution of BLAST best-scoring sequence pairs (BSPs) for consensus queries from three chromosome-length depth of coverage by taxonomic rank. Aligned reads (ARs) are the number of reads that contributed to a given consensus sequence query. %ARs are scaled by the sum of reads comprising queries with at least one BSP. Results are shown for taxonomic ranks comprising >1% ARs and the hierarchy is collapsed to the lowest rank with identical results. %ID is mean percent sequence identity, length is the mean alignment length, e-value is the mean expect value, and bitscore is the mean bitscore.

kingdom	phylum	class	order	family	%ARs	%ID	length	e-value	bitscore
				LR633957.1					
Metazoa	Chordata	Actinopteri			100.00	95	206	1^{-28}	321
Metazoa	Chordata	Actinopteri	Gadiformes	Gadidae	94.10%	95	207	5^{-34}	326
Metazoa	Chordata	Actinopteri	Cypriniformes		1.56	86	171	7^{-27}	167
Metazoa	Chordata	Actinopteri	Perciformes		1.49	93	182	4^{-29}	267
				LR633961.1					
Metazoa	Chordata	Actinopteri			100.00	94	194	4^{-29}	296
Metazoa	Chordata	Actinopteri	Gadiformes	Gadidae	98.18	94	194	4^{-29}	296
				LR633944.1					
Metazoa	Chordata	Actinopteri			100.00	95	207	1^{-30}	328
Metazoa	Chordata	Actinopteri	Gadiformes		97.33	95	208	1^{-30}	330
Metazoa	Chordata	Actinopteri	Gadiformes	Gadidae	96.79	95	208	1^{-30}	329
Metazoa	Chordata	Actinopteri	Perciformes		1.07	93	205	2^{-65}	290

Finally, *Gadus* was also used to compare the results of the two BLAST approaches we initially considered. Out of 100 randomly selected paired-end reads, 99% of the alignments were within Gadidae, with 98% sequence identity (Table 29). Both approaches therefore indicate a low misclassification rate for *Gadus*, from 1% to the 2.5% estimated from the combined evidence of the consensus sequences.

Table 29. Distribution of BLAST best-scoring sequence pairs (BSPs) by taxonomic rank for 100 randomly-selected pair-end reads. Results are shown for taxonomic ranks comprising >5% of reads and the hierarchy is collapsed to the lowest rank with identical results. %ID is mean percent sequence identity, length is the mean alignment length, e-value is the mean expect value, and bitscore is the mean bitscore.

kingdom	phylum	class	order	family	%BSPs	%ID	length	e-value	bitscore

Metazoa					100	97	122	1^{-27}	207
Metazoa	Chordata	Actinopteri	Gadiformes	Gadidae	99	98	123	6^{-28}	207

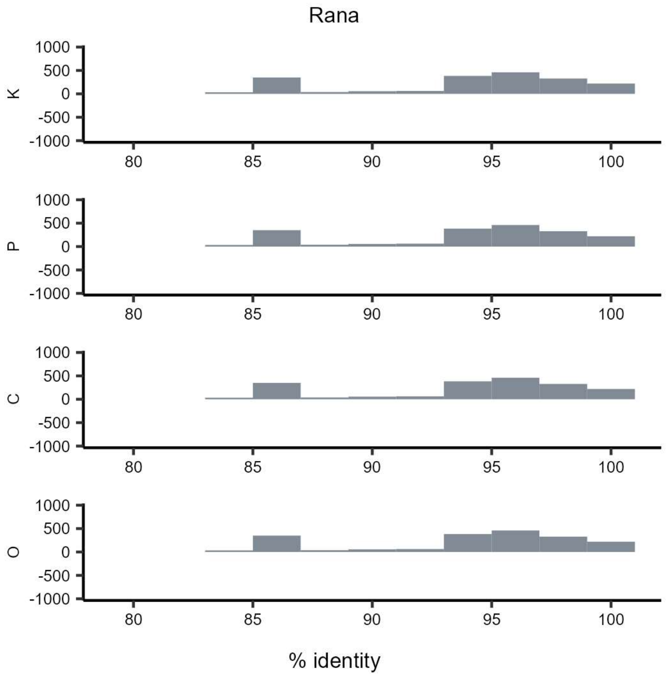

Figure 20. Mirrored histograms of the sequence identity of aligned reads (ARs) with BLAST best-scoring sequence pairs (BSPs) within (top) or outside (bottom) the kingdom (K), phylum (P), class (C), and order (O) ranks of *Rana*. BSPs are shown for the 100 consensus sequences with the highest depth of coverage. ARs are the number of reads covering a given consensus sequence query and are given as *n*-fold differences.

Rana; Chordata; Amphibia; Anura; Ranidae (8399)

Label: positive
Predicted probability: 0.99
Mapped reads: 1,174
Mapping rate: 69%
Base pairs queried: 13,242
Unique contigs queried: 86
Unique contigs aligned: 54

No nuclear genome assemblies for *Rana* were available when we constructed the Kraken 2 reference database. At that time, the longest available reference sequence was a mitochondrial genome and the median length of sequences in the Kraken 2 database was 494 bp. 69% of the *Rana*-classified reads mapped back to their refence sequences, but we did not investigate the correlation between contig length and mapped read count.

Rana-classified reads were not abundant in our dataset, but despite this, consensus queries had BSPs exclusively with anurans, and 98% of ARs aligned within the Ranidae family (Fig. 21, Table 30). This, combined with the high sequence identity with Randidae sequences, support *Rana* as a true positive genus.

Table 30. Distribution of BLAST best-scoring sequence pairs (BSPs) for the 100 consensus sequences with the highest depth of coverage by taxonomic rank. Aligned reads (ARs) are the number of reads that contributed to a given consensus sequence query. %ARs are scaled by the sum of reads comprising queries with at least one BSP. Results are shown for taxonomic ranks comprising >5% of ARs and the hierarchy is collapsed to the lowest rank with identical results. %ID is mean percent sequence identity, length is the mean alignment length, e-value is the mean expect value, and bitscore is the mean bitscore.

kingdom	phylum	class	order	family	%ARs	%ID	length	e-value	bitscore
Metazoa	Chordata	Amphibia	Anura		100	94	215	1^{-49}	338
Metazoa	Chordata	Amphibia	Anura	Ranidae	98	95	215	2^{-49}	339

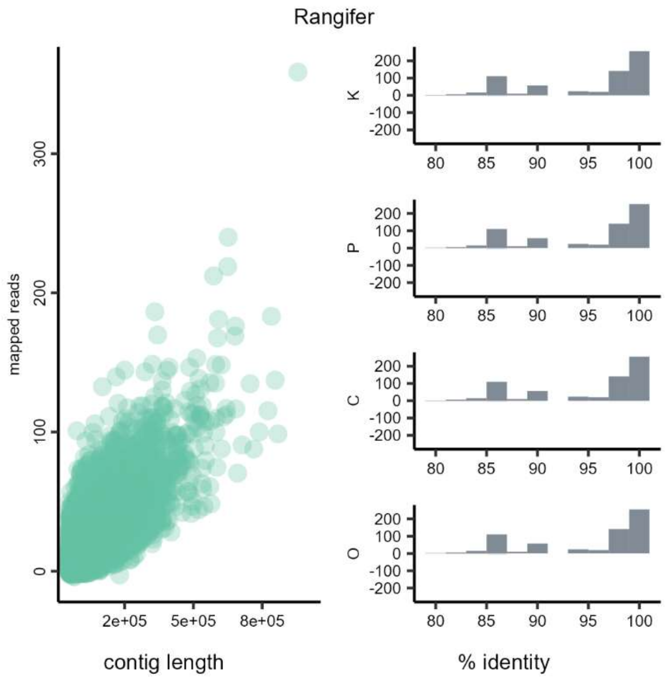

Figure 21. Left: number of mapped reads *vs*. contig length for contigs > 50 kb. Right: mirrored histograms of the sequence identity of aligned reads (ARs) with BLAST best-scoring sequence pairs (BSPs) within (top) or outside (bottom) the kingdom (K), phylum (P), class (C), and order (O) ranks of *Rangifer*. BSPs are shown for the 100 consensus sequences with the highest depth of coverage. ARs are the number of reads covering a given consensus sequence query and are given as *n*-fold differences.

***Rangifer*; Chordata; Mammalia; Artiodactyla; Cervidae (9869)**

Label: positive
Predicted probability: 0.87
Mapped reads: 551,500
Mapping rate: 93%
Base pairs queried: 26,770
Unique contigs queried: 88
Unique contigs aligned: 81

A nuclear genome assembly from *Rangifer tarandus* (2.8 Gb, N50: 86 kb), was included in the Kraken 2 reference database. Most (93%) reads could be mapped back to their sequences in the reference database. Contig length and mapped read count were positively correlated ($r = 0.74$), the pattern expected from a positive genus (Fig. 22).

We called consensus sequences from the 100 regions with the highest read depth and queried these against the BLAST nt database. Most best-scoring sequence pairs (BSPs) for these queries were within Artiodactyla and most were furthermore within the Cervidae family (Table 31). At the genus rank, 85% of ARs were with *Rangifer* sequences that were not included in the Kraken 2 reference database. Cow (*Bos*) DNA is a common kit contaminant, and 9% of ARs were within Bovidae. However, all queries with a bovid BSP also had cervid BSPs, and the sequence identity with bovine BSPs were much lower (84% *vs*. 97%) on average. This suggests the BSPs within Bovidae resulted from homology rather than contamination.

Table 31. Distribution of BLAST best-scoring sequence pairs (BSPs) for the 100 consensus sequences with the highest depth of coverage by taxonomic rank. Aligned reads (ARs) are the number of reads that contributed to a given consensus sequence query. %ARs are scaled by the sum of reads comprising queries with at least one BSP. Results are shown for taxonomic ranks comprising >5% of ARs and the hierarchy is collapsed to the lowest rank with identical results. %ID is mean percent sequence identity, length is the mean alignment length, e-value is the mean expect value, and bitscore is the mean bitscore.

kingdom	phylum	class	order	family	%ARs	%ID	length	e-value	bitscore
Metazoa					98	97	282	1^{-40}	454
Metazoa	Chordata	Mammalia	Artiodactyla		97	97	283	6^{-71}	456
Metazoa	Chordata	Mammalia	Artiodactyla	Cervidae	88	97	278	6^{-71}	454
Metazoa	Chordata	Mammalia	Artiodactyla	Bovidae	9	84	538	1^{-83}	595

Salmo; Metazoa; Chordata; Actinopteri; Salmoniformes; Salmonidae (8028)

Label: positive
Predicted probability: 0.89
Mapped reads: 411,164
Mapping rate: 76%
Base pairs queried: 40,073
Unique contigs queried: 63
Unique contigs aligned: 37

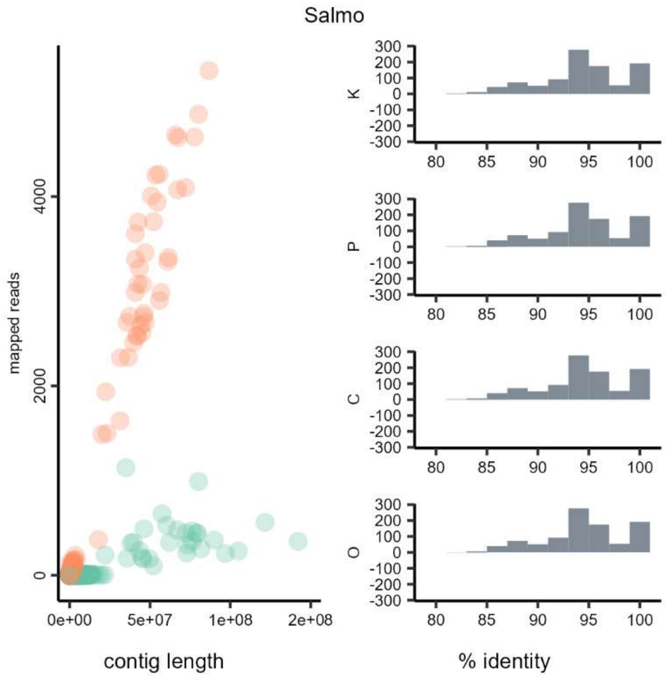

Figure 22. Left: number of mapped reads *vs*. contig length for contigs > 50 kb. Right: mirrored histograms of the sequence identity of aligned reads (ARs) with BLAST best-scoring sequence pairs (BSPs) within (top) or outside (bottom) the kingdom (K), phylum (P), class (C), and order (O) ranks of *Salmo*. BSPs are shown for the 100 consensus sequences with the highest depth of coverage. ARs are the number of reads covering a given consensus sequence query and are given as *n*-fold differences.

A nuclear genome assembly for *Salmo trutta* (1.7 Gb, N50: 568 kb) and *S. salar* (2.0 Gb, N50: 76.2 Mb) were included in the Kraken 2 reference database. More reads mapped to *S. trutta*, in orange, than *S. salar*, in green. Within each assembly, the number of reads mapped to a contig was correlated with contig length (*S. salar* $r = 0.80$, *S. trutta* $r = 0.99$), the pattern expected for true positives (Fig. 23).

98% of ARs corresponding to the 100 highest-depth consensus sequences aligned within the Salmonidae family (Table 32). 96% were furthermore aligned with *Salmo* specifically, with *Coregonus* accounting for the remaining 2%.

Table 32. Distribution of BLAST best-scoring sequence pairs (BSPs) for the 100 consensus sequences with the highest depth of coverage by taxonomic rank. Aligned reads (ARs) are the number of reads that contributed to a given consensus sequence query. %ARs are scaled by the sum of reads comprising queries with at least one BSP. Results are shown for taxonomic ranks comprising >5% of ARs and the hierarchy is collapsed to the lowest rank with identical results. %ID is mean percent sequence identity, length is the mean alignment length, e-value is the mean expect value, and bitscore is the mean bitscore.

kingdom	phylum	class	order	family	%ARs	%ID	length	e-value	bitscore
Metazoa					100	94	497	1^{-37}	772
Metazoa	Chordata	Actinopteri			99	94	520	2^{-39}	813
Metazoa	Chordata	Actinopteri	Salmoniformes	Salmonidae	98	94	522	2^{-46}	818

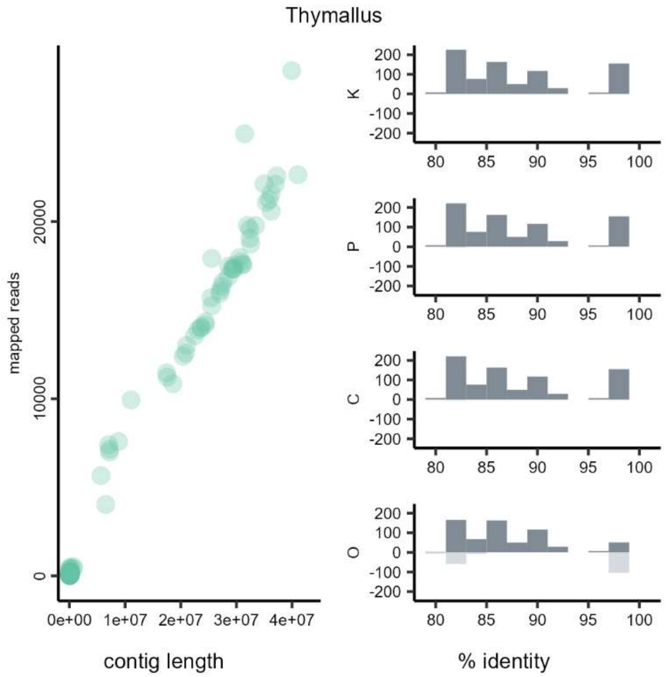

Figure 23. Left: number of mapped reads *vs*. contig length for contigs > 50 kb. Right: mirrored histograms of the sequence identity of aligned reads (ARs) with BLAST best-scoring sequence pairs (BSPs) within (top) or outside (bottom) the kingdom (K), phylum (P), class (C), and order (O) ranks of *Thymallus*. BSPs are shown for the 100 consensus sequences with the highest depth of coverage. ARs are the number of reads covering a given consensus sequence query and are given as *n*-fold differences.

Thymallus; Chordata; Actinopteri; Salmoniformes; Salmonidae (36184)

Label: unlabeled
Predicted probability: 0.98
Mapped reads: 1,019,770 (89%)
Base pairs queried: 3,667,056
Unique contigs queried: 52
Unique contigs aligned: 32

Thymallus was not included as a positive training taxon but is common in rivers near the aerosol monitoring station. We selected this taxon for validation because of the abundance of *Thymallus*-classified reads and the significance of detecting aquatic vertebrates in airborne eDNA.

A nuclear genome assembly for the European grayling, *Thymallus thymallus* (1.4 Gb, N50: 29.7 Mb), was included in the Kraken 2 reference database. 89% of *Thymallus*-classified reads mapped back to their reference sequences and the number of mapped reads per contig was strongly correlated ($r = 0.99$) with contig length (Fig. 24).

In the initial blast query, 99% of ARs had their best-scoring alignments within Acintopteri and 79% within the Salmonidae family (Table 33). *Thymallus* was not well-represented in the nt database and most of these alignments were with other salmonid genera, which likely explains the lower mean sequence identity compared to *Salmo*. However, salmonids in general are well-sequenced, and alignments within Cypriniformes and Argentiniformes were not seen in *Salmo*. These queries originated from five chromosome-sized scaffolds and two smaller contigs (34 and 43 kb).

Table 33. Distribution of BLAST best-scoring sequence pairs (BSPs) for the 100 consensus sequences with the highest depth of coverage by taxonomic rank. Aligned reads (ARs) are the number of reads that contributed to a given consensus sequence query. %ARs are scaled by the sum of reads comprising queries with at least one BSP. Results are shown for taxonomic ranks comprising >5% of ARs and the hierarchy is collapsed to the lowest rank with identical results. %ID is mean percent sequence identity, length is the mean alignment length, e-value is the mean expect value, and bitscore is the mean bitscore.

kingdom	phylum	class	order	family	%ARs	%ID	length	e-value	bitscore
Metazoa					100	87	502	8E-35	596
Metazoa	Chordata	Actinopteri			99	87	508	8E-35	605
Metazoa	Chordata	Actinopteri	Salmoniformes	Salmonidae	79	87	533	8E-39	637
Metazoa	Chordata	Actinopteri	Cypriniformes	Nemacheilidae	12	97	654	0E+00	1,097
Metazoa	Chordata	Actinopteri	Argentiniformes	Argentinidae	6	81	1,168	0E+00	1,119

We queried an additional 3,569,467 bp of consensus sequences in non-overlapping 3,000 bp segments from the five chromosome-sized scaffolds (QMII01003786, QMII01003816, QMII01003819, and QMII01003826). BSPs with queries from four of these scaffolds were exclusively within the Salmonidae (Table 34). Twenty-three of the 351 queries from QMII01003786 had BSPs within Cypriniformes and Argentiniformes, or about 6% of ARs. These results do not clearly implicate reference contamination but provide a conservative estimate for the misclassification rate of *Thymallus*-classified reads.

Table 34. Distribution of BLAST best-scoring sequence pairs (BSPs) by taxonomic rank for consensus sequences from five chromosome-size scaffolds from the *Thymallus thymallus* reference genome. Aligned reads (ARs) are the number of reads that contributed to a given consensus sequence query. %ARs are scaled by the sum of reads comprising queries with at least one BSP. Results are shown for taxonomic ranks comprising >5% of ARs and the hierarchy is collapsed to the lowest rank with identical results. %ID is mean percent sequence identity, length is the mean alignment length, e-value is the mean expect value, and bitscore is the mean bitscore.

kingdom	phylum	class	order	family	%ARs	%ID	length	e-value	bitscore
Metazoa	Chordata	Actinopteri	Salmoniformes	Salmonidae	92	91	215	1^{-28}	274
Metazoa	Chordata	Actinopteri	Cypriniformes		6	93	254	5^{-34}	371

Insects

Aedes; Arthropoda; Insecta; Diptera; Culicidae (7158)

Label: positive
Predicted probability: 0.83
Mapped reads: 12,285,472
Mapping rate: 44%
Base pairs queried: 78,690
Unique contigs queried: 51
Unique contigs aligned: 47

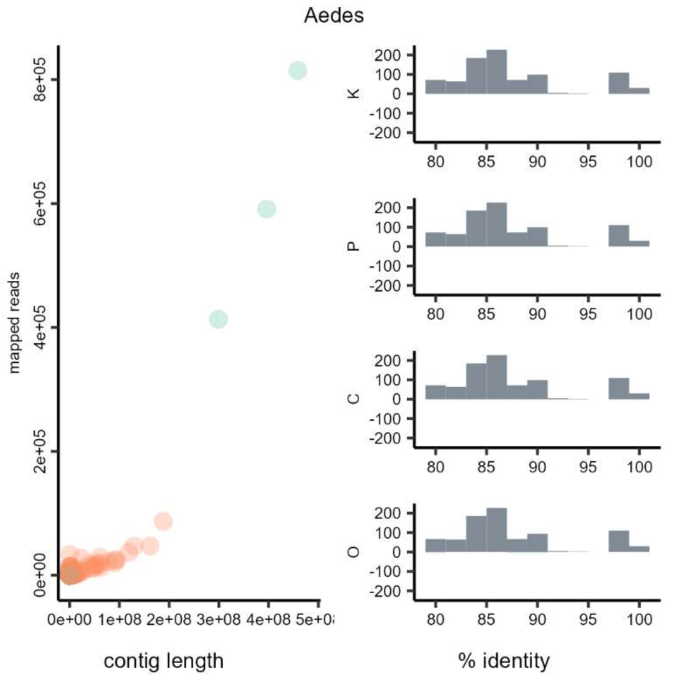

Figure 24. Left: number of mapped reads *vs*. contig length for contigs > 50 kb. Right: mirrored histograms of the sequence identity of aligned reads (ARs) with BLAST best-scoring sequence pairs (BSPs) within (top) or outside (bottom) the kingdom (K), phylum (P), class (C), and order (O) ranks of *Aedes*. BSPs are shown for the 100 consensus sequences with the highest depth of coverage. ARs are the number of reads covering a given consensus sequence query and are given as *n*-fold differences.

Seven *Aedes* species have been documented in Norrbotten province, six in subgenus *Ochlerotatus* and one in subgenus *Aedes* (Lundström et al. 2013). *Ochelerotatus* is considered its own genus by some authors (cf. Reinert 2000; Reisen 2016) and both uses of the name have been applied to sequences in the NCBI databases.

Assemblies for *A. aegypti* (1.2 Gb, N50: 396.3 Mb) and *A. albopictus* (2.5 Gb, N50: 333 kb) were included in the Kraken 2 reference database. A relatively low fraction (44%) of *Aedes*-classified reads mapped to their reference sequences. Of the mapped reads, 50% mapped to the *A. albopictus* assembly, in orange , and 45% to the chromosome-level *A. aegypti* assembly, in green in Fig. 25. More reads mapped to longer contigs (r = 0.99), as expected in a true positive genus.

We called consensus sequences from the 100 regions with the highest read depth and queried these against the BLAST nt database. Almost all best-scoring sequence pairs (BSPs) aligned within the Culicidae family (Table 35). At the genus-rank, ARs were distributed among *Wyeomyia* (36%), *Ochlerotatus* (27%), and *Aedes* (27%). The large share of reads aligned with *Wyeomyia* was unexpected, as this genus is not closely related to *Aedes* (Lorenz et al. 2021). However, this result appears due to the number of BSPs considered and the taxonomic composition of the nt database. Restricted to the single best BSP per query, 88% of ARs were with *Ochelerotatus* (43%) or *Aedes* (35%), with 12% aligned with *Wyeomyia*.

Table 35. Distribution of BLAST best-scoring sequence pairs (BSPs) for the 100 consensus sequences with the highest depth of coverage by taxonomic rank. Aligned reads (ARs) are the number of reads that contributed to a given consensus sequence query. %ARs are scaled by the sum of reads comprising queries with at least one BSP. Results are shown for taxonomic ranks comprising >5% of ARs and the hierarchy is collapsed to the lowest rank with identical results. %ID is

mean percent sequence identity, length is the mean alignment length, e-value is the mean expect value, and bitscore is the mean bitscore.

kingdom	phylum	class	order	family	%ARs	%ID	length	e-value	bitscore
Metazoa	Arthropoda	Insecta			100	87	332	8^{-38}	417
Metazoa	Arthropoda	Insecta	Diptera		98	87	342	8-^{38}	431
Metazoa	Arthropoda	Insecta	Diptera	Culicidae	97	88	335	9^{-38}	425

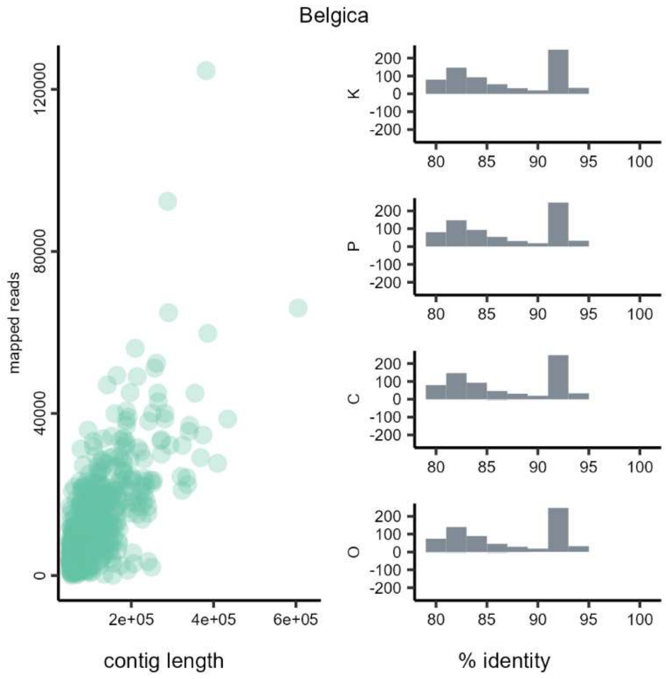

Figure 25. Left: number of mapped reads *vs*. contig length for contigs > 50 kb. Right: mirrored histograms of the sequence identity of aligned reads (ARs) with BLAST best-scoring sequence pairs (BSPs) within (top) or outside (bottom) the kingdom (K), phylum (P), class (C), and order (O) ranks of *Belgica*. BSPs are shown for the 100 consensus sequences with the highest depth of coverage. ARs are the number of reads covering a given consensus sequence query and are given as *n*-fold differences.

Belgica; Arthropoda; Insecta; Diptera; Chironomidae (315555)

Label: unlabeled
Predicted probability: 0.82
Mapped reads: 9,719,924
Mapping rate: 86%
Base pairs queried: 1,357,575
Unique contigs queried: 87
Unique contigs aligned: 83

We flagged *Belgica* for further validation because this genus was abundant among the classified reads, yet is a flightless insect endemic to Antarctica. We mapped *Belgica*-classified reads from weeks 1974:26, 1976:39, 1978:27, 1980:23, 1982:27, 1984:26, 1986:28, 1988:30, 1990:24, 1992:27, 1994:31, 1996:31, 1998:32, 2000:30, 2002:27, 2004:34, 2006:23, and 2008:35 to their Kraken 2 reference sequences (89.6 Mb, N50: 14 kb).

Most of the *Belgica*-classified reads (86%) mapped to their sequences in the reference database. We found a positive correlation ($r = 0.70$) between contig length and the number of mapped reads (Fig. 26), as observed for almost all other positive taxa.

We called consensus sequences from the 100 regions with the highest read depth and queried these against the BLAST nt database. Best-scoring sequence pairs (BSPs) for these queries were almost entirely within Diptera (Table 36). We scaled the consensus queries by their read depth to obtain the number of reads represented by each alignment, and 89% of these aligned reads (ARs) were within the Chironomidae family.

Table 36. Distribution of BLAST best-scoring sequence pairs (BSPs) for the 100 consensus sequences with the highest depth of coverage by taxonomic rank. Aligned reads (ARs) are the number of reads that contributed to a given consensus sequence query. %ARs are scaled by the sum of reads comprising queries with at least one BSP. Results are shown for taxonomic ranks comprising >5% of ARs and the hierarchy is collapsed to the lowest rank with identical results. %ID is

mean percent sequence identity, length is the mean alignment length, e-value is the mean expect value, and bitscore is the mean bitscore.

kingdom	phylum	class	order	family	%ARs	%ID	length	e-value	bitscore
Metazoa	Arthropoda				100	84	325	4^{-29}	374
Metazoa	Arthropoda	Insecta			99	84	325	4^{-29}	374
Metazoa	Arthropoda	Insecta	Diptera		96	84	323	4^{-29}	374
Metazoa	Arthropoda	Insecta	Diptera	Chironomidae	89	84	328	5^{-29}	379
Metazoa	Arthropoda	Insecta	Diptera	Culicidae	6	85	299	5^{-47}	351

Belgica was one of the genera used to compare the results from directly querying reads *vs.* the consensus sequences approach. Out of 100 randomly selected paired-end reads, 96% of alignments were within Diptera and 89% within Chironomidae (Table 37), which are the same proportions found by the consensus queries. From this combined evidence, we conclude that 'Belgica' most likely originates from a chironomid absent from the reference database. 'Belgica' would then be correctly classified as a positive occurrence, even though the genera-rank assignment is extremely unlikely to be correct.

Table 37. Distribution of BLAST best-scoring sequence pairs (BSPs) by taxonomic rank for 100 randomly-selected pair-end reads. Results are shown for taxonomic ranks comprising >5% of reads and the hierarchy is collapsed to the lowest rank with identical results. %ID is mean percent sequence identity, length is the mean alignment length, e-value is the mean expect value, and bitscore is the mean bitscore.

kingdom	phylum	class	order	family	%BSPs	%ID	length	e-value	bitscore
Metazoa	Arthropoda	Insecta	Diptera		96	91	123	2^{-27}	167
Metazoa	Arthropoda	Insecta	Diptera	Chironomidae	89	91	123	2^{-27}	167

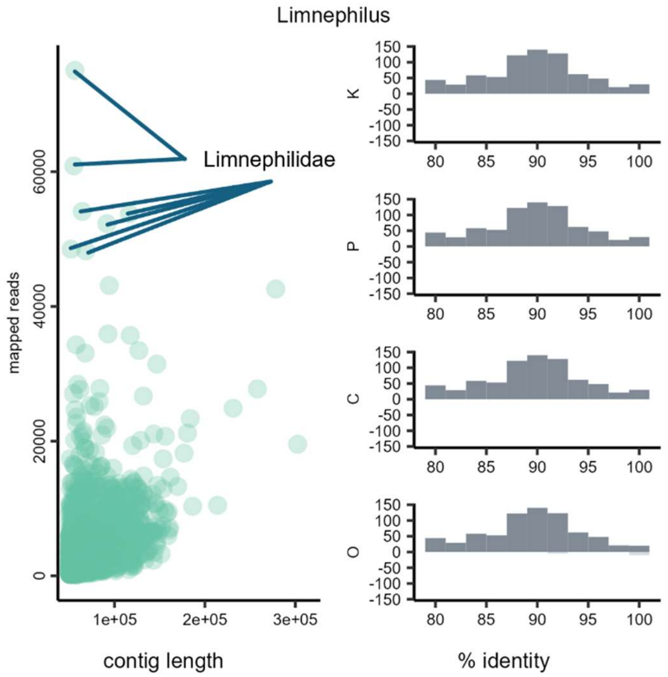

Figure 26. Left: number of mapped reads *vs*. contig length for contigs > 50 kb. Right: mirrored histograms of the sequence identity of aligned reads (ARs) with BLAST best-scoring sequence pairs (BSPs) within (top) or outside (bottom) the kingdom (K), phylum (P), class (C), and order (O) ranks of *Limnephilus*. BSPs are shown for the 100 consensus sequences with the highest depth of coverage. ARs are the number of reads covering a given consensus sequence query and are given as *n*-fold differences.

Limnephilus; Arthropoda; Insecta; Trichoptera; Limnephilidae (177674)

Label: positive
Predicted probability: 0.87
Mapped reads: 109,667,028
Mapping rate: 56%
Base pairs queried: 260,855
Unique contigs queried: 100
Unique contigs aligned: 99

A nuclear genome assembly from *Limnephilus lunatus* (1.2 Gb, N50: 23 kb), was included in the Kraken 2 reference database. Thirty-four *Limnephilus* species have been reported in Norrbotten county, including *L. lunatus*, but *L. femoratus* and *L. borealis* are the most frequently reported.

Relatively few (56%) *Limnephilus*-classified reads mapped back to their sequences in the reference database, compared to the mean 74% mapping rate ($\sigma = 25\%$) for all 57 genera. Contig length and mapped read count were weakly correlated ($r = 0.35$; Fig. 27).

Although the read mapping results did not clearly show the patterns expected for a true positive genus, the BLAST alignments were less ambiguous. We called consensus sequences from the 100 regions with the highest read depth and queried these against nt database. Best-scoring sequence pairs (BSPs) were entirely within Insecta (Table 38). We scaled the consensus queries by their read depth to obtain the number of reads represented by each alignment, and 96% of these aligned reads (ARs) were furthermore within Limnephilidae. This included consensus queries from the cluster of seven contigs with an unexpectedly larger number of mapped reads, as indicated in Fig. 27.

Table 38. Distribution of BLAST best-scoring sequence pairs (BSPs) for the 100 consensus sequences with the highest depth of coverage by taxonomic rank. Aligned reads (ARs) are the number of reads that contributed to a given consensus sequence query. %ARs are scaled by the sum of reads comprising queries with at least one BSP. Results are shown for taxonomic ranks comprising >5% of ARs and the hierarchy is collapsed to the lowest rank with identical results. %ID is mean percent sequence identity, length is the mean alignment length, e-value is the mean expect value, and bitscore is the mean bitscore.

kingdom	phylum	class	order	family	%ARs	%ID	length	e-value	bitscore
Metazoa	Arthropoda	Insecta			100	90	865	1^{-74}	1,206
Metazoa	Arthropoda	Insecta	Trichoptera		98	90	874	1^{-74}	1,215

kingdom	phylum	class	order	family	%ARs	%ID	length	e-value	bitscore
Metazoa	Arthropoda	Insecta	Trichoptera	Limnephilidae	96	89	878	1^{-74}	1,217

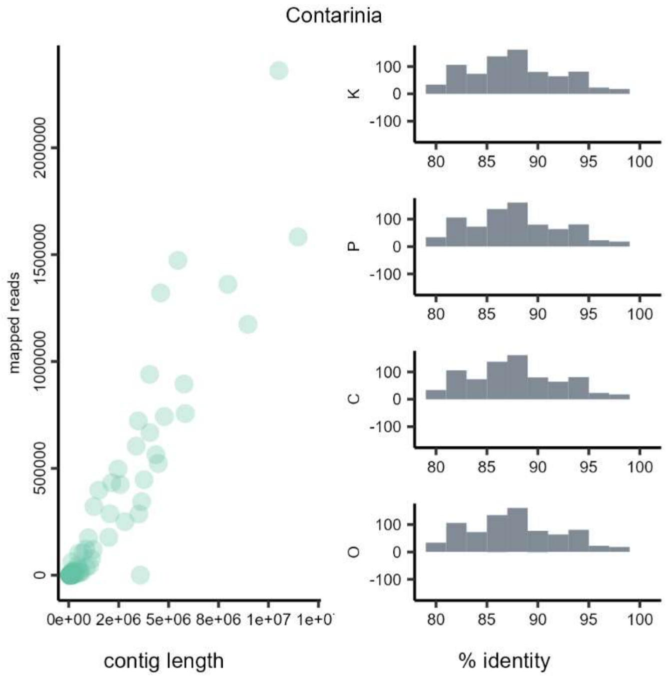

Figure 27. Left: number of mapped reads *vs*. contig length for contigs > 50 kb. Right: mirrored histograms of the sequence identity of aligned reads (ARs) with BLAST best-scoring sequence pairs (BSPs) within (top) or outside (bottom) the kingdom (K), phylum (P), class (C), and order (O) ranks of *Contarinia*. BSPs are shown for the 100 consensus sequences with the highest depth of coverage. ARs are the number of reads covering a given consensus sequence query and are given as *n*-fold differences.

Contarinia; Arthropoda; Insecta; Diptera; Cecidomyiidae (153220)

Label: unlabeled
Predicted probability: 0.76
Mapped reads: 33,096,236
Mapping rate: 54%
Base pairs queried: 1,349,155
Unique contigs queried: 32
Unique contigs aligned: 30

Contarinia was flagged for validation because this genus was abundant among the classified reads but was not reported to occur within 40 km of the filter station between 1974 and 2008. A nuclear genome assembly for *C. nasturtii* (162.2 Mb, N50: 3.8 Mb) was included in the Kraken 2 database.

Relatively few of the *Contarinia*-classified reads (54%) mapped back to their reference database sequences. However, the number of reads mapped per contig was strongly correlated with contig length ($r = 0.94$), the pattern expected from a true positive genus (Fig. 28).

We called consensus sequences from the 100 regions with the highest read depth and queried these against the BLAST nt database. All best-scoring sequence pairs (BSPs) for these queries were exclusively within Insecta (Table 39). We scaled the consensus queries by their read depth to obtain the number of reads represented by each alignment, and 98% of these aligned reads (ARs) were within the Cecidomyiidae family. We also queried 100 randomly-selected paired-end reads against the nt database and found a similar taxonomic distribution of the alignments (Table 40). These results indicate that the *Contarinia*-classified reads most likely originated from a fly within the Cecidomyiidae family.

Table 39. Distribution of BLAST best-scoring sequence pairs (BSPs) for the 100 consensus sequences with the highest depth of coverage by taxonomic rank. Aligned reads (ARs) are the number of reads that contributed to a given consensus sequence query. %ARs are scaled by the sum of reads comprising queries with at least one BSP. Results are shown for taxonomic ranks comprising >5% of ARs and the hierarchy is collapsed to the lowest rank with identical results. %ID is mean percent sequence identity, length is the mean alignment length, e-value is the mean expect value, and bitscore is the mean bitscore.

kingdom	phylum	class	order	family	%ARs	%ID	length	e-value	bitscore

Metazoa	Arthropoda	Insecta			100	88	832	6^{-45}	1,087
Metazoa	Arthropoda	Insecta	Diptera		99	88	850	6^{-45}	1,111
Metazoa	Arthropoda	Insecta	Diptera	Cecidomyiidae	98	88	849	6^{-45}	1,109

Cecidomyiidae are speciose in Sweden, with 639 species distributed among 97 genera. A similar number of taxa are likely present in Sweden but are still undescribed (Ronquist et al. 2020). *Contarinia* was reported 100 km south of the aerosol monitoring station after 2008, but given the potentially large number of Cecidomyiidae species without a reference genome, the genus-rank assignment may not be accurate.

Table 40. Distribution of BLAST best-scoring sequence pairs (BSPs) by taxonomic rank for 100 randomly-selected pair-end reads. Results are shown for taxonomic ranks comprising >5% of reads and the hierarchy is collapsed to the lowest rank with identical results. %ID is mean percent sequence identity, length is the mean alignment length, e-value is the mean expect value, and bitscore is the mean bitscore.

kingdom	phylum	class	order	family	%BSPs	%ID	length	e-value	bitscore
Metazoa	Arthropoda	Insecta			100	92	128	3^{-27}	182
Metazoa	Arthropoda	Insecta	Diptera		95	92	129	3^{-27}	182
Metazoa	Arthropoda	Insecta	Diptera	Cecidomyiidae	90	92	129	3^{-27}	184

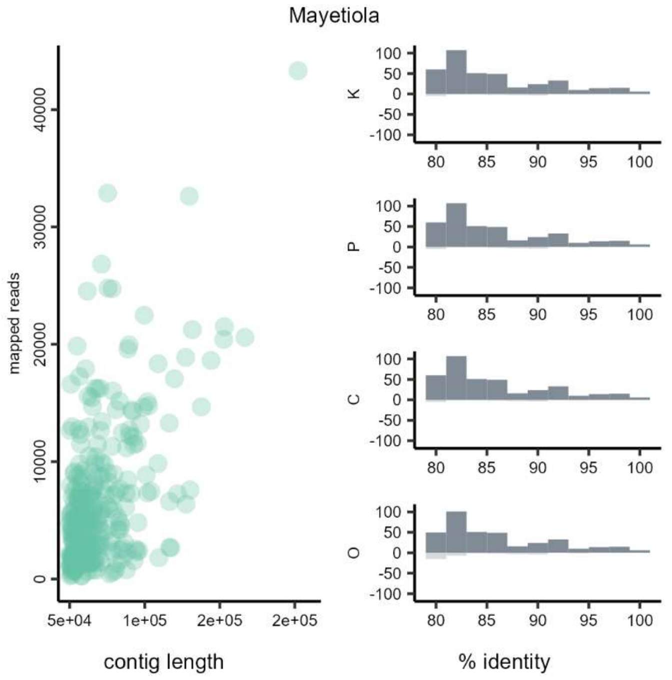

Figure 28. Left: number of mapped reads *vs*. contig length for contigs > 50 kb. Right: mirrored histograms of the sequence identity of aligned reads (ARs) with BLAST best-scoring sequence pairs (BSPs) within (top) or outside (bottom) the kingdom (K), phylum (P), class (C), and order (O) ranks of *Mayetiola*. BSPs are shown for the 100 consensus sequences with the highest depth of coverage. ARs are the number of reads covering a given consensus sequence query and are given as *n*-fold differences.

Mayetiola; Arthropoda; Insecta; Diptera; Cecidomyiidae (39757)

Label: unlabeled
Predicted probability: 0.87
Mapped reads: 53,085,824
Mapping rate: 71%
Base pairs queried: 717,529
Unique contigs queried: 98
Unique contigs aligned: 67

Mayetiola destructor is an important cereal pest worldwide, but the genus has not been reported in Fennoscandia. Two draft genomes for *M. destructor* of similar size and contiguity were included in the Kraken 2 database (126.0 Mb, N50: 9 kb and 139.9 Mb, N50: 13 kb).

71% of *Mayetiola*-classified reads mapped back to their sequences in the reference database. The number of reads mapped per contig was moderately ($r = 0.54$) correlated with contig length (Fig. 29).

Most of the 100 consensus queries had BSPs within Insecta, and 91% of ARs had BSPs within Diptera (Table 41). 4.2% of ARs had BSPs with *Alphadintovirus mayetiola*, a double-stranded DNA virus named for its host, *Mayetiola* (Table 40). These alignments (average 84.7% identity and 688 bp) may result from endogenized transposons rather than intact viruses, but we did not investigate them further. No other family than Cecidomyiidae comprised ≥ 5% of ARs.

Table 41. Distribution of BLAST best-scoring sequence pairs (BSPs) for the 100 consensus sequences with the highest depth of coverage by taxonomic rank. Aligned reads (ARs) are the number of reads that contributed to a given consensus sequence query. %ARs are scaled by the sum of reads comprising queries with at least one BSP. Results are shown for taxonomic ranks comprising >5% of ARs and the hierarchy is collapsed to the lowest rank with identical results. %ID is mean percent sequence identity, length is the mean alignment length, e-value is the mean expect value, and bitscore is the mean bitscore.

kingdom	phylum	class	order	family	%ARs	%ID	length	e-value	bitscore
Metazoa	Arthropoda	Insecta			95	85	619	1^{-29}	720
Metazoa	Arthropoda	Insecta	Diptera		91	85	641	1^{-29}	748
Metazoa	Arthropoda	Insecta	Diptera	Cecidomyiidae	66	85	715	2^{-29}	843

Mayetiola was also used to compare the results of directly querying reads against the nt database *vs.* the results from focusing on high-coverage consensus queries. Out of the 100 randomly-selected paired-end reads, 87% aligned within Diptera and 69% within the Cecidomyiidae (Table

42). These results are comparable to the consensus queries, although directly querying the reads found alignments within Lepidoptera and Hemiptera. While the genus-rank label is presumably incorrect, 'Mayetiola' appears to be a fly, most likely from the Cecidomyiidae family.

Table 42. Distribution of BLAST best-scoring sequence pairs (BSPs) by taxonomic rank for 100 randomly-selected pair-end reads. Results are shown for taxonomic ranks comprising >5% of reads and the hierarchy is collapsed to the lowest rank with identical results. %ID is mean percent sequence identity, length is the mean alignment length, e-value is the mean expect value, and bitscore is the mean bitscore.

kingdom	phylum	class	order	family	%BSPs	%ID	length	e-value	bitscore
Metazoa	Arthropoda	Insecta			100	87	137	1^{-27}	166
Metazoa	Arthropoda	Insecta	Diptera		87	87	137	1^{-27}	166
Metazoa	Arthropoda	Insecta	Diptera	Cecidomyiidae	69	87	139	3^{-30}	170
Metazoa	Arthropoda	Insecta	Lepidoptera		8	87	142	1^{-28}	176
Metazoa	Arthropoda	Insecta	Hemiptera	Pseudococcidae	5	85	132	2^{-31}	149

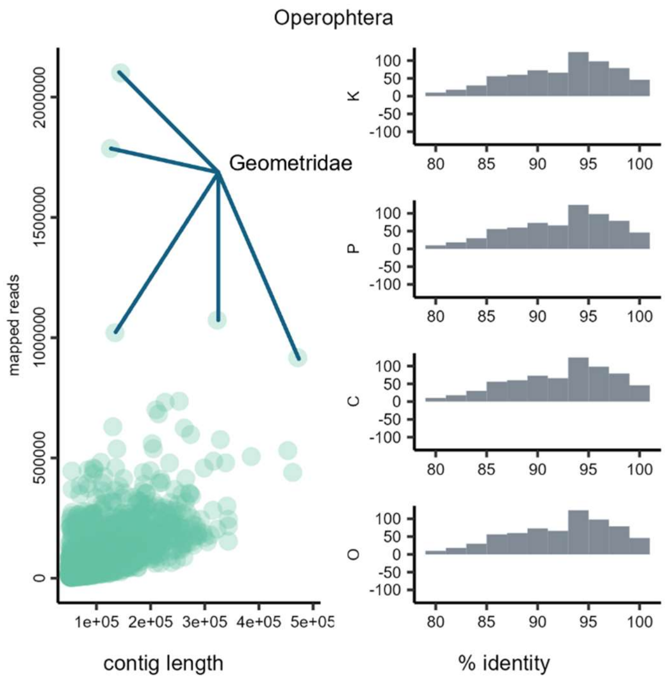

Figure 29. Left: number of mapped reads *vs*. contig length for contigs > 50 kb. Right: mirrored histograms of the sequence identity of aligned reads (ARs) with BLAST best-scoring sequence pairs (BSPs) within (top) or outside (bottom) the kingdom (K), phylum (P), class (C), and order (O) ranks of *Operophtera*. BSPs are shown for the 100 consensus sequences with the highest depth of coverage. ARs are the number of reads covering a given consensus sequence query and are given as *n*-fold differences.

Operophtera; Arthropoda; Insecta; Lepidoptera; Geometridae (104451)

Label: positive
Predicted probability: 0.96
Mapped reads: 624,563,548
Mapping rate: 49%
Base pairs queried: 2,793,501
Unique contigs queried: 100
Unique contigs aligned: 100

A nuclear genome assembly for *Operophtera brumata* (638.2 Mb, N50 =29 kb) was included in the Kraken 2 reference database. *O. brumata* is the most common species in northern Sweden, with occasional reports of *O. fagata*.

As observed in most other insect genera, relatively few (49%) of the Operophthera-*classified* reads mapped to their reference database sequences. Similarly, the number of reads mapped per contig was moderately correlated with contig length ($r = 0.56$; Fig. 29), a pattern we observed in the majority of insect genera.

All consensus queries had BSPs exclusively within Lepidoptera and 98% of ARs were specifically within the Geometridae family (Table 43). This included consensus queries from contigs with very high depth of coverage, as indicated in the scatterplot in Fig. 30.

Table 43. Distribution of BLAST best-scoring sequence pairs (BSPs) for the 100 consensus sequences with the highest depth of coverage by taxonomic rank. Aligned reads (ARs) are the number of reads that contributed to a given consensus sequence query. %ARs are scaled by the sum of reads comprising queries with at least one BSP. Results are shown for taxonomic ranks comprising >5% of ARs and the hierarchy is collapsed to the lowest rank with identical results. %ID is mean percent sequence identity, length is the mean alignment length, e-value is the mean expect value, and bitscore is the mean bitscore.

kingdom	phylum	class	order	family	%ARs	%ID	length	e-value	bitscore
Metazoa	Arthropoda	Insecta	Lepidoptera		100	92	1,341	3^{-75}	1,944
Metazoa	Arthropoda	Insecta	Lepidoptera	Geometridae	98	92	1,336	3^{-75}	1,938

Operophtera was also one of the genera used to compare the results of directly querying reads against the nt database. Out of 100 randomly selected paired-end reads, all alignments were within Lepidoptera and 98% within the Geometridae family (Table 44). These proportions are identical to those estimated by the mapped consensus queries, even though a relatively small (49%) fraction of *Operophtera*-classified reads could be successfully mapped back to their Kraken 2 reference database sequences. Based on this combined evidence, we consider *Operophtera* a true positive genus with few misclassified reads.

Table 44. Distribution of BLAST best-scoring sequence pairs (BSPs) by taxonomic rank for 100 randomly-selected pair-end reads. Results are shown for taxonomic ranks comprising >5% of reads and the hierarchy is collapsed to the lowest rank with identical results. %ID is mean percent sequence identity, length is the mean alignment length, e-value is the mean expect value, and bitscore is the mean bitscore.

kingdom	phylum	class	order	family	%BSPs	%ID	length	e-value	bitscore
Metazoa	Arthropoda	Insecta	Lepidoptera		100	96	135	7^{-28}	216
Metazoa	Arthropoda	Insecta	Lepidoptera	Geometridae	98	96	135	5^{-28}	216

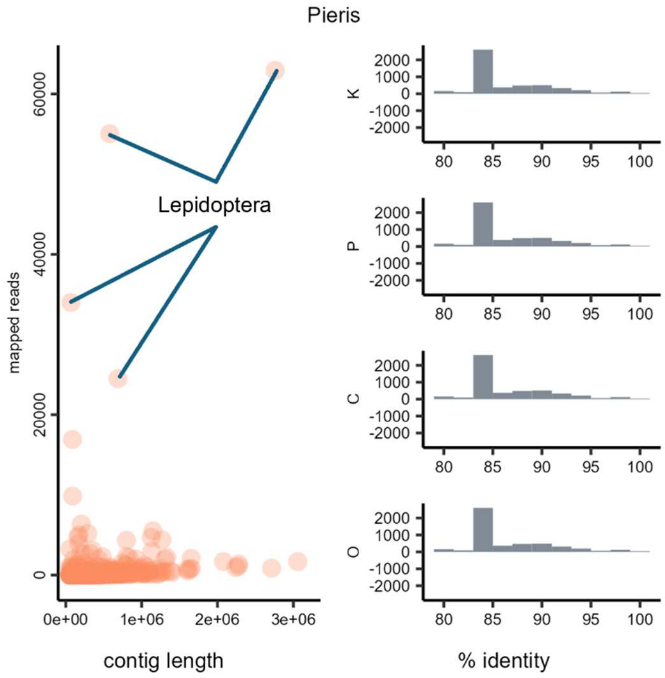

Figure 30. Left: number of mapped reads *vs*. contig length for contigs > 50 kb. Right: mirrored histograms of the sequence identity of aligned reads (ARs) with BLAST best-scoring sequence pairs (BSPs) within (top) or outside (bottom) the kingdom (K), phylum (P), class (C), and order (O) ranks of *Pieris*. BSPs are shown for the 100 consensus sequences with the highest depth of coverage. ARs are the number of reads covering a given consensus sequence query and are given as *n*-fold differences.

Pieris; Arthropoda; Insecta; Lepidoptera; Pieridae (7115)

Label: positive
Predicted probability: 1.00
Mapped reads: 934,290
Mapping rate: 58%
Base pairs queried: 49,066
Unique contigs queried: 92
Unique contigs aligned: 89

Pieris was included in the GBM training dataset as a ‘positive’ genus. A nuclear genome assembly for *Pieris rapae* (230.3 Mb, N50: 578 kb) was available at the time of the Kraken 2 database construction. *P. napi* is common in northern Sweden.

While the mapped reads did not show the positive correlation with reference contig length expected from a true positive (r = 0.22; Fig. 31), the sequences from these high coverage contigs had BSPs with Lepidoptera (Table 45). Overall, 98% of ARs were aligned within Lepidoptera and 55% within the Pieridae family. All BSPs within Pieridae were specifically with *Pieris*.

Table 45. Distribution of BLAST best-scoring sequence pairs (BSPs) for the 100 consensus sequences with the highest depth of coverage by taxonomic rank. Aligned reads (ARs) are the number of reads that contributed to a given consensus sequence query. %ARs are scaled by the sum of reads comprising queries with at least one BSP. Results are shown for taxonomic ranks comprising >5% of ARs and the hierarchy is collapsed to the lowest rank with identical results. %ID is mean percent sequence identity, length is the mean alignment length, e-value is the mean expect value, and bitscore is the mean bitscore.

kingdom	phylum	class	order	family	%ARs	%ID	length	e-value	bitscore
Metazoa	Arthropoda	Insecta			100	91	464	2^{-31}	665
Metazoa	Arthropoda	Insecta	Lepidoptera		98	91	484	2^{-31}	695
Metazoa	Arthropoda	Insecta	Lepidoptera	Pieridae	55	88	533	2^{-34}	711
Metazoa	Arthropoda	Insecta	Lepidoptera	Pyralidae	11	89	540	5^{-50}	712
Metazoa	Arthropoda	Insecta	Lepidoptera	Oecophoridae	9	89	237	2^{-55}	326
Metazoa	Arthropoda	Insecta	Lepidoptera	Geometridae	8	91	466	4^{-33}	653
Metazoa	Arthropoda	Insecta	Lepidoptera	Yponomeutidae	5	87	304	2^{-45}	388

Fungi

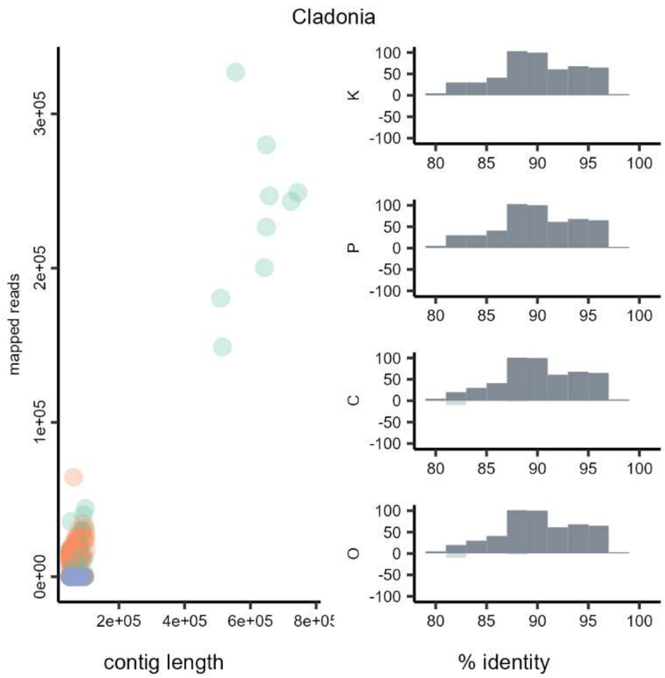

Figure 31. Left: number of mapped reads *vs*. contig length for contigs > 50 kb. Right: mirrored histograms of the sequence identity of aligned reads (ARs) with BLAST best-scoring sequence pairs (BSPs) within (top) or outside (bottom) the kingdom (K), phylum (P), class (C), and order (O) ranks of *Cladonia*. BSPs are shown for the 100 consensus sequences with the highest depth of coverage. ARs are the number of reads covering a given consensus sequence query and are given as *n*-fold differences.

***Cladonia*; Ascomycota; Lecanoromycetes; Lecanorales; Cladoniaceae (5199)**

Label: positive
Predicted probability: 0.98
Mapped reads: 21,045,418
Mapping rate: 93%
Base pairs queried: 1,616,189
Unique contigs queried: 72
Unique contigs aligned: 34

Fifty *Cladonia* species have been documented in Norrbotten county, with *C. parasitica* and *C. rangiferina* the most common. Assemblies of the *ca*. 35 Mb *Cladonia* mycobiont nuclear genome were available for *C. rangeriferina* (35.6 Mb, N50: 141 kb), shown in green, and *C. uncialils* (32.3 Mb, N50: 35 kb), in orange in Fig. 31. Most of the *Cladonia*-classified reads mapped to their sequences in the reference database and the number of mapped reads per contig was positively correlated with contig length ($r = 0.95$; Fig. 31).

Most consensus queries had BSPs exclusively within Lecanorales and 94% of their ARs were within the Cladoniaceae family; all the latter were specifically with *Cladonia* (Table 46).

Table 46. Distribution of BLAST best-scoring sequence pairs (BSPs) for the 100 consensus sequences with the highest depth of coverage by taxonomic rank. Aligned reads (ARs) are the number of reads that contributed to a given consensus sequence query. %ARs are scaled by the sum of reads comprising queries with at least one BSP. Results are shown for taxonomic ranks comprising >5% of ARs and the hierarchy is collapsed to the lowest rank with identical results. %ID is mean percent sequence identity, length is the mean alignment length, e-value is the mean expect value, and bitscore is the mean bitscore.

kingdom	phylum	class	order	family	%ARs	%ID	length	e-value	bitscore
Fungi	Ascomycota				100	88	2,356	4^{-38}	3,242
Fungi	Ascomycota	Lecanoromycetes	Lecanorales		98	89	2,404	4^{-38}	3,311
Fungi	Ascomycota	Lecanoromycetes	Lecanorales	Cladoniaceae	94	89	2,508	3^{-38}	3,456

We also queried 100 randomly selected paired end reads against the nt database and found that 95% of alignments were within the Cladoniaceae family, all of which were specifically with *Cladonia* (Table 47).

Table 47. Distribution of BLAST best-scoring sequence pairs (BSPs) by taxonomic rank for 100 randomly-selected pair-end reads. Results are shown for taxonomic ranks comprising >5% of reads and the hierarchy is collapsed to the lowest rank with identical results. %ID is mean percent sequence identity, length is the mean alignment length, e-value is the mean expect value, and bitscore is the mean bitscore.

kingdom	phylum	class	order	family	%BSPs	%ID	length	e-value	bitscore
Fungi	Ascomycota				100	94	134	1^{-27}	204
Fungi	Ascomycota	Lecanoromycetes	Lecanorales		98	94	134	1^{-27}	204
Fungi	Ascomycota	Lecanoromycetes	Lecanorales	Cladoniaceae	95	94	134	1^{-27}	204

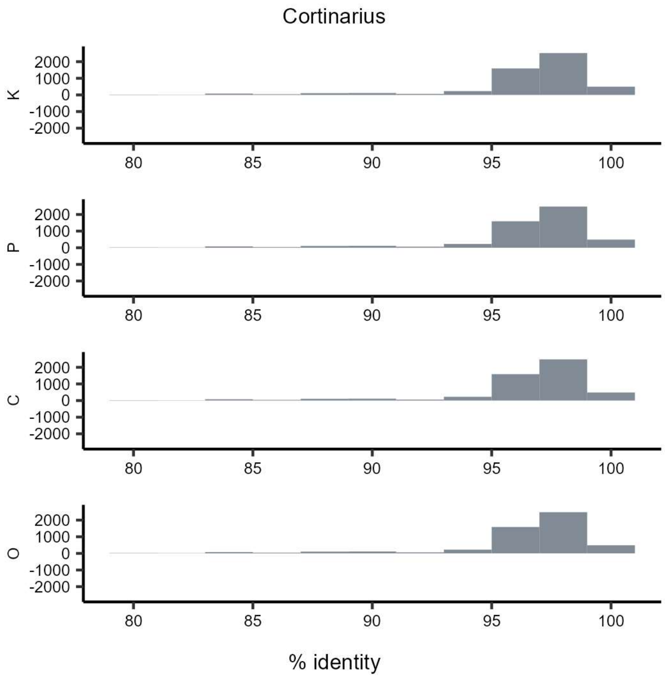

Figure 32. Mirrored histograms of the sequence identity of aligned reads (ARs) with BLAST best-scoring sequence pairs (BSPs) within (top) or outside (bottom) the kingdom (K), phylum (P), class (C), and order (O) ranks of *Cortinarius*. BSPs are shown for the 100 consensus sequences with the highest depth of coverage. ARs are the number of reads covering a given consensus sequence query and are given as *n*-fold differences.

***Cortinarius*; Basidiomycota; Agaricomycetes; Agaricales; Cortinariaceae (34451)**

Label: positive
Predicted probability: 0.99
Mapped reads: 347,926
Mapping rate: 80%
Base pairs queried: 49,883
Unique contigs queried: 96
Unique contigs aligned: 94

Forty-eight *Cortinarius* species were reported within 40 km of the aerosol monitoring station between 1974 and 2008.

No nuclear genome assemblies were available for the Kraken 2 database, but 3,456 sequences, largely from the rRNA operon and mitochondrial genes, were included (3.1 Mb, N50: 1 kb). Most *Cortinarius*-classified reads mapped back to these sequences but we did not investigate the correlation between contig length and mapped read count.

Almost all consensus query BSPs were within the Agaricales, and the majority of ARs (72%) were within the Cortinariaceae family (Fig. 33, Table 48). BSPs were also found within the Strophariaceae family with similar sequence identity and alignment lengths. Strophariaceae as presently defined is polyphyletic (Vizzini et al. 2024; Wang et al. 2024) and relationships within *Cortinarius* (until recently the only genus in Cortinariaceae) are not resolved (Liimatainen et al. 2022; Gallone et al. 2024). Given this uncertainty, it seems plausible that some *Cortinarius*-classified reads may originate from taxa that will eventually be recognized in different genera or families. Such misclassifications, however, would not directly result from problems with the

classifier or the reference library but rather reflect our current (lack of) understanding of the diversity of *Cortinarius*.

Table 48. Distribution of BLAST best-scoring sequence pairs (BSPs) for the 100 consensus sequences with the highest depth of coverage by taxonomic rank. Aligned reads (ARs) are the number of reads that contributed to a given consensus sequence query. %ARs are scaled by the sum of reads comprising queries with at least one BSP. Results are shown for taxonomic ranks comprising >5% of ARs and the hierarchy is collapsed to the lowest rank with identical results. %ID is mean percent sequence identity, length is the mean alignment length, e-value is the mean expect value, and bitscore is the mean bitscore.

kingdom	phylum	class	order	family	%ARs	%ID	length	e-value	bitscore
Fungi					100	94	512	3^{-49}	798
Fungi	Basidiomycota	Agaricomycetes	Agaricales		99	94	512	3^{-49}	797
Fungi	Basidiomycota	Agaricomycetes	Agaricales	Cortinariaceae	72	94	518	5^{-64}	810
Fungi	Basidiomycota	Agaricomycetes	Agaricales	Strophariaceae	26	93	749	9^{-124}	1,171

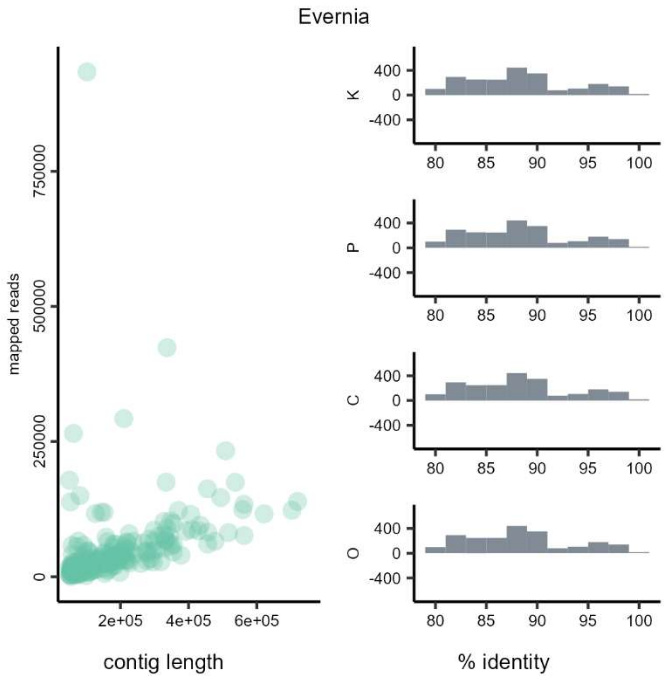

Figure 33. Left: number of mapped reads *vs*. contig length for contigs > 50 kb. Right: mirrored histograms of the sequence identity of aligned reads (ARs) with BLAST best-scoring sequence pairs (BSPs) within (top) or outside (bottom) the kingdom (K), phylum (P), class (C), and order (O) ranks of *Evernia*. BSPs are shown for the 100 consensus sequences with the highest depth of coverage. ARs are the number of reads covering a given consensus sequence query and are given as *n*-fold differences.

Evernia; Ascomycota; Lecanoromycetes; Lecanorales; Parmeliaceae (87256)

Label: unlabeled
Predicted probability: 0.98
Mapped reads: 18,029,788
Mapping rate: 90%
Base pairs queried: 780,267
Unique contigs queried: 88
Unique contigs aligned:71

Evernia was flagged for validation because <°4 observations were reported within 40 km of the monitoring station between 1974-2008, although >°100 have been made since then.

A nuclear genome assembly for *Evernia prunastri* (39.4 Mb; N50: 260 kb) was included in the Kraken°2 reference database. This species, along with *E. mesomorpha* and *E. divaricate* have been documented near the aerosol station in recent years. Contig length and the number of mapped reads were weakly correlated (*r*°=°0.30) but this increased to *r*°=°0.52 without the single high-depth contig.

Consensus sequences BSPs were entirely within the order Lecanorales (Table 49). 96% of their ARs were within the Parmeliaceae family. Only 159 nucleotide sequences from *Evernia* were in

the nucleotide database (September 2014) and, as a result, genera-rank alignments were with other Parmeliaceae genera. The consensus sequence from the contig with the most mapped reads aligned with three Parmeliaceae genomes released by two different institutions.

Table 49. Distribution of BLAST best-scoring sequence pairs (BSPs) for the 100 consensus sequences with the highest depth of coverage by taxonomic rank. Aligned reads (ARs) are the number of reads that contributed to a given consensus sequence query. %ARs are scaled by the sum of reads comprising queries with at least one BSP. Results are shown for taxonomic ranks comprising >5% of ARs and the hierarchy is collapsed to the lowest rank with identical results. %ID is mean percent sequence identity, length is the mean alignment length, e-value is the mean expect value, and bitscore is the mean bitscore.

kingdom	phylum	class	order	family	%ARs	%ID	length	e-value	bitscore
Fungi	Ascomycota	Lecanoromycetes	Lecanorales		100	88	1,134	1E-34	1,477
Fungi	Ascomycota	Lecanoromycetes	Lecanorales	Parmeliaceae	96	88	1,119	1E-34	1,467

Fibularhizoctonia **(syn. *Athelia*); Basidiomycota; Agaricomycetes; Atheliales; Atheliaceae (56747)**

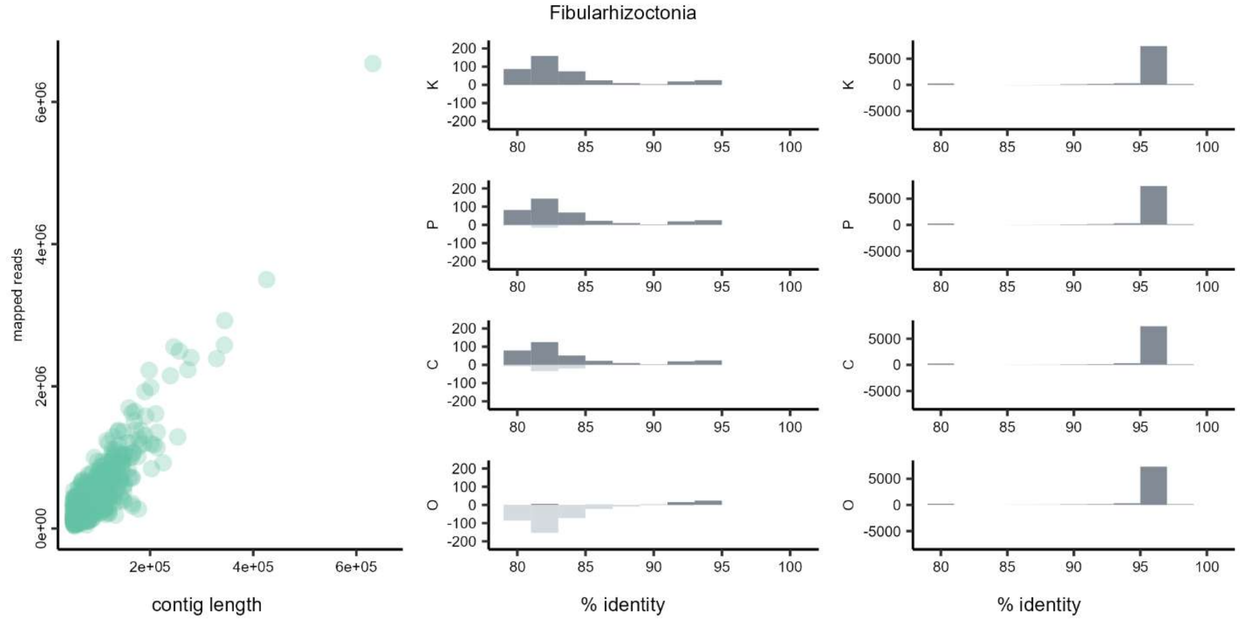

Figure 34. Left: number of mapped reads *vs*. contig length for contigs > 50 kb. Middle: mirrored histograms of the sequence identity of aligned reads (ARs) with BLAST best-scoring sequence pairs (BSPs) within (top) or outside (bottom) the kingdom (K), phylum (P), class (C), and order (O) ranks of *Athelia*. BSPs are shown for the 100 consensus sequences with the highest depth of coverage. Right: as the middle panel but with consensus sequences from the 21 reference contigs that were not part of the Fibularhizoctonia genome assembly. ARs are the number of reads covering a given consensus sequence query and are given as *n*-fold differences.

Label: unlabeled
Predicted probability: 0.99
Mapped reads: 410,092,778
Mapping rate: 90%

Base pairs queried: 12,810,516
Unique contigs queried: 100
Unique contigs aligned: 69

We selected *Athelia* (syn. *Fibularhizoctonia*) for validation based on its abundance and the large decline in its γ-diversity contribution in the second half of the time series. No occurrences have been reported within 40 km of the aerosol monitoring station, but corticoid fungi with ephemeral fruiting bodies like *Athelia* are difficult to find without molecular methods (Berglund et al. 2005).

A nuclear genome assembly for *Fibularhizoctonia sp.* CBS 109695 was included in the Kraken 2 reference database (88.1 Mb, N50: 54 kb). Note a dual naming system for sexual and asexual forms persists in fungi and this name is an accepted synonym for *Athelia* (Turland et al. 2017). Most (90%) of *Athelia*-classified reads mapped to their sequences in the reference database. The number of read mapped per contig was strongly correlated with contig length ($r = 0.90$), the expected pattern for a positive genus (Fig. 35).

BLAST results for 100 high-depth consensus queries were ambiguous (Table 50). While BSPs for these sequences were entirely within Fungi and the majority within Agaricomycetes, they aligned with taxa from the Polyporales, Agaricales, Boletales, and Russulales orders. Only a minority of the corresponding aligned reads (10%) had BSPs within the expected Atheliales order. This result was surprising, as we queried 12.8 Mb of consensus sequences and this would suggest that almost the entire *Fibularhizoctonia* assembly is from a different organism.

Table 50. Distribution of BLAST best-scoring sequence pairs (BSPs) for the 100 consensus sequences with the highest depth of coverage by taxonomic rank. Aligned reads (ARs) are the number of reads that contributed to a given consensus sequence query. %ARs are scaled by the sum of reads comprising queries with at least one BSP. Results are shown for taxonomic ranks comprising >5% of ARs and the hierarchy is collapsed to the lowest rank with identical results. %ID is mean percent sequence identity, length is the mean alignment length, e-value is the mean expect value, and bitscore is the mean bitscore.

kingdom	phylum	class	order	family	%ARs	%ID	length	e-value	bitscore
Fungi					100	83	309	1^{-29}	336
Fungi	Basidiomycota				93	83	311	2^{-31}	339
Fungi	Basidiomycota	Agaricomycetes			83	83	314	2^{-31}	342
Fungi	Basidiomycota	Agaricomycetes	Polyporales		30	83	316	3^{-31}	338
Fungi	Basidiomycota	Agaricomycetes	Polyporales	Polypora-ceae	13	83	322	7^{-37}	349
Fungi	Basidiomycota	Agaricomycetes	Polyporales	Fomitops-idaceae	7	82	363	1^{-30}	374
Fungi	Basidiomycota	Agaricomycetes	Agaricales		22	83	276	3^{-31}	291
Fungi	Basidiomycota	Agaricomycetes	Boletales		11	82	304	5^{-34}	315
Fungi	Basidiomycota	Agaricomycetes	Boletales	Suillaceae	5	82	359	3^{-57}	371
Fungi	Basidiomycota	Agaricomycetes	Atheliales		10	89	632	9^{-105}	868
Fungi	Basidiomycota	Agaricomycetes	Russulales		6	82	294	7^{-34}	305

Alternatively, we suspected the taxonomic distribution of BSPs could be explained by the poor representation of Atheliales in the nt database (1,768 sequences, August 2024). We conducted a second search using queries from the 21 reference contigs that were not part of the *Fibularhizoctonia* genome assembly. Most of these were from the rRNA operon or mitochondrion. Almost all BSPs with all these queries, shown on the right in Fig. 35 and summarized in Table 51, were within the Atheliaceae family. Despite the unexpected

distribution of BSPs at the order rank for the genomic consensus queries, we conclude these sequences are also most likely from Atheliaceae, if not *Athelia* itself.

Table 51. Distribution of BLAST best-scoring sequence pairs (BSPs) for 21 consensus sequences that were not part of the *Fibularhizoctonia* assembly by taxonomic rank. Aligned reads (ARs) are the number of reads that contributed to a given consensus sequence query. %ARs are scaled by the sum of reads comprising queries with at least one BSP. Results are shown for taxonomic ranks comprising >5% of ARs and the hierarchy is collapsed to the lowest rank with identical results. %ID is mean percent sequence identity, length is the mean alignment length, e-value is the mean expect value, and bitscore is the mean bitscore.

kingdom	phylum	class	order	family	%ARs	%ID	length	e-value	bitscore
Fungi	Basidiomycota	Agaricomycetes			100	93	505	4^{-67}	755
Fungi	Basidiomycota	Agaricomycetes	Atheliales	Atheliaceae	99	94	531	1^{-67}	802

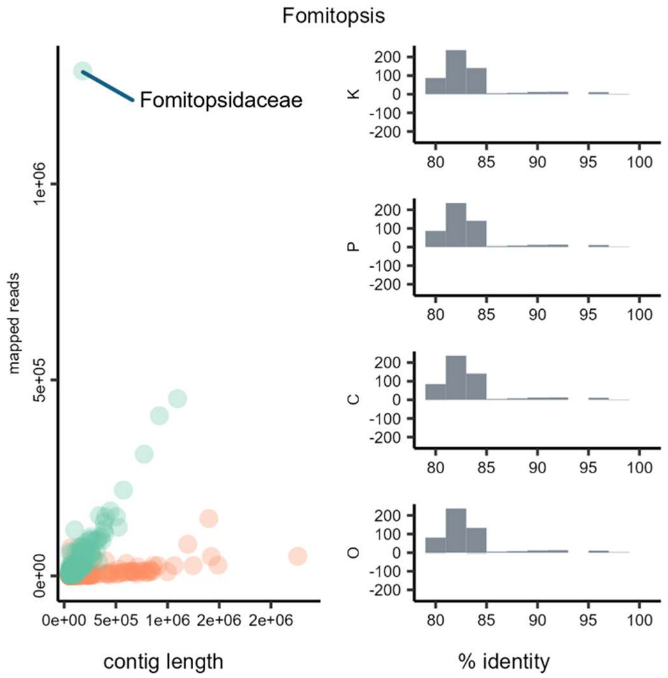

Figure 35. Left: number of mapped reads *vs*. contig length for contigs > 50 kb. Right: mirrored histograms of the sequence identity of aligned reads (ARs) with BLAST best-scoring sequence pairs (BSPs) within (top) or outside (bottom) the kingdom (K), phylum (P), class (C), and order (O) ranks of *Fomitopsis*. BSPs are shown for the 100 consensus sequences with the highest depth of coverage. ARs are the number of reads covering a given consensus sequence query and are given as *n*-fold differences.

Fomitopsis; Basidiomycota; Agaricomycetes; Polyporales; Fomitopsidaceae (34474)

Label: positive
Predicted probability: 0.99
Mapped reads: 27,663,078
Mapping rate: 88%
Base pairs queried: 476,216
Unique contigs queried: 99
Unique contigs aligned: 29

Nuclear genome assemblies for *Fomitopsis pinicola* (45.1 Mb, N50: 107 kb), *F. palustris* (43.5 MB, N50: 664 kb), and *F. rosea* (36.4 Mb, N50: 39 kb) were included in the Kraken 2 reference database.

Most (88%) *Fomitopsis*-classified reads could be mapped back to their reference sequences. Of these, more reads mapped to the *F. pinicola* assembly (84%), in green, than to *F. palustris* (5%), in orange, or *F. rosea* (10%), which is not visible in Fig. 30 due to the relatively low contiguity of this assembly. *F. pinicola* is common throughout Fennoscandia, whereas *F. rosea* is geographically widespread but is restricted to older spruce forests. *F. palustris* is reported from the Caribbean and the Gulf of Mexico.

Longer contigs in the *F. pinicola* generally tended to have more mapped reads ($r = 0.52$), with one conspicuous outlier, as labeled on the scatterplot (Fig. 36). The consensus sequence from this contig had BSPs with genomes in the Fomitopsidaceae and Taiwanofungaceae , a related family (Han et al. 2016), produced by three different labs.

All consensus queries had their BSPs exclusively within Basidiomycota and 97% of their ARs were within the Polyporales order (Table 52). At the family rank, most BSPs were within Fomitopsidaceae but about 16% their comprising reads aligned to taxa in the Polyporaceae. These two families contain morphologically similar taxa and effort to delimit reciprocally monophyletic families (and genera, Han, et al. 2016) within the Polyporales (e.g. Justo and Hibbett 2011; Justo et al. 2017) is ongoing. Therefore, we consider the taxonomic distribution of BSPs to result from unresolved phylogenies rather than reference contamination.

Table 52. Distribution of BLAST best-scoring sequence pairs (BSPs) for the 100 consensus sequences with the highest depth of coverage by taxonomic rank. Aligned reads (ARs) are the number of reads that contributed to a given consensus sequence query. %ARs are scaled by the sum of reads comprising queries with at least one BSP. Results are shown for taxonomic ranks comprising >5% of ARs and the hierarchy is collapsed to the lowest rank with identical results. %ID is mean percent sequence identity, length is the mean alignment length, e-value is the mean expect value, and bitscore is the mean bitscore.

kingdom	phylum	class	order	family	%ARs	%ID	length	e-value	bitscore
Fungi	Basidiomycota				100	84	559	3^{-28}	667
Fungi	Basidiomycota	Agaricomycetes			99	84	564	2^{-28}	673
Fungi	Basidiomycota	Agaricomycetes	Polyporales		97	84	529	1^{-28}	638
Fungi	Basidiomycota	Agaricomycetes	Polyporales	Fomitopsidaceae	76	85	662	2^{-28}	817
Fungi	Basidiomycota	Agaricomycetes	Polyporales	Polyporaceae	16	82	263	1^{-30}	266

Lachnellula; Ascomycota; Leotiomycetes; Helotiales; Lachnaceae (47830)

Label: unlabeled
Predicted probability: 0.99
Mapped reads: 54,176,612
Mapping rate: 95%
Base pairs queried: 6,510,865
Unique contigs queried: 100
Unique contigs aligned: 63

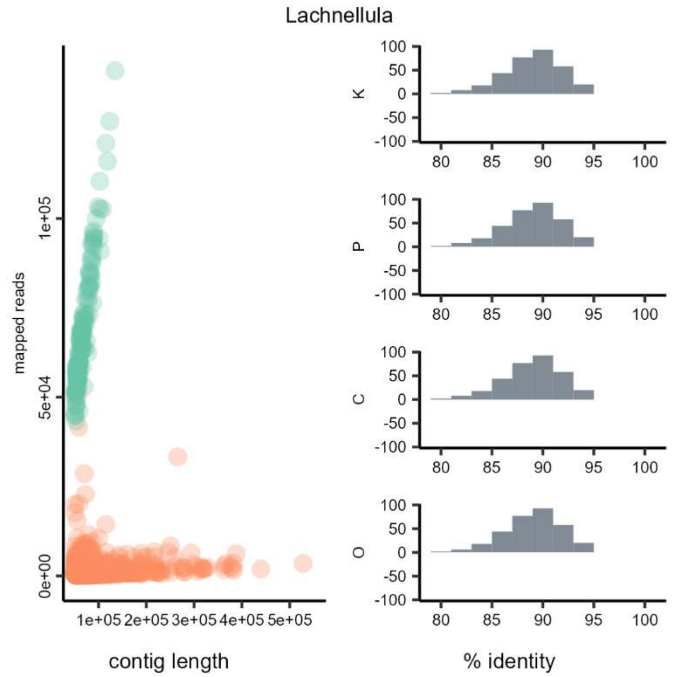

Figure 36. Left: number of mapped reads *vs*. contig length for contigs > 50 kb. Right: mirrored histograms of the sequence identity of aligned reads (ARs) with BLAST best-scoring sequence pairs (BSPs) within (top) or outside (bottom) the kingdom (K), phylum (P), class (C), and order (O) ranks of *Lachnellula*. BSPs are shown for the 100 consensus sequences with the highest depth of coverage. ARs are the number of reads covering a given consensus sequence query and are given as *n*-fold differences.

We flagged *Lachnellula* for validation based on its abundance and because of its large decline in γ-diversity contribution in the second half of the time series. Seven *Lachnellula* draft assemblies, all from the same lab, were included in the Kraken 2. These were published by the Canadian Food Inspection Agency, although no other locality information was provided.

95% of the *Lachnellula*-classified reads mapped to back to their reference sequences. Of these, 85% mapped to the *L. cervina* (48.3 Mb, N50: 24 kb) assembly, indicated in green in Fig. 37. Contig length and mapped read count were strongly correlated for this assembly ($r = 0.97$; Fig. 37). To our knowledge, *L. cervina* has not been reported in Sweden, but we could not locate more information about this species at all. Nevertheless, some *Lachnellula* species are documented as conifer pathogens in Fennoscandia.

All consensus queries had BSPs within the class Leotiomycetes and 99% of their ARs were with the Lachnaceae family (Table 53). Although information about this genus is lacking, we found no evidence of reference contamination and conclude the *Lachnellula*-classified reads are from this or related genus.

Table 53. Distribution of BLAST best-scoring sequence pairs (BSPs) for the 100 consensus sequences with the highest depth of coverage by taxonomic rank. Aligned reads (ARs) are the number of reads that contributed to a given consensus sequence query. %ARs are scaled by the sum of reads comprising queries with at least one BSP. Results are shown for taxonomic ranks comprising >5% of ARs and the hierarchy is collapsed to the lowest rank with identical results. %ID is mean percent sequence identity, length is the mean alignment length, e-value is the mean expect value, and bitscore is the mean bitscore.

kingdom	phylum	class	order	family	%ARs	%ID	length	e-value	bitscore
Fungi	Ascomycota	Leotiomycetes			100	89	1,862	0.0	2,490
Fungi	Ascomycota	Leotiomycetes	Helotiales	Lachnaceae	99	89	1,864	0.0	2,500

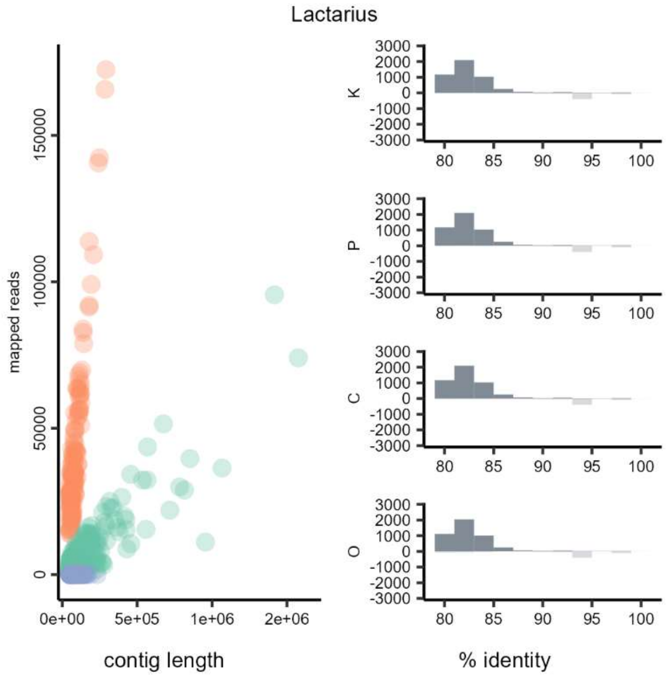

Figure 37. Left: number of mapped reads *vs*. contig length for contigs > 50 kb. Right: mirrored histograms of the sequence identity of aligned reads (ARs) with BLAST best-scoring sequence pairs (BSPs) within (top) or outside (bottom) the kingdom (K), phylum (P), class (C), and order (O) ranks of *Lactarius*. BSPs are shown for the 100 consensus sequences with the highest depth of coverage. ARs are the number of reads covering a given consensus sequence query and are given as *n*-fold differences.

Lactarius; Basidiomycota; Agaricomycetes; Russulales; Russulaceae (34444)

Label: positive
Predicted probability: 0.99
Mapped reads: 28,041,808
Mapping rate: 85%
Base pairs queried: 19,514,921
Unique contigs queried: 715
Unique contigs aligned: 467

Seven reference assemblies were available for *Lactarius*: two for *L. deliciosus* (53.9 Mb, N50: 20 kb and 54.3 Mb, N50: 114 kb), and one assembly for *L. hatsudake* (73.1 Mb, N50: 5 kb), *L. indigo* (75.4 Mb, N50: 13 kb, *L. piperatus* (49.9 Mb, N50: 16 kb), *L*. trivialis (34.9 Mb, N50: 34 kb), and *L. volemus* (43.6 Mb, N50: 27 kb). *Lactarius deliciosus* has been reported from northern Sweden and *L. trivalis* and is common throughout Fennoscandia.

85% of the *Lactarius*-classified reads mapped to their sequences in the reference database. Of these, 70% mapped to *L. trivalis*, shown in orange, 19% mapped to one of the *L. deliciosus* assemblies, in green in Fig XX. Less than 1% of reads mapped to *L. volemus* and *L. piperatus*, and *ca*. 5% mapped to *L. hatsudake* and *L. indigo*, all indicated in blue in Fig XX. The number of mapped reads per contig was positively correlated with contig length for *L. trivalis* (r = 0.96) and the *L. deliciosus* assemblies (r = 0.88; Fig. 38).

In our initial BLAST search of the top 100 consensus sequences by depth, 31% of reads aligned within Insecta, mostly (20%) within the dipteran family Cylindrotomidae. These alignments within Insecta originated from consensus sequences from four short (< 7 kb) contigs from two different assemblies. Two of the four queries had exceptionally high read depths of 339,900× and 194,096×, compared to the median of 30,560× coverage for the top 100 consensus sequences. This suggested that reference contamination may be largely confined to a few contigs. We expanded the BLAST analysis to the top 1,000 sequences by depth of coverage to investigate this possibility. These queries represent 12,445,807 aligned reads, or 44% of the total number of mapped reads.

The majority of BSPs for the 1,000 consensus queries were within Fungi, all of which were also within the class Agaricomycetes, and 85% of reads aligned within the Russulaceae family (Table 54). In total, we found six contigs that are likely from insects among the 715 unique queried contigs. Median depth of coverage for contaminant contigs (51,817×) was greater than the putatively non-contaminated contigs (8,834×), even excluding the two contaminants with

exceptional depth (26,992×). The top 1,000 consensus queries included depths greater than 4,558× and the lowest depth for a contaminant contig was 11,658×, which suggests we identified the bulk of relevant contaminants in the *Lactarius* assemblies. Therefore, we expect 10% to be a conservative estimate of the fraction of misclassified *Lactarius* reads due to reference contamination.

Table 54. Distribution of BLAST best-scoring sequence pairs (BSPs) for the 1,000 consensus sequences with the highest depth of coverage by taxonomic rank. Aligned reads (ARs) are the number of reads that contributed to a given consensus sequence query. %ARs are scaled by the sum of reads comprising queries with at least one BSP. Results are shown for taxonomic ranks comprising >5% of ARs and the hierarchy is collapsed to the lowest rank with identical results. %ID is mean percent sequence identity, length is the mean alignment length, e-value is the mean expect value, and bitscore is the mean bitscore.

kingdom	phylum	class	order	family	%ARs	%ID	length	e-value	bitscore
Fungi					89	82	1,532	1^{-28}	1,639
Fungi	Basidiomycota	Agaricomycetes			88	82	1,537	1^{-28}	1,644
Fungi	Basidiomycota	Agaricomycetes	Russulales		86	83	1,594	1^{-28}	1,706
Fungi	Basidiomycota	Agaricomycetes	Russulales	Russulaceae	85	83	1,606	1^{-28}	1,718
Metazoa	Arthropoda	Insecta	Diptera		10	92	646	3^{-31}	960
Metazoa	Arthropoda	Insecta	Diptera	Cylindrotomidae	6	93	1,236	0.0	1,845

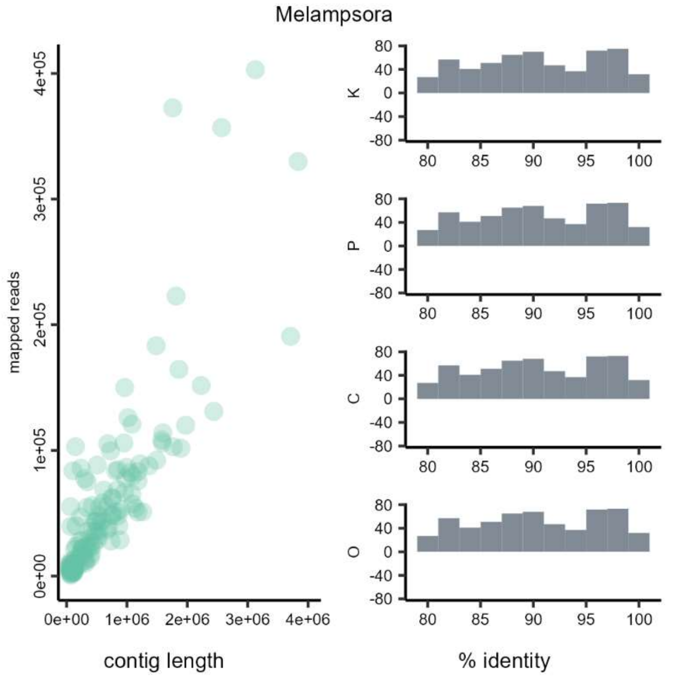

Figure 38. Left: number of mapped reads *vs*. contig length for contigs > 50 kb. Right: mirrored histograms of the sequence identity of aligned reads (ARs) with BLAST best-scoring sequence pairs (BSPs) within (top) or outside (bottom) the kingdom (K), phylum (P), class (C), and order (O) ranks of *Melampsora*. BSPs are shown for the 100 consensus sequences with the highest depth of coverage. ARs are the number of reads covering a given consensus sequence query and are given as *n*-fold differences.

***Melampsora*; Basidiomycota; Pucciniomycetes; Pucciniales; Melampsoraceae (5260)**

Label: unlabeled
Predicted probability: 0.88
Mapped reads: 31,073,142
Mapping rate: 79%
Base pairs queried: 649,996
Unique contigs queried: 79
Unique contigs aligned: 71

Melampsora includes fungal pathogens of pine and deciduous trees and occurs in Norrbotten province. We selected this taxon for validation because of the abundance of *Melampsora*-classified reads and because this genus, along with pine, was among the few with a greater γ-diversity contribution in the latter half of the time series.

Genome assemblies for *M. larici-populina* (95.2 Mb, N50: 1.1 Mb), *M. abietis-canadensis* (68.9 Mb, N50: 5 kb), *M. aecidioides* (56.5 Mb, N50: 2 kb), *M. allii-populina* (43.6 Mb, N50: 3 kb), *M. medusae*

(77.3 Mb, N50: 4 kb) and *M. occidentalis* (89.2 Mb, N50: 4 kb) were included in the Kraken 2 reference database. 79% of the *Melampsora*-classified reads mapped back to these assemblies, with the largest fraction mapping to *M. larici-populina* (36%), followed by *M. allii-populina* (23%) and *M. aecidioides* (22%). Only reads mapped to *M. larcini-populina* are visible in Fig. 39 due to the low contiguity of the other assembles and these were positively correlated with contig length ($r = 0.83$)

All best-scoring sequence pairs (BSPs) were within Fungi (Table 55). Weighted by read depth, 99% of alignments were within Pucciniales that 80% were also aligned to sequences from the Melampsoraceae family.

Table 55. Distribution of BLAST best-scoring sequence pairs (BSPs) for the 100 consensus sequences with the highest depth of coverage by taxonomic rank. Aligned reads (ARs) are the number of reads that contributed to a given consensus sequence query. %ARs are scaled by the sum of reads comprising queries with at least one BSP. Results are shown for taxonomic ranks comprising >5% of ARs and the hierarchy is collapsed to the lowest rank with identical results. %ID is mean percent sequence identity, length is the mean alignment length, e-value is the mean expect value, and bitscore is the mean bitscore.

kingdom	phylum	class	order	family	%ARs	%ID	length	e-value	bitscore
Fungi					100	91	496	4^{-29}	712
Fungi	Basidiomycota	Pucciniomycetes	Pucciniales		99	91	492	4^{-29}	705
Fungi	Basidiomycota	Pucciniomycetes	Pucciniales	Melampsoraceae	80	92	501	5^{-29}	742
Fungi	Basidiomycota	Pucciniomycetes	Pucciniales	Pucciniaceae	11	87	425	2^{-35}	497

Paxillus; Basidiomycota; Agaricomycetes; Boletales; Paxillaceae (5395)

Label: positive
Predicted probability: 0.99
Mapped reads: 37,506,486
Mapping rate: 97%
Base pairs queried: 6,840,022
Unique contigs queried: 199
Unique contigs aligned: 64

A nuclear genome assembly for *Paxillus involutus* (48.8 Mb, N50: 10 kb) was included in the Kraken 2 reference database.

Almost all (97%) *Paxillus*-classified reads mapped back to their database sequences. Contig length and mapped reads were positively correlated ($r = 0.67$) and this increased to $r = 0.91$ with the exclusion of the two contigs with unusually few mapped reads (Fig. 40).

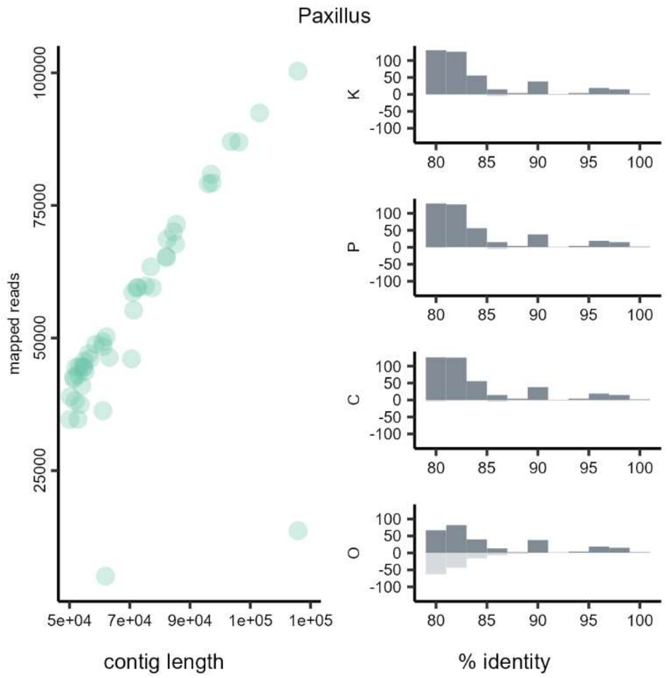

Figure 39. Left: number of mapped reads *vs*. contig length for contigs > 50 kb. Right: mirrored histograms of the sequence identity of aligned reads (ARs) with BLAST best-scoring sequence pairs (BSPs) within (top) or outside (bottom) the kingdom (K), phylum (P), class (C), and order (O) ranks of *Paxillus*. BSPs are shown for the 100 consensus sequences with the highest depth of coverage. ARs are the number of reads covering a given consensus sequence query and are given as *n*-fold differences.

The taxonomic distribution of consensus sequence BSPs were more ambiguous. While most (67%) were with the Boletales, BSPs were also found within Polyporales and Agaricales (Table 56). At the genus rank, 32% of ARs were with *Suillus* (Boletales: Suillaceae) but sequence identities were much higher with alignments in Paxillaceae (90% *vs.* 82%). No more than 1.63% of ARs were with any genus in the Polyporales or Agaricales. While these BLAST results do not provide clear support for the identity of the *Paxillus*-classified reads, they also do not indicate reference contamination. Given the high mapping rate, positive correlation between contig length and mapped reads, and moderate support for an origin within he Boletales from the BLAST results, we consider *Paxillus* to most likely be a true positive genus.

Table 56. Distribution of BLAST best-scoring sequence pairs (BSPs) for the 100 consensus sequences with the highest depth of coverage by taxonomic rank. Aligned reads (ARs) are the number of reads that contributed to a given consensus sequence query. %ARs are scaled by the sum of reads comprising queries with at least one BSP. Results are shown for taxonomic ranks comprising >5% of ARs and the hierarchy is collapsed to the lowest rank with identical results. %ID is mean percent sequence identity, length is the mean alignment length, e-value is the mean expect value, and bitscore is the mean bitscore.

kingdom	phylum	class	order	family	%ARs	%ID	length	e-value	bitscore
Fungi					99	83	384	5^{-30}	417
Fungi	Basidiomycota	Agaricomycetes			98	83	386	6^{-30}	420
Fungi	Basidiomycota	Agaricomycetes	Boletales		67	84	408	6^{-33}	466
Fungi	Basidiomycota	Agaricomycetes	Boletales	Suillaceae	32	82	336	6^{-33}	336

Fungi	Basidiomycota	Agaricomycetes	Boletales	Paxillaceae	11	90	559	1^{-81}	794
Fungi	Basidiomycota	Agaricomycetes	Boletales	Boletaceae	10	87	726	1-^{53}	986
Fungi	Basidiomycota	Agaricomycetes	Boletales	Pisolithaceae	6	82	334	3^{-32}	329
Fungi	Basidiomycota	Agaricomycetes	Agaricales		16	82	374	3-29	361
Fungi	Basidiomycota	Agaricomycetes	Polyporales		11	82	342	7^{-30}	339

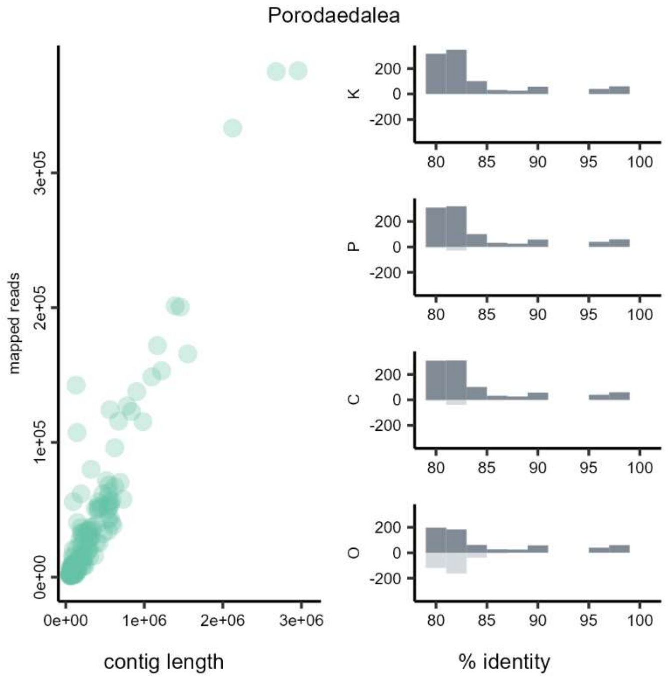

Figure 40. Left: number of mapped reads *vs*. contig length for contigs > 50 kb. Right: mirrored histograms of the sequence identity of aligned reads (ARs) with BLAST best-scoring sequence pairs (BSPs) within (top) or outside (bottom) the kingdom (K), phylum (P), class (C), and order (O) ranks of *Porodaedalea*. BSPs are shown for the 100 consensus sequences with the highest depth of coverage. ARs are the number of reads covering a given consensus sequence query and are given as *n*-fold differences.

***Porodaedalea*; Basidiomycota; Agaricomycetes; Hymenochaetales; Hymenochaetaceae (175857)**

Label: positive
Predicted probability: 0.98
Mapped reads: 7,747,266
Mapping rate: 94%
Base pairs queried: 4,248,871
Unique contigs queried: 50
Unique contigs aligned: 46

Porodaedalea was included in the training data as a positive genus based on occurrence records within 40 km of the aerosol monitoring station. We selected it for further validation because both species in northern Sweden are red-listed and we found a large decline in the γ-diversity contribution of this genus.

A nuclear genome assembly for *Porodaedalea pini* (52.7 Mb, N50: 563 kb) was included in the Kraken 2 reference database. *P. pini* occurs throughout the region, including along the road to the aerosol monitoring station, but *P. chrysoloma* is found much more frequently both locally and throughout northern Sweden. The mapping rate for the *Porodaedalea*-classified reads was high (94%) and the number of reads mapped per contig was correlated with contig length (r = 0.95, Fig. 41).

All BSPs were with fungi and 95% of ARs aligned had their BSPs within Agaricomycetes (Table 57). At the order rank, BSPs representing 66% of ARs were within Hymenochaetales, all of which were further within the Hymenochaetaceae family. Polyporales, an order of morphologically similar taxa, had the second largest number of BSPs (16% of ARs). Work to establish reciprocally monophyletic Polyporales and Hymenochaetales is ongoing (*e.g* Justo et al. 2017; Wang et al. 2023), and this phylogenetic uncertainty likely explains the taxonomic distribution of BSPs.

Table 57. Distribution of BLAST best-scoring sequence pairs (BSPs) for the 100 consensus sequences with the highest depth of coverage by taxonomic rank. Aligned reads (ARs) are the number of reads that contributed to a given consensus sequence query. %ARs are scaled by the sum of reads comprising queries with at least one BSP. Results are shown for taxonomic ranks comprising >5% of ARs and the hierarchy is collapsed to the lowest rank with identical results. %ID is

mean percent sequence identity, length is the mean alignment length, e-value is the mean expect value, and bitscore is the mean bitscore.

kingdom	phylum	class	order	family	%ARs	%ID	length	e-value	bitscore
Fungi					100	83	499	1^{-30}	575
Fungi	Basidiomycota				97	83	504	1^{-30}	580
Fungi	Basidiomycota	Agaricomycetes			95	83	504	1^{-30}	582
Fungi	Basidiomycota	Agaricomycetes	Hymenocha-etales	Hymenochaet-aceae	66	84	555	2^{-30}	679
Fungi	Basidiomycota	Agaricomycetes	Polyporales		16	82	473	3^{-40}	468
Fungi	Basidiomycota	Agaricomycetes	Polyporales	Polyporaceae	8	82	441	7^{-40}	438

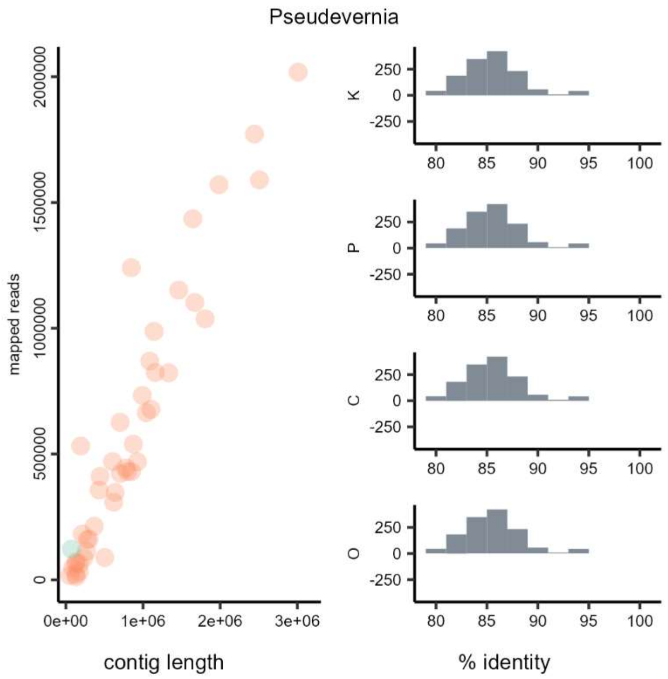

Figure 41. Left: number of mapped reads *vs*. contig length for contigs > 50 kb. Right: mirrored histograms of the sequence identity of aligned reads (ARs) with BLAST best-scoring sequence pairs (BSPs) within (top) or outside (bottom) the kingdom (K), phylum (P), class (C), and order (O) ranks of *Pseudevernia*. BSPs are shown for the 100 consensus sequences with the highest depth of coverage. ARs are the number of reads covering a given consensus sequence query and are given as *n*-fold differences.

Pseudevernia; Ascomycota; Lecanoromycetes; Lecanorales; Parmeliaceae (88743)

Label: unlabeled
Predicted probability: 0.98
Mapped reads: 28,231,746
Mapping rate: 93%
Base pairs queried: 1,279,121
Unique contigs queried: 31
Unique contigs aligned: 22

This genus was flagged for validation because of the abundance of *Pseudevernia*-classified reads and the lack of occurrence records within 40 km of the aerosol monitoring station. However, *Pseudevernia* is well-documented along the Baltic coast in northern Sweden and is abundant further south.

A nuclear genome assembly for *P. furfuracea* (37.1 Mb, N50: 1.2 Mb) was included in the Kraken 2 reference database. Most (93%) *Pseudevernia*-classified reads mapped back to this reference and the number of mapped reads scaled positively with contig length ($r = 0.95$; Fig. 42).

Almost all BSPs were exclusively within the Parmeliaceae family (Table 58). We did not attempt to determine if the *Pseudevernia*-classified reads came from this genus or a relative, but we found no evidence to suggest they originated from either reference contamination or an organism outside of the Parmeliaceae. Therefore, we consider ‘Pseudevernia’ a true biological positive, with the caveat that genera-rank assignments become more uncertain when a lineage is not well-sequenced.

Table 58. Distribution of BLAST best-scoring sequence pairs (BSPs) for the 100 consensus sequences with the highest depth of coverage by taxonomic rank. Aligned reads (ARs) are the number of reads that contributed to a given consensus sequence query. %ARs are scaled by the sum of reads comprising queries with at least one BSP. Results are shown for taxonomic ranks comprising >5% of ARs and the hierarchy is collapsed to the lowest rank with identical results. %ID is mean percent sequence identity, length is the mean alignment length, e-value is the mean expect value, and bitscore is the mean bitscore.

kingdom	phylum	class	order	family	%ARs	%ID	length	e-value	bitscore
Fungi	Ascomycota				100	85	2,413	2^{-42}	2,892
Fungi	Ascomycota	Lecanoromycetes	Lecanorales	Parmeliaceae	99	85	2,444	2^{-42}	2,934

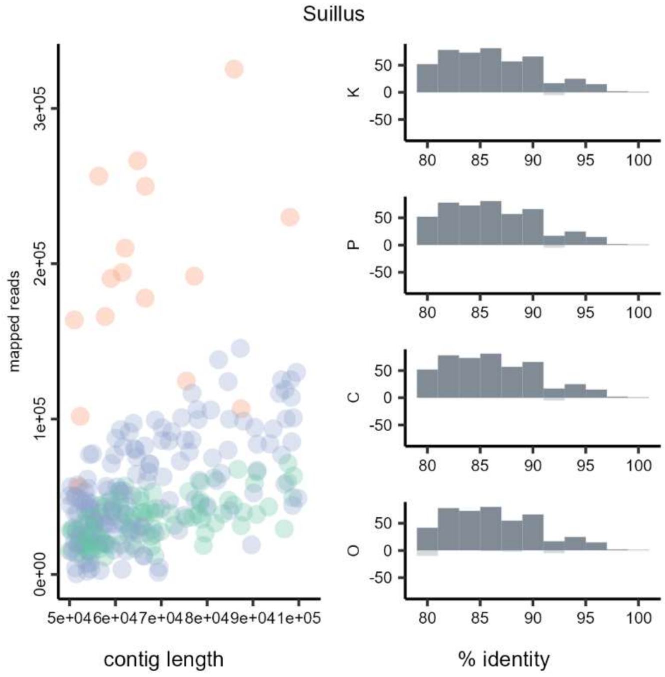

Figure 42. Left: number of mapped reads *vs*. contig length for contigs > 50 kb. Right: mirrored histograms of the sequence identity of aligned reads (ARs) with BLAST best-scoring sequence pairs (BSPs) within (top) or outside (bottom) the kingdom (K), phylum (P), class (C), and order (O) ranks of *Suillus*. BSPs are shown for the 100 consensus sequences with the highest depth of coverage. ARs are the number of reads covering a given consensus sequence query and are given as *n*-fold differences.

***Suillus*; Basidiomycota; Agaricomycetes; Boletales; Suillaceae (5379)**

Label: positive
Predicted probability: 0.99
Mapped reads: 162,035,406
Mapping rate: 89%
Base pairs queried: 1,050,093
Unique contigs queried: 100
Unique contigs aligned: 83

Nuclear genome sequences for *Suillus placidus* (40.1 Mb, N50: 7 kb), *S. spraguei* (88.4 Mb, N50: 11 kb), and *S. alpinus* (37.9 Mb, N50: 6 kb) were included in the Kraken 2 reference database.

Most (89%) *Suillus*-classified reads mapped back to their reference sequences. Of these, the largest fraction of reads (57%) mapped to the *S. placidus* genome, in blue, followed by *S. spraguei* (30%) in green, and *S. alpinus* (12%) in orange (Fig. 43). None of these species occur in Sweden, nor do they seem closely related to the two species common near the aerosol monitoring station, *S. luteus* and *S. variegatus* (Nguyen et al. 2016). Nevertheless, the number of mapped reads per contig was correlated with contig length in all three assemblies, as expected for a true-positive taxon (Fig. 43).

Almost all consensus sequences from the 100 highest coverage regions had BSPs exclusively within Agaricomycetes (Table 59). 96% of the corresponding ARs were within the order Boletales and 90% were also within the Suillaceae family.

Table 59. Distribution of BLAST best-scoring sequence pairs (BSPs) for the 100 consensus sequences with the highest depth of coverage by taxonomic rank. Aligned reads (ARs) are the number of reads that contributed to a given consensus sequence query. %ARs are scaled by the sum of reads comprising queries with at least one BSP. Results are shown for taxonomic ranks comprising >5% of ARs and the hierarchy is collapsed to the lowest rank with identical results. %ID is mean percent sequence identity, length is the mean alignment length, e-value is the mean expect value, and bitscore is the mean bitscore.

kingdom	phylum	class	order	family	%ARs	%ID	length	e-value	bitscore
Fungi	Basidiomycota	Agaricomycetes			99	86	1,502	2^{-32}	1,855
Fungi	Basidiomycota	Agaricomycetes	Boletales		96	86	1,525	2^{-32}	1,884
Fungi	Basidiomycota	Agaricomycetes	Boletales	Suillaceae	90	86	1,558	2^{-32}	1,924

Suillus was one of the genera used to compare the results of the two BLAST approaches we initially considered. Of 100 randomly selected paired-end reads, 99% of alignments were within the Suillaceae family (Table 60).

Table 60. Distribution of BLAST best-scoring sequence pairs (BSPs) by taxonomic rank for 100 randomly-selected pair-end reads. Results are shown for taxonomic ranks comprising >5% of reads and the hierarchy is collapsed to the lowest rank with identical results. %ID is mean percent sequence identity, length is the mean alignment length, e-value is the mean expect value, and bitscore is the mean bitscore.

kingdom	phylum	class	order	family	%BSPs	%ID	length	e-value	bitscore
Fungi	Basidiomycota	Agaricomycetes			100	96	125	2^{-28}	200
Fungi	Basidiomycota	Agaricomycetes	Boletales	Suillaceae	99	96	125	2^{-28}	200

***Russula*; Basidiomycota; Agaricomycetes; Russulales; Russulaceae (5402)**

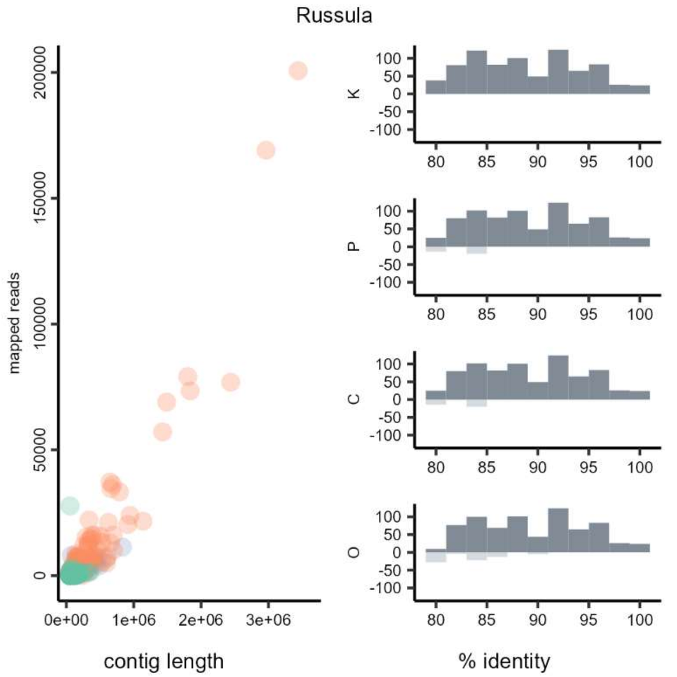

Figure 43. Left: number of mapped reads *vs*. contig length for contigs > 50 kb. Right: mirrored histograms of the sequence identity of aligned reads (ARs) with BLAST best-scoring sequence pairs (BSPs) within (top) or outside (bottom) the kingdom (K), phylum (P), class (C), and order (O) ranks of *Russula*. BSPs are shown for the 100 consensus sequences with the highest depth of coverage. ARs are the number of reads covering a given consensus sequence query and are given as *n*-fold differences.

Label: positive
Predicted probability: 0.99
Mapped reads: 4,313,644
Mapping rate: 85%
Base pairs queried:490,973
Unique contigs queried: 70
Unique contigs aligned:69

Five nuclear genome assemblies were included in the Kraken 2 database: *Russula abietina* (52.7 Mb, N50: 10 kb), *R. foetens* (49.1 Mb, N50: 10 kb), *R. griseocarnosa* (64.2 Mb, N50: 328 kb), *R. lepida* (40.7 Mb, N50: 48 kb), and *R. virescens* (63.7 Mb, N50: 30 kb). Out of these taxa, only *R. foetens* is reported from Norrbotten province in northern Sweden and it is apparently rare (9 of the 1,050 localities reported to Artportalen).

83% of the Russula-classified reads mapped to their sequences in the reference database. Of these, most mapped to *R. griseocarnso* (53%), in orange, followed by *R. abietina* (32%) in green, and *R. lepida* (8%), denoted by the single, admittedly barely visible, blue dot (Fig. 44). More reads tended to map to longer contigs (r = 0.93), as expected from a true-positive taxon.

Most consensus sequences from the 100 highest coverage regions had BSPs exclusively within Agaricomycetes (Table 61). 91% of the corresponding ARs were within the order Russulales and 90% were also within the Russulaceae family.

Table 61. Distribution of BLAST best-scoring sequence pairs (BSPs) for the 100 consensus sequences with the highest depth of coverage by taxonomic rank. Aligned reads (ARs) are the number of reads that contributed to a given consensus sequence query. %ARs are scaled by the sum of reads comprising queries with at least one BSP. Results are shown for taxonomic ranks comprising >5% of ARs and the hierarchy is collapsed to the lowest rank with identical results. %ID is mean percent sequence identity, length is the mean alignment length, e-value is the mean expect value, and bitscore is the mean bitscore.

kingdom	phylum	class	order	family	%ARs	%ID	length	e-value	bitscore
Fungi					100	87	1,187	4^{-29}	1,520
Fungi	Basidiomycota	Agaricomycetes			96	88	1,212	9^{-32}	1,556
Fungi	Basidiomycota	Agaricomycetes	Russulales		91	88	1,234	4^{-90}	1,588
Fungi	Basidiomycota	Agaricomycetes	Russulales	Russulaceae	90	88	1,230	4^{-90}	1,580

Trametes; Basidiomycota; Agaricomycetes; Polyporales; Polyporaceae (5324)

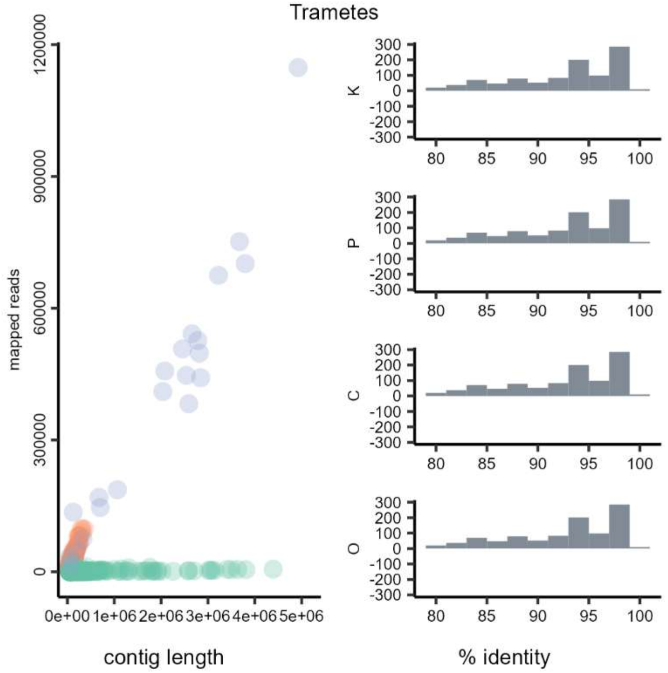

Figure 44. Left: number of mapped reads *vs*. contig length for contigs > 50 kb. Right: mirrored histograms of the sequence identity of aligned reads (ARs) with BLAST best-scoring sequence pairs (BSPs) within (top) or outside (bottom) the kingdom (K), phylum (P), class (C), and order (O) ranks of *Trametes*. BSPs are shown for the 100 consensus sequences with the highest depth of coverage. ARs are the number of reads covering a given consensus sequence query and are given as *n*-fold differences.

Label: positive
Predicted probability: 0.90
Mapped reads: 28,086,424
Mapping rate: 92%
Base pairs queried: 3,653,366
Unique contigs queried: 76
Unique contigs aligned: 34

Reference genomes were available for *Trametes versicolor* (49.5 Mb, N50: 2.8 Mb), in blue, *T. pubescens* (39.4 Mb, N50: 72 kb) in orange, and *T. hirusta* and *T. sanguinea*, both in green in Fig 39. *T. versicolor* is rare in northern Sweden but is closely related to the most abundant species, *T. ochracea* (Justo and Hibbett 2011).

92% of the Trametes-classified reads mapped to their sequences in the reference database. Of these, 52% of reads mapped to the *T. pubescens* assembly, in orange, and 44% of reads to the much more contiguous *T. versicolor* assembly, in blue (Fig. 45). Less than 1% mapped to the *T. hirusta* and *T. sanguinea* assemblies, indicated in green (Fig. 45).

More reads mapped to longer contigs from the *T. versicolor* and *T. pubescens* ($r = 0.59$) assemblies, as expected for a correctly classified positive genus.

All BSPs were exclusively within the Polyporales order and 99% of ARs were furthermore within the Polyporaceae family (Table 62).

Table 62. Distribution of BLAST best-scoring sequence pairs (BSPs) for the 100 consensus sequences with the highest depth of coverage by taxonomic rank. Aligned reads (ARs) are the number of reads that contributed to a given consensus sequence query. %ARs are scaled by the sum of reads comprising queries with at least one BSP. Results are shown for taxonomic ranks comprising >5% of ARs and the hierarchy is collapsed to the lowest rank with identical results. %ID is mean percent sequence identity, length is the mean alignment length, e-value is the mean expect value, and bitscore is the mean bitscore.

kingdom	phylum	class	order	family	%ARs	%ID	length	e-value	bitscore
Fungi	Basidiomycota	Agaricomycetes	Polyporales		100	89	1,185	5^{-51}	1,571
Fungi	Basidiomycota	Agaricomycetes	Polyporales	Polyporaceae	99	89	1,190	5^{-51}	1,579

***Tricholoma*; Basidiomycota; Agaricomycetes; Agaricales; Tricholomataceae (40144)**

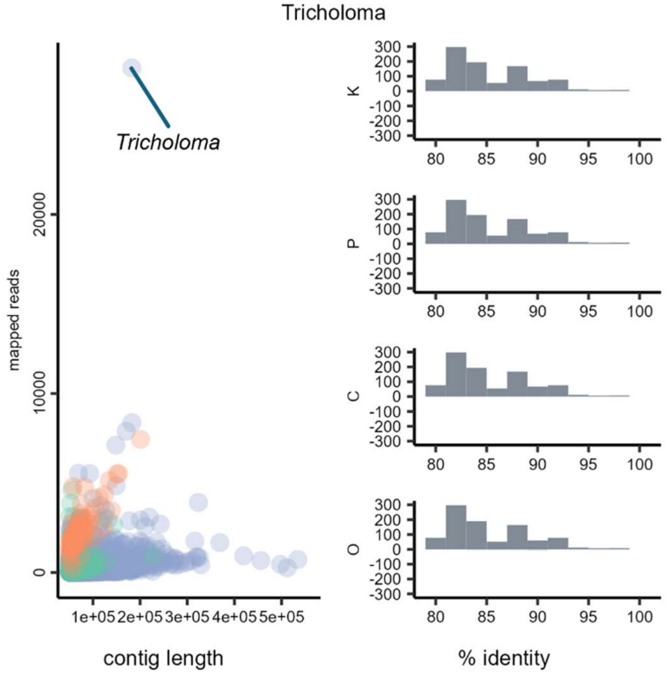

Figure 45. Left: number of mapped reads *vs*. contig length for contigs > 50 kb. Right: mirrored histograms of the sequence identity of aligned reads (ARs) with BLAST best-scoring sequence pairs (BSPs) within (top) or outside (bottom) the kingdom (K), phylum (P), class (C), and order (O) ranks of *Tricholoma*. BSPs are shown for the 100 consensus sequences with the highest depth of coverage. ARs are the number of reads covering a given consensus sequence query and are given as *n*-fold differences.

Label: positive
Predicted probability: 0.99
Mapped reads: 13,819,480
Mapping rate: 73%
Base pairs queried: 360,003
Unique contigs queried: 98
Unique contigs aligned: 82

Three nuclear genome assemblies for *Tricholoma matsutake* (128.0 Mb, N50: 3 kb; 141.5 Mb, N50: 79 kb and 148.9 Mb, N50: 9 kb) were included in the Kraken 2 reference database and a single assembly for *T. bakamatsutake* (136.1 Mb, N50: 10 kb), *T. flavovirens* (115.6 Mb, N50: 8 kb), *T. saponaceum* (54.8 Mb, N50: 12 kb), and *T. terreum* (81.9 Mb, N50: 10 kb). Thirty-one *Tricholoma* species have at least one reported occurrence locality in northern Sweden, but *T. matsutake* accounts for 40% of all records for the genus.

73% of the Tricholoma-classified reads mapped to their sequences in the reference database. Of these, the majority mapped to the *T. matsutake* assemblies (31%), in blue, or *T. flavovirens* (31%), in orange (Fig. 46). 10-16% of reads mapped to the *T. bakamatsutake*, *T. saponaceum*, and *T. terreum* assemblies. Longer contigs in the *T. flavovirens* assembly had more mapped reads ($r = 0.82$), the expected pattern for a positive taxon, but this did not hold for the *T. matsutake* assemblies, either together (in blue), or individually.

Despite the mapping results, nearly all BSPs with the consensus queries were within the Agaricales order (Table 63). 90% of consensus query ARs were within the Tricholomataceae family, all of which were specifically with *Tricholoma*. These results include the contig with extremely high depth for its length, as denoted in the scatterplot.

Table 63. Distribution of BLAST best-scoring sequence pairs (BSPs) for the 100 consensus sequences with the highest depth of coverage by taxonomic rank. Aligned reads (ARs) are the number of reads that contributed to a given consensus sequence query. %ARs are scaled by the sum of reads comprising queries with at least one BSP. Results are shown for taxonomic ranks comprising >5% of ARs and the hierarchy is collapsed to the lowest rank with identical results. %ID is mean percent sequence identity, length is the mean alignment length, e-value is the mean expect value, and bitscore is the mean bitscore.

kingdom	phylum	class	order	family	%ARs	%ID	length	e-value	bitscore
Fungi	Basidiomycota	Agaricomycetes			100	85	1,234	1^{-30}	1,475
Fungi	Basidiomycota	Agaricomycetes	Agaricales		98	85	1,215	1^{-30}	1,447
Fungi	Basidiomycota	Agaricomycetes	Agaricales	Tricholomataceae	90	85	1,216	1^{-30}	1,446

Plants

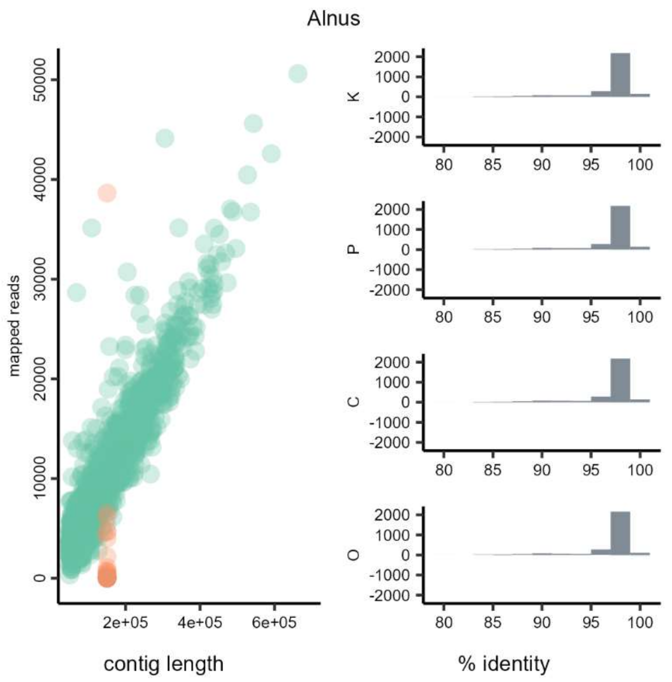

Figure 46. Left: number of mapped reads *vs*. contig length for contigs > 50 kb. Right: mirrored histograms of the sequence identity of aligned reads (ARs) with BLAST best-scoring sequence pairs (BSPs) within (top) or outside (bottom) the kingdom (K), phylum (P), class (C), and order (O) ranks of *Alnus*. BSPs are shown for the 100 consensus sequences with the highest depth of coverage. ARs are the number of reads covering a given consensus sequence query and are given as *n*-fold differences.

***Alnus*; Streptophyta; Magnoliopsida; Fagales; Betulaceae (3515)**

Label: positive
Predicted probability: 0.99
Mapped reads: 54,198,074
Mapping rate: 94%
Base pairs queried: 352,764
Unique contigs queried: 99
Unique contigs aligned: 97

A nuclear genome assembly for *Alnus glutinosa* (506.0 Mb, N50: 75 kb) was available for the Kraken 2 database. *A. incana* is more common in the interior of central and northern Sweden but *A. glutinosa* occurs along the Baltic coast.

Most (94%) of the *Alnus*-classified reads mapped to their reference database sequences. The number of mapped reads per contig was correlated with contig length (r = 0.92), as expected for a true-positive taxon (Fig. 47).

Chloroplast genome sequences were also available for several other species; reads mapped to these genomes are indicated in orange in Fig 41. Of these, most reads mapped the genome from *A. nepalensis*, a species from the Himalayas.

Most BSPs for the consensus sequences were within the Betulaceae family, and 93% of corresponding reads aligned within *Alnus* (Table 64). About 3% of ARs aligned with arthropods, split equally between aphids (Insecta: Hemiptera) and mites (Arachnida: Trombidiformes).

Table 64. Distribution of BLAST best-scoring sequence pairs (BSPs) for the 100 consensus sequences with the highest depth of coverage by taxonomic rank. Aligned reads (ARs) are the number of reads that contributed to a given consensus sequence query. %ARs are scaled by the sum of reads comprising queries with at least one BSP. Results are shown for taxonomic ranks comprising >5% of ARs and the hierarchy is collapsed to the lowest rank with identical results. %ID is mean percent sequence identity, length is the mean alignment length, e-value is the mean expect value, and bitscore is the mean bitscore.

kingdom	phylum	class	order	family	%ARs	%ID	length	e-value	bitscore
Viridiplantae	Streptophyta				97	95	1,029	2^{-68}	1,589
Viridiplantae	Streptophyta	Magnoliopsida			96	95	1,031	2^{-68}	1,592
Viridiplantae	Streptophyta	Magnoliopsida	Fagales	Betulaceae	94	95	1,055	3^{-78}	1,625

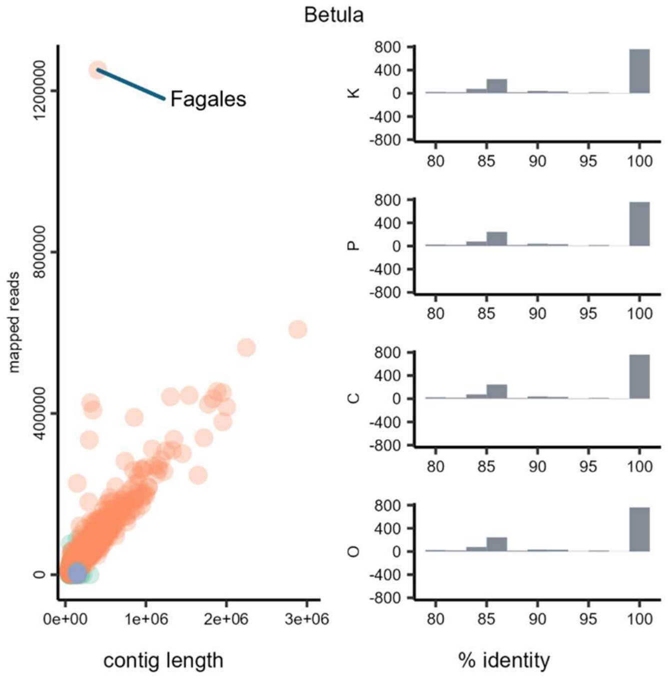

Figure 47. Left: number of mapped reads *vs*. contig length for contigs > 50 kb. Right: mirrored histograms of the sequence identity of aligned reads (ARs) with BLAST best-scoring sequence pairs (BSPs) within (top) or outside (bottom) the kingdom (K), phylum (P), class (C), and order (O) ranks of *Betula*. BSPs are shown for the 100 consensus sequences with the highest depth of coverage. ARs are the number of reads covering a given consensus sequence query and are given as *n*-fold differences.

Betula; Streptophyta; Magnoliopsida; Fagales; Betulaceae (3504)

Label: positive
Predicted probability: 0.98
Mapped reads: 210,999,702
Mapping rate: 89%
Base pairs queried: 4,200,332
Unique contigs queried: 71
Unique contigs aligned: 25

Nuclear genome assemblies for *Betula pendula* (390.3 Mb, N50: 220 kb) and *B. nana* (472.7 Mb, N50: 17 kb) were included in the Kraken 2 reference database. *B. pendula* is common throughout much of Eurasia but is replaced by the downy birch, *B. pubescens,* in northern Fennoscandia. *B. nana* is common on the alpine/arctic tundra and in wetlands throughout Fennoscandia.

89% of the *Betula*-classified reads mapped to their reference database sequences. Of these, 58% mapped to the *B. pendula,* indicated in orange in Fig. 48. While 42% of the reads mapped to *B. nana*, this assembly was less contiguous and most contigs (in green) are not shown in the scatterplot in Fig 48.

Reads mapped to the *B. pendula* genome showed a positive correlation (r = 0.86) with contig length, as expected for a true-positive taxon, with the exception of one conspicuous outlier. We queried 1.4 Mb of consensus sequence divided into 3,000 bp non-overlapping segments from reads mapped to this scaffold (FXXK01000142.1) against the BLAST nt database and found all BSPs were within Magnoliopsida, 91% were within Fagales, and 79% were within the Betulaceae (Table 65). Despite the unusually high read depth, these results do not suggest reference contamination and we conclude the reads mapped to this scaffold likely originated from *Betula*.

BSPs for the 100 highest depth consensus queries were almost entirely within Betulaceae. At the genus level, 61% of reads aligned with *Betula* and 29% with *Corylus*. However, average sequence identity with *Betula* was 98%, compared to 87% with *Corylus.* These *Corylus* alignments are likely due to the number of alignments we considered per query (five BSPs) and the taxonomic composition of the nt database.

Table 65. Distribution of BLAST best-scoring sequence pairs (BSPs) for the 100 consensus sequences with the highest depth of coverage by taxonomic rank. Aligned reads (ARs) are the number of reads that contributed to a given consensus

sequence query. %ARs are scaled by the sum of reads comprising queries with at least one BSP. Results are shown for taxonomic ranks comprising >5% of ARs and the hierarchy is collapsed to the lowest rank with identical results. %ID is mean percent sequence identity, length is the mean alignment length, e-value is the mean expect value, and bitscore is the mean bitscore.

kingdom	phylum	class	order	family	%ARs	%ID	length	e-value	bitscore
Viridiplantae	Streptophyta				100	88	1,465	4^{-28}	1,908
Viridiplantae	Streptophyta	Magnoliopsida	Fagales		99	88	1,487	5^{-28}	1,935
Viridiplantae	Streptophyta	Magnoliopsida	Fagales	Betulaceae	98	89	1,495	5^{-28}	1,952

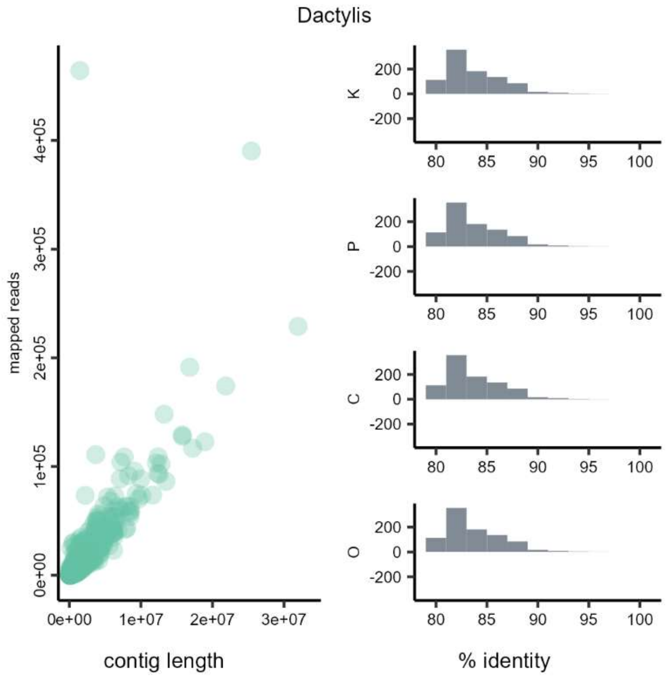

Figure 48. Left: number of mapped reads *vs*. contig length for contigs > 50 kb. Right: mirrored histograms of the sequence identity of aligned reads (ARs) with BLAST best-scoring sequence pairs (BSPs) within (top) or outside (bottom) the kingdom (K), phylum (P), class (C), and order (O) ranks of *Dactylis*. BSPs are shown for the 100 consensus sequences with the highest depth of coverage. ARs are the number of reads covering a given consensus sequence query and are given as *n*-fold differences.

***Dactylis*; Streptophyta; Magnoliopsida; Poales; Poaceae (4508)**

Label: unlabeled
Predicted probability: 0.81
Mapped reads: 38,128,670
Mapping rate: 77%
Base pairs queried: 522,271
Unique contigs queried: 83
Unique contigs aligned: 72

We flagged *Dactylis* for validation because of its abundance and the lack of occurrence records within 40 km of the aerosol monitoring station. Two recent (2021 and 2022) observations occurred about 80 km south, but most records are closer to the Baltic coast. Two nuclear genome assemblies for *D. glomerata* (1.7 Gb, N50: 3.3 Mb and 2.5 Gb, N50: 3 kb) were included in the Kraken 2 reference database.

77% of the Dactylis-classified reads mapped to their sequences in the reference database. The number of mapped reads per contig was correlated with contig length ($r = 0.80$), as expected for a true-positive taxon (Fig. 49).

All consensus query BSPs were within the Poaceae family (Table 66). *Dactylis* was also used to compare the results of directly querying reads against the nt databases *vs.* the mapped consensus approached we applied to all genera. Of 100 randomly-selected paired end reads, 100% of alignments were within the Poaceae family (Table 67).

Table 66. Distribution of BLAST best-scoring sequence pairs (BSPs) for the 100 consensus sequences with the highest depth of coverage by taxonomic rank. Aligned reads (ARs) are the number of reads that contributed to a given consensus sequence query. %ARs are scaled by the sum of reads comprising queries with at least one BSP. Results are shown for taxonomic ranks comprising >5% of ARs and the hierarchy is collapsed to the lowest rank with identical results. %ID is

mean percent sequence identity, length is the mean alignment length, e-value is the mean expect value, and bitscore is the mean bitscore.

kingdom	phylum	class	order	family	%ARs	%ID	length	e-value	bitscore
Viridiplantae	Streptophyta	Magnoliopsida	Poales	Poaceae	100	84	777	4^{-34}	848

Whie we did not attempt to determine if the *Dactylis*-classified reads originate from their assigned genus, these results show that they are at least from a related taxon.

Table 67 Distribution of BLAST best-scoring sequence pairs (BSPs) by taxonomic rank for 100 randomly-selected pair-end reads. Results are shown for taxonomic ranks comprising >5% of reads and the hierarchy is collapsed to the lowest rank with identical results. %ID is mean percent sequence identity, length is the mean alignment length, e-value is the mean expect value, and bitscore is the mean bitscore.

kingdom	phylum	class	order	family	%BSPs	%ID	length	e-value	bitscore
Viridiplantae	Streptophyta	Magnoliopsida	Poales	Poaceae	100	91	131	2^{-27}	180

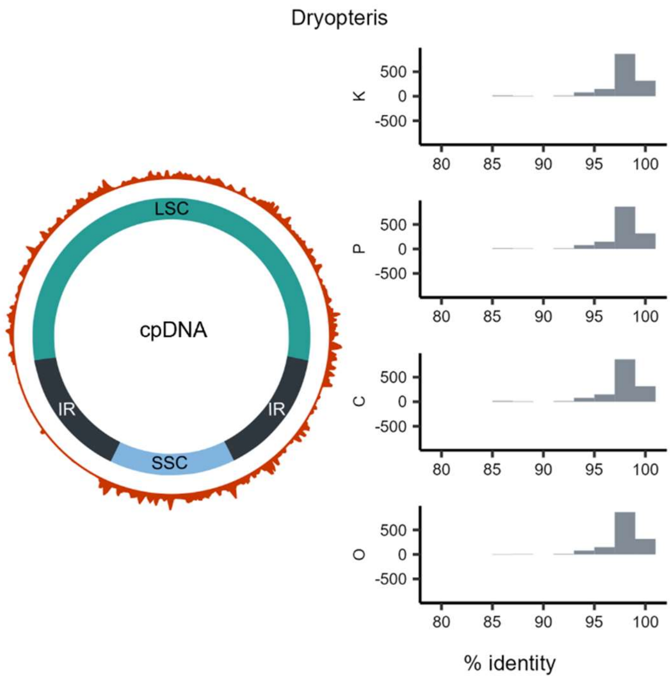

Figure 49. Left: the outer, red ring shows the mean depth (500 bp moving window) of reads mapped to the *Dryopteris* chloroplast genome (cpDNA) and the inner ring indicates the position of the large single copy (LSC), small single copy (SSC) and inverted repeat (IRs) regions. Right: mirrored histograms of the sequence identity of aligned reads (ARs) with BLAST best-scoring sequence pairs (BSPs) within (top) or outside (bottom) the kingdom (K), phylum (P), class (C), and order (O) ranks of *Dryopteris*. BSPs are shown for the 100 consensus sequences with the highest depth of coverage. ARs are the number of reads covering a given consensus sequence query and are given as *n*-fold differences.

***Dryopteris*; Streptophyta; Polypodiopsida; Polypodiales; Dryopteridaceae (3287)**

Label: positive
Predicted probability: 0.99
Mapped reads: 596,760
Mapping rate: 88%
Base pairs queried: 266,981
Unique contigs queried: 18
Unique contigs aligned: 18

No nuclear genome assembly for *Dryopteris* was available for the Kraken 2 reference database, but chloroplast genomes from *D. blanfordii, D. decipiens*, *D. filix-mas*, *D. fragrans*, and *D. villarii* were included.

A plurality of reads mapped to the *D. filix-mas* chloroplast genome (31%) and 12-15% of reads mapped to each of the other four genomes. Depth of coverage across the *D. filix-mas* genome is shown in Fig. 50, with annotations for the large single copy (LSC), small single copy (SSC) and inverted repeat regions (IR). For a well-behaved true positive genus, reads should map continuously and

with similar depth over the LSC and SSC regions and be concentrated in one of the two IRs. This is indeed the pattern found in *Dryopteris*.

Almost all BSPs for the consensus queries were within the Polypodiales order, and almost all of these were within the Dryopteridaceae family (Table 68). Furthermore, all the BSPs within Dryopteridaceae were with *Dryopteris* specifically.

Table 68. Distribution of BLAST best-scoring sequence pairs (BSPs) for the 100 consensus sequences with the highest depth of coverage by taxonomic rank. Aligned reads (ARs) are the number of reads that contributed to a given consensus sequence query. %ARs are scaled by the sum of reads comprising queries with at least one BSP. Results are shown for taxonomic ranks comprising >5% of ARs and the hierarchy is collapsed to the lowest rank with identical results. %ID is mean percent sequence identity, length is the mean alignment length, e-value is the mean expect value, and bitscore is the mean bitscore.

kingdom	phylum	class	order	family	%ARs	%ID	length	e-value	bitscore
Viridiplantae	Streptophyta	Polypodiopsida			100	96	1,374	0.0	2,273
Viridiplantae	Streptophyta	Polypodiopsida	Polypodiales		99	96	1,361	0.0	2,266
Viridiplantae	Streptophyta	Polypodiopsida	Polypodiales	Dryopteridaceae	96	97	1,355	0.0	2,284

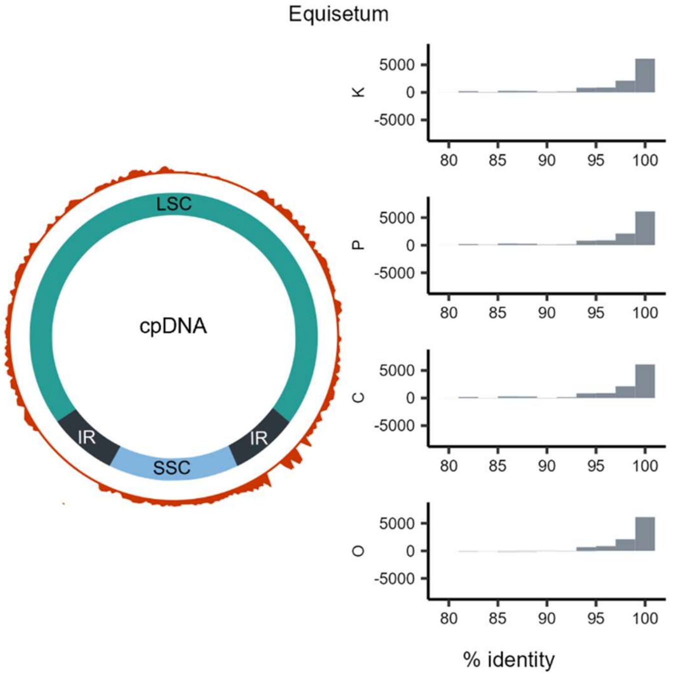

Figure 50. Left: the outer, red ring shows the mean depth (500 bp moving window) of reads mapped to the *Equisetum* chloroplast genome (cpDNA) and the inner ring indicates the position of the large single copy (LSC), small single copy (SSC) and inverted repeat (IRs) regions. Right: mirrored histograms of the sequence identity of aligned reads (ARs) with BLAST best-scoring sequence pairs (BSPs) within (top) or outside (bottom) the kingdom (K), phylum (P), class (C), and order (O) ranks of *Equisetum*. BSPs are shown for the 100 consensus sequences with the highest depth of coverage. ARs are the number of reads covering a given consensus sequence query and are given as *n*-fold differences.

***Equisetum*; Streptophyta; Polypodiopsida; Equisetales; Equisetaceae (3257)**

Label: positive
Predicted probability:0.99
Mapped reads: 47,585,894
Mapping rate: 94%
Base pairs queried: 100,959
Unique contigs queried: 94
Unique contigs aligned: 94

No nuclear genome assembly for *Equisetum* was available for the Kraken 2 reference database, but chloroplast genomes from *E. arvense* and *E. hyemale* were included. Eight *Equisetum* species have been reported near the aerosol monitoring station, of which *E. fluviatile* is the most common (n = 701), but *E. arvense* (n = 284) and *E. hyemele* (n = 26) also occur.

82% of reads mapped to the *E. arvense* chloroplast genome. Depth of coverage across chloroplast genome is shown in Fig. 51 and the large single copy (LSC), small single copy (SSC) and inverted repeat regions (IR) are annotated. Like *Dryopteris,* reads mapped over the LSC

and SSC regions with similar depth and were concentrated in one copy of the IR. Read depth on the LSC was lower in the large and hypervariable trnE-trnY intergenic spacer (Kim and Kim 2014), which is also unsurprising. Overall, these mapping results are consistent with true positive genus.

Almost all BSPs with the consensus queries were within Polypodiopsida (Table 59). All BSPs within Equisetales were with *Equisetum* specifically. Alignments within Polypoidiopsida but outside of the Equisetales were largely within the Psilotales (3% ARs), Osmundales (2% ARs), and the Marattiales (1% ARs). These alignments had much lower sequence identity (87%) and most likely result from conserved sequences among Polypodiopsida chloroplast genomes.

Table 69. Distribution of BLAST best-scoring sequence pairs (BSPs) for the 100 consensus sequences with the highest depth of coverage by taxonomic rank. Aligned reads (ARs) are the number of reads that contributed to a given consensus sequence query. %ARs are scaled by the sum of reads comprising queries with at least one BSP. Results are shown for taxonomic ranks comprising >5% of ARs and the hierarchy is collapsed to the lowest rank with identical results. %ID is mean percent sequence identity, length is the mean alignment length, e-value is the mean expect value, and bitscore is the mean bitscore.

kingdom	phylum	class	order	family	%ARs	%ID	length	e-value	bitscore
Viridiplantae	Streptophyta				100	96	834	8^{-52}	1,380
Viridiplantae	Streptophyta	Polypodiopsida			99	97	844	8^{-52}	1,409
Viridiplantae	Streptophyta	Polypodiopsida	Equisetales	Equisetaceae	89	98	829	4^{-77}	1,431

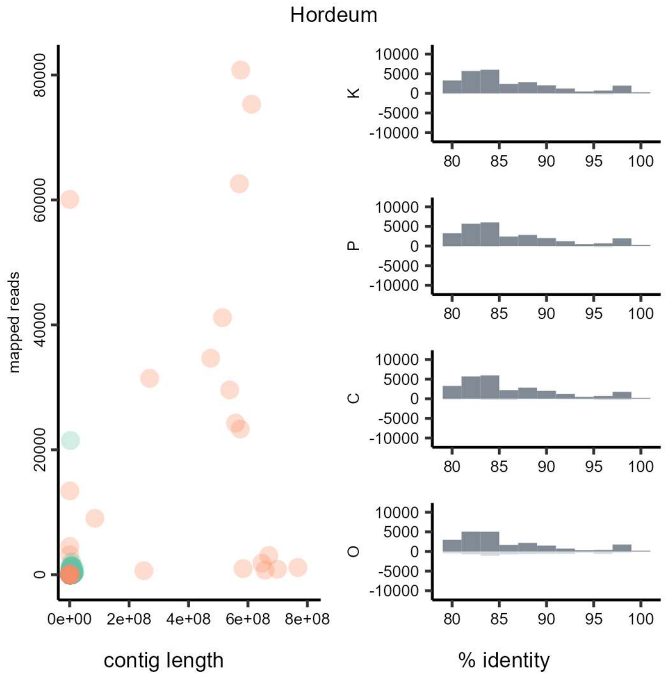

Figure 51. Left: number of mapped reads *vs*. contig length for contigs > 50 kb. Right: mirrored histograms of the sequence identity of aligned reads (ARs) with BLAST best-scoring sequence pairs (BSPs) within (top) or outside (bottom) the kingdom (K), phylum (P), class (C), and order (O) ranks of *Hordeum*. BSPs are shown for the 1,000 consensus sequences with the highest depth of coverage. ARs are the number of reads covering a given consensus sequence query and are given as *n*-fold differences.

Hordeum; Streptophyta; Magnoliopsida; Poales; Poaceae (4512)

Label: unlabeled
Predicted probability: 0.88
Mapped reads: 18,055,068
Mapping rate: 84%
Base pairs queried: 1,335,339
Unique contigs queried: 756
Unique contigs aligned: 510

We selected *Hordeum* for validation because of its abundance and lack of occurrence reports within 40 km of the aerosol monitoring station. Less than 0.5% of Norrbotten county is arable land and most of this is in leys. However, *Hordeum jubatum* is naturalized along the Gulf of Bothnia and has been reported 80 km south of the aerosol monitoring station.

Seventeen partial or full-length nuclear genome assemblies for *Hordeum* were included in the Kraken 2 reference database: 10 for *H. vulgare* subsp. *vulgare* (six partial), five for *H. vulgare* (three partial and one twice the expected 4 Gb size), and one for *H. bulbosum* and *H. pubiflorum*. Most importantly, the three partial *H. vulgare* assemblies were actually metagenomes from the *Hordeum* root-soil interface (CEGK00000000.1, CEGL00000000.1, CEGM000000000.1). While these projects were correctly identified as metagenomic in the BioProject and BioSample databases, they are still listed as *Hordeum* whole-genome shotgun sequences in Genbank and were thus erroneously included in the Kraken 2 database.

84% of the *Hordeum*-classified reads mapped to their reference database sequences. Of these, a plurality of reads (47%) mapped to *H. pubiflorum*, which shares a more recent common ancestor with *H. jubatum* than *H. vulgare* (Brassac and Blattner 2015) but this assembly was highly fragmented (1.4 Gb, N50: 2 kb) and only a single contig was longer than 50 kb. The remaining reads were largely divided between the *H. vulgare* rhizosphere metagenomes (29%) and the *H. vulgare* subsp. *vulgare* (15%) assemblies. The scatterplot in Fig. 52 shows reads mapped to contigs > 50 kb, which were mostly from *H. vulgare* subsp. *vulgare* assemblies, in orange, and all other taxa are shown in green. The number of reads mapped to the *H. vulgare* subsp. *vulgare* contigs was not correlated with contig length. However, *H. jubatum* is tetraploid, whereas *H. vulgare* is diploid, which may make this criterion uninformative for distinguishing true from spurious genera.

In our initial BLAST search of the top 100 consensus sequences by depth, 17% of reads aligned within the Betulaceae family (Magnoliopsida: Fagales) and 74% of reads aligned within the

Poaceae (Table 70). We then expanded the analysis to the top 1,000 sequences by depth of coverage. The results for this expanded BLAST search were similar, with 77% of reads aligned within the Poaceae family and 16% within Betulaceae.

Overall, we found evidence for a non-Poales origin of 83 from 13 different assemblies. Reads mapped to the three metagenomic sequences were unsurprisingly similar to common soil bacteria (e.g. *Sphingomonas*), a diverse group of saprotrophic and mycorrhizal fungi, *Pinus* and bryophytes. These metagenomes, however, did not contribute to the *ca*. 16% of *Hordeum*-classified reads that likely originated from the Betulaceae family. We found Betulaceae-like contigs in four assemblies, which were all released by the same institution, including one of the chromosome-level assemblies (CABEFD01). Unusually for the genera examined here, the likely source of the majority of misclassified reads in *Hordeum* was a 670 Mb scaffold. Consensus queries were BSPs within Poaceae and Betulaceae were spatially segregated, possibly indicating this contaminant was introduced during the scaffolding procedures.

Table 70. Distribution of BLAST best-scoring sequence pairs (BSPs) for the 1,000 consensus sequences with the highest depth of coverage by taxonomic rank. Aligned reads (ARs) are the number of reads that contributed to a given consensus sequence query. %ARs are scaled by the sum of reads comprising queries with at least one BSP. Results are shown for taxonomic ranks comprising >5% of ARs and the hierarchy is collapsed to the lowest rank with identical results. %ID is mean percent sequence identity, length is the mean alignment length, e-value is the mean expect value, and bitscore is the mean bitscore.

kingdom	phylum	class	order	family	%ARs	%ID	length	e-value	bitscore
Viridiplantae	Streptophyta				96	86	648	6^{-29}	795
Viridiplantae	Streptophyta	Magnoliopsida			94	86	654	6^{-29}	802
Viridiplantae	Streptophyta	Magnoliopsida	Poales		78	86	621	6^{-29}	762
Viridiplantae	Streptophyta	Magnoliopsida	Poales	Poaceae	77	85	580	7^{-29}	695
Viridiplantae	Streptophyta	Magnoliopsida	Fagales	Betulaceae	16	87	974	7^{-31}	1,198

Hordeum was used to compare the results from simply querying reads against the nt database *vs*. the consensus sequence approach we ultimately used for validation. Of the 100 randomly selected paired-end reads, 81% aligned with Poaceae sequences and the largest and 10% from prokaryotes, mostly the α-proteobacteria (Table 71). These two methods both indicate a misclassification rate of *ca*. 20%, although they differ in the identity of the misclassified reads. Therefore, we expect the true misclassification rate for the *Hordeum* reads to be close to 20%, which although higher than many of the other genera we inspected, nevertheless suggests 80% of the reads likely originated from *Hordeum* or a related genus. We considered this level of confidence adequate to consider *Hordeum* as a true positive genus for the specific analyses conducted in this paper, but we acknowledge other applications may require more precise read classifications.

Table 71. Distribution of BLAST best-scoring sequence pairs (BSPs) by taxonomic rank for 100 randomly-selected pair-end reads. Results are shown for taxonomic ranks comprising >5% of reads and the hierarchy is collapsed to the lowest rank with identical results. %ID is mean percent sequence identity, length is the mean alignment length, e-value is the mean expect value, and bitscore is the mean bitscore.

kingdom	phylum	class	order	family	%BSPs	%ID	length	e-value	bitscore
Viridiplantae	Streptophyta	Magnoliopsida	Poales		84	90	133	1^{-27}	179

Viridiplantae	Streptophyta	Magnoliopsida	Poales	Poaceae	81	90	132	1^{-27}	178
Prokaryota					10	85	143	6^{-27}	160
Prokaryota	Pseudomonadota	α-proteobacteria			6	82	144	8^{-27}	146

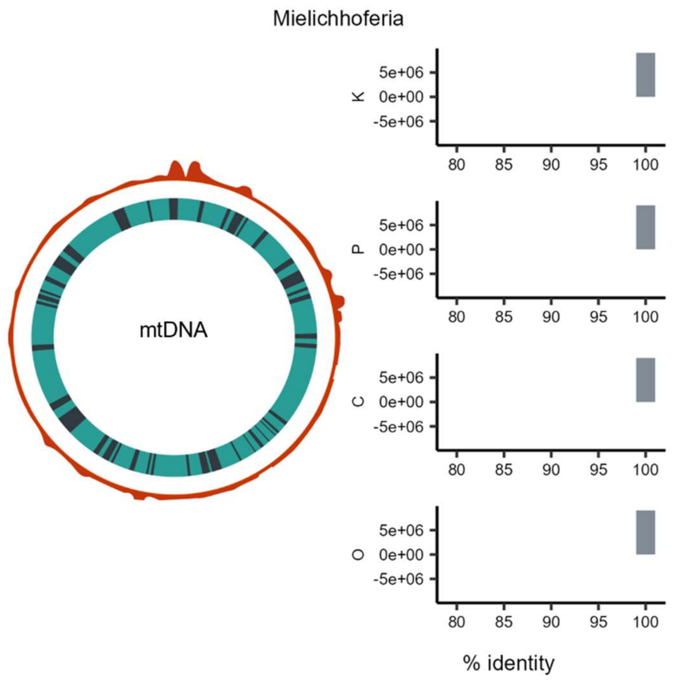

Figure 52. Left: outer, red ring shows the mean depth (500 bp moving window) of reads mapped to the *Mielichhoferia* mitochondrial genome (mtDNA) and the inner ring indicates genic (green) and intergenic (dark grey) regions. Right: mirrored histograms of the sequence identity of aligned reads (ARs) with BLAST best-scoring sequence pairs (BSPs) within (top) or outside (bottom) the kingdom (K), phylum (P), class (C), and order (O) ranks of *Mielichhoferia*. BSPs are shown for the 100 consensus sequences with the highest depth of coverage. ARs are the number of reads covering a given consensus sequence query and are given as *n*-fold differences.

***Mielichhoferia*; Streptophyta; Bryopsida; Bryales; Mniaceae (67233)**

Label: unlabeled
Predicted probability: 0.99
Mapped reads: 7,232,088
Mapping rate: 99%
Base pairs queried: 85734
Unique contigs queried: 26
Unique contigs aligned: 12

Mielichhoferia was selected for validation because the two species in Sweden are classified either as vulnerable (*M. elongata*) or endangered (*M. mielichhoferiana*). The closest reported observations of both species were made *ca*. 70 km northwest of the aerosol monitoring station.

A mitochondrial genome assembly for *M. eleongata* (100 kb) was included in the Kraken 2 reference database. Plant mitochondrial genomes are often large and structurally dynamic (Sullivan, et al. 2019) but those of bryophytes appear to more conserved, gene-rich, and structurally stable (Dong and Liu 2021). Reads did not map evenly across the assembly and were particularly concentrated in a few genic regions, primarily nad9, atp9, nad3, rpl6-rps13 and sdh3 (Fig 47). These genes may be present in higher copy number due to the formation of subgenomic molecules (Woloszynska 2010), although this phenomenon does not seem common in bryophytes (*e.g.* Knoop 2013). Nevertheless, 95% of the assembly had at least 1× coverage.

Consensus sequences unambiguously aligned within the Mniaceae family (Table 72). At the genus level, these alignments were predominately with one of two *Pohlia* complete mitochondrial genomes. These two genomes, along with the *Mielichhoferia* assembly included in the Kraken 2 reference database, are the only released mitochondrial genomes for Mniaceae. If we excluded alignments to the *Pohlia* mitochondrial genomes, then 43% of reads aligned to other *Pohlia* sequences (%ID = 95), 27% to other *Mielichhoferia* sequences (%ID = 98), and 12% within *Plagiomnium* (%ID = 98). While we would strongly recommend developing a larger

library for the Mniaceae before using *Mielichhoferia*-classified reads to inform management and conservation actions, they appear correctly classified insofar as the available reference sequences permit.

Table 72. Distribution of BLAST best-scoring sequence pairs (BSPs) for the 100 consensus sequences with the highest depth of coverage by taxonomic rank. Aligned reads (ARs) are the number of reads that contributed to a given consensus sequence query. %ARs are scaled by the sum of reads comprising queries with at least one BSP. Results are shown for taxonomic ranks comprising >5% of ARs and the hierarchy is collapsed to the lowest rank with identical results. %ID is mean percent sequence identity, length is the mean alignment length, e-value is the mean expect value, and bitscore is the mean bitscore.

kingdom	phylum	class	order	family	%ARs	%ID	length	e-value	bitscore
Viridiplantae	Streptophyta	Bryopsida	Bryales	Mniaceae	100	94	700	5^{-29}	1,192

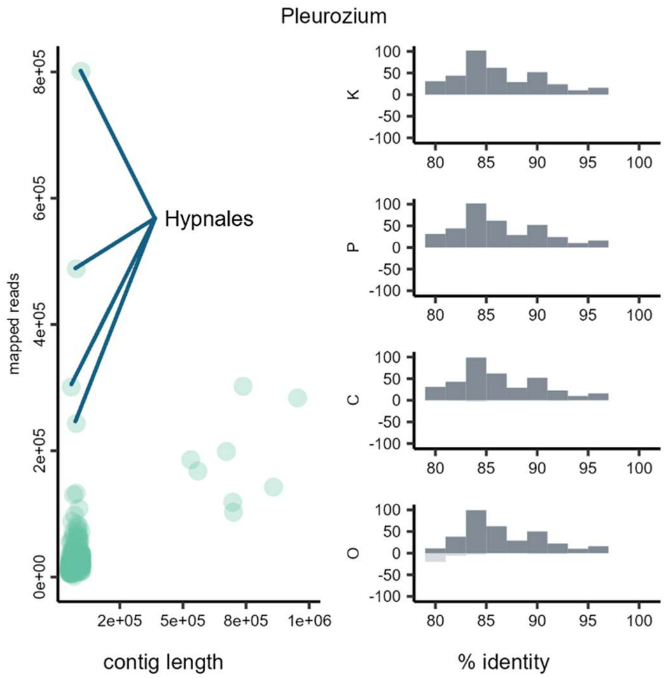

Figure 53. Left: number of mapped reads *vs*. contig length for contigs > 50 kb. Right: mirrored histograms of the sequence identity of aligned reads (ARs) with BLAST best-scoring sequence pairs (BSPs) within (top) or outside (bottom) the kingdom (K), phylum (P), class (C), and order (O) ranks of *Pleurozium*. BSPs are shown for the 100 consensus sequences with the highest depth of coverage. ARs are the number of reads covering a given consensus sequence query and are given as *n*-fold differences.

Pleurozium; Streptophyta; Bryopsida; Hypnales; Hylocomiaceae (34162)

Label: positive
Predicted probability: 0.84
Mapped reads: 95,015,720
Mapping rate: 71%
Base pairs queried: 3,162,221
Unique contigs queried: 98
Unique contigs aligned: 24

A nuclear genome assembly for *Pleurozium schreberi* (220.0 Mb, N50: 148 kb) was included in the Kraken 2 reference database. *P. schreberi* is widely distributed across the northern hemisphere and is one of the most common mosses in the boreal forest.

71% of the *Pleurozium*-classified reads mapped to their reference database sequences. More reads tended to map to the longer contigs in the *P. schreberi* assembly ($r = 0.42$), however, this correlation was weak due to several contigs in the *ca*. 50 kb range with a disproportionately large number of mapped reads (Fig. 54).

Pleurozium was poorly represented in the nt database, with only 118 sequences, of which 65 were from organelles (August 2024). Representation of the Hylocomiaceae more generally was also poor, with 425 non-*Pleurozium* sequences, of which 209 were from organelles (August 2024). This makes the taxonomic distribution of the consensus query BSPs somewhat less informative, they cannot be expected to be primarily within Hylocomiaceae. Nevertheless, nearly all BSPs were within Bryopsida and 91% of reads aligned within the Hypnales order (Table 73).

At the family rank, most alignments were with four Hypnales genomes sequenced as part of the Wellcome Sanger Tree of Life Programme[3]. Consensus sequences from all contigs with a disproportionately large number of mapped reads also aligned with these Hypnales genomes, as annotated in the scatterplot in Fig 54.

Table 73. Distribution of BLAST best-scoring sequence pairs (BSPs) for the 100 consensus sequences with the highest depth of coverage by taxonomic rank. Aligned reads (ARs) are the number of reads that contributed to a given consensus sequence query. %ARs are scaled by the sum of reads comprising queries with at least one BSP. Results are shown for taxonomic ranks comprising >5% of ARs and the hierarchy is collapsed to the lowest rank with identical results. %ID is mean percent sequence identity, length is the mean alignment length, e-value is the mean expect value, and bitscore is the mean bitscore.

kingdom	phylum	class	order	family	%ARs	%ID	length	e-value	bitscore
Viridiplantae	Streptophyta				100	86	2,261	1^{-120}	2,848
Viridiplantae	Streptophyta	Bryopsida			99	86	2,248	1^{-120}	2,835
Viridiplantae	Streptophyta	Bryopsida	Hypnales		91	86	2,262	1^{-120}	2,855
Viridiplantae	Streptophyta	Bryopsida	Hypnales	Lembophyllaceae	21	86	2,288	0.0	2,902
Viridiplantae	Streptophyta	Bryopsida	Hypnales	Hylocomiaceae	13	86	2,068	2^{-121}	2,591
Viridiplantae	Streptophyta	Bryopsida	Hypnales	Leucodontaceae	13	87	2,459	0.0	3,140
Viridiplantae	Streptophyta	Bryopsida	Hypnales	Brachytheciaceae	12	86	2,734	0.0	3,464
Viridiplantae	Streptophyta	Bryopsida	Hypnales	Climaciaceae	10	88	1,986	7^{-120}	2,613
Viridiplantae	Streptophyta	Bryopsida	Hypnales	Thuidiaceae	7	83	1,932	0.0	2,137
Viridiplantae	Streptophyta	Bryopsida	Hypnales	Neckeraceae	6	87	2,491	0.0	3,201
Viridiplantae	Streptophyta	Bryopsida	Hypnales	Calliergonaceae	9	86	2,469	0.0	3,090
Viridiplantae	Streptophyta	Bryopsida	Pottiales	Pottiaceae	7	85	1,940	0.0	2,404

We also queried 100 randomly-selected paired end reads against the nt database. These results were similar, with alignments distributed across the Hypnales with similar frequencies, sequence identities, and alignment lengths (Table 74). Despite the lack of sequences from the Hylocomiaceae family for comparison, *Pleurozium* is monotypic genus and we expect these reads to originate specifically from *P. schreberi*.

Table 74. Distribution of BLAST best-scoring sequence pairs (BSPs) by taxonomic rank for 100 randomly-selected pair-end reads. Results are shown for taxonomic ranks comprising >5% of reads and the hierarchy is collapsed to the lowest rank with identical results. %ID is mean percent sequence identity, length is the mean alignment length, e-value is the mean expect value, and bitscore is the mean bitscore.

kingdom	phylum	class	order	family	%BSPs	%ID	length	e-value	bitscore
Viridiplantae	Streptophyta				100	93	136	9^{-28}	204
Viridiplantae	Streptophyta	Bryopsida			96	93	136	1^{-27}	204
Viridiplantae	Streptophyta	Bryopsida	Hypnales		93	93	136	1^{-27}	204
Viridiplantae	Streptophyta	Bryopsida	Hypnales	Hylocomiaceae	16	94	133	7^{-31}	203
Viridiplantae	Streptophyta	Bryopsida	Hypnales	Brachytheciaceae	13	94	142	3^{-29}	215
Viridiplantae	Streptophyta	Bryopsida	Hypnales	Calliergonaceae	13	92	133	8^{-29}	190
Viridiplantae	Streptophyta	Bryopsida	Hypnales	Leucodontaceae	13	93	135	2^{-28}	203
Viridiplantae	Streptophyta	Bryopsida	Hypnales	Lembophyllaceae	12	92	132	4^{-27}	191

[3] https://www.sanger.ac.uk/programme/tree-of-life/, last accessed August 20, 2024

Viridiplantae	Streptophyta	Bryopsida	Hypnales	Neckeraceae	9	94	136	2^{-27}	204
Viridiplantae	Streptophyta	Bryopsida	Hypnales	Climaciaceae	9	95	140	2^{-27}	222
Viridiplantae	Streptophyta	Bryopsida	Hypnales	Thuidiaceae	8	93	142	7^{-29}	213

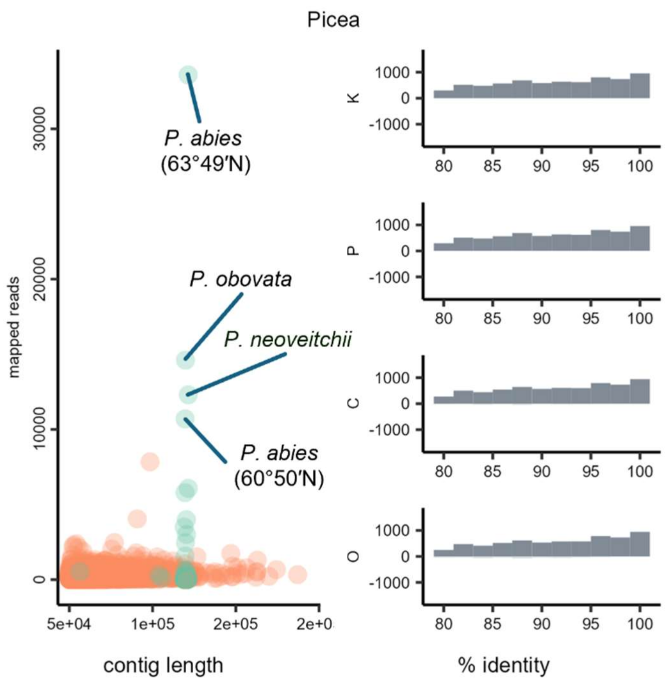

Figure 54. Left: number of mapped reads *vs*. contig length for contigs > 50 kb. Right: mirrored histograms of the sequence identity of aligned reads (ARs) with BLAST best-scoring sequence pairs (BSPs) within (top) or outside (bottom) the kingdom (K), phylum (P), class (C), and order (O) ranks of *Picea*. BSPs are shown for the 1,000 consensus sequences with the highest depth of coverage. ARs are the number of reads covering a given consensus sequence query and are given as *n*-fold differences.

Picea; Streptophyta; Pinopsida; Pinales; Pinaceae (3328)

Label: positive
Predicted probability: 0.87
Mapped reads: 293,201,090
Mapping rate: 84%
Base pairs queried: 2,576,099
Unique contigs queried: 964
Unique contigs aligned: 897

We extracted *Picea*-classified reads from the three weeks with the highest pollen production (1998:27, 2004:26, and 1980:25) and mapped these back to reference database.

Two approximately full-length assemblies for *Picea glauca* were included in the Kraken 2 reference database (19.6 Gb, N50: 6 kb and 21.2 Gb, N50: 8 kb), in addition to a *P. glauca* exome (244.1 Mb, N50: 1 kb), and a partial assembly for *P. abies* (42.0 Mb, N50: 1 kb). *P. abies* and is common across Fennoscandia and most of boreal-Alpine Eurasia, although gene flow from *P. obovata* is detectable in least part of Fennoscandia (Sullivan 2020, Li et al. 2022).

Most (84%) *Picea*-classified reads mapped back to their sequences in the reference database. Of these, a similar fraction mapped to the two *P. glauca* assemblies (42% and 45%), shown in orange in the scatterplot in Fig. 55. Reads mapping to the partial *P. abies* assembly, *P. glauca* exome, and several chloroplast genomes are shown in green. We did not find a positive correlation between mapped read count and contig length, although this not surprising given that long-terminal repeats comprise *ca.* 70% of the *Picea* genome (Sullivan 2020) and the low contiguity of the reference assemblies. Of the chloroplast genome assemblies, the largest number of reads mapped to genomes from a *P. abies* from north-central Sweden, *P. obovata*, *P. neoveitchii,* and a *P. abies* from southern Norway. The large number of reads mapped to the *P. neoveitchii* is surprising (Sullivan et al. 2017; Feng et al. 2018) but the other chloroplast genomes from this taxon do not show the same pattern.

In our initial BLAST search of the top 100 consensus sequences by depth, 26% of reads aligned within *Timema* (Insecta: Phasmatodea) with a mean sequence identity of 91%. Specifically, these alignments were with the *Timema* assemblies we found to be contaminated by *Picea*, as described in the results section for *Timema*. We found no evidence of other contamination after excluding the BSPS with these assemblies, but we expanded the BLAST analysis to the top 1,000 sequences by depth of coverage to confirm that these BSPs had not masked other potential contaminants.

For the 1,000 consensus queries, we found that 97% of their corresponding reads aligned within the class Pinopsida (Table 75. The 3% of reads aligned within Magnoliopsida resulted from alignments to mitochondrial genomes spanning 30 different orders. This genome localization and combined with the diffuse taxonomic distribution of the BSPs is more consistent with a conserved mitochondrial sequence, rather than reference contamination by an angiosperm. Alignments within Pinopsida but outside of Pinaceae resulted from BSPs within Cupressales and Auricarales. At the genus rank, 61% of reads aligned within *Picea*, 12% with its sister clade, *Cathaya*, and 2-8% with the other Pinaceae genera.

Table 75. Distribution of BLAST best-scoring sequence pairs (BSPs) for the 1,000 consensus sequences with the highest depth of coverage by taxonomic rank. Aligned reads (ARs) are the number of reads that contributed to a given consensus sequence query. %ARs are scaled by the sum of reads comprising queries with at least one BSP. Results are shown for taxonomic ranks comprising >5% of ARs and the hierarchy is collapsed to the lowest rank with identical results. %ID is mean percent sequence identity, length is the mean alignment length, e-value is the mean expect value, and bitscore is the mean bitscore.

kingdom	phylum	class	order	family	%ARs	%ID	length	e-value	bitscore
Viridiplantae	Streptophyta				100	92	802	9^{-30}	1,208
Viridiplantae	Streptophyta	Pinopsida			97	92	807	10^{-30}	1,219
Viridiplantae	Streptophyta	Pinopsida	Pinales	Pinaceae	93	92	816	1^{-29}	1,243

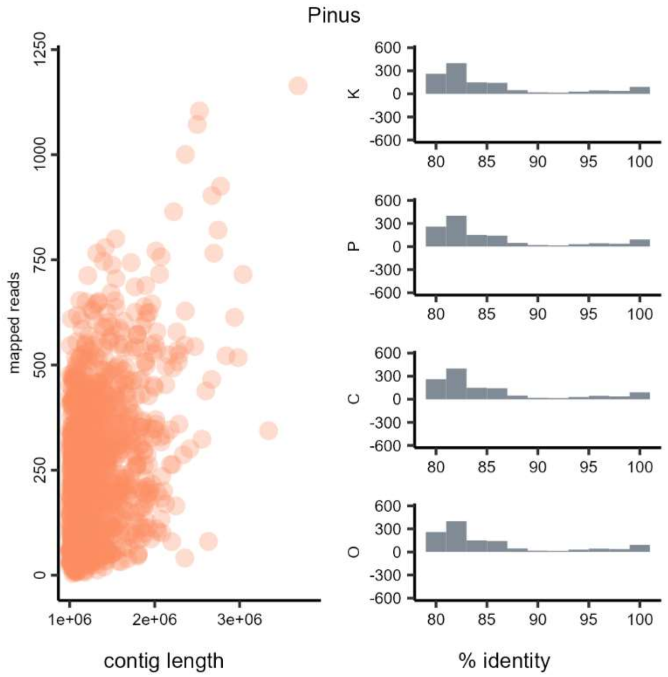

Figure 55. Left: number of mapped reads *vs*. contig length for contigs > 1 Mb. Right: mirrored histograms of the sequence identity of aligned reads (ARs) with BLAST best-scoring sequence pairs (BSPs) within (top) or outside (bottom) the kingdom (K), phylum (P), class (C), and order (O) ranks of *Pleurozium*. BSPs are shown for the 100 consensus sequences with the highest depth of coverage. ARs are the number of reads covering a given consensus sequence query and are given as *n*-fold differences.

***Pinus*; Streptophyta; Pinopsida; Pinales; Pinaceae (3337)**

Label: positive
Predicted probability: 0.92
Mapped reads: 254,682,890
Mapping rate: 77%
Base pairs queried: 1,616,985
Unique contigs queried: 99
Unique contigs aligned: 99

Nuclear genome assemblies for *Pinus taeda* (19.7 Gb, N50: 93 kb) and *P. lambertiana* (24.5 Gb, N50: 270 kb) were included in the Kraken 2 reference database. *Pinus* is divided in two deeply-diverged subgenera (Jin et al. 2021) and *P. taeda*, *P. sylvestris*, the native species in Fennoscandia, and *P. contorta,* a species planted in central and northern Sweden, belong to the subgenus *Pinus*.

We mapped *Pinus*-classified reads from weeks 1980:26, 1990:27, and 2004:28 back to their Kraken 2 reference database sequences. These were within the flowering period of *Pinus* but only 1980:26 was a peak pollen week.

Most reads (97%) mapped to the *P. taeda* assembly, a result consistent with the closer relatedness of the subgenus *Pinus* taxa. The number of reads mapped to a contig was positively correlated with length for the longest contigs (> 1 Mb) but this pattern weakened with shorter contigs (Fig. 56). This result is unsurprising, as conifer genomes mostly comprise long-terminal repeats, an attribute that has limited the contiguity of assemblies until recently (Sullivan 2020), an attribute that has limited the contiguity of assemblies until recently.

All BSPs for the 100 highest-depth consensus sequences were within Pinaceae. BSPs within *Pinus* specifically accounted for 99.3% of the corresponding reads (Table 76).

Table 76. Distribution of BLAST best-scoring sequence pairs (BSPs) for the 100 consensus sequences with the highest depth of coverage by taxonomic rank. Aligned reads (ARs) are the number of reads that contributed to a given consensus sequence query. %ARs are scaled by the sum of reads comprising queries with at least one BSP. Results are shown for taxonomic ranks comprising >5% of ARs and the hierarchy is collapsed to the lowest rank with identical results. %ID is mean percent sequence identity, length is the mean alignment length, e-value is the mean expect value, and bitscore is the mean bitscore.

kingdom	phylum	class	order	family	%ARs	%ID	length	e-value	bitscore
Viridiplantae	Streptophyta	Pinopsida	Pinales	Pinaceae	100	85	1,612	4^{-45}	1,872

Pinus was used to compare the results of directly querying reads against the nt database *vs.* the consensus sequence approach we applied to all genera. Of the 100 randomly-selected paired-end reads, all alignments were within Pinaceae and 99% were with *Pinus* specifically (Table 77).

Table 77. Distribution of BLAST best-scoring sequence pairs (BSPs) by taxonomic rank for 100 randomly-selected pair-end reads. Results are shown for taxonomic ranks comprising >5% of reads and the hierarchy is collapsed to the lowest rank with identical results. %ID is mean percent sequence identity, length is the mean alignment length, e-value is the mean expect value, and bitscore is the mean bitscore.

kingdom	phylum	class	order	family	%BSPs	%ID	length	e-value	bitscore
Viridiplantae	Streptophyta	Pinopsida	Pinales	Pinaceae	100	88	137	2^{-27}	170

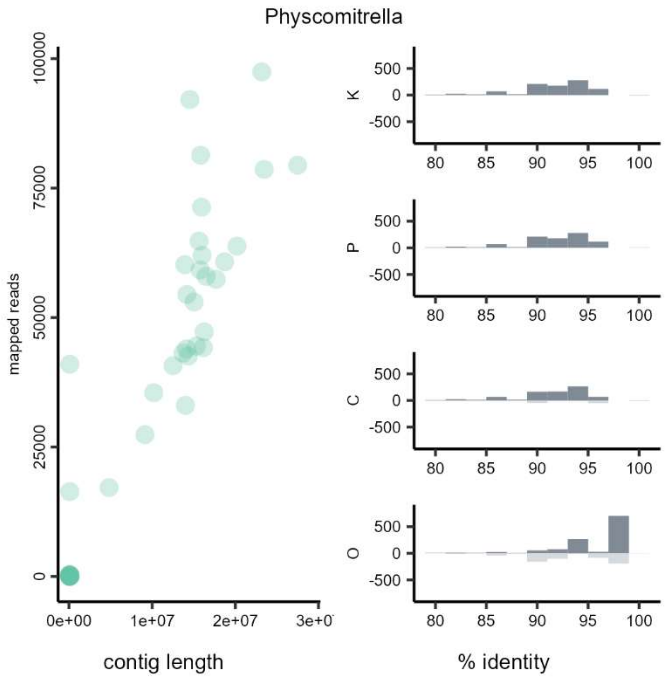

Figure 56. Left: number of mapped reads *vs*. contig length for contigs > 50 kb. Right: mirrored histograms of the sequence identity of aligned reads (ARs) with BLAST best-scoring sequence pairs (BSPs) within (top) or outside (bottom) the kingdom (K), phylum (P), class (C), and order (O) ranks of *Physcomitrella*. BSPs are shown for the 100 consensus sequences with the highest depth of coverage. ARs are the number of reads covering a given consensus sequence query and are given as *n*-fold differences.

Physcomitrella; Streptophyta; Bryopsida; Funariales; Funariaceae (3217)

Label: unlabeled
Predicted probability: 0.99
Mapped reads: 4,975,808
Mapping rate: 72%
Base pairs queried: 268,811
Unique contigs queried: 39
Unique contigs aligned: 38

Physcomitrella was flagged for validation based on the lack of occurrence records near the monitoring station. A nuclear genome assembly for *P. patens* was included in the Kraken 2 reference database. Contig length and the number of mapped reads were positively correlated ($r = 0.92$; Fig. 57).

As found in *Pleurozium*, a substantial fraction of consensus BSPs were outside of the Funariales, likely due to the limited number of sequenced bryophytes (Table 78). This makes the BLAST results less informative, but we found no evidence to suggest the *Physcomitrella*-classified reads did not originate from a bryophyte. At the genus rank, 48% of ARs were with *Funaria*, a genus that does occur near the monitoring station, with an average 97% sequence identity. This, combined with the correlation between contig length and mapped reads, suggest the *Physcomitrella*-classified reads likely originated within the Funariaceae.

Table 78. Distribution of BLAST best-scoring sequence pairs (BSPs) for the 100 consensus sequences with the highest depth of coverage by taxonomic rank. Aligned reads (ARs) are the number of reads that contributed to a given consensus sequence query. %ARs are scaled by the sum of reads comprising queries with at least one BSP. Results are shown for taxonomic ranks comprising >5% of ARs and the hierarchy is collapsed to the lowest rank with identical results. %ID is

mean percent sequence identity, length is the mean alignment length, e-value is the mean expect value, and bitscore is the mean bitscore.

kingdom	phylum	class	order	family	%reads	%ID	length	e-value	bitscore
Viridiplantae	Streptophyta				100	91	1,188	8E-39	1,717
Viridiplantae	Streptophyta	Bryopsida			93	91	1,215	4E-126	1,755
Viridiplantae	Streptophyta	Bryopsida	Funariales	Funariaceae	64	91	1,126	6E-126	1,665
Viridiplantae	Streptophyta	Bryopsida	Pottiales	Pottiaceae	13	91	1,592	0.0	2,286
Viridiplantae	Streptophyta	Bryopsida	Hypnales		7	87	1,135	0.0	1,418
Viridiplantae	Streptophyta	Bryopsida	Bryales		7	98	763	2E-171	1,251
Viridiplantae	Streptophyta	Polytrichopsida	Polytrichales	Polytrichaceae	7	92	947	0E+00	1,376

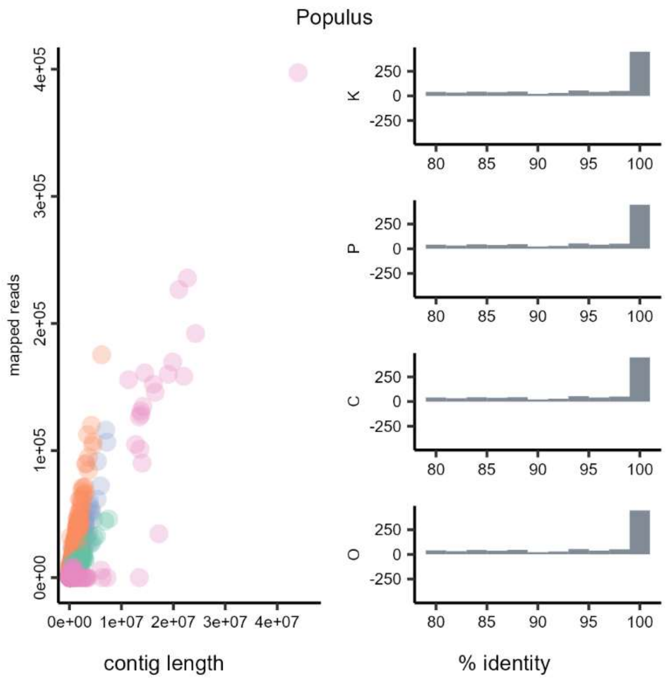

Figure 57 . Left: number of mapped reads *vs*. contig length for contigs > 50 kb. Right: mirrored histograms of the sequence identity of aligned reads (ARs) with BLAST best-scoring sequence pairs (BSPs) within (top) or outside (bottom) the kingdom (K), phylum (P), class (C), and order (O) ranks of *Pleurozium*. BSPs are shown for the 100 consensus sequences with the highest depth of coverage. ARs are the number of reads covering a given consensus sequence query and are given as *n*-fold differences.

Populus; Streptophyta; Magnoliopsida; Malpighiales; Salicaceae (3689)

Label: positive
Predicted probability: 0.98
Mapped reads: 44,977,262
Mapping rate: 84%
Base pairs queried: 870,257
Unique contigs queried: 56
Unique contigs aligned: 55

Nuclear genome assemblies for *Populus alba* (368.4 Mb, N50: 998 kb), *P. euphratica* (523.1 Mb, N50: 513 kb), *P. simonii* (391.5 Mb, N50: 1.7 Mb) and *P. trichocarpa* (392.2 Mb, N50: 13.2 Mb) were included in the Kraken 2 reference database.

Most (84%) *Populus*-classified reads could be mapped back to their sequences included in the reference database. Of these, the largest fraction (47%) mapped to the *P. alba* assembly, in orange (Fig. 58), which is closely related to related to *P. tremula* (Wang, et al. 2020), the species present in Fennoscandia. 25% of reads mapped to the *P. simonii* assembly, in blue, 19% to *P. trichocarpa*, in pink, and 9% to *P. euphratica* in green. More reads mapped to longer contigs, as expected for a true positive genus, both within each assembly and across all contigs (r = 0.88)

Consensus query BSPs were entirely within the Salicaceae family. 86% of ARs were with *Populus* and the remainder with *Salix* (Table 79).

Table 79. Distribution of BLAST best-scoring sequence pairs (BSPs) for the 100 consensus sequences with the highest depth of coverage by taxonomic rank. Aligned reads (ARs) are the number of reads that contributed to a given consensus sequence query. %ARs are scaled by the sum of reads comprising queries with at least one BSP. Results are shown for taxonomic ranks comprising >5% of ARs and the hierarchy is collapsed to the lowest rank with identical results. %ID is mean percent sequence identity, length is the mean alignment length, e-value is the mean expect value, and bitscore is the mean bitscore.

kingdom	phylum	class	order	family	%ARs	%ID	length	e-value	bitscore
Viridiplantae	Streptophyta	Magnoliopsida	Malpighiales	Salicaceae	100	89	1,928	3^{-72}	2,629

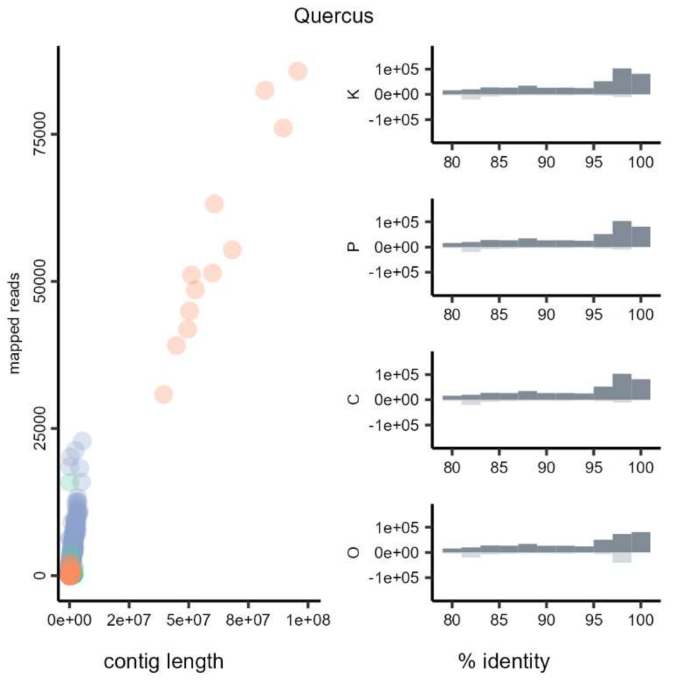

Figure 58. Left: number of mapped reads *vs*. contig length for contigs > 50 kb. Right: mirrored histograms of the sequence identity of aligned reads (ARs) with BLAST best-scoring sequence pairs (BSPs) within (top) or outside (bottom) the kingdom (K), phylum (P), class (C), and order (O) ranks of *Quercus*. BSPs are shown for the 1,000 consensus sequences with the highest depth of coverage. ARs are the number of reads covering a given consensus sequence query and are given as *n*-fold differences.

Quercus; Streptophyta; Magnoliopsida; Fagales; Fagaceae (3511)

Label: unlabeled
Predicted probability: 0.96
Mapped reads: 9,650,488
Mapping rate: 91%
Base pairs queried: 11,179,665
Unique contigs queried: 466
Unique contigs aligned: 397

We selected *Quercus* for validation because the northern distributional limit for the two most widely distribution European species, *Q. robur* and *Q. petrea,* is in southern Sweden. Neither is hardy in the interior of northern Sweden but can be used as ornamentals closer to the Gulf of Bothnia. The North American *Q. rubra* is more cold tolerant and could be planted closer to the aerosol monitoring station but to our knowledge is not widely used in landscaping in this part of Sweden.

Two nuclear genome assemblies for *Q. robur* (599.2 Mb, N50: 15 kb; 728.0 Mb, N50: 1.2 Mb) and single assemblies for *Q. lobata* (771.9 Mb, N50: 60.1 Mb) and *Q. suber (*861.9 Mb, N50: 420 kb) were included in the Kraken 2 reference database.

The mapping rate for the *Quercus*-classified reads was high (91%). Of these, 67% mapped to the *Q. robur* assemblies, indicated together in blue in Fig. 52, 14% to the highly-contiguous *Q. lobata* assembly, in orange, and 19% to *Q. suber*, in green. The number of mapped reads per contig was correlated with contig length (r = 0.92), a pattern consistent with a true positive genus (Fig. 59).

In our initial BLAST search of the top 100 consensus sequences by depth, 24% of reads aligned within the fungal class Dothideomycetes, the majority of which (14%) aligned with

Cladosporium, a genus of ubiquitous and abundant molds. 14% of reads aligned within Arthropoda, including lepidopterans (8%), mites (3%) and aphids (3%). Like the pattern we observed in *Lactarius*, these originated from short reference sequences (median = 6,097 bp) with high depth of coverage (median = 8,164×; range = 2,274 to 375,290×). Therefore, we expanded the analysis to the top 1,000 sequences by depth of coverage (1,348,002 bp) to investigate the extent of the contamination. We found eleven larger scaffolds (> 1 Mb) with BSPs within and outside of Fagales and, for these, we queried consensus sequences in non-overlapping 3,000 bp segments from their entirety (summing to 9.8 Mb) to verify their identity. Combined, these BLAST queries represent 4,047,756 aligned reads, or 42% of the total number of mapped reads.

The majority of BSPs were within Magnoliopsida and 78% of reads aligned within the Fagaceae family and 74% with *Quercus* specifically (Table 80). However, we found that 56 of the 466 unique queried contigs were likely reference contaminants from bacteria, arthropods, or fungi. The alignments with Poaceae appear to be from shared *Cladosporium* contamination in the *Q. suber* and *Digitaria exilis* nuclear genome assemblies. We found no evidence for contamination in the *Q. lobata* assembly but the three other *Quercus* assemblies had dubious contigs. Most of the contaminated contigs, however, were from the *Q. suber* assembly (n = 39). A single *Q. suber* contig (NW_019828185.1) likely resulted in the misclassification of 375,292 reads, that is, 50% of the total contaminated aligned reads.

Table 80. Distribution of BLAST best-scoring sequence pairs (BSPs) by taxonomic rank for the 1,000 consensus sequences with the highest depth of coverage and consensus sequences from the entirety of eleven large (> 1 Mb) scaffolds. Aligned reads (ARs) are the number of reads that contributed to a given consensus sequence query. %ARs are scaled by the sum of reads comprising queries with at least one BSP. Results are shown for taxonomic ranks comprising >5% of ARs and the hierarchy is collapsed to the lowest rank with identical results. %ID is mean percent sequence identity, length is the mean alignment length, e-value is the mean expect value, and bitscore is the mean bitscore.

kingdom	phylum	class	order	family	%ARs	%ID	length	e-value	bitscore
Viridiplantae	Streptophyta	Magnoliopsida			87	94	809	8^{-33}	1,265
Viridiplantae	Streptophyta	Magnoliopsida	Fagales		81	94	816	8^{-33}	1,275
Viridiplantae	Streptophyta	Magnoliopsida	Fagales	Fagaceae	78	94	826	9^{-33}	1,294
Viridiplantae	Streptophyta	Magnoliopsida	Poales	Poaceae	6	98	479	0.00	820
Fungi	Ascomycota				7	85	614	2^{-34}	718
Fungi	Ascomycota	Dothideomycetes			6	86	681	1^{-39}	807

Despite this evidence for misclassification due to contamination, most *Quercus*-classified reads are plausibly from *Quercus*. *Quercus* was one of the genera used to compare the results from simply querying reads against the nt database *vs*. the consensus sequence approach we ultimately used for validation. Of the 100 randomly selected paired-end reads, 89% had BSPs exclusively within Fagaceae (Table 81). We expect the true misclassification rate to be between the 10-20% estimated by the two approaches and thus consider *Quercus* a true positive.

Table 81. Distribution of BLAST best-scoring sequence pairs (BSPs) by taxonomic rank for 100 randomly-selected pair-end reads. Results are shown for taxonomic ranks comprising >5% of reads and the hierarchy is collapsed to the lowest rank with identical results. %ID is mean percent sequence identity, length is the mean alignment length, e-value is the mean expect value, and bitscore is the mean bitscore.

kingdom	phylum	class	order	family	%BSPs	%ID	length	e-value	bitscore

Viridiplantae	Streptophyta				92	97	128	2^{-28}	217
Viridiplantae	Streptophyta	Magnoliopsida			90	97	129	2^{-28}	218
Viridiplantae	Streptophyta	Magnoliopsida	Fagales	Fagaceae	89	98	129	2^{-28}	218

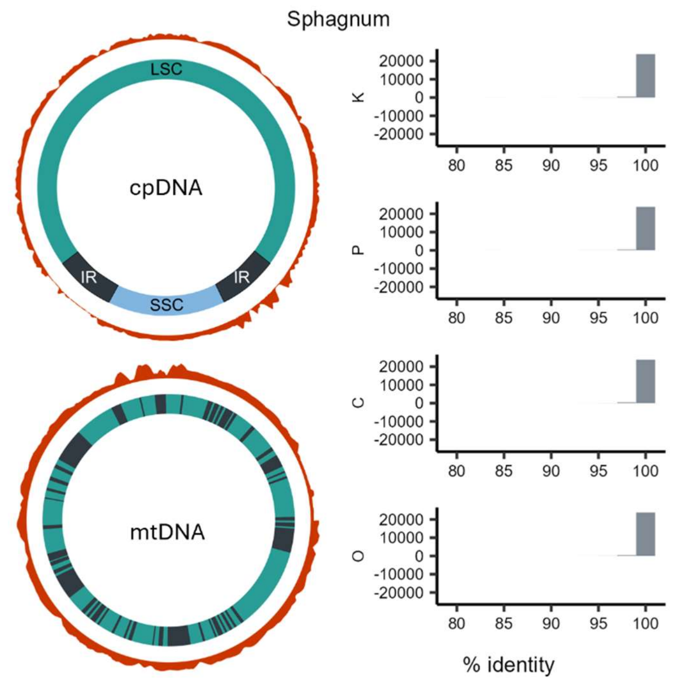

Figure 59. Left: outer, red rings show the mean depth (500 bp moving window) of reads mapped to the *Sphagnum* chloroplast (cpDNA) and mitochondrial (mtDNA) genomes. The inner ring for the cpDNA show the position of the large single copy (LSC), small single copy (SSC) and inverted repeat (IRs) regions. For the mtDNA, the inner ring indicates genic (green) and intergenic (dark grey) regions. Right: mirrored histograms of the sequence identity of aligned reads (ARs) with BLAST best-scoring sequence pairs (BSPs) within (top) or outside (bottom) the kingdom (K), phylum (P), class (C), and order (O) ranks of *Sphagnum*. BSPs are shown for the 100 consensus sequences with the highest depth of coverage. ARs are the number of reads covering a given consensus sequence query and are given as *n*-fold differences.

***Sphagnum*; Streptophyta; Sphagnopsida; Sphagnales; Sphagnaceae (13804)**

Label: positive
Predicted probability: 0.99
Mapped reads: 21,994,848
Mapping rate: 97%
Base pairs queried: 383,793
Unique contigs queried: 97
Unique contigs aligned: 97

No nuclear genome assembly for *Sphagnum* was published when the Kraken 2 database was constructed, but organelle genomes for *S. palustre* were included.

Depth of coverage across chloroplast and mitochondrial genomes are shown in Fig. 60 and the large single copy (LSC), small single copy (SSC) and inverted repeat regions (IR) are annotated. Like *Dryopteris* and *Equisetum,* reads mapped over the LSC and SSC regions with similar depth and were concentrated in one copy of the IR. Reads also mapped over the entirety of the mtDNA genome, although depths were higher in three genes (nad1, nad9, and cob) and lowest in atp9. Overall, both organelles fit the expectations for a true positive genus.

All consensus query BSPs were specifically with *Sphagnum* and had an average alignment length of 1,089 bp with 99% sequence identity (Table 82).

Table 82. Summary of BLAST best-scoring sequence pairs (BSPs) by taxonomic rank for the 100 consensus sequences with the highest read depth. Aligned reads (ARs) are the number of reads that contributed to a given consensus sequence query. %ARs are scaled by the sum of reads comprising queries with at least one BSP. Results are shown for taxonomic ranks comprising >5% of ARs and the hierarchy is collapsed to the lowest rank with identical results. %ID is mean percent sequence identity, length is the mean alignment length, e-value is the mean expect value, and bitscore is the mean bitscore.

kingdom	phylum	class	order	family	%reads	%ID	length	e-value	bitscore

Viridiplantae	Streptophyta	Sphagnopsida	Sphagnales	Sphagnaceae	100	99	1,089	2E-76	1,919

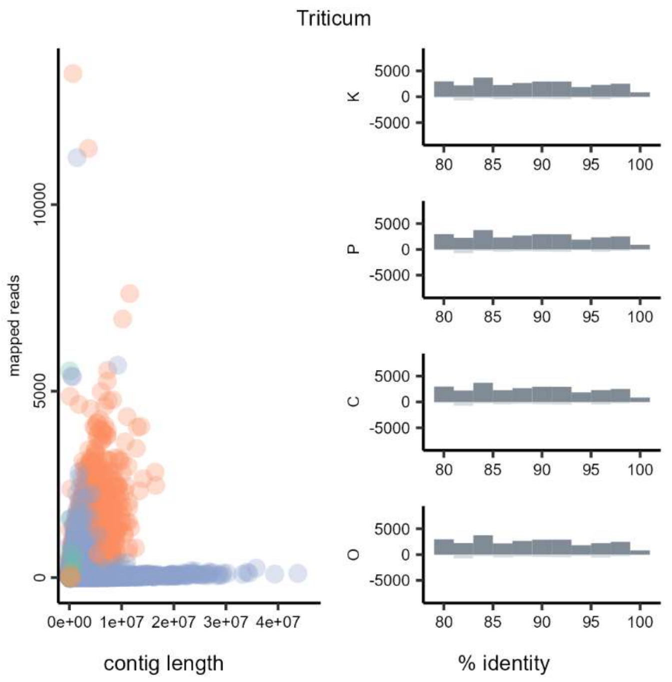

Figure 60. Left: number of mapped reads *vs*. contig length for contigs > 50 kb. Right: mirrored histograms of the sequence identity of aligned reads (ARs) with BLAST best-scoring sequence pairs (BSPs) within (top) or outside (bottom) the kingdom (K), phylum (P), class (C), and order (O) ranks of *Triticum*. BSPs are shown for the 1,000 consensus sequences with the highest depth of coverage. ARs are the number of reads covering a given consensus sequence query and are given as *n*-fold differences.

Triticum; Streptophyta; Magnoliopsida; Poales; Poaceae (4564)

Label: unlabeled
Predicted probability: 0.94
Mapped reads: 30,304,912
Mapping rate: 91%
Base pairs queried: 1,074,372
Unique contigs queried: 786
Unique contigs aligned: 551

We selected *Triticum* for validation because of its abundance and lack of occurrence reports within 40 km of the aerosol monitoring station. Less than 0.5% of Norrbotten county is arable land and most of this is in leys. However, *T. aestivum* is naturalized along the Gulf of Bothnia and has been reported 95 km southeast of the aerosol monitoring station.

Nine *Triticum* assemblies were included in the Kraken 2 database: five partial and one complete assembly (14.5 Gb, N50: 2.3 Mb) for *T. aestivium*, two for *T. dicoccoides* (8.0 Gb, N50: 8.4 Mb and 10.1 Gb, N50: 1.4 Mb) and one for *T. uratru* (2.8 Gb, N50: 61 kb).

Most (91%) of the *Triticum*-classified reads mapped to their reference database sequences. Of these, 79% mapped to a *T. aestivium* assembly, shown in orange in Fig. 61, 12% to the fragmented *T. uratru* assembly, in green, and 9% to the *T. dicoccoides* assemblies, in blue. However, most contigs with mapped reads from *T. dicoccoides* were from the more fragmented assembly, with disproportionately few reads mapping to the more contiguous assembly. Nevertheless, the number of reads mapped to *T. aestivum* contigs were positively correlated (r = 0.77) with contig length, as expected for a true positive genus (Fig. 61).

In our initial BLAST search of the top 100 consensus sequences by depth, 83% of reads aligned within the Poaceae family and 4-5% from within fungi, Diptera (Arthropoda: Insecta), and γ-proteobacteria. We then expanded the analysis to the top 1,000 sequences by depth of coverage. The proportion of reads aligned within Poaceae increased slightly to 85%, as did the proportion aligned with fungi, to 6% (Table 83). Fungal BSPs spanned multiple orders within Basidiomycota and Ascomycota, with the largest fraction of reads (1.5%) aligning within Polyporales (Basidiomycota: Agaricomycetes).

Unlike some genera, such as *Quercus* and *Lactarius*, the depth of coverage of putative contaminant contigs substantially overlapped with those of the Poaceae-like contigs

(median = 952×, range = 338 - 31,390× for contaminants *vs*. median = 596×, range = 336 - 117,828×). They were, however, substantially shorter, with a median length of 1,343 bp compared to 168,128 bp for the Poaceae-like contigs. In total, we found 140 contigs putatively of fungal, prokaryotic, or metazoan origin across six different assemblies. Most contaminants (n = 95) originated from the *T. uratru* assembly, which is likely responsible for 60% of the misclassified reads found here.

Table 83. Distribution of BLAST best-scoring sequence pairs (BSPs) for the 1,000 consensus sequences with the highest depth of coverage by taxonomic rank. Aligned reads (ARs) are the number of reads that contributed to a given consensus sequence query. %ARs are scaled by the sum of reads comprising queries with at least one BSP. Results are shown for taxonomic ranks comprising >5% of ARs and the hierarchy is collapsed to the lowest rank with identical results. %ID is mean percent sequence identity, length is the mean alignment length, e-value is the mean expect value, and bitscore is the mean bitscore.

kingdom	phylum	class	order	family	%ARs	%ID	length	e-value	bitscore
Viridiplantae	Streptophyta	Magnoliopsida			87	89	536	8^{-30}	726
Viridiplantae	Streptophyta	Magnoliopsida	Poales		86	89	531	8^{-30}	715
Viridiplantae	Streptophyta	Magnoliopsida	Poales	Poaceae	85	88	533	8^{-30}	717
Fungi					6	89	484	7^{-37}	635

Triticum was one of the genera used to compare the results from simply querying reads against the nt database *vs*. the consensus sequence approach we applied to all genera for validation. Of the 100 randomly selected paired-end reads, 96% aligned with Poaceae (Table 84). This suggests the 15% misclassification rate inferred from the consensus sequence approach could be overly conservative. Whether the true misclassification rate is closer to 5% or 15%, we consider this level of accuracy to be high enough to consider *Triticum* as a true positive genus for the analyses conducted in this paper.

Table 84. Distribution of BLAST best-scoring sequence pairs (BSPs) by taxonomic rank for 100 randomly-selected pair-end reads. Results are shown for taxonomic ranks comprising >5% of reads and the hierarchy is collapsed to the lowest rank with identical results. %ID is mean percent sequence identity, length is the mean alignment length, e-value is the mean expect value, and bitscore is the mean bitscore.

kingdom	phylum	class	order	family	%BSPs	%ID	length	e-value	bitscore
Viridiplantae					98	92	131	4^{-28}	191
Viridiplantae	Streptophyta	Magnoliopsida	Poales	Poaceae	96	92	131	4^{-28}	190

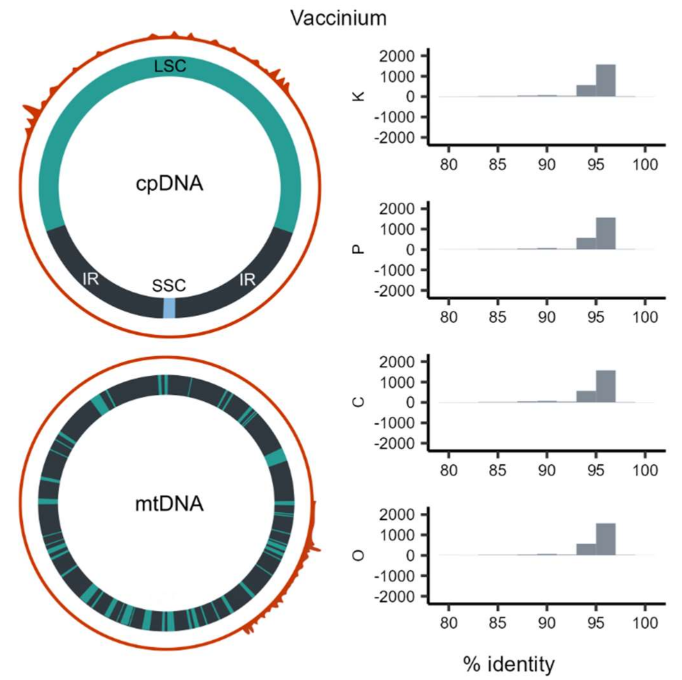

Figure 61. Left: outer, red rings show the mean depth (500 bp moving window) of reads mapped to the *Vaccinium* chloroplast (cpDNA) and mitochondrial (mtDNA) genomes. The inner ring for the cpDNA show the position of the large single copy (LSC), small single copy (SSC) and inverted repeat (IRs) regions. For the mtDNA, the inner ring indicates genic (green) and intergenic (dark grey) regions. Right: mirrored histograms of the sequence identity of aligned reads (ARs) with BLAST best-scoring sequence pairs (BSPs) within (top) or outside (bottom) the kingdom (K), phylum (P), class (C), and order (O) ranks of *Vaccinium*. BSPs are shown for the 100 consensus sequences with the highest depth of coverage. ARs are the number of reads covering a given consensus sequence query and are given as *n*-fold differences.

***Vaccinium*; Streptophyta; Magnoliopsida; Ericales; Ericaceae (13749)**

Label: positive
Predicted probability: 0.99
Mapped reads: 3,774,846
Mapping rate: 77%
Base pairs queried: 274,295
Unique contigs queried: 49
Unique contigs aligned: 48

No nuclear genome assembly for *Vaccinium* was published when the Kraken 2 database was constructed, but chloroplast genomes for *V. macrocarpon* and *V. oldhamii* and a mitochondrial genome for *V. macrocarpon* were included. Neither species occurs in Europe. Five *Vaccinium* species occur in northern Sweden, all with a comparable number of occurrence records: *V. microcarpum*, *V. myrtillus*, *V. oxycoccos*, *V. uliginosum,* and *V. vitis-idaea*.

Chloroplast genome size and structure is unusually variable within *Vaccinium*, with published assemblies ranging from *ca*. 173 to 196 kb with 126-147 genes. *V. myrtillus*, in particular, has a chloroplast genome *ca*. 16 kb larger than the assemblies in the Kraken 2 reference database and shows low sequence identity with other *Vaccinium* species in the inverted repeat regions (Fahrenkrog et al. 2022). This may explain the observed pattern of reads largely mapping to a few positions in the large single copy (LSC; Fig. 62), rather than the expected pattern of similar read depth across the single copy regions and higher depth in one copy of the inverted repeat (*cf*. *Dryopteris*).

Plant mitochondrial genomes are often structurally complex and may be multichromosomal, highly recombinant, and can show high gene copy number variation through the formation of subgenomic molecules (Sullivan et al. 2019). While mitochondrial genome evolution has not been investigated in *Vaccinium* specifically, uneven coverage of a plant mitochondrial genome is not surprising.

While their complex genome structure made the read mapping analysis uninformative, mutation rates, particularly within genes, are still expected to be low in plant organelle genomes. All BSPs for the *Vaccinium* consensus sequences were within the Magnoliopsida and 99% of ARs were within the Ericaceae family (Table 85). At the genus level, 91% of reads aligned with *Vaccinium*

(%ID = 91, length = 1,065 bp) and the remaining with *Rhododendron* and *Calluna* (%ID = 90, length = 619 bp).

Table 85. Distribution of BLAST best-scoring sequence pairs (BSPs) for the 100 consensus sequences with the highest depth of coverage by taxonomic rank. Aligned reads (ARs) are the number of reads that contributed to a given consensus sequence query. %ARs are scaled by the sum of reads comprising queries with at least one BSP. Results are shown for taxonomic ranks comprising >5% of ARs and the hierarchy is collapsed to the lowest rank with identical results. %ID is mean percent sequence identity, length is the mean alignment length, e-value is the mean expect value, and bitscore is the mean bitscore.

kingdom	phylum	class	order	family	%ARs	%ID	length	e-value	bitscore
Viridiplantae	Streptophyta	Magnoliopsida			100	90	780	8^{-57}	1,121
Viridiplantae	Streptophyta	Magnoliopsida	Ericales	Ericaceae	99	91	810	9^{-57}	1,168

False positives

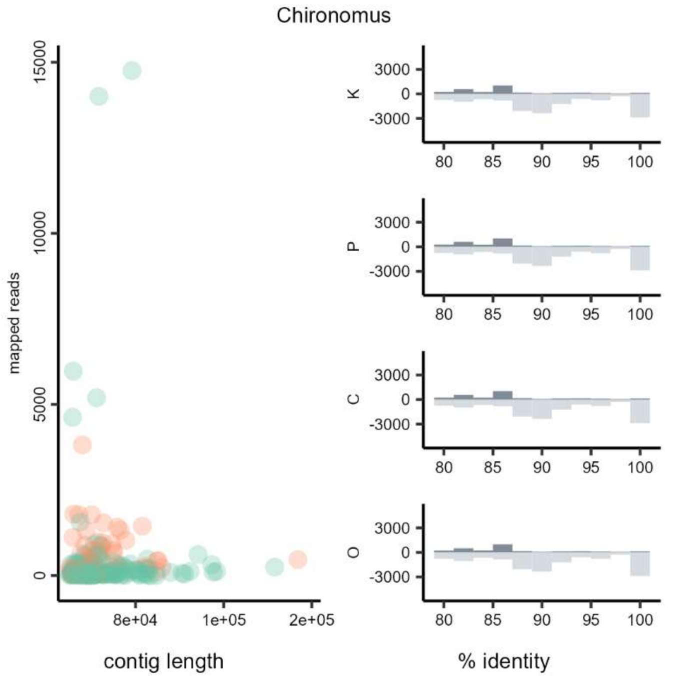

Figure 62. Left: number of mapped reads *vs*. contig length for contigs > 50 kb. Right: mirrored histograms of the sequence identity of aligned reads (ARs) with BLAST best-scoring sequence pairs (BSPs) within (top) or outside (bottom) the kingdom (K), phylum (P), class (C), and order (O) ranks of *Chironomus*. BSPs are shown for the 100 consensus sequences with the highest depth of coverage. ARs are the number of reads covering a given consensus sequence query and are given as *n*-fold differences.

Chironomus; **Arthropoda; Insecta; Diptera; Chironomidae (7150)**

Label: positive
Predicted probability: 0.98
Mapped reads: 17,234,178
Mapping rate: 1%
Base pairs queried: 2,385,309
Unique contigs queried: 645
Unique contigs aligned: 625

Two nuclear genomes assemblies for *Chironomus riparius* (154.5 Mb, N50: 7 kb and 180.7 Mb, N50: 6 kb) and one for *C. tentans* (213.5 Mb, N50: 7 kb) were included in the Kraken 2 reference database. An estimated 680 Chironomidae species occur in Sweden (Ronquist, Forshage, Haggqvist, et al. 2020), including *C. riparius* and *tentans*, although occurrence records are usually made at the sub-family or family rank.

The mapping rate of the *Chironomus*-classified reads to their Kraken 2 reference database sequences was extremely low (1%). This result was unexpected, especially compared to the average 74% (σ = 25%) mapping rate for all 57 genera analyzed here. Of mapped reads, 87% mapped to the two *C. riparius* assemblies, indicated in green in Fig. 63, and 11% to the *C. tentans* assembly, in orange. The number of reads mapped per contig was uncorrelated with contig length (r = 0.00), unlike the majority of the true positive genera.

In our initial BLAST search of the top 100 consensus sequences by depth, 82% of reads aligned with a prokaryote. Most of these hits lacked any further taxonomic data, but 35% were with *Wolbachia*, a ubiquitous endosymbiont of arthropods. We expanded the search to the top 1,000 consensus queries by depth to assess the extent of *Wolbachia* contamination but the fraction of alignments with bacteria remained unchanged (Table 86). In total, we found 483 unique contigs of likely prokaryote origin between the two *C. riparius* assemblies but none in the *C. tentans* assembly.

Table 86. Distribution of BLAST best-scoring sequence pairs (BSPs) for the 1,000 consensus sequences with the highest depth of coverage by taxonomic rank. Aligned reads (ARs) are the number of reads that contributed to a given consensus sequence query. %ARs are scaled by the sum of reads comprising queries with at least one BSP. Results are shown for taxonomic ranks comprising >5% of ARs and the hierarchy is collapsed to the lowest rank with identical results. %ID is

mean percent sequence identity, length is the mean alignment length, e-value is the mean expect value, and bitscore is the mean bitscore.

kingdom	phylum	class	order	family	%ARs	%ID	length	e-value	bitscore
Prokaryota					82	90	1,296	9^{-32}	1,863
Prokaryota	Pseudomonadota	α-proteobacteria	Rickettsiales	Anaplasmataceae	28	90	1,847	2^{-35}	2,694
Metazoa	Arthropoda	Insecta			18	88	598	7^{-35}	774
Metazoa	Arthropoda	Insecta	Diptera		17	88	594	8^{-35}	777
Metazoa	Arthropoda	Insecta	Diptera	Chironomidae	16	89	591	5^{-38}	788

Some *Chironomus*-classified reads aligned to the reference database appear to originate from a chironomid (Table 86) and this fraction could be higher among the reads mapped to lower-coverage contigs. However, only 1% of the total *Chironomus*-classified reads to the reference sequences. In a sample of 100 randomly selected paired end reads, 90% of BSPs were within the moth family Geometridae (Table 87). Alignments occurred with sequences from numerous species, including short amplicons, mRNA sequences, and chromosome-level assemblies. Relaxing the stringency thresholds for the BSPs resulted in more alignments with other lepidopteran families. Therefore, the taxonomic distribution of the BSPs cannot be readily explained by a contaminant (*e.g. Wolbachia*) common to both the *Chironomus* and lepidopteran assemblies or by contamination of the lepidopteran assemblies by *Chironomus* (*cf. Picea* and *Timema*). While reference contamination was a clear source of classification error, most *Chironomus*-classified reads originated from moths, plants, fungi, or bacteria, making this a false positive genus.

Table 87. Distribution of BLAST best-scoring sequence pairs (BSPs) by taxonomic rank for 100 randomly-selected pair-end reads. Results are shown for taxonomic ranks comprising >5% of BSPs and the hierarchy is collapsed to the lowest rank with identical results. %ID is mean percent sequence identity, length is the mean alignment length, e-value is the mean expect value, and bitscore is the mean bitscore.

kingdom	phylum	class	order	family	%BSPs	%ID	length	e-value	bitscore
Metazoa	Arthropoda	Insecta	Lepidoptera	Geometridae	89	93	133	4^{-19}	195
Viridiplantae	Streptophyta				5	90	141	2^{-26}	189
Fungi					5	93	137	2^{-27}	204

Brugia; Nematoda; Chromadorea; Rhabditida; Onchocercidae (6278)

Label: unlabeled
Predicted probability: 0.98
Mapped reads: 17,365,122
Mapping rate: 97%
Base pairs queried: 41,190
Unique contigs queried: 85
Unique contigs aligned: 28

Brugia was flagged for validation because this genus of filarial nematodes is only documented in southeast Asia. Four *Brugia* genome assemblies were included in the Kraken 2 reference database: two for *B. pahangi* (69.1 Mb, N50: 143 kb and 74.6 Mb, N50: 59 kb) and one for *B. malayi* (76.2 Mb, N50: 47 kb) and *B. timori* (56.9 Mb, N50: 5 kb).

The mapping rate for the *Brugia*-classified reads was extremely high (97%), but all 508 of these reads mapped to the *B. timori* assembly, indicated in green in Fig. 64. Furthermore, 7 million reads (83%) mapped to just two contigs from this assembly, with lengths of 653 and 567 bp.

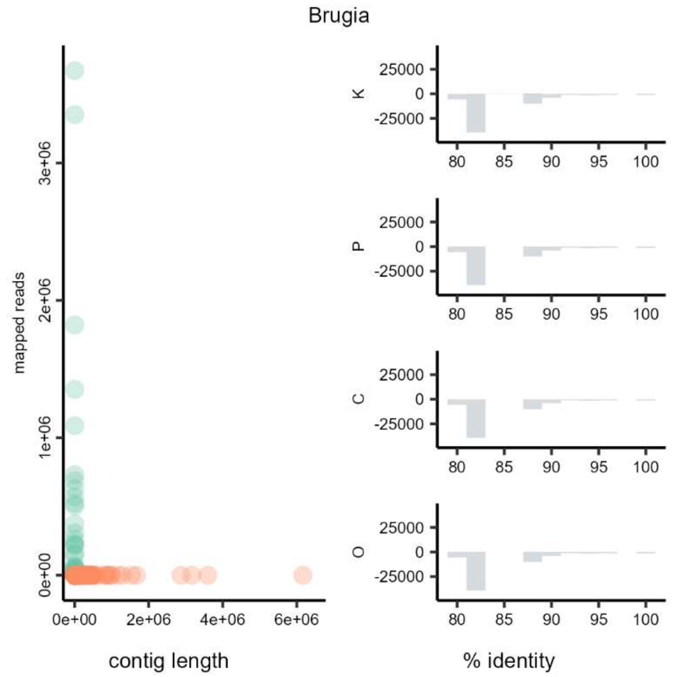

Figure 63. Left: number of mapped reads *vs*. contig length. Right: mirrored histograms of the sequence identity of aligned reads (ARs) with BLAST best-scoring sequence pairs (BSPs) within (top) or outside (bottom) the kingdom (K), phylum (P), class (C), and order (O) ranks of *Brugia*. BSPs are shown for the 100 consensus sequences with the highest depth of coverage. ARs are the number of reads covering a given consensus sequence query and are given as *n*-fold differences

All BSPs for the 100 consensus sequences with the highest depth of coverage were within the plant phylum Streptophyta (Table 88). BSPs within Pinaceae accounted for 96% of aligned reads and 94% were specifically with *Pinus.*

Table 88. Distribution of BLAST best-scoring sequence pairs (BSPs) for the 100 consensus sequences with the highest depth of coverage by taxonomic rank. Aligned reads (ARs) are the number of reads that contributed to a given consensus sequence query. %ARs are scaled by the sum of reads comprising queries with at least one BSP. Results are shown for taxonomic ranks comprising >5% of ARs and the hierarchy is collapsed to the lowest rank with identical results. %ID is mean percent sequence identity, length is the mean alignment length, e-value is the mean expect value, and bitscore is the mean bitscore.

kingdom	phylum	class	order	family	%ARs	%ID	length	e-value	bitscore
Viridiplantae	Streptophyta				100	90	469	2^{-33}	633
Viridiplantae	Streptophyta	Pinopsida	Pinales	Pinaceae	96	90	469	2^{-33}	622

Brugia was among the genera used to compare the results of directly querying reads against the nt database *vs.* the consensus sequence approach we applied to all genera. 96% of these BSPs were also within Pinaceae (Table 82), with 94% with *Pinus*. We found no evidence to suggest that any of the *Brugia*-classified reads were correctly classified at the even the kingdom rank, making this genus an unambiguous false positive.

Unsurprisingly, the abundance *Brugia*-classified reads was strongly correlated to *Pinus*. In our hierarchical clustering of pairwise log-ratio variance, *Brugia* was placed in the *Pinus*-dominated cluster. Consequently, the *Brugia*-classified effectively contributed to the time series of their actual source organism.

Table 89. Distribution of BLAST best-scoring sequence pairs (BSPs) by taxonomic rank for 100 randomly-selected pair-end reads. Results are shown for taxonomic ranks comprising >5% of reads and the hierarchy is collapsed to the lowest rank with identical results. %ID is mean percent sequence identity, length is the mean alignment length, e-value is the mean expect value, and bitscore is the mean bitscore.

kingdom	phylum	class	order	family	%BSPs	%ID	length	e-value	bitscore
Viridiplantae	Streptophyta				100	88	139	3^{-27}	175
Viridiplantae	Streptophyta	Pinopsida	Pinales	Pinaceae	96	88	140	4^{-27}	176

***Gavia*;Chordata;Aves;Gaviiformes;Gaviidae (37038)**

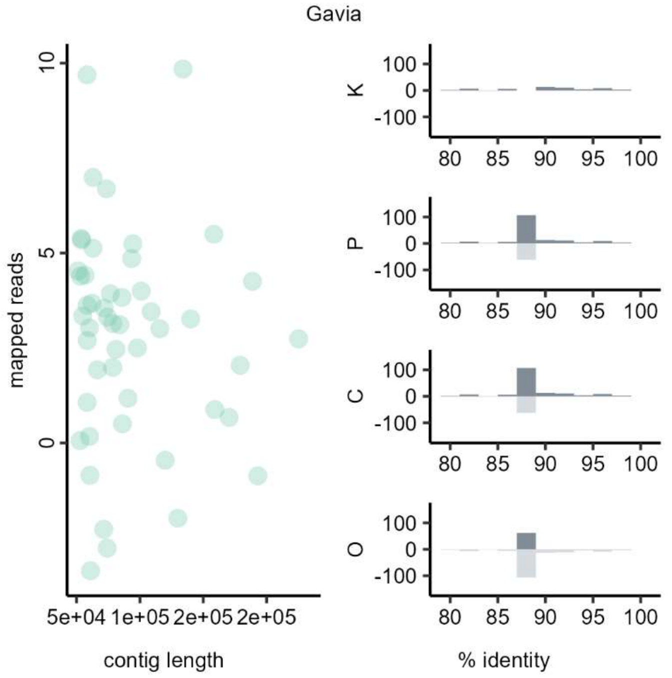

Figure 64. Left: number of mapped reads *vs*. contig length for contigs > 50 kb. Right: mirrored histograms of the sequence identity of aligned reads (ARs) with BLAST best-scoring sequence pairs (BSPs) within (top) or outside (bottom) the kingdom (K), phylum (P), class (C), and order (O) ranks of *Gavia*. BSPs are shown for the 100 consensus sequences with the highest depth of coverage. ARs are the number of reads covering a given consensus sequence query and are given as *n*-fold differences.

Label: positive
Predicted probability: 0.78
Mapped reads: 476
Mapping rate: 1%
Base pairs queried: 8,697
Unique contigs queried: 99
Unique contigs aligned: 22

A nuclear genome assembly for *Gavia stellata* (1.1 Gb, N50: 45 kb) was included in the Kraken 2 reference database. This species and *G. arctica* are reported in northern interior Sweden with approximately equal frequency.

Very few (1%) of the *Gavia*-classified reads could be mapped back to their reference database sequences. The number of mapped reads per contig was not correlated with contig length (r = 0.15). Only *Chironomus*, another false positive detection, had a similarly low mapping rate.

Consensus queries BSPs were predominately within Aves, with 28% of ARs within the Gaviidae family (Table 90). The entire Gaviiformes order comprises five extant species, of which only *G. stellata* has a nuclear genome assembly. The distribution of BSPs within Aves likely reflects this limited sequence availability. However, 28% of ARs were also within Insecta, which cannot be explained by the taxonomic composition of the nt database.

Table 90. Distribution of BLAST best-scoring sequence pairs (BSPs) for the 100 consensus sequences with the highest depth of coverage by taxonomic rank. Aligned reads (ARs) are the number of reads that contributed to a given consensus sequence query. %ARs are scaled by the sum of reads comprising queries with at least one BSP. Results are shown for taxonomic ranks comprising >5% of ARs and the hierarchy is collapsed to the lowest rank with identical results. %ID is mean percent sequence identity, length is the mean alignment length, e-value is the mean expect value, and bitscore is the mean bitscore.

kingdom	phylum	class	order	family	%ARs	%ID	length	e-value	bitscore
Metazoa					100	89	175	2E-32	231
Metazoa	Chordata	Aves			72	89	176	2E-32	232
Metazoa	Chordata	Aves	Gaviiformes	Gaviidae	28	88	158	2E-46	200
Metazoa	Chordata	Aves	Psittaciformes		15	88	156	1E-45	199
Metazoa	Chordata	Aves	Psittaciformes	Psittaculidae	14	87	158	2E-45	196
Metazoa	Chordata	Aves	Passeriformes		9	92	163	4E-51	232
Metazoa	Chordata	Aves	Accipitriformes	Accipitridae	7	91	179	5E-34	248
Metazoa	Arthropoda	Insecta			28	88	159	3E-47	204
Metazoa	Arthropoda	Insecta	Diptera	Hippoboscidae	14	88	159	5E-47	202
Metazoa	Arthropoda	Insecta	Thysanoptera	Thripidae	14	89	158	4E-48	205

We also queried all 1,729 *Gavia*-classified reads longer than 100 bp against the BLAST nt database. Only 52 had BSPs meeting our filtering criteria (> 80% identity, > 100 bp, and e-value < 1.0 x 10^{-15}, which increased to 384 if we allowed alignments as short as 50 bp. In either case, the taxonomic distribution of BSPs were similar, with *ca*. 65% of reads aligned within Aves and *ca.* 8% within Gaviidae. As with the consensus query BSPs, about 28% of read BSPs unambiguously indicated contamination (Table 91).

The majority (78%) of read and consensus queries lacked any BLAST hits at all. Given this and the lack of success read mappings, the misclassification rate could be much higher than the 30% indicated by the successful BLAST alignments. Overall, we consider *Gavia* to be a false positive, or at least too unreliable for individual analyses, if even some reads are correctly classified.

Table 91. Distribution of BLAST best-scoring sequence pairs (BSPs) by taxonomic rank for 1,729 reads longer than 100 bp. Results are shown for taxonomic ranks comprising >5% of reads and the hierarchy is collapsed to the lowest rank with identical results. %ID is mean percent sequence identity, length is the mean alignment length, e-value is the mean expect value, and bitscore is the mean bitscore.

kingdom	phylum	class	order	family	%BSPs	%ID	length	e-value	bitscore
Metazoa					97	92	95	8E-13	136
Metazoa	Chordata				74	92	97	9E-13	140
Metazoa	Chordata	Aves			66	92	99	9E-13	143
Metazoa	Chordata	Aves	Passeriformes		20	94	92	5E-13	140
Metazoa	Chordata	Aves	Accipitriformes	Accipitridae	15	93	97	2E-12	142
Metazoa	Chordata	Aves	Gaviiformes	Gaviidae	8	89	116	1E-15	148
Metazoa	Arthropoda				20	91	81	3E-13	111
Metazoa	Arthropoda	Insecta			16	91	84	2E-14	115
Metazoa	Arthropoda	Insecta	Diptera		7	90	90	5E-17	120
Metazoa	Chordata	Actinopteri			8	92	67	6E-13	95

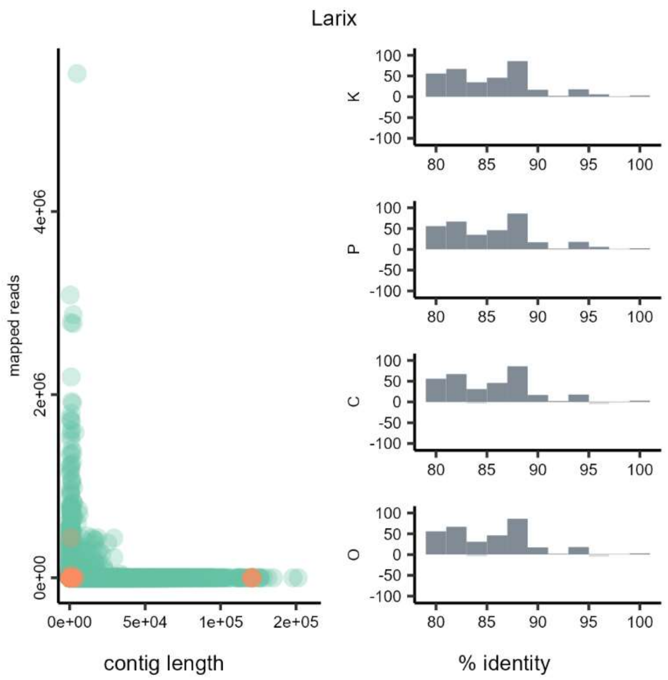

Figure 65. Left: number of mapped reads *vs*. contig length for contigs > 50 kb. Right: mirrored histograms of the sequence identity of aligned reads (ARs) with BLAST best-scoring sequence pairs (BSPs) within (top) or outside (bottom) the kingdom (K), phylum (P), class (C), and order (O) ranks of *Larix*. BSPs are shown for the 100 consensus sequences with the highest depth of coverage. ARs are the number of reads covering a given consensus sequence query and are given as *n*-fold differences.

Larix; Streptophyta; Pinopsida; Pinales; Pinaceae (3325)

Label: negative
Predicted probability: 0.85
Mapped reads: 801,974,162
Mapping rate: 84%
Base pairs queried: 136,811
Unique contigs queried: 100
Unique contigs aligned: 46

We flagged *Larix* for validation because this genus was the second most abundant on average, after *Pinus*. This was surprising because *Larix* was not used in commercial forestry in Sweden prior to 2000 and comprised < 0.1% of northern Swedish stands by 2012 (Skogstyrelsen 2020). We extracted reads from the week with the highest abundance in each year for *Larix*: 1974:25, 1976:27, 1978:26, 1980:26, 1982:28, 1984:23, 1986:26, 1988:21, 1990:27, 1992:25, 1994:27, 1996:28, 2000:28, 2002:23, and 2004:28.

A partial nuclear assembly for *L. sibirica* (7.8 Gb of *ca.* 12 Gb, N50: 1 kb) was included in the Kraken 2 reference database. All mapped reads are shown in Fig. 66, with the *L. sibirica* assembly in green. Short Sanger sequences and seven plastid genomes (*ca.* 120 kb) were also included in the reference database, indicated in orange. No more than 62 reads mapped to any plastid genome. Overall, 70.5% of reads mapped to contigs < 1,000 bp.

All BSPs for the 100 consensus queries were within Viridiplantae, with of 97% of ARs within the Pinaceae family and 96% with *Pinus* specifically (Table 92). Less than 1% of ARs (0.72%) aligned with *Larix* and these had lower mean sequence identity (81%) and alignment lengths (384 bp) than the BSPs with *Pinus* (%ID = 85%, length = 684 bp), *Cathaya* (Pinopsida: Pinaceae; %ARs = 0.79, %ID = 99, length = 1,787 bp) or *Carex* (Magnoliopsida: Poales; %ARs = 0.95, %ID = 97, length = 1,409 bp).

Table 92. Distribution of BLAST best-scoring sequence pairs (BSPs) for the 100 consensus sequences with the highest depth of coverage by taxonomic rank. Aligned reads (ARs) are the number of reads that contributed to a given consensus sequence query. %ARs are scaled by the sum of reads comprising queries with at least one BSP. Results are shown for taxonomic ranks comprising >5% of ARs and the hierarchy is collapsed to the lowest rank with identical results. %ID is mean percent sequence identity, length is the mean alignment length, e-value is the mean expect value, and bitscore is the mean bitscore.

kingdom	phylum	class	order	family	%ARs	%ID	length	e-value	bitscore
Viridiplantae	Streptophyta				100	85	724	2^{-30}	899
Viridiplantae	Streptophyta	Pinopsida	Pinales	Pinaceae	97	85	703	2^{-30}	852

Larix was among the genera used to compare the results of directly querying reads against the nt database *vs.* the consensus sequence approach we applied to all genera. 92% of these BSPs were within Pinaceae (Table 93) and 88% were with *Pinus* specifically. None of the reads aligned with *Larix.*

In summary, we found that ≥ 90% of the *Larix*-classified reads originated from *Pinus*. As with *Brugia,* the hierarchical clustering of pairwise log-ratio variances grouped *Larix* in the *Pinus*-dominated cluster. Despite their misclassification, these reads therefore contributed to the time series of their actual source.

Table 93. Distribution of BLAST best-scoring sequence pairs (BSPs) by taxonomic rank for 100 randomly-selected pair-end reads. Results are shown for taxonomic ranks comprising >5% of reads and the hierarchy is collapsed to the lowest rank with identical results. %ID is mean percent sequence identity, length is the mean alignment length, e-value is the mean expect value, and bitscore is the mean bitscore.

kingdom	phylum	class	order	family	%BSPs	%ID	length	e-value	bitscore
Viridiplantae	Streptophyta				100	90	143	2^{-28}	188
Viridiplantae	Streptophyta	Pinopsida	Pinales	Pinaceae	92	89	145	2^{-28}	186
Viridiplantae	Streptophyta	Magnoliopsida	Poales	Cyperaceae	8	97	125	1^{-49}	212

Termitomyces; Basidiomycota; Agaricomycetes; Agaricales; Lyophyllaceae (71927)

Label: negative
Predicted probability: 0.75
Mapped reads: 2,137,222
Mapping rate: 90%
Base pairs queried: 63,931
Unique contigs queried: 67
Unique contigs aligned: 50

Termitomyces was included as a negative genus in the GBM training dataset because this genus occurs only in the tropics and genomes were available for the Lyophyllaceae genera found in Europe. However, this genus still received a high enough predicted probability from the GBM to be classified as a positive occurrence.

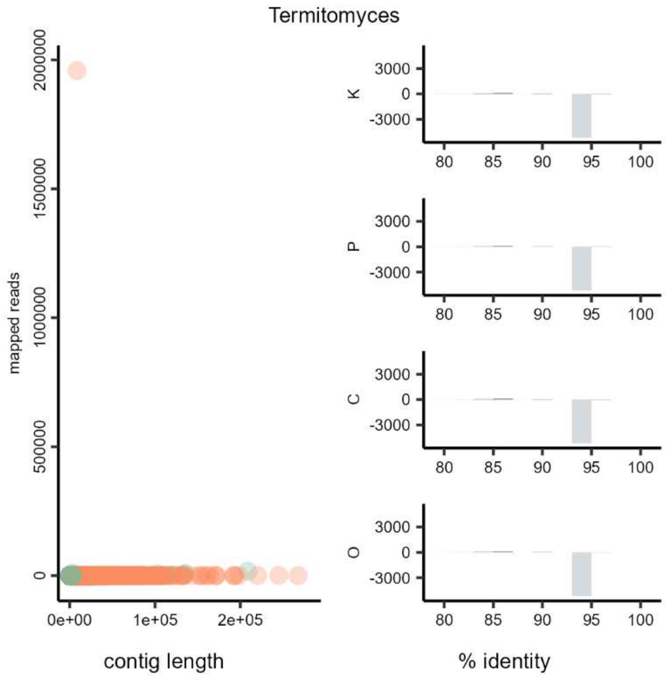

Figure 66. Left: number of mapped reads *vs*. contig length. Right: mirrored histograms of the sequence identity of aligned reads (ARs) with BLAST best-scoring sequence pairs (BSPs) within (top) or outside (bottom) the kingdom (K), phylum (P), class (C), and order (O) ranks of *Termitomyces*. BSPs are shown for the 100 consensus sequences with the highest depth of coverage. ARs are the number of reads covering a given consensus sequence query and are given as *n*-fold differences.

Nuclear genome assemblies for *T. eurrhizus* (85.9 Mb, N50: 6 kb) and *T. heimii* (56.9 Mb, N50: 26 kb), in addition to six mitochondrial genomes (103 to 209 kb) from unspecified species, were included in the Kraken 2 reference database.

90% of the *Termitomyces*-classified reads mapped to their reference database sequences. Of these, 94% mapped to *T. heimii* assembly, indicated in orange in Fig 67, with 1,958,210 reads (92%) mapping to a single 8,610 bp contig. Reads mapped to the *T. eurrhizus* assembly or the six mitochondrial genomes are shown in green in Fig. 67.

The 3,000 bp consensus query from the extremely high-depth *T. heimii* contig aligned with a 28S ribosomal rRNA sequence from a Cecidomyiidae midge with 94% sequence identity (Table 94). Larvae of some cecidomyiid midges inhabit fungal fruiting bodies, and a resident of the *T. heimii* sporocarp sample (Li et al. 2018) may have inadvertently contributed DNA to the sequencing library. This contig was the only source of non-fungal BSPs detected among the 67 unique contigs represented by the 100 consensus queries and likely explains 92% of all *Termitomyces*-classified reads. Similar results were found by directly querying 100 random paired-end reads against the nt database (Table 95).

Table 94. Distribution of BLAST best-scoring sequence pairs (BSPs) for the 100 consensus sequences with the highest depth of coverage by taxonomic rank. Aligned reads (ARs) are the number of reads that contributed to a given consensus sequence query. %ARs are scaled by the sum of reads comprising queries with at least one BSP. Results are shown for taxonomic ranks comprising >5% of ARs and the hierarchy is collapsed to the lowest rank with identical results. %ID is

mean percent sequence identity, length is the mean alignment length, e-value is the mean expect value, and bitscore is the mean bitscore.

kingdom	phylum	class	order	family	%ARs	%ID	length	e-value	bitscore
Metazoa	Arthropoda	Insecta	Diptera	Cecidomyiidae	95	94	3,004	0.0	4,627
Fungi	Basidiomycota	Agaricomycetes			5	86	394	6^{-33}	484

Queries from the mitochondrial genomes, along with putative mitochondrial contigs in the two nuclear assemblies, had BSPs spanning eight different Agaricomycetes orders. Similarly, 8% of alignments with the 100 randomly selected reads had BSPs distributed over seven Agaricales families. This diffuse taxonomic distribution of BSPs may indicate a relatively conserved sequenced that is consistently classified as *Termitomyces* by Kraken 2, but we did not investigate if the low resolution of the BLAST alignments could be explained by the absence of informative polymorphism in these reads or by the composition of the nt database. In either case, *ca*. 95% of the *Termitomyces*-classified reads most likely originated from insects, making this genus a false positive.

Table 95. Distribution of BLAST best-scoring sequence pairs (BSPs) by taxonomic rank for 100 randomly-selected pair-end reads. Results are shown for taxonomic ranks comprising >5% of reads and the hierarchy is collapsed to the lowest rank with identical results. %ID is mean percent sequence identity, length is the mean alignment length, e-value is the mean expect value, and bitscore is the mean bitscore.

kingdom	phylum	class	order	family	%BSPs	%ID	length	e-value	bitscore
Metazoa	Arthropoda	Insecta	Diptera		91	95	135	8^{-28}	213
Metazoa	Arthropoda	Insecta	Diptera	Cecidomyiidae	83	95	136	9^{-28}	214
Metazoa	Arthropoda	Insecta	Diptera	Bibionidae	5	94	115	4^{-31}	173
Fungi	Basidiomycota	Agaricomycetes	Agaricales		8	94	100	1^{-26}	154

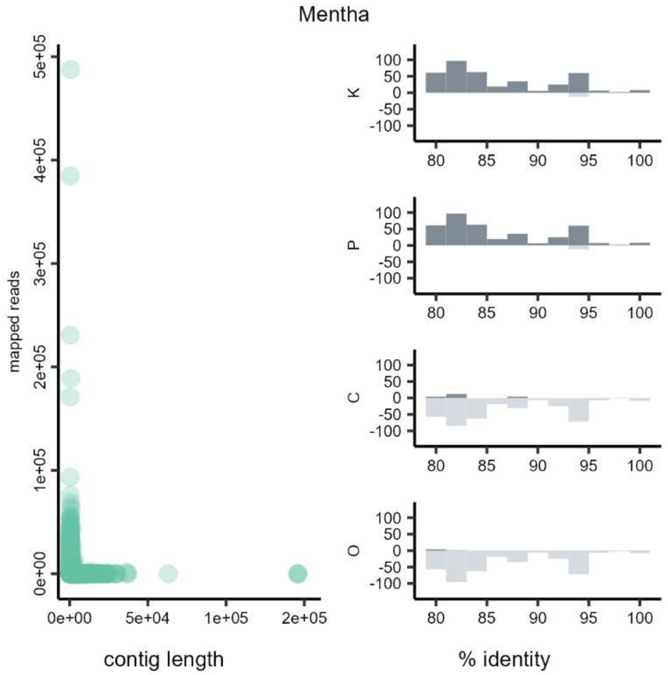

Figure 67. Left: number of mapped reads *vs*. contig length. Right: mirrored histograms of the sequence identity of aligned reads (ARs) with BLAST best-scoring sequence pairs (BSPs) within (top) or outside (bottom) the kingdom (K), phylum (P), class (C), and order (O) ranks of *Mentha*. BSPs are shown for the 100 consensus sequences with the highest depth of coverage. ARs are the number of reads covering a given consensus sequence query and are given as *n*-fold differences.

Mentha; Streptophyta; Magnoliopsida; Lamiales; Lamiaceae (21819)

Label: unlabeled
Predicted probability: 0.99
Mapped reads: 6,748,864
Mapping rate: 68%
Base pairs queried: 44,320
Unique contigs queried: 100
Unique contigs aligned: 54

Mentha was flagged for validation because no reports of this genus have been made within 40 km of the aerosol monitoring station. *M. arvensis* occurs in northern Sweden but all records are close to the Gulf of Bothnia.

A nuclear genome assembly for *M. longifolia* (329.1 Mb, N50: 3 kb) was included in the Kraken 2 database. This assembly has been substantially revised (LSBG02) and the reference contaminants we discovered may not be in the current version.

Of the 68% of successfully mapped reads, 99% mapped to contigs < 10,00 bp in length. These are all shown in Fig 68.

Most BSPs for the consensus sequence queries were within Streptophyta but 94% of reads aligned with sequences from ferns, horsetails, mosses, liverworts, or other non-seed plants (Table 96). This broad taxonomic distribution was unchanged for the single best BSP per query, suggesting either multiple contaminant taxa or that the non-seed plant sequences classified as *Mentha* are relatively conserved.

Table 96. Distribution of BLAST best-scoring sequence pairs (BSPs) for the 100 consensus sequences with the highest depth of coverage by taxonomic rank. Aligned reads (ARs) are the number of reads that contributed to a given consensus sequence query. %ARs are scaled by the sum of reads comprising queries with at least one BSP. Results are shown for taxonomic ranks comprising >5% of ARs and the hierarchy is collapsed to the lowest rank with identical results. %ID is mean percent sequence identity, length is the mean alignment length, e-value is the mean expect value, and bitscore is the mean bitscore.

kingdom	phylum	class	order	family	%ARs	%ID	length	e-value	bitscore
Viridiplantae	Streptophyta				97	86	389	2^{-28}	456
Viridiplantae	Streptophyta	Polypodiopsida			44	86	429	1^{-30}	508
Viridiplantae	Streptophyta	Polypodiopsida	Polypodiales		11	83	329	7^{-30}	323
Viridiplantae	Streptophyta	Polypodiopsida	Ophioglossales	Ophioglossaceae	11	83	406	3^{-50}	414
Viridiplantae	Streptophyta	Polypodiopsida	Psilotales	Psilotaceae	8	84	414	1^{-44}	435
Viridiplantae	Streptophyta	Polypodiopsida	Equisetales	Equisetaceae	7	95	542	2^{-60}	863
Viridiplantae	Streptophyta	Polypodiopsida	Polypodiales	Dryopteridaceae	6	83	328	5^{-49}	324

kingdom	phylum	class	order	family	%ARs	%ID	length	e-value	bitscore
Viridiplantae	Streptophyta	Bryopsida			22	88	396	1^{-53}	505
Viridiplantae	Streptophyta	Bryopsida	Hypnales		11	86	396	7^{-62}	468
Viridiplantae	Streptophyta	Bryopsida	Hypnales	Neckeraceae	9	89	355	2^{-114}	453
Viridiplantae	Streptophyta	Marchantiopsida			9	86	416	5^{-34}	481
Viridiplantae	Streptophyta	Marchantiopsida	Marchantiales		8	86	409	5^{-34}	474
Viridiplantae	Streptophyta	Marchantiopsida	Marchantiales	Ricciaceae	5	87	459	5^{-94}	539
Viridiplantae	Streptophyta	Andreaeopsida	Andreaeales	Andreaeaceae	6	84	314	2^{-27}	346

BSPs for 100 randomly selected paired end reads were similarly distributed among non-seed plants but with a larger fraction concentrated in the Ophioglossales and Equisetales (Table 97). These results unambiguously show the *Mentha*-classified reads mostly originated from a non-seed plant and, consequently, *Mentha* is a false-positive genus.

When we compared pairwise log-ratio variances to identify groups of taxa with similar temporal trends, *Mentha* was placed in the *Picea*-dominated cluster, which also included *Equisetum* (Polypodiopsida: Equisetales), *Ptilidium* (Polypodiopsida: Psilotales), *Botrychium* (Polypodiopsida: Ophioglossales), and nine genera of mosses (Bryopsida). This means that, despite being a false positive, the *Mentha* was analyzed as part of a larger group that may include the actual source organisms of these reads.

Table 97. Distribution of BLAST best-scoring sequence pairs (BSPs) by taxonomic rank for 100 randomly-selected pair-end reads. Results are shown for taxonomic ranks comprising >5% of reads and the hierarchy is collapsed to the lowest rank with identical results. %ID is mean percent sequence identity, length is the mean alignment length, e-value is the mean expect value, and bitscore is the mean bitscore.

kingdom	phylum	class	order	family	%BSPs	%ID	length	e-value	bitscore
Viridiplantae	Streptophyta				94	88	142	1^{-28}	176
Viridiplantae	Streptophyta	Polypodiopsida			46	86	145	2^{-28}	165
Viridiplantae	Streptophyta	Polypodiopsida	Ophioglossales	Ophioglossaceae	15	88	136	3^{-28}	176
Viridiplantae	Streptophyta	Polypodiopsida	Equisetales	Equisetaceae	14	84	147	3^{-28}	161
Viridiplantae	Streptophyta	Polypodiopsida	Psilotales	Psilotaceae	6	85	150	6^{-32}	167
Viridiplantae	Streptophyta	Polypodiopsida	Salviniales	Salviniaceae	6	83	146	6^{-29}	146
Viridiplantae	Streptophyta	Sphagnopsida	Sphagnales	Sphagnaceae	15	87	134	4^{-30}	160
Viridiplantae	Streptophyta	Bryopsida			15	97	136	2^{-30}	232
Viridiplantae	Streptophyta	Bryopsida	Splachnales		6	99	144	1^{-55}	255
Viridiplantae	Streptophyta	Bryopsida	Bartramiales	Bartramiaceae	5	96	126	5^{-30}	204
Viridiplantae	Streptophyta	Lycopodiopsida	Lycopodiales	Lycopodiaceae	9	87	151	3^{-38}	187
Fungi	Basidiomycota	Agaricomycetes	Agaricales	Omphalotaceae	6	87	127	4^{-28}	139

References

Alström P, Ericson PGP, Olsson U, Sundberg P. 2006. Phylogeny and classification of the avian superfamily Sylvioidea. Molecular Phylogenetics and Evolution 38:381-397.

Berglund H, Edman M, Ericson L. 2005. Temporal variation of wood-fungi diversity in boreal old-growth forests: Implications for monitoring. Ecological Applications 15:970-982.

Brassac J, Blattner FR. 2015. Species-level phylogeny and polyploid relationships in *Hordeum* (Poaceae) inferred by next-generation sequencing and *in silico* cloning of multiple nuclear loci. Systematic Biology 64:792-808.

Dong S, Liu Y. 2021. The mitochondrial genomes of bryophytes. Bryophyte Diversity and Evolution 43.

Fahrenkrog AM, Matsumoto GO, Toth K, Jokipii-Lukkari S, Salo HM, Haggman H, Benevenuto J, Munoz PR. 2022. Chloroplast genome assemblies and comparative analyses of commercially important Vaccinium berry crops. Sci Rep 12:21600.

Feng S, Ru D, Sun Y, Mao K, Milne R, Liu J. 2018. Trans-lineage polymorphism and nonbifurcating diversification of the genus Picea New Phytologist 222: 576-587. https://doi.org/10.1111/nph.15590

Gallone B, Kuyper TW, Nuytinck J. 2024. The genus Cortinarius should not (yet) be split. IMA Fungus 15:24.

Gonzalez A, Vázquez-Baeza Y, Pettengill JB, Ottesen A, McDonald D, Knight R. 2016. Avoiding Pandemic Fears in the Subway and Conquering the Platypus. mSystems 1.

Han M-L, Chen Y-Y, Shen L-L, Song J, Vlasák J, Dai Y-C, Cui B-K. 2016. Taxonomy and phylogeny of the brown-rot fungi: Fomitopsis and its related genera. Fungal Diversity 80:343-373.

Jin W-T, Gernandt DS, Wehenkel C, Xia X-M, Wei X-X, Wang X-Q. 2021. Phylogenomic and ecological analyses reveal the spatiotemporal evolution of global pines. Proceedings of the National Academy of Sciences 118:e2022302118.

Justo A, Hibbett DS. 2011. Phylogenetic classification of Trametes (Basidiomycota, Polyporales) based on a five–marker dataset. Taxon 60:1567-1583.

Justo A, Miettinen O, Floudas D, Ortiz-Santana B, Sjokvist E, Lindner D, Nakasone K, Niemela T, Larsson KH, Ryvarden L, et al. 2017. A revised family-level classification of the Polyporales (Basidiomycota). Fungal Biol 121:798-824.

Kim HT, Kim K-J. 2014. Chloroplast Genome Differences between Asian and American Equisetum arvense (Equisetaceae) and the Origin of the Hypervariable trnY-trnE Intergenic Spacer. PLOS ONE 9:e103898.

Knoop V. 2013. Plant mitochondrial genome peculiarities evolving in the earliest vascular plant lineages. Journal of Systematics and Evolution 51:1-12.

Leonard JA, Shanks O, Hofreiter M, Kreuz E, Hodges L, Ream W, Wayne RK, Fleischer RC. 2007. Animal DNA in PCR reagents plagues ancient DNA research. Journal of Archaeological Science 34:1361-1366.

Li H, Wu S, Ma X, Chen W, Zhang J, Duan S, Gao Y, Kui L, Huang W, Wu P, et al. 2018. The Genome Sequences of 90 Mushrooms. Sci Rep 8:9982.

Li L, Milesi P, Tiret M, Chen J, Sendrowski J, Baison J, Chen Z-q, Zhou L, Karlsson B, Berlin M, et al. 2022. Teasing apart the joint effect of demography and natural selection in the birth of a contact zone. New Phytologist 236:1976-1987.

Liimatainen K, Kim JT, Pokorny L, Kirk PM, Dentinger B, Niskanen T. 2022. Taming the beast: a revised classification of Cortinariaceae based on genomic data. Fungal Diversity 112:89-170.

Lorenz C, Alves JM, Foster PG, Suesdek L, Sallum MAM. 2021. Phylogeny and temporal diversification of mosquitoes (Diptera: Culicidae) with an emphasis on the Neotropical fauna. Systematic Entomology 46:798-811.

Lundström JO, Schäfer ML, Hesson JC, Blomgren E, Lindström A, Wahlqvist P, Halling A, Hagelin A, Ahlm C, Evander M. 2013. The geographic distribution of mosquito species in Sweden. Journal of the European Mosquito Control Association 31:21-35.

Nguyen NH, Vellinga EC, Bruns TD, Kennedy PG. 2016. Phylogenetic assessment of global Suillus ITS sequences supports morphologically defined species and reveals synonymous and undescribed taxa. Mycologia 108:1216-1228.

Nortcutt CG, Jiang L, Chuang IL. 2022. Confident learning: estimating uncertainty in dataset labels. Preprint at https://doi.org/10.48550/arXiv.1911.00068

Reinert JF. 2000. New classification for the composite genus Aedes (Diptera: Culicidae: Aedini), elevation of subgenus Ochlerotatus to generic rank, reclassification of the other subgenera, and notes on certain subgenera and species. Journal of the American Mosquito Control Association-Mosquito News 16:175-188.

Reisen W. 2016. Update on journal policy of Aedine mosquito genera and subgenera. Journal of Medical Entomology 53:249-249.

Richardson AO, Rice DW, Young GJ, Alverson AJ, Palmer JD. 2013. The “fossilized” mitochondrial genome of *Liriodendron tulipifera*: ancestral gene content and order, ancestral editing sites, and extraordinarily low mutation rate. BMC Biology 11:1-17.

Ronquist F, Forshage M, Häggqvist S, Karlsson D, Hovmöller R, Bergsten J, Holston K, Britton T, Abenius J, Andersson B, et al. 2020. Completing Linnaeus's inventory of the Swedish insect fauna: Only 5,000 species left? PLOS ONE 15:e0228561.

Sangster G, Alström P, Forsmark E, Olsson U. 2010. Multi-locus phylogenetic analysis of Old World chats and flycatchers reveals extensive paraphyly at family, subfamily and genus level (Aves: Muscicapidae). Molecular Phylogenetics and Evolution 57:380-392.

Skogsstyrelsen 2020. Forest management in Sweden: Current practice and historical background. Rapport 2020/4. Available online. https://www.skogsstyrelsen.se/globalassets/om-oss/rapporter/rapporter-20222021202020192018/rapport-2020-4-forest-management-in-sweden.pdf. Accessed 15 September 2024.

Sullivan, AR. (2020). A forest dark : an evolutionary history of Norway spruce (PhD dissertation, Umeå University). Retrieved from https://urn.kb.se/resolve?urn=urn:nbn:se:umu:diva-168048

Sullivan AR, Eldfjell Y, Schiffthaler B, Delhomme N, Asp T, Hebelstrup KH, Keech O, Öberg L, Møller IM, Arvestad L, et al. 2019. The mitogenome of Norway spruce and a reappraisal of mitochondrial recombination in plants. Genome Biology and Evolution 12:3586-3598.

Sullivan AR, Schiffthaler B, Thompson SL, Street NR, Wang X-R. 2017. Interspecific plastome recombination reflects ancient reticulate evolution in Picea (Pinaceae). Molecular Biology and Evolution 34:1689-1701.

Turland, N. J., Wiersema, J. H., Barrie, F. R., Greuter, W., Hawksworth, D. L., Herendeen, P. S., Knapp, S., Kusber, W.-H., Li, D.-Z., Marhold, K., May, T. W., McNeill, J., Monro, A. M., Prado, J., Price, M. J. & Smith, G. F. (eds.) 2018: *International Code of Nomenclature for algae, fungi, and plants (Shenzhen Code) adopted by the Nineteenth International Botanical Congress Shenzhen, China, July 2017*. Regnum Vegetabile 159. Glashütten: Koeltz Botanical Books. DOI https://doi.org/10.12705/Code.2018

Vizzini A, Alvarado P, Consiglio G, Marchetti M, Xu J. 2024. Family matters inside the order Agaricales: systematic reorganization and classification of incertae sedis clitocyboid, pleurotoid and tricholomatoid taxa based on an updated 6-gene phylogeny. Stud Mycol 107:67-148.

Wang GS, Cai Q, Hao YJ, Bau T, Chen ZH, Li MX, David N, Kraisitudomsook N, Yang ZL. 2024. Phylogenetic and taxonomic updates of Agaricales, with an emphasis on Tricholomopsis. Mycology 15:180-209.

Wang M, Zhang L, Zhang Z, Li M, Wang D, Zhang X, Xi Z, Keefover-Ring K, Smart LB, DiFazio SP, et al. 2020. Phylogenomics of the genus Populus reveals extensive interspecific gene flow and balancing selection. New Phytol 225:1370-1382.

Wang XW, Liu SL, Zhou LW. 2023. An updated taxonomic framework of Hymenochaetales (Agaricomycetes, Basidiomycota). Mycosphere 14:452-496.

Woloszynska M. 2010. Heteroplasmy and stoichiometric complexity of plant mitochondrial genomes-though this be madness, yet there's method in't. Journal of Experimental Botany 61:657-671.
