# Supplementary material for: Airborne eDNA captures three decades of ecosystem biodiversity
Source: Nat Commun. 2025 Dec 18;16:11281. doi: 10.1038/s41467-025-67676-7 (PMC12717267; doi:10.1038/s41467-025-67676-7)
Supplement: Supplementary file 16 — Reporting Summary [file 41467_2025_67676_MOESM16_ESM.pdf]

nature portfolio

Corresponding author(s): Per Stenberg

Last updated by author(s): Nov 12, 2025

Reporting Summary

Nature Portfolio wishes to improve the reproducibility of the work that we publish. This form provides structure for consistency and transparency in reporting. For further information on Nature Portfolio policies, see our Editorial Policies and the Editorial Policy Checklist.

Statistics

For all statistical analyses, confirm that the following items are present in the figure legend, table legend, main text, or Methods section.

n/a	Confirmed	
☐	☒	The exact sample size (n) for each experimental group/condition, given as a discrete number and unit of measurement
☐	☒	A statement on whether measurements were taken from distinct samples or whether the same sample was measured repeatedly
☐	☒	The statistical test(s) used AND whether they are one- or two-sided<br>*Only common tests should be described solely by name; describe more complex techniques in the Methods section.*
☐	☒	A description of all covariates tested
☐	☒	A description of any assumptions or corrections, such as tests of normality and adjustment for multiple comparisons
☐	☒	A full description of the statistical parameters including central tendency (e.g. means) or other basic estimates (e.g. regression coefficient) AND variation (e.g. standard deviation) or associated estimates of uncertainty (e.g. confidence intervals)
☐	☒	For null hypothesis testing, the test statistic (e.g. F, t, r) with confidence intervals, effect sizes, degrees of freedom and P value noted<br>*Give P values as exact values whenever suitable.*
☐	☒	For Bayesian analysis, information on the choice of priors and Markov chain Monte Carlo settings
☒	☐	For hierarchical and complex designs, identification of the appropriate level for tests and full reporting of outcomes
☒	☐	Estimates of effect sizes (e.g. Cohen's d, Pearson's r), indicating how they were calculated

Our web collection on statistics for biologists contains articles on many of the points above.

Software and code

Policy information about availability of computer code

Data collection	No software was used to collect data for this study.

Data analysis	Land cover: ArcGIS (v. 10.3)<br>Catchment area: PELLO and R packages 'boot' (v. 1.3-28.1) and 'nlme' (v. 3.1-163)<br>Adapter trimming: cutadapt (v. 2.0)<br>Mapping: BBmap (v. 38.69), bedtools v.2.18, samtools v.1.20, and Hisat2 (v. 2.2.1)<br>Read classification: Kraken 2 (v. 2.0.8-beta) and StringMeUp (v. 0.1.4)<br>Zero replacement: R package 'zCompositions' (v. 1.4.0-1)<br>Pivot coordinate log-ratio (PLR) transformations: R package 'robCompositions' (v. 2.3.1)<br>Isometric log-ratio (ILR) transformations: R package 'compositions' (v. 2.0-6)<br>Generalized linear models (GLM): R package 'statsmodels' (v. 0.11.1)<br>Redundancy analysis (RDA): R package 'vegan' (v. 2.6-4)<br>Gradient boosting machine (GBM): R package 'xgboost' (v. 1.7.5.1)<br>Blast: blast (v. 2.10.1+)<br>Climatic covariable selection: R package 'caret' (v. 6.0-93)<br>Climatic covariable clustering: R packages 'densvis' (v. 1.8.1) and 'dbscan' (v. 1.1-11)<br>eDNA abundance modeling: R package 'bsts' (v. 0.9.9)<br>Time series models convergence testing: R package 'coda' (v. 0.19-4)<br>Abundance trends from count data: R package 'MARSS' (v. 3.11.4)<br>Back-trajectories: HYSPLIT (Hybrid Single-Particle Lagrangian Integrated Trajectory) and R package 'OpenAir' (v. 2.18-2)

For manuscripts utilizing custom algorithms or software that are central to the research but not yet described in published literature, software must be made available to editors and reviewers. We strongly encourage code deposition in a community repository (e.g. GitHub). See the Nature Portfolio guidelines for submitting code & software for further information.

Data

Policy information about availability of data

All manuscripts must include a data availability statement. This statement should provide the following information, where applicable:

- Accession codes, unique identifiers, or web links for publicly available datasets
- A description of any restrictions on data availability
- For clinical datasets or third party data, please ensure that the statement adheres to our policy

Sequencing data are available through the NCBI Sequence Read Archive (SRA) under project PRJNA808200.

Research involving human participants, their data, or biological material

Policy information about studies with human participants or human data. See also policy information about sex, gender (identity/presentation), and sexual orientation and race, ethnicity and racism.

Reporting on sex and gender	This study does not involve human participants, their data, or their biological material.
Reporting on race, ethnicity, or other socially relevant groupings	This study does not involve human participants, their data, or their biological material.
Population characteristics	This study does not involve human participants, their data, or their biological material.
Recruitment	This study does not involve human participants, their data, or their biological material.
Ethics oversight	This study does not involve human participants, their data, or their biological material.

Note that full information on the approval of the study protocol must also be provided in the manuscript.

Field-specific reporting

Please select the one below that is the best fit for your research. If you are not sure, read the appropriate sections before making your selection.

☐ Life sciences ☐ Behavioural & social sciences ☒ Ecological, evolutionary & environmental sciences

For a reference copy of the document with all sections, see nature.com/documents/nr-reporting-summary-flat.pdf

Ecological, evolutionary & environmental sciences study design

All studies must disclose on these points even when the disclosure is negative.

Study description	DNA sequencing of archived air filter samples from a single aerosol sampling station in northern Sweden. The archive consists of weekly filters collected from 1960's and onward. Here we sequence 380 filters between 1974 and 2008. We selected filters installed in even numbered years and from weeks with a mean temperature above 0 degrees centigrades. Sequence reads were taxonomically classified using available reference genomes and time series of relative abundances were reconstructed.

Research sample	The air filters are made from glass fiber and are designed to collect particles down to 0.2 micrometers in size. When installed they measure 600 x 600 mm. After being taken down, they are rolled and compressed into a circular disc with a diameter of 60 mm. Compressed air filters are stored individually in airtight containers.
Sampling strategy	Three punches were randomly taken from each compressed filter and DNA extracted, after which the three samples were pooled. Approximately 5% of any given filter was used for DNA extraction.
Data collection	Data was collected at a single location (67.84 N, 20.42 E). Each sample represents more than 100,000 cubic meters of ground-level air that were actively passed through a silica-based filter for seven days. Every week the filter was replaced. Weekly filters from even years between the years 1974 and 2008 with a mean temperature above zero degrees Celsius were initially included in the study. Weekly samples which did not generate more that 10 ng DNA were subsequently removed from analysis. In total 380 weekly samples were analyzed in the study.
Timing and spatial scale	Each filter was installed for a duration of one week. The spatial scale of the origin of the aerosol particles that end up at the filter station were estimated using particle distribution models.
Data exclusions	Filter samples that failed to produce sufficient DNA (10 ng or more) for sequencing library preparation were excluded.
Reproducibility	Since we sequenced single archived air filters, replicates were not possible to produce. Multiple punches from each compressed air filter were taken to get a representative sample of each filter.
Randomization	The order of the DNA extractions were randomized. The sequencing order was randomized.
Blinding	All air filters were randomly coded before DNA extraction and subsequent sequencing.

Did the study involve field work? ☐ Yes ☒ No

Reporting for specific materials, systems and methods

We require information from authors about some types of materials, experimental systems and methods used in many studies. Here, indicate whether each material, system or method listed is relevant to your study. If you are not sure if a list item applies to your research, read the appropriate section before selecting a response.

Materials & experimental systems

n/a	Involved in the study
☒	☐ Antibodies
☒	☐ Eukaryotic cell lines
☒	☐ Palaeontology and archaeology
☒	☐ Animals and other organisms
☒	☐ Clinical data
☒	☐ Dual use research of concern
☒	☐ Plants

Methods

n/a	Involved in the study
☒	☐ ChIP-seq
☒	☐ Flow cytometry
☒	☐ MRI-based neuroimaging

Plants

Seed stocks	We did not use any seed stocks.
Novel plant genotypes	We did not produce any novel plant genotypes.
Authentication	No authentication procedures were applied since no seed stocks were used, nor were any novel plant genotypes generated.
